# Computational analysis for identification of the extracellular matrix molecules involved in endometrial cancer progression

**DOI:** 10.1371/journal.pone.0231594

**Published:** 2020-04-21

**Authors:** Vijesh Kumar Yadav, Tzong-Yi Lee, Justin Bo-Kai Hsu, Hsien-Da Huang, Wei-Chung Vivian Yang, Tzu-Hao Chang

**Affiliations:** 1 The Program for Translational Medicine, Graduate Institute of Biomedical Informatics, College of Medical Science and Technology, Taipei Medical University, Taipei, Taiwan; 2 Warshel Institute for Computational Biology, The Chinese University of Hong Kong, Shenzhen, Longgang District, Shenzhen, Guangdong Province, China; 3 School of Life and Health Science, The Chinese University of Hong Kong, Shenzhen, Longgang District, Shenzhen, Guangdong Province, China; 4 Department of Medical Research, Taipei Medical University Hospital, Taipei, Taiwan; 5 The PhD Program for Translational Medicine, College of Medical Science and Technology, Taipei Medical University, Taipei, Taiwan; 6 Graduate Institute of Biomedical Informatics, College of Medical Science and Technology, Taipei Medical University, Taipei, Taiwan; 7 Clinical Big Data Research Center, Taipei Medical University Hospital, Taipei, Taiwan; University of Crete, GREECE

## Abstract

Recurrence and poorly differentiated (grade 3 and above) and atypical cell type endometrial cancer (EC) have poor prognosis outcome. The mechanisms and characteristics of recurrence and distal metastasis of EC remain unclear. The extracellular matrix (ECM) of the reproductive tract in women undergoes extensive structural remodelling changes every month. Altered ECMs surrounding cells were believed to play crucial roles in a cancer progression. To decipher the associations between ECM and EC development, we generated a PAN-ECM Data list of 1516 genes including ECM molecules (ECMs), synthetic and degradation enzymes for ECMs, ECM receptors, and soluble molecules that regulate ECM and used RNA-Seq data from The Cancer Genome Atlas (TCGA) for the studies. The alterations of PAN-ECM genes by comparing the RNA-Seq expressions profiles of EC samples which have been grouped as tumorigenesis and metastasis group based on their pathological grading were identified. Differential analyses including functional enrichment, co-expression network, and molecular network analysis were carried out to identify the specific PAN-ECM genes that may involve in the progression of EC. Eight hundred and thirty-one and 241 PAN-ECM genes were significantly involved in tumorigenesis (*p*-value <1.571e-15) and metastasis (*p*-value <2.2e-16), respectively, whereas 140 genes were in the intersection of tumorigenesis and metastasis. Interestingly, 92 of the 140 intersecting PAN-ECM genes showed contrasting fold changes between the tumorigenesis and metastasis datasets. Enrichment analysis for the contrast PAN-ECM genes indicated pathways such as GP6 signaling, ILK signaling, and interleukin (IL)-8 signaling pathways were activated in metastasis but inhibited in tumorigenesis. The significantly activated ECM and ECM associated genes in GP6 signaling, ILK signaling, and interleukin (IL)-8 signaling pathways may play crucial roles in metastasis of EC. Our study provides a better understanding of the etiology and the progression of EC.

## Introduction

**Fig 1:**
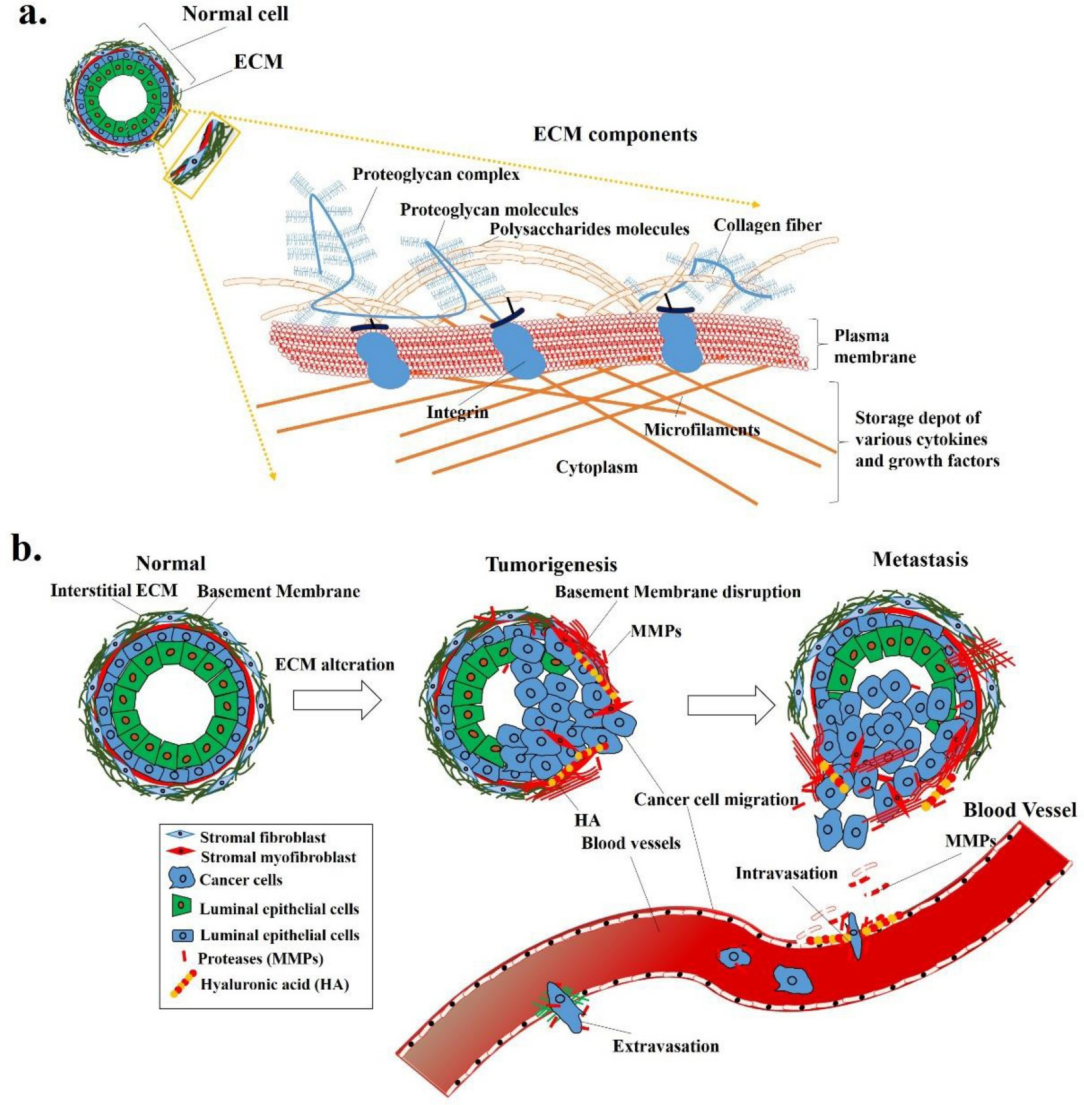
Extracellular matrix (ECM) components and their alterations during tumor development and progression. (a) ECM components, including proteoglycans, collagens, integrins, and many other substances, form a network structure, and (b) alterations of ECM networks may allow tumor cells to migrate from the original site to distant organs and develop into tumors.

Extracellular matrix (ECM), a critical component of tissues, is an interlocking mesh of fibrous proteins and polysaccharides including collagen, elastin/nidogen, laminins, and proteoglycans that form a well-organized structure in multicellular organisms [1]. The basic structure of the ECM complex, as described in Fig 1A, provides a supporting structure for cells to anchor [2]. In addition, soluble molecules, such as glycopeptidases and growth factors, are present in spaces among cells and the ECM structure, may regulate cell proliferation, differentiation, and the movement [3], as well as many secreted signaling molecule such as semaphorin III family members genes (SEMA3F) also plays there role in Endometrial cancer [4]. The ECM networks and various cytokines and growth factors create a microenvironment for cell growth and development. ECM receptors at the cell surfaces mediate 2-way signals for cells to sense the microenvironment and react to the stimuli [5]. In cancer patients, tumor cells do not grow along, and their behaviours are regulated by their own biology and also by interacting with the surrounding microenvironment [6]. Many components of ECM, like fibrous collagen and elastin create tissue stiffness [7]. Increases in collagen linearization and tissue stiffness in precancerous tissues of breast cancer were reported, and such changes increase the tumor incidence and progression [7]. Proteolysis is a significant factor that leads to metabolic reprogramming of ECM dynamics, and disruption of these control mechanisms causes ECM dysregulation and disorganization, which may contribute to malignant processes or cancer [8, 9]. The ECM paves the way for cancer cell development by creating a favourable microenvironment, more-intense stiffness, etc. [10].

Endometrial cancer (EC) is a commonly occurred malignancy of the female reproductive tract that arises from the uterus lining [11]. While the occurrence of the disease varies widely among countries, EC has become the most common female cancer in areas like North America, Europe, and middle-income developing countries such as South Africa and India [12]. In most cases, women with more-aggressive EC have higher-grade tumors, and the disease spreads from the uterus within 1 year [13]. About 75% of EC patients are diagnosed at an early stage (International Federation of Gynecology and Obstetrics (FIGO) stages I and II) and have an excellent prognosis with a 5-year survival rate of 80% [14, 15]. However, survival rates decrease in severe cases, at 52% and 27% for patients at stages III and IV, respectively [15]. EC is classified into two groups, type I and II endometrioid tumors [16]. Type I is estrogen-dependent [17], obesity is the major risk factor [18], and it has a favourable prognosis; in contrast, type II tumors occur in elderly, non-obese women, are estrogen-independent and exhibit worse outcomes [16, 19]. The most common treatment for patients with EC is surgery combined with adjuvant radiotherapy for early-stage type I EC, and combined with chemotherapy for type II EC [11]. Many studies showed that women with a family history of EC have a higher risk of developing EC in their lifetime [20]. The pathological classification of EC is based on the FIGO system [21, 22]. Stage I cancer is described as the tumor only being found in the uterus or womb and not having spread to other parts of the body. Stage II or beyond is described when a tumor has spread out of the uterus, depending on the migration site and invasion of lymph nodes.

ECM plays a critical role in the female endometrium, being involved in major remodelling changes every month. The ECM of the reproductive tract in women undergoes extensive structural remodelling for decidualization, implantation, and renewal of the endometrial layer each month, while abnormal ECM changes can contribute to processes such as endometriosis, infertility, and cancer development and metastasis [23]. The invasive property is considered as cancer cells intrude into surrounding normal tissues and metastasize. Alterations of ECM, ECM-associated molecules, and the local ECM microenvironment that can promote tumor progression are illustrated in Fig 1. Some of the ECM molecules such as aggrecan, nidogen, collagen type VIII chain α1 and type XI chain α2, were overexpressed in stage III EC [24], matrix metalloproteinase (MMP)-9, an ECM degradation enzyme, might play a vital role in uterine cancer progression [25]. Previous studies by Grabarek B et.al [26] also shows normal human dermal fibroblast cells along with JAK/STAT signaling pathways regulates the numerous miR’s expression under the influence of adalimumab therapy, indicating ECM molecules can be targeted for treatment effectiveness. However, the regulatory mechanisms of ECM and ECM-associated molecules in EC are still unclear.

**Fig 2:**
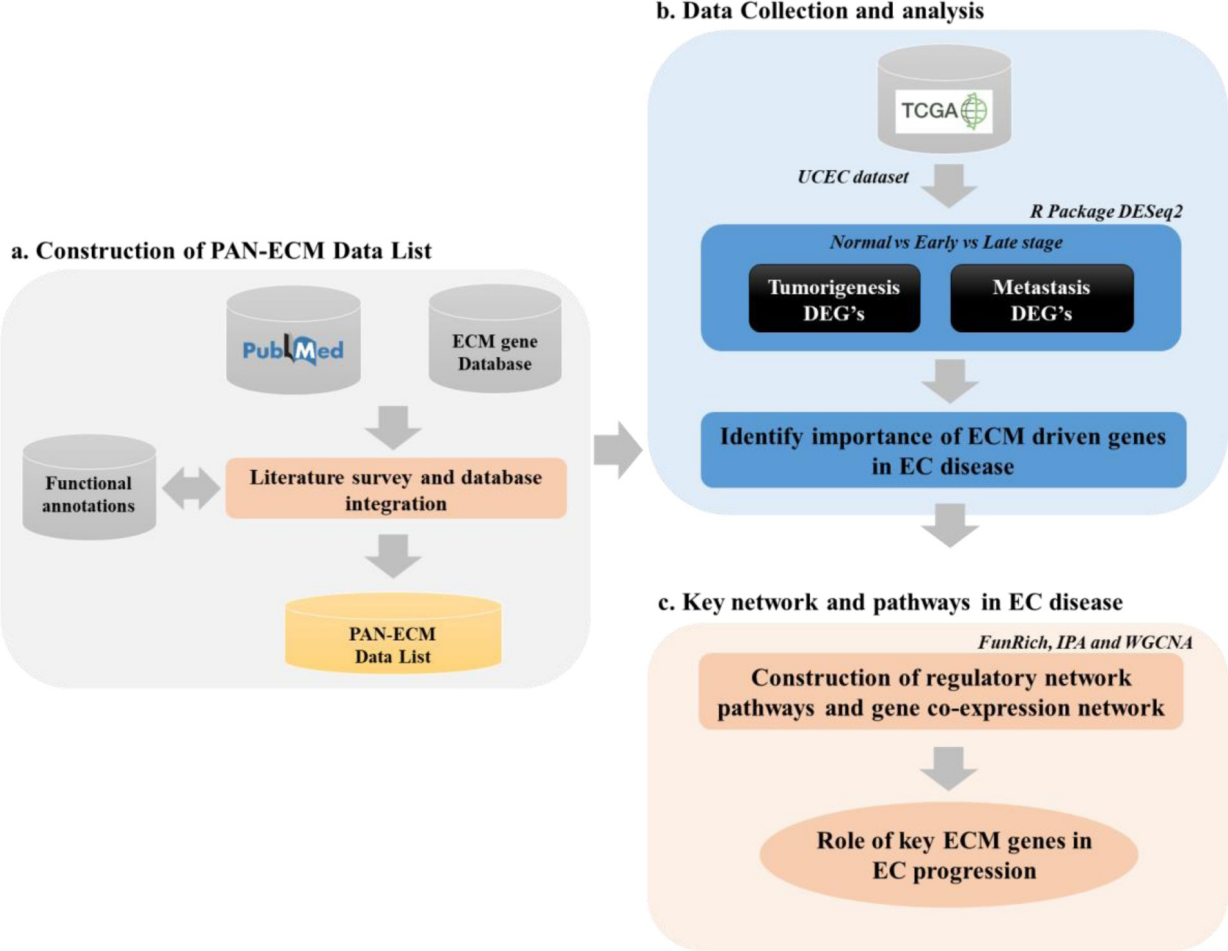
An overview and flowchart of the study. This study includes three main parts, (a) construction of the PAN-ECM Data List, (b) Identification of the ECMs, ECM-associateds molecules, the involved signaling pathways participating in EC development, and (c) Networking analyses for verification of the key ECM genes involved in EC progression.

The ECM and the ECM associated molecules involved in the microenvironment surrounding the cells seem to play important roles in tumor development; however, a comprehensive analysis of the regulatory mechanisms of ECM during EC development is still lacking. In this study, we attempted to establish a PAN-ECM Data List by collecting ECM and ECM-related genes through a literature survey and incorporation of related databases. Subsequently, we retrieved the gene expression profiles of EC samples at different stages (normal, early, and late) from The Cancer Genome Atlas-Uterine Corpus Endometrial Carcinoma (TCGA-UCEC) data, and used different bioinformatic analysis tools including DESeq2, FunRich, Ingenuity Pathway Analysis (IPA), and Weighted correlation network analysis (WGCNA) to identify and verify the key ECMs and their associates that may participate EC progression. A flow chart and an overview of the computational analysis used in this study was shown in Fig 2.

## Materials and methods

### Construction of the PAN-ECM Data List

Through a literature survey (S1 Table) and incorporation of data from related databases (MatrisomeDB; http://matrisomeproject.mit.edu/) [27], a complete PAN-ECM Data List was generated for further analysis. The Matrisome project provides information on ECM proteins which are collected by computational identification methods, and a proteomics pipeline was developed to characterize compositions of ECM tissues and tumors [28]. However, several essential molecules were lacking, such as ECM-associated regulatory factors (e.g., *SMC3*), ECM synthetic/degradation enzymes (e.g., *UXS1*, *XYLT1*, and *XYLT2*), cell adhesion molecules (e.g., *CDH1* and *CD44*), ECM microenvironment-associated molecules (e.g., *SMAD1* and *SMAD7*), etc. that were previously identified as playing critical roles in ECM regulation [29–35].

Therefore, we herein focused on developing a complete dataset for ECM research and called it the PAN-ECM Data List. The construction pipeline is depicted in Fig 2A. ECM-associated genes were collected from a literature search using such keywords as cell adhesion molecules, extracellular matrix protein, proteoglycan, collagen, glycoprotein, ECM regulator, ECM micro-environment associated, proteoglycans and secreted factors, etc. Additionally, ECM genes in MatrisomeDB were also integrated into the PAN-ECM Data List, and their functions were annotated from functional annotation databases including Gencard.

### EC data collection and analysis of differentially expressed genes (DEGs)

#### Inclusion and exclusion criteria for UCEC sample and the classification of DEGs

The RNA-Seq level 3-gene expression data for normal and solid tumors from UCEC patients were collected from TCGA [36] (Broad GDAC Firehose database: http://firebrowse.org/?cohort=UCEC). In total, 35 normal samples, and 476 tumor samples were used for this study. Based on FIGO staging for endometrial carcinoma, the tumor samples at stage IA was classified as early-stage samples, samples obtained from patients at stage II and above were classified as late-stage samples. In this study, 159 samples were late-stage and 317 samples were early-stage samples. Based on the description of the pathology in FIGO staging, EC tumors at stage IB has ≥ 50% invasion of the myometrium. To avoid the interferences, the data from the samples at stage IB patient was excluded in this study.

Both raw read counts and normalized fragments per kilobase of transcripts per million mapped reads (FPKM) values (FPKM-UQ) at the gene level were used for further analysis. Clinical XML files were also downloaded to retrieve stage and related clinical information, which was used to recognize tumorigenesis and metastasis samples, as explained in FIGO guidelines [21, 22]. Tumorigenesis DEGs were identified by comparison the DEGs from early-stage (stage IA) to normal samples, and metastatic DEGs were identified by comparing the DEGs from late-stage (stage II or above) to early-stage EC samples (stage IA).

#### Statistical analysis

Expression data were pre-processed and analyzed using DESeq2, an R package [37], and log2 fold change cutoffs of >1.5 and adjusted *p* values of <0.01 were used to determine significant DEGs. With thousands of genes tested in an RNA-Seq, multiple comparison adjustments were necessary. The Bonferroni method was applied for filtering DEGs; this controls the mean number of false positives, that can be used for multiplicity adjustment [38]. Samples at different stages were taken for a comparative analysis to identify tumorigenesis- and metastasis-associated DEGs during EC development, as illustrated in Fig 2B. A Chi-squared test was applied to test whether ECM genes were significantly associated with EC tumorigenesis or metastasis.

### Analysis of ECM regulatory mechanisms during EC development

To identify potential functions and regulatory roles of the ECM during EC development, as shown in Fig 2C, three different network analytical approaches were applied: a canonical pathway network analysis, molecular interaction network analysis, and co-expression network analysis. The canonical pathway network analysis was performed by using an Ingenuity Pathway Analysis (IPA) [39], that uses the popular activation z-score analytical method. It was proposed by Kraèmer et al. in 2014 [39], and it measures activation states, (either increased or decreased) of pathways affected by DEGs. We used a statistical approach to define a quantitative z-score, which determines whether a biological function has significantly more “increased” predictions than “decreased” predictions (z-score >0) or *vice versa* (z-score <0). In general practice, an absolute z-score of >2 or <-2 may be significant. A molecular interaction network analysis was performed using FunRich [40] to generate networks based on predicted or experimentally validated interactions, such as protein-protein interactions and gene-gene regulation to identify critical ECM-involved networks or critical ECM hub genes. The coexpression network analysis was performed using WGCNA, an R package [41], for EC samples. The BlockwiseModules function was applied to construct gene coexpression hierarchical clustering, and dynamic tree cut methods were used to identify co-expression networks.

## Results

### PAN-ECM Data List

**Fig 3:**
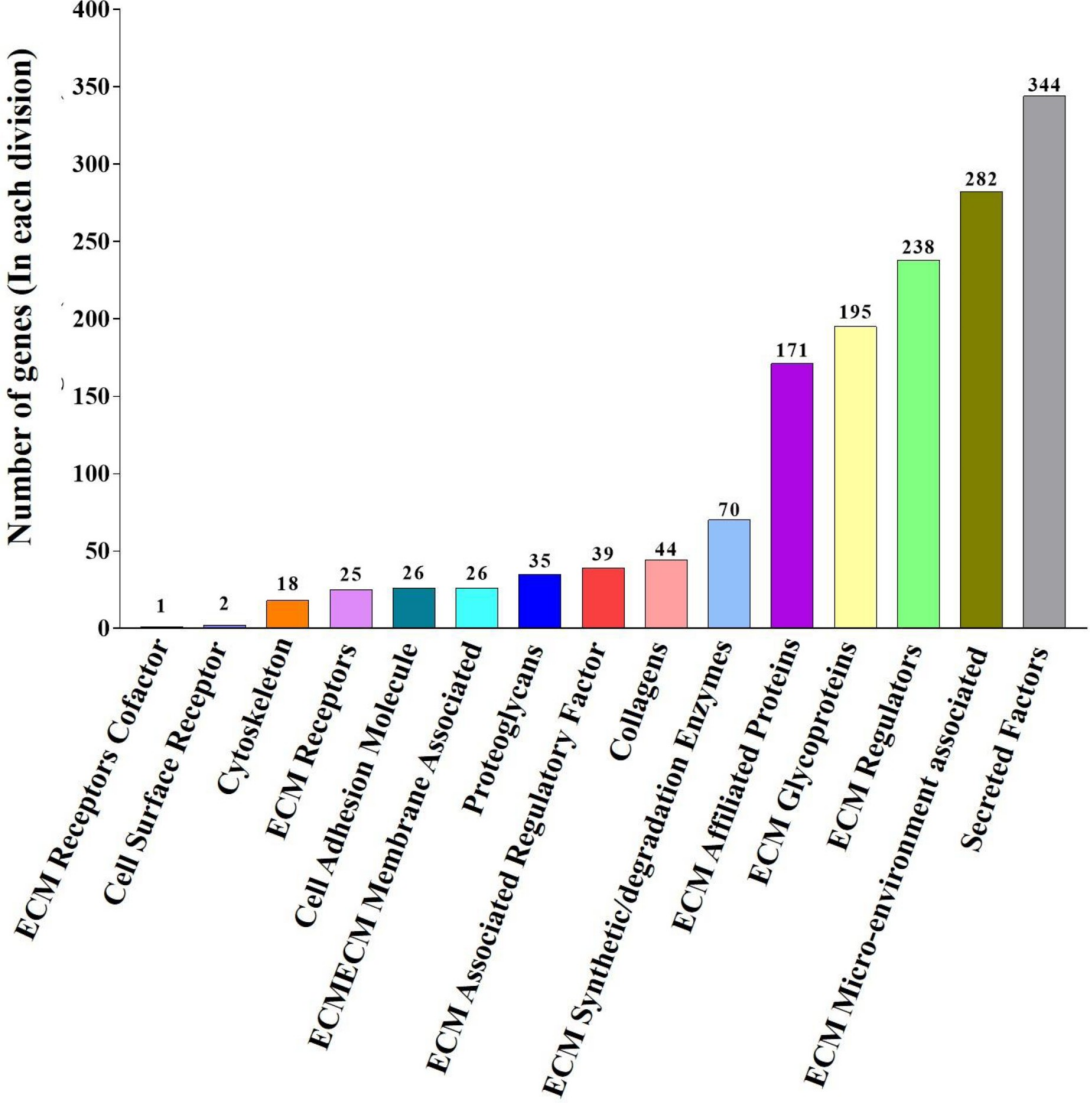
PAN-ECM Data List description generated by a literature search. Bar chart indicated the distribution of total 1516 PAN-ECM genes in the 15 classified categories. Numbers of the PAN-ECM genes in each category are shown at the top of each bar.

Based on a literature survey, a list of ECM and ECM-associated molecules including 1516 ECM-related genes, called as PAN-ECM Data List was generated **(**S1 Table**)**. The PAN-ECM genes were then classified into 15 categories according to their biological functions and localizations: cell surface receptors, cytoskeleton, cell adhesion molecules, collagens, ECM receptor cofactors, ECM receptors, ECM, ECM-associated regulatory factors, ECM synthetic/degradation enzymes, ECM-affiliated proteins, ECM glycoproteins, ECM regulators, ECM microenvironment-associated, proteoglycans, and secreted factors. Numbers of genes in different categories of the PAN-ECM Data List are given in Fig 3.

### Tumorigenesis and metastasis DEG analysis

Total of 20,531 DEGs from EC patients was retrieved from TCGA. Background distribution of 1516 PAN-ECM genes in a total of 20,531 DEGs is 7.38%. Based on FIGO staging for endometrial carcinoma development, we investigated the expression level of the DEGs and classified the DEGs from normal endometrium as the normal group, the DEGs from EC samples at stage IA as an early-stage group, DEGs from EC samples at stage II and the above as late-stage group. By comparing the expression level, 7880 DEGs were considered to be tumorigenesis-associated (early vs. normal) and 1677 DEGs were metastasis-associated (late vs. early), respectively. In those tumorigeneses- and metastasis-associated DEGs, 831 (S2 Table**)** and 241 (S3 Table**)** DEGs were PAN-ECM genes, respectively. Interestingly, the 831 tumorigenesis-associated PAN-ECM genes of 7880 DEGs (10.54%, Chi-squared *p*<1.571e-15) and the 241 metastasis-associated PAN-ECM genes of 1677 DEGs (14.37%, Chi-square *p*<2.2e-16) were significant, as compared to the background distribution of 7.38% in the total of 20,531 DEGs. Detailed information about fold change values in correlation with tumorigenesis and metastasis and the list of 140 intersecting PAN-ECM DEGs were shown in Table 1. These PAN-ECM genes were separated into four different categories according to their fold changes as up/up (37 genes), up/down (35 genes), down/up (57 genes), and down/down (11 genes) in tumorigenesis and metastasis, respectively. It indicates that 48 PAN-ECM genes were either up/up or down/down throughout all stages during EC development, whereas 92 PAN-ECM genes were expressed in contrasting direction of fold change (up/down or down/up) at tumorigenesis and metastasis stages.

**Table 1:** The expression of 140 intersecting PAN-ECM DEGs in EC at tumorigenesis and metastasis stages.

Up/Up Genes (37^#^) (FC_T*, FC_M^†^)	Up/Down Genes (35^#^) (FC_T*, FC_M^†^)	Down/Up Genes (57^#^) (FC_T*, FC_M†)		Down/Down Genes (11^#^) (FC_T*, FC_M^†^)
ATP1A3 (1.30, 1.17)	ADAM28 (1.16, -0.72)	ADAM33 (-2.54, 1.05)	LOXL4 (-1.91, 0.59)	ADAMTSL3 (-1.97, -0.69)
C1QL1 (1.62, 1.27)	ADAMTS6 (1.41, -1.09)	ADAMTS1 (-2.36, 0.61)	MAG (-4.30, 1.48)	CLEC3B (-3.98, -0.62)
C1QL4 (4.78, 0.90)	ADAMTSL2 (1.41, -0.70)	ADAMTS3 (-2.22, 1.41)	MEGF10 (-1.87, 1.03)	DPT (-6.28, -1.04)
CAMK2N2 (1.76, 0.77)	BMPR1B (1.04, -0.59)	ADAMTSL4 (-2.36, 0.59)	NCAM1 (-4.28, 1.21)	MASP1 (-4.75, -0.62)
CCL7 (1.53, 0.84)	CCL24 (2.69, -1.37)	AGTR1 (-4.45, 1.49)	NELL2 (-1.89, 0.92)	MATN2 (-1.70, -0.68)
COL10A1 (1.42, 1.42)	CELA3A (3.42, -1.07)	ANGPT1 (-2.62, 1.06)	NTF4 (-3.87, 1.82)	NDP (-1.47, -0.68)
COL11A1 (2.44, 0.99)	CELA3B (2.05, -0.88)	ANGPTL7 (-2.52, 1.30)	PAPLN (-0.84, 0.85)	OGN (-5.52, -0.98)
COL11A2 (1.46, 0.98)	CST1 (6.09, -1.15)	CADM3 (-3.20, 1.09)	PCOLCE2 (-2.53, 0.83)	OVGP1 (-2.43, -1.81)
COL26A1 (4.72, 0.88)	CST4 (6.22, -1.96)	CCL11 (-1.74, 0.82)	POSTN (-2.72, 0.60)	PLG (-1.70, -1.64)
COL9A1 (3.51, 1.90)	CTSV (5.15, -0.77)	CHRD (-1.70, 0.94)	PRL (-1.52, 0.96)	SFRP4 (-2.05, -0.94)
COMP (1.85, 2.00)	DMBT1 (3.05, -1.43)	CHSY3 (-1.18, 0.63)	RELN (-1.98, 1.13)	VWA3B (-1.52, -0.96)
CRLF1 (1.32, 0.63)	FBLN1 (1.13, -0.68)	COL19A1 (-1.18, 0.62)	SCG2 (-1.13, 0.94)	
DCSTAMP (1.28, 0.60)	GAD1 (5.24, -0.89)	COL20A1 (-2.10, 1.51)	SEMA6D (-2.26, 0.73)	
EGFL6 (1.92, 0.66)	GDF5 (3.84, -1.05)	COL4A4 (-1.88, 1.67)	SRPX (-3.80, 0.82)	
ERBB2 (0.60, 0.73)	HHIP (1.68, -0.62)	COL6A3 (-2.20, 0.80)	SYT1 (-3.47, 0.77)	
EREG (2.84, 0.84)	IHH (2.89, -1.52)	COL6A6 (-2.89, 1.26)	TGM1 (-1.48, 0.72)	
F2 (2.89, 0.77)	IL19 (4.92, -0.89)	COL8A2 (-1.13, 0.91)	THBS2 (-2.14, 0.65)	
FGFR4 (1.83, 0.61)	ITIH2 (2.00, -0.86)	CRHBP (-4.00, 0.64)	TLL1 (-2.59, 0.63)	
FZD9 (1.49, 0.81)	LMAN1L (3.55, -1.15)	DNM3 (-1.10, 0.67)	TPO (-2.49, 0.94)	
IL11 (2.52, 1.21)	MATN1 (1.04, -0.65)	EYS (-0.79, 0.67)	WFIKKN2 (-2.37, 0.92)	
INHBE (1.30, 0.62)	MMP26 (2.47, -3.07)	FAP (-2.45, 0.69)		
ITGB6 (2.12, 0.92)	MUC13 (2.62, -0.88)	FN1 (-1.05, 0.79)		
KCP (1.75, 0.70)	MUC5AC (6.62, -0.90)	FREM1 (-2.34, 0.62)		
LIF (1.46, 1.24)	MUC5B (4.57, -1.13)	FSTL3 (-1.43, 0.60)		
MATN4 (1.92, 0.62)	PF4V1 (4.66, -1.07)	GDF6 (-2.56, 1.05)		
MMP1 (4.57, 1.05)	PLA2G10 (2.46, -0.72)	GREM1 (-2.92, 0.77)		
MMP10 (3.71, 1.14)	BDNF (1.63, -1.76)	HGF (-2.38, 0.60)		
MMP13 (4.07, 1.12)	S100A3 (2.23, -0.61)	HPSE (-1.08, 0.66)		
PRSS1 (3.87, 1.34)	S100A7 (5.31, -1.33)	IGSF10 (-2.65, 0.69)		
PRSS3 (2.75, 1.01)	SEMA3E (2.18, -0.84)	IL5 (-1.53, 1.00)		
RSPO4 (1.73, 1.28)	SERPINA11 (4.81, -1.87)	IL6 (-2.88, 1.51)		
SFTPB (2.76, 1.08)	NTF3 (1.43, -1.18)	IMPG2 (-3.78, 0.66)		
SLIT1 (1.25, 0.59)	TNFSF14 (1.29, -0.79)	ISM2 (-1.86, 0.78)		
SST (3.72, 2.93)	TPH1 (1.79, -0.84)	ITGB3 (-1.54, 1.02)		
TMPRSS15 (2.33, 0.99)	WIF1 (2.75, -1.06)	ITLN1 (-1.70, 0.87)		
TNF (2.55, 0.61)		LAMA2 (-3.10, 0.68)		
WNT7A (3.64, 0.97)		LGI2 (-3.55, 1.07)		

Total of 140 intersecting PAN-ECM genes were identified and grouped as Up/Up, Up/Down, Down/Up, Down/Down expression in EC at tumorigenesis and metastasis stages respectively.

^#^Number of genes.

*The fold change of DEGs in tumorigenesis (FC_T).

†the fold change of DEGs in metastasis (FC_M).

### Pathway analysis of tumorigenesis- and metastasis-related PAN-ECM genes

**Fig 4:**
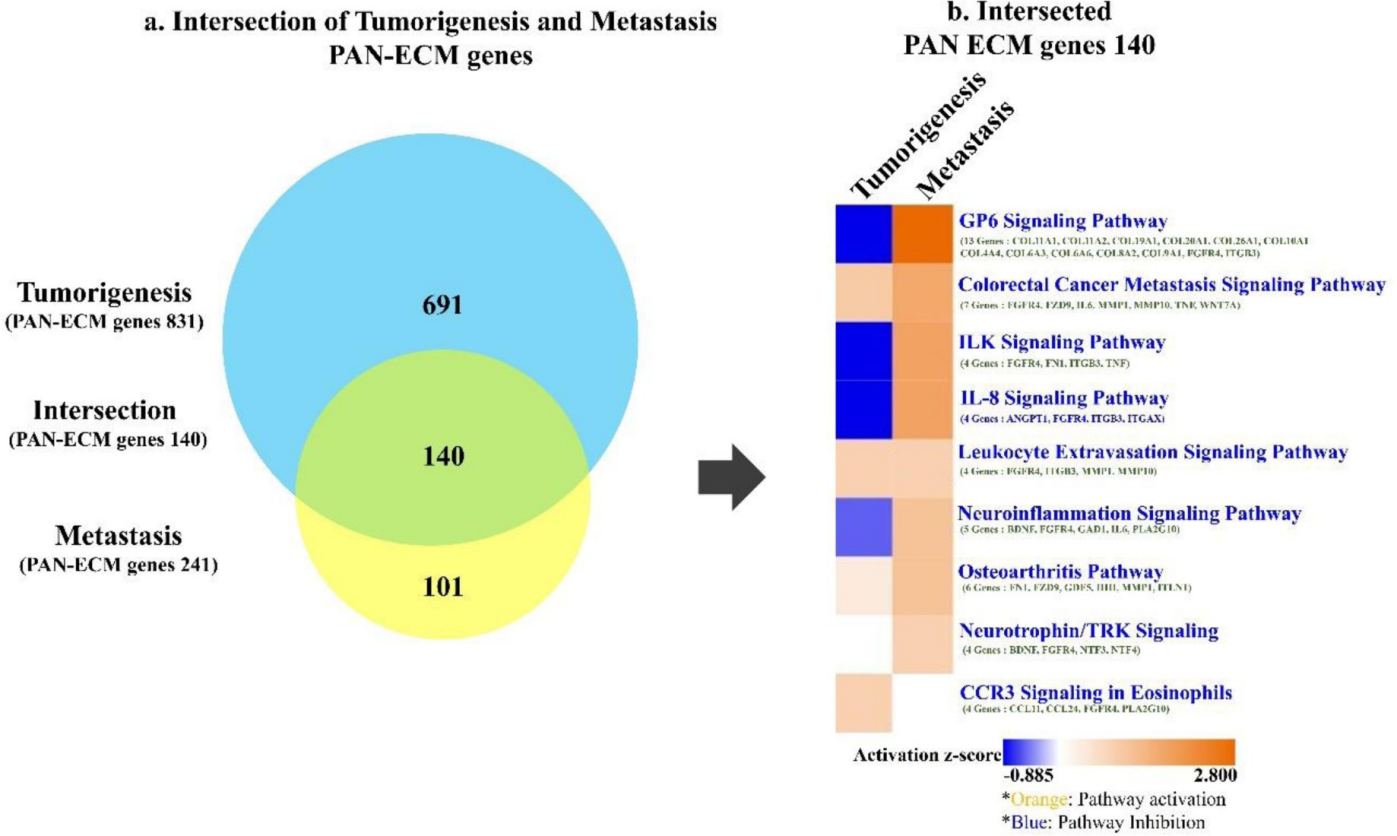
Diagram of the intersecting PAN-ECM genes and their involved canonical pathways in association with tumorigenesis and metastasis of EC by IPA analysis. (a) The diagram shows the PAN-ECM genes in the intersection between tumorigenesis and metastasis. (b) The heat map represents the intersecting PAN-ECM gene involved canonical pathways and their expressions in tumorigenesis and metastasis of EC. The orange color indicates activation of functions/pathways, blue color indicates inhibition of functions/pathways. Intensive color indicates higher absolute z-score.

IPA was applied for pathway analysis for the identified EC-associated PAN-ECM genes (Fig 4A). Numerous signaling pathways associated with the140 intersecting PAN-ECM DEGs were identified (Fig 4B). The identified pathways included GP6 signaling, IL-8 signaling, ILK signaling, neuroinflammation signaling pathway, colorectal cancer metastasis signaling, leukocyte extravasation signaling, and osteoarthritis pathway.

Interestingly, several signalling pathways were expressed in opposite fold changes at tumorigenesis and metastasis stages. GP6 signaling pathway was inhibited with the lowest z-score (-0.832) in tumorigenesis but activated in metastasis with the highest z-score (3.605). IL-8 signaling (z-score = -1), ILK signaling (z-score = -1), and neuroinflammation signaling pathways (z-score = -0.447) were also inhibited at tumorigenesis, but activated in metastasis. Moreover, pathways such as colorectal cancer metastasis signaling (z-score = 1.133 in tumorigenesis and 1.889 in metastasis), leukocyte extravasation signaling (z-score = 1 and 1), and osteoarthritis (z-scores = 0.447 and 1.341) were activated at both tumorigenesis and metastasis stages.

**Fig 5:**
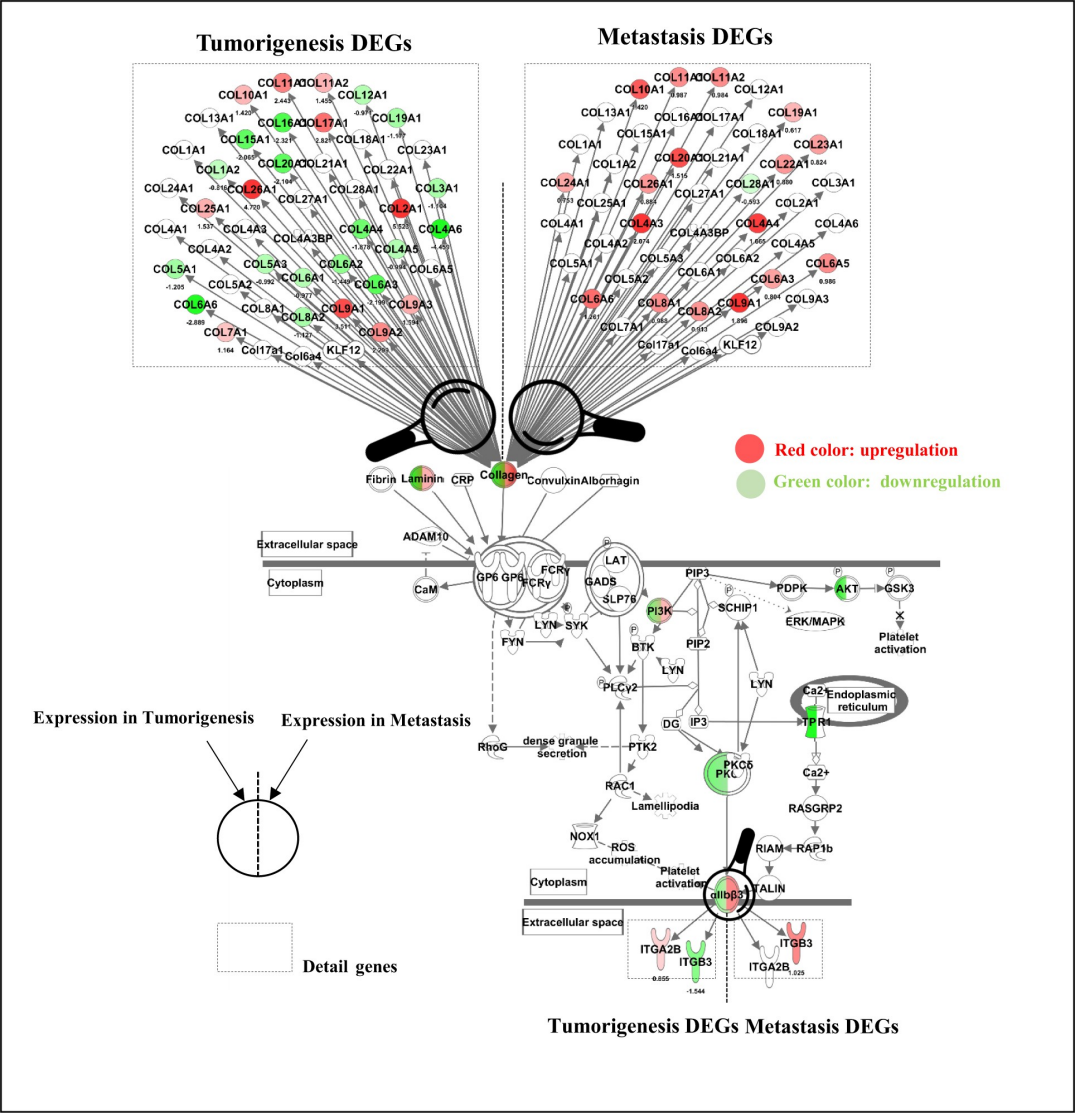
Tumorigenesis- and metastasis-associated PAN-ECM genes and their expressions inGP6 signaling pathway which were generated using QIAGEN’s Ingenuity® Pathway Analysis (IPA®, QIAGEN Redwood City) Software (27). Typical EC related tumorigenesis- and metastasis-associated PAN-ECM genes were identified. Most of them were collage-related genes and were downregulated in tumorigenesis and upregulated in metastasis of EC. The PAN-ECM genes in volved in GP6 signaling pathway with opposite expression in tumorigenesis and metastasis of EC were colored. Green colour genes indicates an inhibition z-score of -1.414. Red colour genes indicates an activation z-score of 4.146; The color is more intensive, the absolute z-score is higher.

Pathway analyses were also carried out for the identified tumorigenesis and metastasis-associated PAN-ECM genes, detailed canonical pathways generated from 831 tumorigenesis- and 241 metastasis-associated PAN-ECM genes were shown in S1 Fig. Notably, GP6 signaling pathway had the highest z-score (4.14) in the metastasis-associated PAN-ECM genes and the lowest z-score (-1.414) in the tumorigenesis-associated PAN-ECM genes, that resembles the results from the analyses for the intersecting PAN-ECM genes. The PAN-ECM genes involved in the GP6 signaling pathway were listed in Fig 5. The PAN-ECM genes including *COL4A4*, *COL6A6*, *ITGB3*, *PI3K*, and *αIIbβ3* in GP6 signaling pathway were upregulated in metastasis and downregulated in tumorigenesis.

### Functional enrichment analysis of tumorigenesis- and metastasis-associated DEGs

To depict the possible roles of the intersecting PAN-ECM genes in tumorigenesis and metastasis of EC, the disease-related and bio functions of the DEGs from IPA were enumerated in Table 2. Interestingly, the most correlated bio functions, cell movement, colony formation, cell proliferation, cell migration, adhesion of immune cells, and invasion of cells were activated with higher z-scores (>2) in metastasis and inhibited in tumorigenesis with lower z-scores (<-2). The bio functions, such as cell death and survival, cell-to-cell signaling, proliferation of epithelial cells, cellular growth, and cell cycle mitogenesis, were activated in both metastasis and tumorigenesis of EC. It suggested that the differential activation and inhibition of the bio functions of the PAN-ECM genes may lead to EC progression.

**Table 2:** Diseases and bio-functions of the intersecting PAN-ECM genes associated with differential expressions in tumorigenesis and metastasis of EC.

Diseases and bio-functions	Tumorigenesis (z-score)	Metastasis (z-score)
Cardiovascular system development and function: cell movement of endothelial cells	-2.116	0.761
Cellular growth and proliferation: colony formation of cells	-1.377	2.021
Cell death and survival, hematological system development and function: cell viability of myeloid cells	-1.217	2.177
Cellular movement, hair and skin development and function: cell movement of epithelial cell lines	-1.05	2.418
Cellular movement: migration of cells	-0.755	3.864
Cell-to-cell signaling and interaction, hematological system development and function, immune cell trafficking: adhesion of immune cells	-0.487	2.381
Cellular development, cellular growth and proliferation: proliferation of myeloma cell lines	-0.478	2.146
Free radical scavenging, small molecule biochemistry: biosynthesis of hydrogen peroxide	-0.462	2.202
Cellular development, cellular growth and proliferation: proliferation of prostate cancer cell lines	-0.349	2.195
Post-translational modification: phosphorylation of proteins	-0.34	3.06
Cell-to-cell signaling and interaction, cellular growth and proliferation: induction of cells	-0.232	2.201
Proliferation of connective tissue cells	-0.194	2.391
Cellular growth and proliferation, connective tissue development and function, tissue development	-0.128	2.402
Lipid metabolism, small molecule biochemistry: synthesis of leukotriene	-0.111	2.352
Cellular movement: invasion of cells	0	2.138
Cell-to-cell signaling and interaction, hematological system development and function: binding of lymphocytes	0.068	3.174
Cellular movement: invasion of tumor cell lines	0.113	2.72
Cell-to-cell signaling and interaction, hematological system development and function: binding of mononuclear leukocytes	0.119	2.584
Cell-to-cell signaling and interaction: binding of myeloid cells	0.221	2.697
Cellular growth and proliferation, tissue development: proliferation of epithelial cells	0.528	4.022
Cellular movement: migration of ovarian cancer cell lines	0.577	2.194
Cell signaling, molecular transport, small molecule biochemistry, vitamin and mineral metabolism: release of Ca2+	0.603	3.046
Cellular development, cellular growth and proliferation: proliferation of stem cells	0.672	2.167
Cell-to-cell signaling and interaction: binding of lymphoma cell lines	0.757	2.366
Cell signaling, molecular transport, vitamin and mineral metabolism: quantity of Ca2+	1	2.387
Cell death and survival: apoptosis of gonadal cells	1.076	2.19
Cellular growth and proliferation: expansion of cells	1.258	2.01

Activated pathways are coloured orange (z-score >2) and inhibited pathways are coloured blue (z-score <-2). The absolute z-score is higher, the color is more intensive.

### Network analysis of intersecting PAN-ECM genes between tumorigenesis and metastasis DEGs

**Fig 6:**
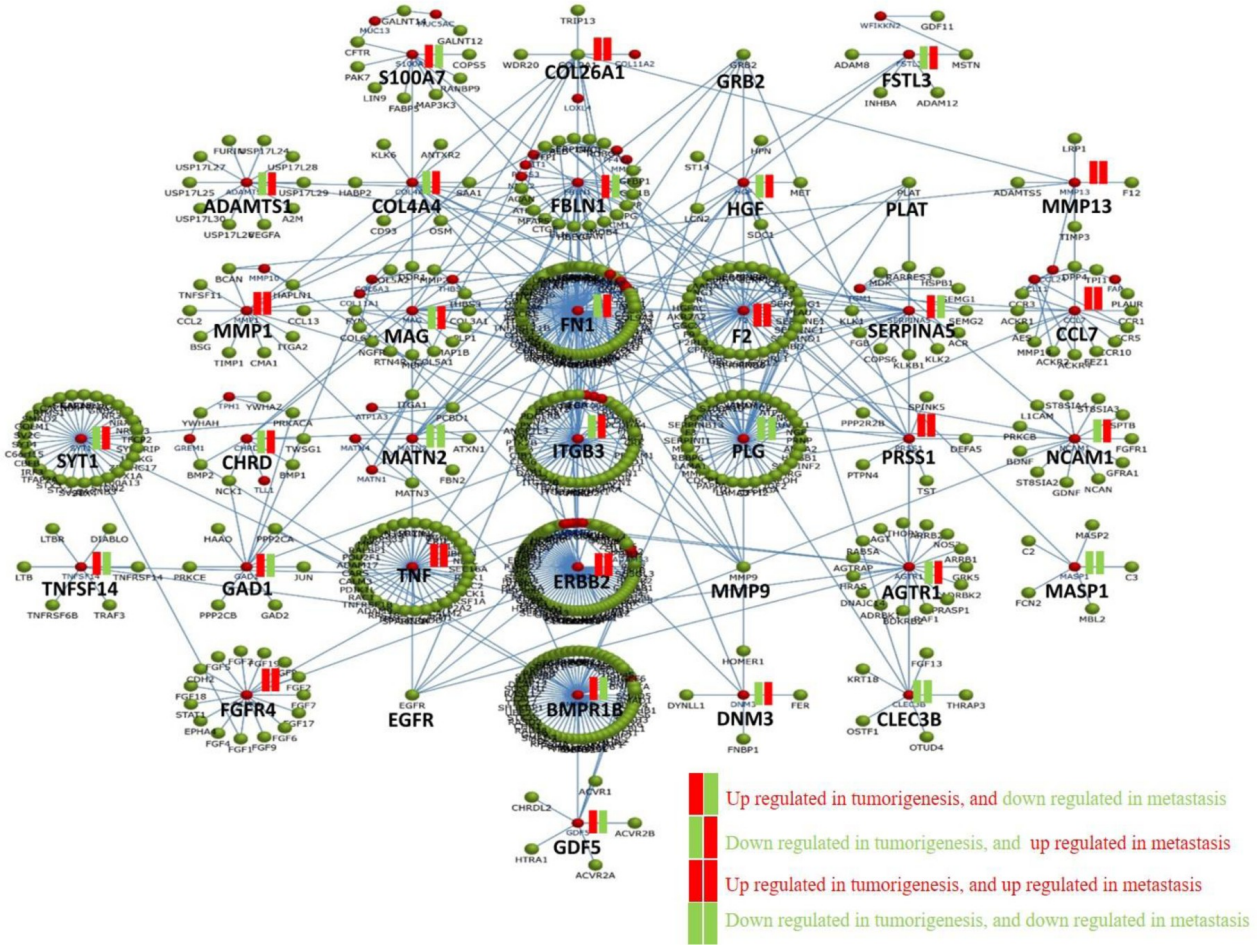
Molecular network of PAN-ECM genes that intersected in tumorigenesis and metastasis. The molecular interaction network of the intersecting PAN-ECM genes are shown. Upregulated and downregulated genes are shown in red and green, respectively. Some genes, such as S100A7, FBLN1, SERPINAS, TNFSF14,BMPR18, and GDF5 were upregulated in tumorigenesis and downregulated in metastasis. In contrast, FSTL3, ADAMTS1. COL4A4, HGF, MAG, FN1, ITGB3, NCAM1, AGTR1, and DNM3 were downregulated an upregulated in tumorigenesis and metastasis respectively.

To decipher the possible regulatory mechanisms of the ECM involved microenvironment during EC development, a molecular interaction network was generated by applying the 140 intersecting PAN-ECM genes using FunRich analysis. The generated network is shown in Fig 6. Each network consists of connected PAN-ECM genes with a hub gene. As indicated, hub genes with the most intensive connections were *FN1*, *ITGB3*, *ERBB2*, and *BMPR1B*. In addition, the hub genes with the contrasting fold change in tumorigenesis and metastasis included 100A7, *FBLN1*, *SERPINAS*, *TNFSF14*, *BMPR18*, and *GDF5*, which were upregulated in tumorigenesis and downregulated in metastasis, and *FSTL3*, *ADAMTS1*. *COL4A4*, *HGF*, *MAG*, *FN1*, *ITGB3*, *NCAM1*, *AGTR1*, and *DNM3*, which were downregulated and upregulated in tumorigenesis and metastasis, respectively.

### Gene coexpression network analysis

**Fig 7:**
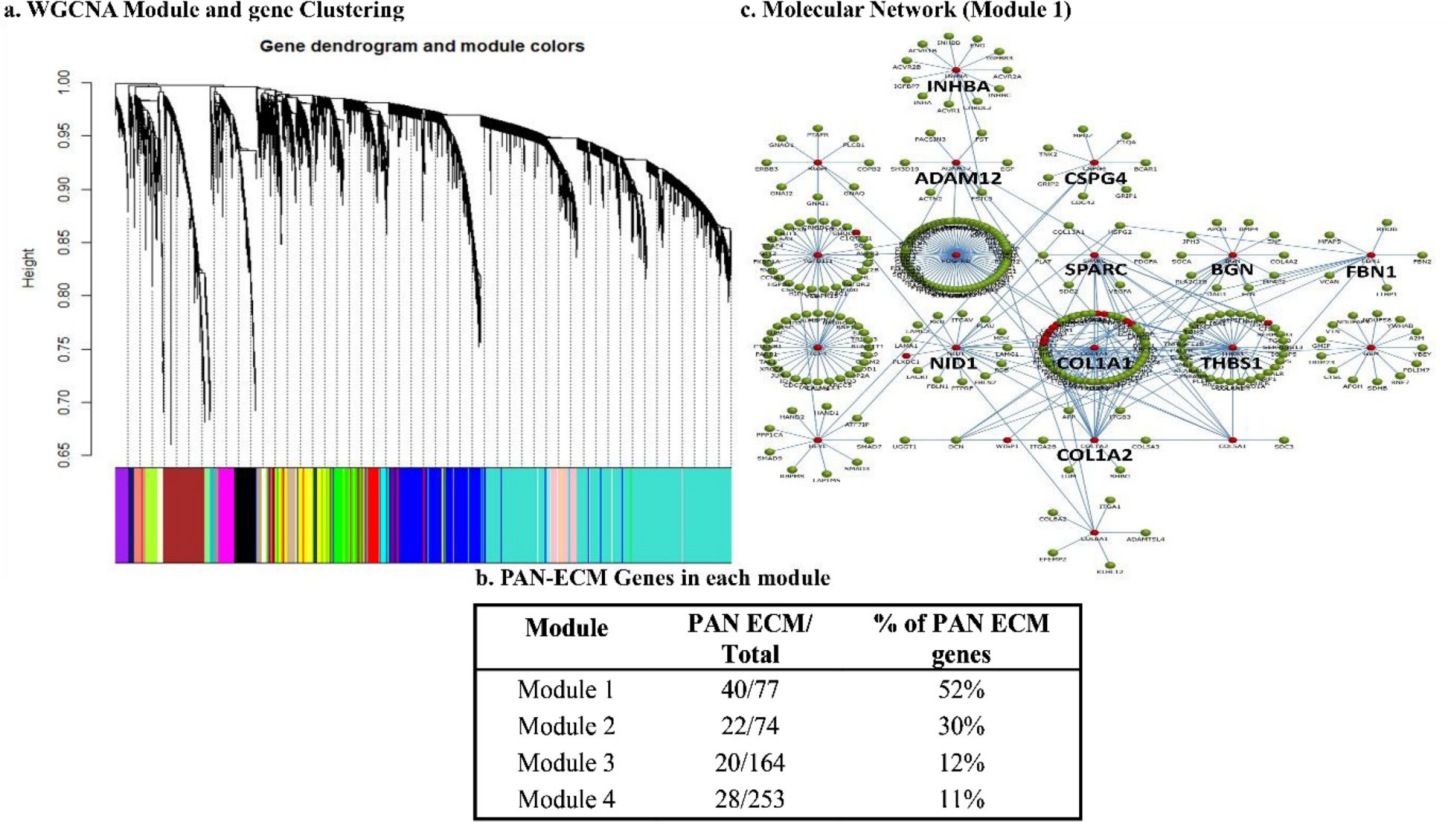
Gene coexpression network during endometrial cancer (EC) development. (a) A WGCNA study for gene coexpression network analysis between late and early stages of EC disease was carried out to determine the relationships between PAN-ECM genes and the other co-expressed differential exressed genes (DEGs) during EC development was varried out. (b) Percentage of PAN-ECM genes in each generated WGCNA module was indicated. (c) The identified hub genes and their molecular interaction of the molecular network of the genes in module 1 by FunRich analysis.

The gene coexpression network was constructed using the retrieved 20,531 genes from EC tumor samples using a systematic unsupervised method, WGCNA. As shown in Fig 7A, numerous modules were generated, whereas four modules consisting of the highest proportions of PAN-ECM genes, ranging 11%~52%, were selected for further analyses Fig 7B. Significantly involved pathways among the modules are enumerated in Table 3.

**Table 3:** Gene clustering of the metastasis-associated PAN-ECM differentially expressed genes (DEGs) by WGCNA module analysis.

Canonical pathway (IPA Knowledgebase database)	Metastasis PAN-ECM (241^+^) (z-score)	Module 1 (77) (z-score^+^)	Module 2 (74) (z-score^+^)	Module 3 (164) (z-score^+^)	Module 4 (253) (z-score^+^)
GP6 signaling pathway	4.15	2.11	1.41	0.00	0.00
Dendritic cell maturation	2.45	2.24	2.45	0.00	0.90
Osteoarthritis pathway	2.31	1.34	0.45		
Colorectal cancer metastasis signaling	2.89		2.14		
Wnt/Î^2^-catenin signaling	2.45		-2.00		0.00
Neuroinflammation signaling pathway	2.45	0.00	1.73	0.00	0.30
ILK signaling	2.45		1.89		
Role of pattern recognition receptors in recognition of bacteria and viruses	2.24	0.00	2.45	0.00	0.38
Neuregulin signaling	2.24	0.00	2.24	0.00	
Signaling by Rho family GTPases	2.00	0.00	2.53		0.00
IL-6 signaling	2.00		3.00		
ErbB signaling	2.00	0.00	2.71	0.00	
Growth hormone signaling	2.00	0.00	2.65		
Glioblastoma multiform signaling	1.89		2.71		
Leukocyte extravasation signaling	1.63		2.53		-1.67
HMGB1 signaling	1.41	0.00	2.83		
IL-8 signaling	1.34	0.00	3.46	-1.00	-0.71
Role of NFAT in cardiac hypertrophy	1.34		2.71		
eNOS signaling	1.00	0.00	2.65		
FGF signaling	0.82	0.00	2.65	0.00	
Acute-phase response signaling	0.45		2.45		
GÎ±12/13 signaling	-0.45	0.00	3.00		
RhoGDI signaling	-1.00		-1.34		
LXR/RXR activation	-1.00	0.00	0.00	0.00	0.45

The canonical pathways involved in each modules was listed and sorted by z-score.

^**+**^Number of genes.

As described previously, high grade and re-occurred EC have poor prognosis outcome. Therefore, we attempted to dig the possible mechanisms and the characteristics of the ECM microenvironment during high-grade EC at metastasis stage. The pathway analyses for metastasis-associated PAN-ECM genes listed in Table 3 showed that module 1 and 2 possessed the highest proportions (52% and 30% respectively) of PAN-ECM genes, where those PAN-ECM genes are involved in three pathways, GP6 signaling, dendritic cell maturation, and osteoarthritis pathways, suggesting that those PAN-ECM genes and their involved pathways may play crucial roles during EC metastasis.

In addition, colorectal cancer metastasis signalling, IL-6 signaling, and IL-8 signaling also were also predicted to be activated during EC metastasis, whereas RhoGDI signaling pathway was predicted to be inhibited in metastatic EC. The molecular network of coexpression module 1 containing the highest proportion of PAN-ECM genes including *COL1A1*, *BGN*, *NID1*, *ADAM12*, *COL1A2*, *INHBA*, *THBS1*, *SPARC*, *FBN1*, and *CSPG4* was shown in Fig 7C. The typical metastasis-associated hub genes were identified.

## Discussion

In this study, PAN-ECM Data List was generated, that covers not only ECMs, but also synthetic and degradation enzyme for ECMs and ECM associated molecules and the regulators, also present in the extracellular microenvironment surrounding cells. The PAN-ECM Data List has a total of 1516 ECMs and ECM-associated genes. Comparison to the Matrisome Project [28], there were 489 additional genes that contribute in 9 categories including cell surface receptor, cytoskeleton, cell adhesion molecules, ECM receptor cofactors, ECM receptors, ECM membrane-associated, ECM-associated regulatory factors, ECM synthetic/degradation enzymes, and ECM microenvironment associated. Some of the genes were reported to be involved in many critical biological processes, for instance, *integrin subunit alpha 2b (ITGA)* and *intercellular adhesion molecule 1 (ICAM1)* genes, which is an ECM receptor and a molecule that can bind to integrin, respectively, both play crucial roles in tumor development [42]. In addition, the cell surface receptor category includes transforming growth factor-beta receptor genes (*TGFBR1* and *TGFBR3*), and that the elevated *TGFBR* levels could induce collagen expression in metastatic breast cancer cells [43]. Several genes in the cell adhesion molecule category, such as *CD44* and *CADM1*, were involved in cancer cell migration and the regulation of ECM adhesion [44, 45]. *ITGB3* and *BMPR2* genes were key regulators of cell migration and invasion of cancer cells [46, 47]. *XYLT1* in ECM synthetic/degradation enzymes play important roles in the initiation step of the biosynthesis of glycosaminoglycan [48]. Differential expression of *XDH* gene modulated the migration of human breast cancer cells [49].

One forty ECM and ECM-associated genes have been identified, that may participate in tumorigenesis and metastasis of EC. Numerous identified genes have been reported to participate in the development of other cancers, for instance, *COL11A1*, *MMP1*, *COL10A1*, *HHIP*, and *COL6A6* could be prognostic and predictive indicators of early-stage non-small cell lung cancer (NSCLC) [50]; upregulation of *ITBG3* and *COL4A3* genes could respectively promote colorectal cancer and breast cancer development [51, 52]. Alterations of *EGFL6* associated with metastasis of ovarian cancer [53].

Interestingly, several PAN-ECM genes involved signaling pathways, GP6 signaling pathway, ILK signaling pathway, and IL-8 signaling pathway were significantly inhibited in tumorigenesis and activated in metastasis of EC, suggesting that those specific PAN-ECM genes may participate in EC metastasis and progression. To our knowledge, PAN-ECM gene involved GP6 signaling in association with EC progression has not been reported. GP6, a glycoprotein, is mainly expressed in platelets. GP6 signaling pathway was primarily thought to participate in platelets and their precursor megakaryocyte activation [54]. Aggregation of platelets may shield circulating tumor cells from immunosurveillance and to promote tumor progression and metastasis [55]. Another report suggested that GP6 served as the primary signaling receptor for collagen, which induces platelet activation and thrombus formation [54]. Defect of GP6 could cause malfunction of platelet reactivity to collagens in blood and lung cancer metastasis [56, 57]. An elevated level of serum platelets lowered the chemotherapy response rate and increased the risk of recurrence in EC [58]. Our current study identified several collagen genes that were upregulated in metastatic EC, suggesting that the upregulated collagens may activate GP6 signaling and protect the EC cells from immunosurveillance and promote tumor metastasis.

ILK signaling pathway was shown to represent an essential kinase activity and also regulated the decidualization of endometrial stromal cells [59]. It also played a role in hormone-dependent cancer progression and regulated a variety of cellular functions including cell survival, migration, and angiogenesis, its gain or loss of function resulted in oncogenic transformation and progression of invasive and metastatic phenotypes [60]. IL-8 signaling belongs to CXC chemokine family. Many studies confirmed high levels of IL-8 in HER2-enriched and basal-like (ER–) primary breast cancer, whereas patients with a low level of IL8 had better prognosis [61].

The hub gene they interact with the other PAN-ECM genes may play crucial roles in regulating the EC development and the progression. From the molecular network analysis **(**Fig 6**)**, we identified several hub genes, such as *FN1*, *ITGB3*, and *BMPR1B*, interacted with the other ECM molecules intensively. These genes were associated with many critical biological functions related to cancer cell metastasis. *FN1* was previously reported to be associated with adverse or poor prognosis of breast and ovarian cancer [62]. Increased expression of *ITGB3* could promote and control the metastasis of breast cancer and EC [63, 64]. *BMPR1B* was correlated with cell proliferation, suggesting that it may play a role in protecting against endometriosis progression [65]. A previous study by Frida et al.'s team explained the transformation of normal cells to malignant and metastatic tumor cells. *VCAN*, an ECM gene was initially downregulated but upregulated in metastasizing cells in the prostate and colorectal cancers [66]. Our results showed that the metastatic EC associated ECM genes were highly expressed at a late stage and might participate in EC progression.

From the coexpression network analysis identified several modules possessing higher proportions of ECM genes. Interestingly, the GP6 signaling pathway along with the dendritic cell maturation and osteoarthritis pathways were activated in modules 1 and 2 and in metastatic DEGs (Table 3). Notably, GP6 signaling was identified not only by functional enrichment analysis but by coexpression network analysis. PAN-ECM genes, such as *COL1A1*, *COL1A2*, *BGN*, *NID1*, *ADAM12*, *INHBA*, *THBS1*, *SPARC*, and *CSPG4*, were identified to act as hub genes. Increased expressions of *COL1A1* and *COL1A2* genes were associated with lower survival of ovarian cancer patients [67]. *BGN* gene could enhance migration and invasion of EC [68]. *NID1* gene was found to be a new therapeutic target biomarker in breast cancer and EC invasion [69]. *ADAM12* was a tumor marker and has negative prognostic value for overall survival in ovarian cancer [70]. Strong colocalization of *INHBA* was highly associated with malignant endometrial tissues [71]. *THBS1* expression might associate with the survival of EC patients [72]. Overexpression of *SPARC* gene could promote migration of EC [73]. Increased expression of *CSPG4* gene was correlated with disease reappearance in patients with breast cancer [74].

Increased matrix stiffness has a profound effect on tumor development and progression [75]. Upregulation of collagen and integrins is known to participate in stiffness [76, 77]. Increased matrix stiffness resulted in increases in cell outgrowth and sprouting, which were correlated with cancer metastasis [78]. Collagen expressions, mainly types I, III, and VI, have been studied in the past few years, and higher expressions of those ECM genes were positively correlated with matrix stiffness [27, 79–81]. In this study, many collagen-associated genes, such as *COL4A4*, *COL19A1*, and *COL6A6* **(**Table 1), showed decreased expressions at early stages and increased expressions at the late stage of EC. These findings suggested that those ECM genes may participate in EC progression.

## Conclusions

In this work, we compiled a comprehensive PAN-ECM Data List including ECM and ECM-associated genes. The ECM genes including collagens and integrins and the signaling pathway, GP6, ILK signaling, and IL-8 signaling pathways, were highly correlated with metastatic EC. The identified PAN-ECM genes and their participated signaling pathways may lead to the development of novel biomarkers and new therapeutic strategies for the re-occurred, poor prognosis of high-grade EC.

## Supporting information

S1 Fig Ingenuity Pathway Analysis (IPA) of tumorigenesis- and metastasis-associated PAN-ECM gene involved canonical pathways. (a) List of tumorigenesis-associated PAN-ECM gene involved canonical pathways (b) List of metastasis-associated PAN-ECM gene involved canonical pathways. Orange color indicates activated pathways with positive z-scores. Blue color indicates inhibited pathways with negative z-scores. The color is darker indicate the absolute z-score is higher.
(DOCX)

S1 Table Complete PAN-ECM Data List with PUBMED ID (1516). (DOCX)

S2 Table Tumorigenesis PAN-ECM DEGs (831 genes). (DOCX)

S3 Table Metastasis PAN-ECM DEGs (241). (DOCX)

## References

[1] Hynes RO. The extracellular matrix: not just pretty fibrils. Science (New York, NY). 2009;326(5957):1216–9. 10.1126/science.1176009 PMC3536535 19965464

[2] Mott JD, Werb Z. Regulation of matrix biology by matrix metalloproteinases. Current Opinion in Cell Biology. 2004;16(5):558–64. 10.1016/j.ceb.2004.07.010 15363807 PMC2775446

[3] Lu P, Weaver VM, Werb Z. The extracellular matrix: a dynamic niche in cancer progression. J Cell Biol. 2012;196(4):395–406. 10.1083/jcb.201102147 22351925 PMC3283993

[4] Konrad D, Marcin O, Beniamin G, Nikola Z, Robert K, Ewa L, et al Changes in Expression Pattern of SEMA3F Depending on Endometrial Cancer Grade—Pilot Study. Current Pharmaceutical Biotechnology. 2019;20(9):727–32. 10.2174/1389201020666190619145655 31215376 PMC7046987

[5] Hynes RO. Integrins: Bidirectional, Allosteric Signaling Machines. Cell. 2002;110(6):673–87. 10.1016/s0092-8674(02)00971-6 12297042

[6] Liu L, Zhang SX, Liao W, Farhoodi HP, Wong CW, Chen CC, et al Mechanoresponsive stem cells to target cancer metastases through biophysical cues. Science Translational Medicine. 2017;9(400). 10.1126/scitranslmed.aan2966 PMC5890431 28747514

[7] Wei SC, Fattet L, Tsai JH, Guo Y, Pai VH, Majeski HE, et al Matrix stiffness drives epithelial-mesenchymal transition and tumour metastasis through a TWIST1-G3BP2 mechanotransduction pathway. Nature cell biology. 2015;17(5):678–88. 10.1038/ncb3157 25893917 PMC4452027

[8] Ali MY, Chuang CY, Saif MT. Reprogramming cellular phenotype by soft collagen gels. Soft matter. 2014;10(44):8829–37. 10.1039/c4sm01602e 25284029 PMC4208984

[9] Cox TR, Erler JT. Remodeling and homeostasis of the extracellular matrix: implications for fibrotic diseases and cancer. Disease Models & Mechanisms. 2011;4(2):165–78. 21324931 10.1242/dmm.004077 PMC3046088

[10] Seewaldt V. ECM stiffness paves the way for tumor cells. Nature Medicine. 2014;20:332 10.1038/nm.3523 24710372

[11] Nicolaije KAH, Ezendam NPM, Vos MC, Boll D, Pijnenborg JMA, Kruitwagen RFPM, et al Follow-up practice in endometrial cancer and the association with patient and hospital characteristics: A study from the population-based PROFILES registry. Gynecologic Oncology. 2013;129(2):324–31. 10.1016/j.ygyno.2013.02.018 23435365

[12] Staff N. Endometrial Cancer Incidence Rising in the US and Worldwide. 2017.

[13] Sutton G, Axelrod JH, Bundy BN, Roy T, Homesley HD, Malfetano JH, et al Whole abdominal radiotherapy in the adjuvant treatment of patients with stage III and IV endometrial cancer: a gynecologic oncology group study. Gynecol Oncol. 2005;97(3):755–63. 10.1016/j.ygyno.2005.03.011 15913742

[14] Boll D, Karim-Kos HE, Verhoeven R.H.A, Burger CW, Coebergh JW, van de Poll-Franse LV, et al Increased incidence and improved survival in endometrioid endometrial cancer diagnosed since 1989 in The Netherlands: a population based study. European Journal of Obstetrics & Gynecology and Reproductive Biology. 2013;166(2):209–14. 23176760 10.1016/j.ejogrb.2012.10.028

[15] Amant F, Moerman P, Neven P, Timmerman D, Van Limbergen E, Vergote I. Endometrial cancer. Lancet (London, England). 2005;366(9484):491–505. 10.1016/S0140-6736(05)67063-8 16084259

[16] Lax SF, Kurman RJ. A dualistic model for endometrial carcinogenesis based on immunohistochemical and molecular genetic analyses. Verhandlungen der Deutschen Gesellschaft fur Pathologie. 1997;81:228–32. 9474874

[17] Owens GL, Lawrence KM, Jackson TR, Crosbie EJ, Sayan BS, Kitchener HC, et al Urocortin suppresses endometrial cancer cell migration via CRFR2 and its system components are differentially modulated by estrogen. Cancer Medicine. 2017;6(2):408–15. 10.1002/cam4.967 28109061 PMC5313640

[18] Onstad MA, Schmandt RE, Lu KH. Addressing the Role of Obesity in Endometrial Cancer Risk, Prevention, and Treatment. Journal of Clinical Oncology. 2016;34(35):4225–30. 10.1200/JCO.2016.69.4638 27903150 PMC5455320

[19] Matias-Guiu X, Catasus L, Bussaglia E, Lagarda H, Garcia A, Pons C, et al Molecular pathology of endometrial hyperplasia and carcinoma. Human pathology. 2001;32(6):569–77. 10.1053/hupa.2001.25929 11431710

[20] Win AK, Reece JC, Ryan S. Family history and risk of endometrial cancer: a systematic review and meta-analysis. Obstetrics and gynecology. 2015;125(1):89–98. 10.1097/AOG.0000000000000563 25560109

[21] Freeman SJ, Aly AM, Kataoka MY, Addley HC, Reinhold C, Sala E. The revised FIGO staging system for uterine malignancies: implications for MR imaging. Radiographics: a review publication of the Radiological Society of North America, Inc. 2012;32(6):1805–27. 10.1148/rg.326125519 23065170

[22] Amin MB, Edge SB, American Joint Committee on C. AJCC cancer staging manual 2017.

[23] DNA-damaging therapies emerging as possible triple-negative breast cancer therapies. Oncology (Williston Park). 2011;25(11):1088, 91. 22106563

[24] Abdel-Fatah TM, Arora A, Moseley PM, Perry C, Rakha EA, Green AR, et al DNA repair prognostic index modelling reveals an essential role for base excision repair in influencing clinical outcomes in ER negative and triple negative breast cancers. Oncotarget. 2015;6(26):21964–78. 10.18632/oncotarget.4157 26267318 PMC4673139

[25] Aaboe M, Offersen BV, Christensen A, Andreasen PA. Vitronectin in human breast carcinomas. Biochim Biophys Acta. 2003;1638(1):72–82. 10.1016/s0925-4439(03)00059-0 12757937

[26] Grabarek B, Wcislo-Dziadecka D, Gola J, Kruszniewska-Rajs C, Brzezinska-Wcislo L, Zmarzly N, et al Changes in the Expression Profile of JAK/STAT Signaling Pathway Genes and Mirnas Regulating their Expression Under the Adalimumab Therapy. Curr Pharm Biotechnol. 2018;19(7):556–65. 10.2174/1389201019666180730094046 30058482

[27] Barcus CE, Keely PJ, Eliceiri KW, Schuler LA. Stiff collagen matrices increase tumorigenic prolactin signaling in breast cancer cells. J Biol Chem. 2013;288(18):12722–32. 10.1074/jbc.M112.447631 23530035 PMC3642318

[28] Naba A, Clauser KR, Ding H, Whittaker CA, Carr SA, Hynes RO. The extracellular matrix: Tools and insights for the “omics” era. Matrix Biology. 2016;49:10–24. 10.1016/j.matbio.2015.06.003 26163349 PMC5013529

[29] Byron A, Humphries JD, Humphries MJ. Defining the extracellular matrix using proteomics. International journal of experimental pathology. 2013;94(2):75–92. 10.1111/iep.12011 23419153 PMC3607136

[30] Moriarity JL, Hurt KJ, Resnick AC, Storm PB, Laroy W, Schnaar RL, et al UDP-glucuronate decarboxylase, a key enzyme in proteoglycan synthesis: cloning, characterization, and localization. The Journal of biological chemistry. 2002;277(19):16968–75. 10.1074/jbc.M109316200 11877387

[31] Gotting C, Kuhn J, Zahn R, Brinkmann T, Kleesiek K. Molecular cloning and expression of human UDP-d-Xylose:proteoglycan core protein beta-d-xylosyltransferase and its first isoform XT-II. Journal of molecular biology. 2000;304(4):517–28. 10.1006/jmbi.2000.4261 11099377

[32] Adhikary A, Chakraborty S, Mazumdar M, Ghosh S, Mukherjee S, Manna A, et al Inhibition of epithelial to mesenchymal transition by E-cadherin up-regulation via repression of slug transcription and inhibition of E-cadherin degradation: dual role of scaffold/matrix attachment region-binding protein 1 (SMAR1) in breast cancer cells. The Journal of biological chemistry. 2014;289(37):25431–44. 10.1074/jbc.M113.527267 25086032 PMC4162148

[33] Herishanu Y, Gibellini F, Njuguna N, Hazan-Halevy I, Farooqui M, Bern S, et al Activation of CD44, a receptor for extracellular matrix components, protects chronic lymphocytic leukemia cells from spontaneous and drug induced apoptosis through MCL-1. Leukemia & lymphoma. 2011;52(9):1758–69. 21649540 10.3109/10428194.2011.569962 PMC3403533

[34] Matsubara T, Araki M, Abe H, Ueda O, Jishage K, Mima A, et al Bone Morphogenetic Protein 4 and Smad1 Mediate Extracellular Matrix Production in the Development of Diabetic Nephropathy. Diabetes. 2015;64(8):2978–90. 10.2337/db14-0893 25995358

[35] Su Y, Yang CY, Li Z, Xu F, Zhang L, Wang F, et al Smad7 siRNA inhibit expression of extracellular matrix in trabecular meshwork cells treated with TGF-beta2. Molecular vision. 2012;18:1881–4. 22876112 PMC3413437

[36] Weinstein JN, Collisson EA, Mills GB, Shaw KRM, Ozenberger BA, Ellrott K, et al The cancer genome atlas pan-cancer analysis project. Nature genetics. 2013;45(10):1113 10.1038/ng.2764 24071849 PMC3919969

[37] Love MI, Huber W, Anders S. Moderated estimation of fold change and dispersion for RNA-seq data with DESeq2. Genome Biology. 2014;15(12):550 10.1186/s13059-014-0550-8 25516281 PMC4302049

[38] Gordon A, Glazko G, Qiu X, Yakovlev A. Control of the mean number of false discoveries, Bonferroni and stability of multiple testing. The Annals of Applied Statistics. 2007:179–90.

[39] Kramer A, Green J, Pollard J Jr., Tugendreich S. Causal analysis approaches in Ingenuity Pathway Analysis. Bioinformatics. 2014;30(4):523–30. 10.1093/bioinformatics/btt703 24336805 PMC3928520

[40] Pathan M, Keerthikumar S, Ang CS, Gangoda L, Quek CYJ, Williamson NA, et al FunRich: An open access standalone functional enrichment and interaction network analysis tool. PROTEOMICS. 2015;15(15):2597–601. 10.1002/pmic.201400515 25921073

[41] Langfelder P, Horvath S. WGCNA: an R package for weighted correlation network analysis. BMC Bioinformatics. 2008;9(1):559. 19114008 10.1186/1471-2105-9-559 PMC2631488

[42] Desgrosellier JS, Cheresh DA. Integrins in cancer: biological implications and therapeutic opportunities. Nature Reviews Cancer. 2010;10(1):9 10.1038/nrc2748 20029421 PMC4383089

[43] Selvamurugan N, Kwok S, Partridge NC. Smad3 interacts with JunB and Cbfa1/Runx2 for transforming growth factor-beta1-stimulated collagenase-3 expression in human breast cancer cells. The Journal of biological chemistry. 2004;279(26):27764–73. 10.1074/jbc.M312870200 15084595

[44] Kuo YC, Su CH, Liu CY, Chen TH, Chen CP, Wang HS. Transforming growth factor-beta induces CD44 cleavage that promotes migration of MDA-MB-435s cells through the up-regulation of membrane type 1-matrix metalloproteinase. International journal of cancer. 2009;124(11):2568–76. 10.1002/ijc.24263 19243022

[45] Moiseeva EP, Straatman KR, Leyland ML, Bradding P. CADM1 controls actin cytoskeleton assembly and regulates extracellular matrix adhesion in human mast cells. PloS one. 2014;9(1):e85980 10.1371/journal.pone.0085980 24465823 PMC3899107

[46] Owens P, Pickup MW, Novitskiy SV, Chytil A, Gorska AE, Aakre ME, et al Disruption of bone morphogenetic protein receptor 2 (BMPR2) in mammary tumors promotes metastases through cell autonomous and paracrine mediators. Proceedings of the National Academy of Sciences. 2012;109(8):2814–9. 10.1073/pnas.1101139108 PMC3286911 21576484

[47] Page JM, Merkel AR, Ruppender NS, Guo R, Dadwal UC, Cannonier S, et al Matrix rigidity regulates the transition of tumor cells to a bone-destructive phenotype through integrin beta3 and TGF-beta receptor type II. Biomaterials. 2015;64:33–44. 10.1016/j.biomaterials.2015.06.026 26115412 PMC4681301

[48] Cuellar K, Chuong H, Hubbell SM, Hinsdale ME. Biosynthesis of chondroitin and heparan sulfate in chinese hamster ovary cells depends on xylosyltransferase II. The Journal of biological chemistry. 2007;282(8):5195–200. 10.1074/jbc.M611048200 17189266

[49] Fini MA, Orchard-Webb D, Kosmider B, Amon JD, Kelland R, Shibao G, et al Migratory activity of human breast cancer cells is modulated by differential expression of xanthine oxidoreductase. Journal of cellular biochemistry. 2008;105(4):1008–26. 10.1002/jcb.21901 18767115 PMC2587521

[50] Lim SB, Tan SJ, Lim W-T, Lim CT. An extracellular matrix-related prognostic and predictive indicator for early-stage non-small cell lung cancer. Nature Communications. 2017;8(1):1734 10.1038/s41467-017-01430-6 29170406 PMC5700969

[51] Lei Y, Huang K, Gao C, Lau QC, Pan H, Xie K, et al Proteomics Identification of ITGB3 as a Key Regulator in Reactive Oxygen Species-induced Migration and Invasion of Colorectal Cancer Cells. Molecular & Cellular Proteomics: MCP. 2011;10(10):M110.005397. 10.1074/mcp.M110.005397 PMC3205852 21622897

[52] Georgiou GK, Igglezou M, Sainis I, Vareli K, Batsis H, Briasoulis E, et al Impact of breast cancer surgery on angiogenesis circulating biomarkers: a prospective longitudinal study. World Journal of Surgical Oncology. 2013;11(1):213. 23981902 10.1186/1477-7819-11-213 PMC3846614

[53] Du XL, Jiang T, Zhao WB, Wang F, Wang GL, Cui M, et al Gene alterations in tumor-associated endothelial cells from endometrial cancer. Int J Mol Med. 2008;22(5):619–32. 18949382

[54] Ezumi Y, Uchiyama T, Takayama H. Molecular Cloning, Genomic Structure, Chromosomal Localization, and Alternative Splice Forms of the Platelet Collagen Receptor Glycoprotein VI. Biochemical and Biophysical Research Communications. 2000;277(1):27–36. 10.1006/bbrc.2000.3624 11027634

[55] Kopp HG, Placke T, Salih HR. Platelet-derived transforming growth factor-beta down-regulates NKG2D thereby inhibiting natural killer cell antitumor reactivity. Cancer research. 2009;69(19):7775–83. 10.1158/0008-5472.CAN-09-2123 19738039

[56] Bellucci S, Huisse MG, Boval B, Hainaud P, Robert A, Fauvel-Lafeve F, et al Defective collagen-induced platelet activation in two patients with malignant haemopathies is related to a defect in the GPVI-coupled signalling pathway. Thrombosis and haemostasis. 2005;93(1):130–8. 10.1160/TH04-05-0312 15630503

[57] Jain S, Russell S, Ware J. Platelet glycoprotein VI facilitates experimental lung metastasis in syngenic mouse models. Journal of thrombosis and haemostasis: JTH. 2009;7(10):1713–7. 10.1111/j.1538-7836.2009.03559.x 19624454

[58] Abdel-Fatah TM, Russell R, Albarakati N, Maloney DJ, Dorjsuren D, Rueda OM, et al Genomic and protein expression analysis reveals flap endonuclease 1 (FEN1) as a key biomarker in breast and ovarian cancer. Mol Oncol. 2014;8(7):1326–38. 10.1016/j.molonc.2014.04.009 24880630 PMC4690463

[59] Yen C-F, Wang H-S, Lee C-L, Liao S-K. Roles of integrin-linked kinase in cell signaling and its perspectives as a therapeutic target. Gynecology and Minimally Invasive Therapy. 2014;3(3):67–72.

[60] Tan C, Cruet-Hennequart S, Troussard A, Fazli L, Costello P, Sutton K, et al Regulation of tumor angiogenesis by integrin-linked kinase (ILK). Cancer Cell. 2004;5(1):79–90. 10.1016/s1535-6108(03)00281-2 14749128

[61] Milovanović J, Todorović-Raković N, Radulovic M. Interleukin-6 and interleukin-8 serum levels in prognosis of hormone-dependent breast cancer. Cytokine. 2018. 10.1016/j.cyto.2018.02.019 29482885

[62] Kenny HA, Kaur S, Coussens LM, Lengyel E. The initial steps of ovarian cancer cell metastasis are mediated by MMP-2 cleavage of vitronectin and fibronectin. The Journal of clinical investigation. 2008;118(4):1367–79. 10.1172/JCI33775 18340378 PMC2267016

[63] Lin TH, Liu HH, Tsai TH, Chen CC, Hsieh TF, Lee SS, et al CCL2 increases alphavbeta3 integrin expression and subsequently promotes prostate cancer migration. Biochim Biophys Acta. 2013;1830(10):4917–27. 10.1016/j.bbagen.2013.06.033 23845726

[64] Xiong S, Klausen C, Cheng JC, Zhu H, Leung PC. Activin B induces human endometrial cancer cell adhesion, migration and invasion by up-regulating integrin beta3 via SMAD2/3 signaling. Oncotarget. 2015;6(31):31659–73. 10.18632/oncotarget.5229 26384307 PMC4741631

[65] Categorization of triple-negative breast cancer patients will help in targeted therapy selection. Oncology (Williston Park). 2011;25(9):849. 21936450

[66] Abecassis J, Millon-Collard R, Klein-Soyer C, Nicora F, Fricker JP, Beretz A, et al Adhesion of human breast cancer cell line MCF-7 to human vascular endothelial cells in culture. Enhancement by activated platelets. Int J Cancer. 1987;40(4):525–31. 10.1002/ijc.2910400416 3666990

[67] Shen Y, Shen R, Ge L, Zhu Q, Li F. Fibrillar type I collagen matrices enhance metastasis/invasion of ovarian epithelial cancer via beta1 integrin and PTEN signals. International journal of gynecological cancer: official journal of the International Gynecological Cancer Society. 2012;22(8):1316–24. 23013730 10.1097/IGC.0b013e318263ef34

[68] Sun H, Wang X, Zhang Y, Che X, Liu Z, Zhang L, et al Biglycan enhances the ability of migration and invasion in endometrial cancer. Archives of gynecology and obstetrics. 2016;293(2):429–38. 10.1007/s00404-015-3844-5 26275380

[69] Pedrola N, Devis L, Llaurado M, Campoy I, Martinez-Garcia E, Garcia M, et al Nidogen 1 and Nuclear Protein 1: novel targets of ETV5 transcription factor involved in endometrial cancer invasion. Clinical & experimental metastasis. 2015;32(5):467–78. 25924802 10.1007/s10585-015-9720-7

[70] Vlad C, Kubelac P, Onisim A, Fetica B, Fulop A, Irimie A, et al Expression of CDCP1 and ADAM12 in the ovarian cancer microenvironment. Journal of BUON: official journal of the Balkan Union of Oncology. 2016;21(4):973–8. 27685922

[71] Torres PB, Florio P, Galleri L, Reis FM, Borges LE, Petraglia F. Activin A, activin receptor type II, nodal, and cripto mRNA are expressed by eutopic and ectopic endometrium in women with ovarian endometriosis. Reproductive sciences (Thousand Oaks, Calif). 2009;16(8):727–33. 10.1177/1933719109334967 19386982

[72] Seki N, Kodama J, Hashimoto I, Hongo A, Yoshinouchi M, Kudo T. Thrombospondin-1 and -2 messenger RNA expression in normal and neoplastic endometrial tissues: correlation with angiogenesis and prognosis. International journal of oncology. 2001;19(2):305–10. 10.3892/ijo.19.2.305 11445843

[73] Yusuf N, Inagaki T, Kusunoki S, Okabe H, Yamada I, Matsumoto A, et al SPARC was overexpressed in human endometrial cancer stem-like cells and promoted migration activity. Gynecol Oncol. 2014;134(2):356–63. 10.1016/j.ygyno.2014.04.009 24769035

[74] Hsu NC, Nien PY, Yokoyama KK, Chu PY, Hou MF. High chondroitin sulfate proteoglycan 4 expression correlates with poor outcome in patients with breast cancer. Biochem Biophys Res Commun. 2013;441(2):514–8. 10.1016/j.bbrc.2013.10.093 24177010

[75] Reid SE, Kay EJ, Neilson LJ, Henze A-T, Serneels J, McGhee EJ, et al Tumor matrix stiffness promotes metastatic cancer cell interaction with the endothelium. The EMBO journal. 2017;36(16):2373–89. 10.15252/embj.201694912 28694244 PMC5556271

[76] Lu P, Weaver VM, Werb Z. The extracellular matrix: A dynamic niche in cancer progression. The Journal of cell biology. 2012;196(4):395–406. 10.1083/jcb.201102147 22351925 PMC3283993

[77] Du J, Zu Y, Li J, Du S, Xu Y, Zhang L, et al Extracellular matrix stiffness dictates Wnt expression through integrin pathway. Scientific Reports. 2016;6:20395 10.1038/srep20395 26854061 PMC4745056

[78] Parikh D, Stack M, Wang H. Matrix Stiffness Regulates the Fate of Breast Cancer Cells. Biophysical Journal. 2018;114(3):651a.

[79] Alexopoulos LG, Youn I, Bonaldo P, Guilak F. Developmental and osteoarthritic changes in Col6a1-knockout mice: biomechanics of type VI collagen in the cartilage pericellular matrix. Arthritis and rheumatism. 2009;60(3):771–9. 10.1002/art.24293 19248115 PMC2724839

[80] Giménez A, Duch P, Puig M, Gabasa M, Xaubet A, Alcaraz J. Dysregulated Collagen Homeostasis by Matrix Stiffening and TGF-β1 in Fibroblasts from Idiopathic Pulmonary Fibrosis Patients: Role of FAK/Akt. International journal of molecular sciences. 2017;18(11):2431. 10.3390/ijms18112431 PMC5713399 29144435

[81] Karamichos D, Brown RA, Mudera V. Collagen stiffness regulates cellular contraction and matrix remodeling gene expression. Journal of biomedical materials research Part A. 2007;83(3):887–94. 10.1002/jbm.a.31423 17567861

