# Supplementary figures and images for: Computational analysis for identification of the extracellular matrix molecules involved in endometrial cancer progression
Source: PLoS One. 2020 Apr 21;15(4):e0231594. doi: 10.1371/journal.pone.0231594 (PMC7173926; doi:10.1371/journal.pone.0231594)

**
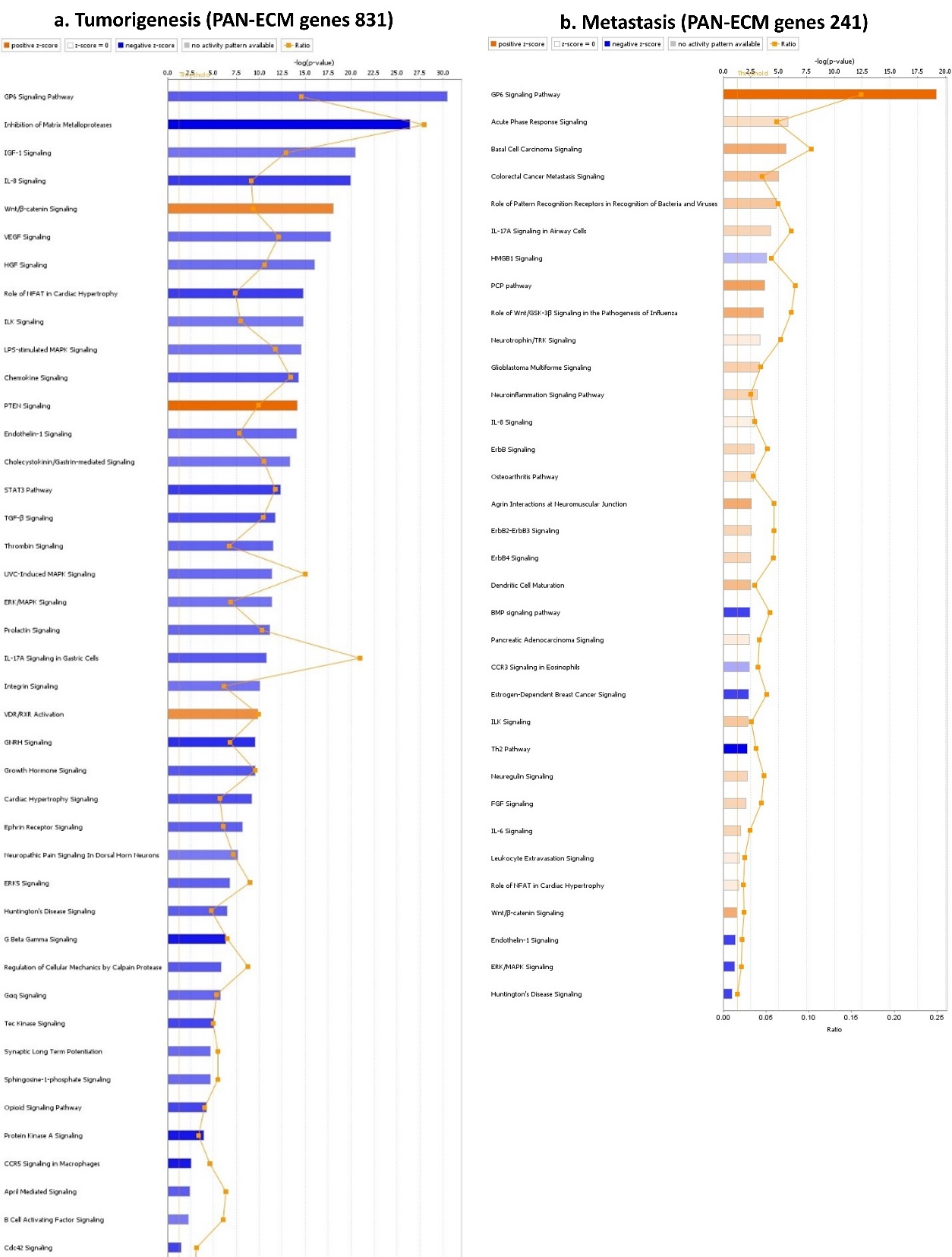
**

Supplement: S1 Fig — (a) List of tumorigenesis-associated PAN-ECM gene involved canonical pathways (b) List of metastasis-associated PAN-ECM gene involved canonical pathways. Orange color indicates activated pathways with positive z-scores. Blue color indicates inhibited pathways with negative z-scores. The color is darker indicate the absolute z-score is higher. (DOCX) [file pone.0231594.s001.docx]
