# Supplementary material for: Computational analysis for identification of the extracellular matrix molecules involved in endometrial cancer progression
Source: PLoS One. 2020 Apr 21;15(4):e0231594. doi: 10.1371/journal.pone.0231594 (PMC7173926; doi:10.1371/journal.pone.0231594)
Supplement: S1 Table — (DOCX) [file pone.0231594.s002.docx]

| **S1 Table. Complete PAN-ECM Data List with PUBMED ID (1516)** | | | |  |
| --- | --- | --- | --- | --- |
| **GeneSymbol** | **Category** | **PubMed IDs** | **Hyper Link for Paper** | **GeneCard annotation** |
| A1BG | ECM Micro-enviornment associated | 27559042 | [27559042](https://www.ncbi.nlm.nih.gov/pubmed/27559042) | [A1BG](https://www.genecards.org/Search/Keyword?queryString=A1BG) |
| A2M | ECM Regulators | 29464020 | [29464020](https://www.ncbi.nlm.nih.gov/pubmed/29464020) | [A2M](https://www.genecards.org/Search/Keyword?queryString=A2M) |
| A2ML1 | ECM Regulators | 23674615 | [23674615](https://www.ncbi.nlm.nih.gov/pubmed/23674615) | [A2ML1](https://www.genecards.org/Search/Keyword?queryString=A2ML1) |
| ABI3BP | ECM Glycoproteins | 23666637 | [23666637](https://www.ncbi.nlm.nih.gov/pubmed/23666637) | [ABI3BP](https://www.genecards.org/Search/Keyword?queryString=ABI3BP) |
| ACAN | Proteoglycans | 23419153 | [23419153](https://www.ncbi.nlm.nih.gov/pubmed/23419153) | [ACAN](https://www.genecards.org/Search/Keyword?queryString=ACAN) |
| ACTA1 | Cytoskeleton | 23580065 | [23580065](https://www.ncbi.nlm.nih.gov/pubmed/23580065) | [ACTA1](https://www.genecards.org/Search/Keyword?queryString=ACTA1) |
| ACTB | Cytoskeleton | 21423176 | [21423176](https://www.ncbi.nlm.nih.gov/pubmed/21423176) | [ACTB](https://www.genecards.org/Search/Keyword?queryString=ACTB) |
| ACTG1 | Cytoskeleton | 23979707 | [23979707](https://www.ncbi.nlm.nih.gov/pubmed/23979707) | [ACTG1](https://www.genecards.org/Search/Keyword?queryString=ACTG1) |
| ACTN1 | Cytoskeleton | 16807302 | [16807302](https://www.ncbi.nlm.nih.gov/pubmed/16807302) | [ACTN1](https://www.genecards.org/Search/Keyword?queryString=ACTN1) |
| ADA2 | ECM Micro-enviornment associated | 20147294 | [20147294](https://www.ncbi.nlm.nih.gov/pubmed/20147294) | [ADA2](https://www.genecards.org/Search/Keyword?queryString=ADA2) |
| ADAM10 | ECM Regulators | 24662289 | [24662289](https://www.ncbi.nlm.nih.gov/pubmed/24662289) | [ADAM10](https://www.genecards.org/Search/Keyword?queryString=ADAM10) |
| ADAM11 | ECM Regulators | 28216310 | [28216310](https://www.ncbi.nlm.nih.gov/pubmed/28216310) | [ADAM11](https://www.genecards.org/Search/Keyword?queryString=ADAM11) |
| ADAM12 | ECM Regulators | 28216310 | [28216310](https://www.ncbi.nlm.nih.gov/pubmed/28216310) | [ADAM12](https://www.genecards.org/Search/Keyword?queryString=ADAM12) |
| ADAM15 | ECM Regulators | 28216310 | [28216310](https://www.ncbi.nlm.nih.gov/pubmed/28216310) | [ADAM15](https://www.genecards.org/Search/Keyword?queryString=ADAM15) |
| ADAM17 | ECM Regulators | 28216310 | [28216310](https://www.ncbi.nlm.nih.gov/pubmed/28216310) | [ADAM17](https://www.genecards.org/Search/Keyword?queryString=ADAM17) |
| ADAM18 | ECM Regulators | 28216310 | [28216310](https://www.ncbi.nlm.nih.gov/pubmed/28216310) | [ADAM18](https://www.genecards.org/Search/Keyword?queryString=ADAM18) |
| ADAM19 | ECM Regulators | 28216310 | [28216310](https://www.ncbi.nlm.nih.gov/pubmed/28216310) | [ADAM19](https://www.genecards.org/Search/Keyword?queryString=ADAM19) |
| ADAM2 | ECM Regulators | 28216310 | [28216310](https://www.ncbi.nlm.nih.gov/pubmed/28216310) | [ADAM2](https://www.genecards.org/Search/Keyword?queryString=ADAM2) |
| ADAM20 | ECM Regulators | 28216310 | [28216310](https://www.ncbi.nlm.nih.gov/pubmed/28216310) | [ADAM20](https://www.genecards.org/Search/Keyword?queryString=ADAM20) |
| ADAM21 | ECM Regulators | 28216310 | [28216310](https://www.ncbi.nlm.nih.gov/pubmed/28216310) | [ADAM21](https://www.genecards.org/Search/Keyword?queryString=ADAM21) |
| ADAM22 | ECM Regulators | 28216310 | [28216310](https://www.ncbi.nlm.nih.gov/pubmed/28216310) | [ADAM22](https://www.genecards.org/Search/Keyword?queryString=ADAM22) |
| ADAM23 | ECM Regulators | 28216310 | [28216310](https://www.ncbi.nlm.nih.gov/pubmed/28216310) | [ADAM23](https://www.genecards.org/Search/Keyword?queryString=ADAM23) |
| ADAM28 | ECM Regulators | 28216310 | [28216310](https://www.ncbi.nlm.nih.gov/pubmed/28216310) | [ADAM28](https://www.genecards.org/Search/Keyword?queryString=ADAM28) |
| ADAM29 | ECM Regulators | 28216310 | [28216310](https://www.ncbi.nlm.nih.gov/pubmed/28216310) | [ADAM29](https://www.genecards.org/Search/Keyword?queryString=ADAM29) |
| ADAM30 | ECM Regulators | 28216310 | [28216310](https://www.ncbi.nlm.nih.gov/pubmed/28216310) | [ADAM30](https://www.genecards.org/Search/Keyword?queryString=ADAM30) |
| ADAM32 | ECM Regulators | 28216310 | [28216310](https://www.ncbi.nlm.nih.gov/pubmed/28216310) | [ADAM32](https://www.genecards.org/Search/Keyword?queryString=ADAM32) |
| ADAM33 | ECM Regulators | 28216310 | [28216310](https://www.ncbi.nlm.nih.gov/pubmed/28216310) | [ADAM33](https://www.genecards.org/Search/Keyword?queryString=ADAM33) |
| ADAM7 | ECM Regulators | 28216310 | [28216310](https://www.ncbi.nlm.nih.gov/pubmed/28216310) | [ADAM7](https://www.genecards.org/Search/Keyword?queryString=ADAM7) |
| ADAM8 | ECM Regulators | 28216310 | [28216310](https://www.ncbi.nlm.nih.gov/pubmed/28216310) | [ADAM8](https://www.genecards.org/Search/Keyword?queryString=ADAM8) |
| ADAM9 | ECM Regulators | 28216310 | [28216310](https://www.ncbi.nlm.nih.gov/pubmed/28216310) | [ADAM9](https://www.genecards.org/Search/Keyword?queryString=ADAM9) |
| ADAMDEC1 | ECM Regulators | 10373500 | [10373500](https://www.ncbi.nlm.nih.gov/pubmed/10373500) | [ADAMDEC1](https://www.genecards.org/Search/Keyword?queryString=ADAMDEC1) |
| ADAMTS1 | ECM Regulators | 28216310 | [28216310](https://www.ncbi.nlm.nih.gov/pubmed/28216310) | [ADAMTS1](https://www.genecards.org/Search/Keyword?queryString=ADAMTS1) |
| ADAMTS10 | ECM Regulators | 28216310 | [28216310](https://www.ncbi.nlm.nih.gov/pubmed/28216310) | [ADAMTS10](https://www.genecards.org/Search/Keyword?queryString=ADAMTS10) |
| ADAMTS12 | ECM Regulators | 28216310 | [28216310](https://www.ncbi.nlm.nih.gov/pubmed/28216310) | [ADAMTS12](https://www.genecards.org/Search/Keyword?queryString=ADAMTS12) |
| ADAMTS13 | ECM Regulators | 28495930 | [28495930](https://www.ncbi.nlm.nih.gov/pubmed/28495930) | [ADAMTS13](https://www.genecards.org/Search/Keyword?queryString=ADAMTS13) |
| ADAMTS14 | ECM Regulators | 28216310 | [28216310](https://www.ncbi.nlm.nih.gov/pubmed/28216310) | [ADAMTS14](https://www.genecards.org/Search/Keyword?queryString=ADAMTS14) |
| ADAMTS15 | ECM Regulators | 25770910 | [25770910](https://www.ncbi.nlm.nih.gov/pubmed/25770910) | [ADAMTS15](https://www.genecards.org/Search/Keyword?queryString=ADAMTS15) |
| ADAMTS16 | ECM Regulators | 25770910 | [25770910](https://www.ncbi.nlm.nih.gov/pubmed/25770910) | [ADAMTS16](https://www.genecards.org/Search/Keyword?queryString=ADAMTS16) |
| ADAMTS17 | ECM Regulators | 28216310 | [28216310](https://www.ncbi.nlm.nih.gov/pubmed/28216310) | [ADAMTS17](https://www.genecards.org/Search/Keyword?queryString=ADAMTS17) |
| ADAMTS18 | ECM Regulators | 25770910 | [25770910](https://www.ncbi.nlm.nih.gov/pubmed/25770910) | [ADAMTS18](https://www.genecards.org/Search/Keyword?queryString=ADAMTS18) |
| ADAMTS19 | ECM Regulators | 26634009 | [26634009](https://www.ncbi.nlm.nih.gov/pubmed/26634009) | [ADAMTS19](https://www.genecards.org/Search/Keyword?queryString=ADAMTS19) |
| ADAMTS2 | ECM Regulators | 25770910 | [25770910](https://www.ncbi.nlm.nih.gov/pubmed/25770910) | [ADAMTS2](https://www.genecards.org/Search/Keyword?queryString=ADAMTS2) |
| ADAMTS20 | ECM Regulators | 25770910 | [25770910](https://www.ncbi.nlm.nih.gov/pubmed/25770910) | [ADAMTS20](https://www.genecards.org/Search/Keyword?queryString=ADAMTS20) |
| ADAMTS3 | ECM Regulators | 25770910 | [25770910](https://www.ncbi.nlm.nih.gov/pubmed/25770910) | [ADAMTS3](https://www.genecards.org/Search/Keyword?queryString=ADAMTS3) |
| ADAMTS4 | ECM Regulators | 25770910 | [25770910](https://www.ncbi.nlm.nih.gov/pubmed/25770910) | [ADAMTS4](https://www.genecards.org/Search/Keyword?queryString=ADAMTS4) |
| ADAMTS5 | ECM Regulators | 25770910 | [25770910](https://www.ncbi.nlm.nih.gov/pubmed/25770910) | [ADAMTS5](https://www.genecards.org/Search/Keyword?queryString=ADAMTS5) |
| ADAMTS6 | ECM Regulators | 26634009 | [26634009](https://www.ncbi.nlm.nih.gov/pubmed/26634009) | [ADAMTS6](https://www.genecards.org/Search/Keyword?queryString=ADAMTS6) |
| ADAMTS7 | ECM Regulators | 25770910 | [25770910](https://www.ncbi.nlm.nih.gov/pubmed/25770910) | [ADAMTS7](https://www.genecards.org/Search/Keyword?queryString=ADAMTS7) |
| ADAMTS8 | ECM Regulators | 25770910 | [25770910](https://www.ncbi.nlm.nih.gov/pubmed/25770910) | [ADAMTS8](https://www.genecards.org/Search/Keyword?queryString=ADAMTS8) |
| ADAMTS9 | ECM Regulators | 25770910 | [25770910](https://www.ncbi.nlm.nih.gov/pubmed/25770910) | [ADAMTS9](https://www.genecards.org/Search/Keyword?queryString=ADAMTS9) |
| ADAMTSL1 | ECM Regulators | 25770910 | [25770910](https://www.ncbi.nlm.nih.gov/pubmed/25770910) | [ADAMTSL1](https://www.genecards.org/Search/Keyword?queryString=ADAMTSL1) |
| ADAMTSL2 | ECM Regulators | 25770910 | [25770910](https://www.ncbi.nlm.nih.gov/pubmed/25770910) | [ADAMTSL2](https://www.genecards.org/Search/Keyword?queryString=ADAMTSL2) |
| ADAMTSL3 | ECM Regulators | 25770910 | [25770910](https://www.ncbi.nlm.nih.gov/pubmed/25770910) | [ADAMTSL3](https://www.genecards.org/Search/Keyword?queryString=ADAMTSL3) |
| ADAMTSL4 | ECM Regulators | 25770910 | [25770910](https://www.ncbi.nlm.nih.gov/pubmed/25770910) | [ADAMTSL4](https://www.genecards.org/Search/Keyword?queryString=ADAMTSL4) |
| ADAMTSL5 | ECM Regulators | 27857980 | [27857980](https://www.ncbi.nlm.nih.gov/pubmed/27857980) | [ADAMTSL5](https://www.genecards.org/Search/Keyword?queryString=ADAMTSL5) |
| ADCY10 | ECM Micro-enviornment associated | 9788104 | [9788104](https://www.ncbi.nlm.nih.gov/pubmed/ 9788104) | [ADCY10](https://www.genecards.org/Search/Keyword?queryString=ADCY10) |
| ADGRL1 | ECM Micro-enviornment associated | 25713288 | [25713288](https://www.ncbi.nlm.nih.gov/pubmed/25713288) | [ADGRL1](https://www.genecards.org/Search/Keyword?queryString=ADGRL1) |
| ADGRL2 | ECM Micro-enviornment associated | 25713288 | [25713288](https://www.ncbi.nlm.nih.gov/pubmed/25713288) | [ADGRL2](https://www.genecards.org/Search/Keyword?queryString=ADGRL2) |
| ADGRL3 | ECM Micro-enviornment associated | 25713288 | [25713288](https://www.ncbi.nlm.nih.gov/pubmed/25713288) | [ADGRL3](https://www.genecards.org/Search/Keyword?queryString=ADGRL3) |
| ADIPOQ | ECM Glycoproteins | 28498357 | [28498357](https://www.ncbi.nlm.nih.gov/pubmed/28498357) | [ADIPOQ](https://www.genecards.org/Search/Keyword?queryString=ADIPOQ) |
| AEBP1 | ECM Glycoproteins | 28675934 | [28675934](https://www.ncbi.nlm.nih.gov/pubmed/28675934) | [AEBP1](https://www.genecards.org/Search/Keyword?queryString=AEBP1) |
| AGER | ECM Micro-enviornment associated | 17048769 | [17048769](https://www.ncbi.nlm.nih.gov/pubmed/17048769) | [AGER](https://www.genecards.org/Search/Keyword?queryString=AGER) |
| AGPS | ECM synthetic/degradation enzyme | 8336670 | [8336670](https://www.ncbi.nlm.nih.gov/pubmed/8336670) | [AGPS](https://www.genecards.org/Search/Keyword?queryString=AGPS) |
| AGRN | ECM Glycoproteins | 28319050 | [28319050](https://www.ncbi.nlm.nih.gov/pubmed/28319050) | [AGRN](https://www.genecards.org/Search/Keyword?queryString=AGRN) |
| AGT | ECM Regulators | 11108152 | [11108152](https://www.ncbi.nlm.nih.gov/pubmed/11108152) | [AGT](https://www.genecards.org/Search/Keyword?queryString=AGT) |
| AGTR1 | ECM Micro-enviornment associated | 11729229 | [11729229](https://www.ncbi.nlm.nih.gov/pubmed/11729229) | [AGTR1](https://www.genecards.org/Search/Keyword?queryString=AGTR1) |
| AKT1 | ECM Micro-enviornment associated | 26393468 | [26393468](https://www.ncbi.nlm.nih.gov/pubmed/26393468) | [AKT1](https://www.genecards.org/Search/Keyword?queryString=AKT1) |
| AKT2 | ECM Micro-enviornment associated | 17914025 | [17914025](https://www.ncbi.nlm.nih.gov/pubmed/17914025) | [AKT2](https://www.genecards.org/Search/Keyword?queryString=AKT2) |
| AKT3 | ECM Micro-enviornment associated | 21191416 | [21191416](https://www.ncbi.nlm.nih.gov/pubmed/21191416) | [AKT3](https://www.genecards.org/Search/Keyword?queryString=AKT3) |
| AMBN | ECM Glycoproteins | 15288841 | [15288841](https://www.ncbi.nlm.nih.gov/pubmed/15288841) | [AMBN](https://www.genecards.org/Search/Keyword?queryString=AMBN) |
| AMBP | ECM Regulators | 27559042 | [27559042](https://www.ncbi.nlm.nih.gov/pubmed/27559042) | [AMBP](https://www.genecards.org/Search/Keyword?queryString=AMBP) |
| AMELX | ECM Glycoproteins | 25789606 | [25789606](https://www.ncbi.nlm.nih.gov/pubmed/25789606) | [AMELX](https://www.genecards.org/Search/Keyword?queryString=AMELX) |
| AMELY | ECM Glycoproteins | 1734713 | [1734713](https://www.ncbi.nlm.nih.gov/pubmed/1734713) | [AMELY](https://www.genecards.org/Search/Keyword?queryString=AMELY) |
| AMH | Secreted Factors | 21637711 | [21637711](https://www.ncbi.nlm.nih.gov/pubmed/21637711) | [AMH](https://www.genecards.org/Search/Keyword?queryString=AMH) |
| ANGPT1 | Secreted Factors | 11447223 | [11447223](https://www.ncbi.nlm.nih.gov/pubmed/11447223) | [ANGPT1](https://www.genecards.org/Search/Keyword?queryString=ANGPT1) |
| ANGPT2 | Secreted Factors | 11447223 | [11447223](https://www.ncbi.nlm.nih.gov/pubmed/11447223) | [ANGPT2](https://www.genecards.org/Search/Keyword?queryString=ANGPT2) |
| ANGPT4 | Secreted Factors | 27335372 | [27335372](https://www.ncbi.nlm.nih.gov/pubmed/27335372) | [ANGPT4](https://www.genecards.org/Search/Keyword?queryString=ANGPT4) |
| ANGPTL1 | Secreted Factors | 28904065 | [28904065](https://www.ncbi.nlm.nih.gov/pubmed/28904065) | [ANGPTL1](https://www.genecards.org/Search/Keyword?queryString=ANGPTL1) |
| ANGPTL2 | Secreted Factors | 28946139 | [28946139](https://www.ncbi.nlm.nih.gov/pubmed/28946139) | [ANGPTL2](https://www.genecards.org/Search/Keyword?queryString=ANGPTL2) |
| ANGPTL3 | Secreted Factors | 27335372 | [27335372](https://www.ncbi.nlm.nih.gov/pubmed/27335372) | [ANGPTL3](https://www.genecards.org/Search/Keyword?queryString=ANGPTL3) |
| ANGPTL4 | Secreted Factors | 17068295 | [17068295](https://www.ncbi.nlm.nih.gov/pubmed/17068295) | [ANGPTL4](https://www.genecards.org/Search/Keyword?queryString=ANGPTL4) |
| ANGPTL5 | Secreted Factors | 24478758 | [24478758](https://www.ncbi.nlm.nih.gov/pubmed/24478758) | [ANGPTL5](https://www.genecards.org/Search/Keyword?queryString=ANGPTL5) |
| ANGPTL6 | Secreted Factors | 24478758 | [24478758](https://www.ncbi.nlm.nih.gov/pubmed/24478758) | [ANGPTL6](https://www.genecards.org/Search/Keyword?queryString=ANGPTL6) |
| ANGPTL7 | Secreted Factors | 24478758 | [24478758](https://www.ncbi.nlm.nih.gov/pubmed/24478758) | [ANGPTL7](https://www.genecards.org/Search/Keyword?queryString=ANGPTL7) |
| ANK1 | Cytoskeleton | 10574708 | [10574708](https://www.ncbi.nlm.nih.gov/pubmed/10574708) | [ANK1](https://www.genecards.org/Search/Keyword?queryString=ANK1) |
| ANK2 | Cytoskeleton | 21186323 | [21186323](https://www.ncbi.nlm.nih.gov/pubmed/21186323) | [ANK2](https://www.genecards.org/Search/Keyword?queryString=ANK2) |
| ANK3 | Cytoskeleton | 14757759 | [14757759](https://www.ncbi.nlm.nih.gov/pubmed/14757759) | [ANK3](https://www.genecards.org/Search/Keyword?queryString=ANK3) |
| ANXA1 | ECM-affiliated Proteins | 29614751 | [29614751](https://www.ncbi.nlm.nih.gov/pubmed/29614751) | [ANXA1](https://www.genecards.org/Search/Keyword?queryString=ANXA1) |
| ANXA10 | ECM-affiliated Proteins | 27071553 | [27071553](https://www.ncbi.nlm.nih.gov/pubmed/ 27071553) | [ANXA10](https://www.genecards.org/Search/Keyword?queryString=ANXA10) |
| ANXA11 | ECM-affiliated Proteins | 27071554 | [27071554](https://www.ncbi.nlm.nih.gov/pubmed/ 27071554) | [ANXA11](https://www.genecards.org/Search/Keyword?queryString=ANXA11) |
| ANXA13 | ECM-affiliated Proteins | 24936355 | [24936355](https://www.ncbi.nlm.nih.gov/pubmed/24936355) | [ANXA13](https://www.genecards.org/Search/Keyword?queryString=ANXA13) |
| ANXA2 | ECM-affiliated Proteins | 21131363 | [21131363](https://www.ncbi.nlm.nih.gov/pubmed/21131363) | [ANXA2](https://www.genecards.org/Search/Keyword?queryString=ANXA2) |
| ANXA3 | ECM-affiliated Proteins | 21131363 | [21131363](https://www.ncbi.nlm.nih.gov/pubmed/21131363) | [ANXA3](https://www.genecards.org/Search/Keyword?queryString=ANXA3) |
| ANXA4 | ECM-affiliated Proteins | 21131363 | [21131363](https://www.ncbi.nlm.nih.gov/pubmed/21131363) | [ANXA4](https://www.genecards.org/Search/Keyword?queryString=ANXA4) |
| ANXA5 | ECM-affiliated Proteins | 21131363 | [21131363](https://www.ncbi.nlm.nih.gov/pubmed/21131363) | [ANXA5](https://www.genecards.org/Search/Keyword?queryString=ANXA5) |
| ANXA6 | ECM-affiliated Proteins | 28071719 | [28071719](https://www.ncbi.nlm.nih.gov/pubmed/28071719) | [ANXA6](https://www.genecards.org/Search/Keyword?queryString=ANXA6) |
| ANXA7 | ECM-affiliated Proteins | 28071719 | [28071719](https://www.ncbi.nlm.nih.gov/pubmed/28071719) | [ANXA7](https://www.genecards.org/Search/Keyword?queryString=ANXA7) |
| ANXA8 | ECM-affiliated Proteins | 28060564 | [28060564](https://www.ncbi.nlm.nih.gov/pubmed/28060564) | [ANXA8](https://www.genecards.org/Search/Keyword?queryString=ANXA8) |
| ANXA8L1 | ECM-affiliated Proteins | 26893367 | [26893367](https://www.ncbi.nlm.nih.gov/pubmed/26893367) | [ANXA8L1](https://www.genecards.org/Search/Keyword?queryString=ANXA8L1) |
| ANXA9 | ECM-affiliated Proteins | 22159717 | [22159717](https://www.ncbi.nlm.nih.gov/pubmed/22159717 ) | [ANXA9](https://www.genecards.org/Search/Keyword?queryString=ANXA9) |
| APLP2 | ECM Micro-enviornment associated | 7616233 | [7616233](https://www.ncbi.nlm.nih.gov/pubmed/7616233) | [APLP2](https://www.genecards.org/Search/Keyword?queryString=APLP2) |
| APOA1 | ECM Micro-enviornment associated | 8621158 | [8621158](https://www.ncbi.nlm.nih.gov/pubmed/8621158) | [APOA1](https://www.genecards.org/Search/Keyword?queryString=APOA1) |
| APOA5 | ECM Micro-enviornment associated | 15774484 | [15774484](https://www.ncbi.nlm.nih.gov/pubmed/15774484) | [APOA5](https://www.genecards.org/Search/Keyword?queryString=APOA5) |
| APOB | ECM Micro-enviornment associated | 19646550 | [19646550](https://www.ncbi.nlm.nih.gov/pubmed/19646550) | [APOB](https://www.genecards.org/Search/Keyword?queryString=APOB) |
| APOC1 | ECM Micro-enviornment associated | 23376485 | [23376485](https://www.ncbi.nlm.nih.gov/pubmed/23376485) | [APOC1](https://www.genecards.org/Search/Keyword?queryString=APOC1) |
| APOC2 | ECM Micro-enviornment associated | 16314153 | [16314153](https://www.ncbi.nlm.nih.gov/pubmed/16314153) | [APOC2](https://www.genecards.org/Search/Keyword?queryString=APOC2) |
| APOC3 | ECM Micro-enviornment associated | 27559042 | [27559042](https://www.ncbi.nlm.nih.gov/pubmed/27559042) | [APOC3](https://www.genecards.org/Search/Keyword?queryString=APOC3) |
| APOC4 | ECM Micro-enviornment associated | 16335952 | [16335952](https://www.ncbi.nlm.nih.gov/pubmed/16335952) | [APOC4](https://www.genecards.org/Search/Keyword?queryString=APOC4) |
| APOE | ECM Micro-enviornment associated | 23103162 | [23103162](https://www.ncbi.nlm.nih.gov/pubmed/23103162) | [APOE](https://www.genecards.org/Search/Keyword?queryString=APOE) |
| APOL4 | ECM Micro-enviornment associated | 22261194 | [22261194](https://www.ncbi.nlm.nih.gov/pubmed/22261194) | [APOL4](https://www.genecards.org/Search/Keyword?queryString=APOL4) |
| APOLD1 | ECM Micro-enviornment associated | 27357248 | [27357248](https://www.ncbi.nlm.nih.gov/pubmed/27357248) | [APOLD1](https://www.genecards.org/Search/Keyword?queryString=APOLD1) |
| APOM | ECM Micro-enviornment associated | 23376485 | [23376485](https://www.ncbi.nlm.nih.gov/pubmed/23376485) | [APOM](https://www.genecards.org/Search/Keyword?queryString=APOM) |
| APP | ECM Micro-enviornment associated | 24117177 | [24117177](https://www.ncbi.nlm.nih.gov/pubmed/24117177) | [APP](https://www.genecards.org/Search/Keyword?queryString=APP) |
| ARAF | ECM Micro-enviornment associated | 8394352 | [8394352](https://www.ncbi.nlm.nih.gov/pubmed/8394352) | [ARAF](https://www.genecards.org/Search/Keyword?queryString=ARAF) |
| AREG | Secreted Factors | 25261255 | [25261255](https://www.ncbi.nlm.nih.gov/pubmed/25261255) | [AREG](https://www.genecards.org/Search/Keyword?queryString=AREG) |
| ARG1 | ECM synthetic/degradation enzyme | 20107769 | [20107769](https://www.ncbi.nlm.nih.gov/pubmed/20107769) | [ARG1](https://www.genecards.org/Search/Keyword?queryString=ARG1) |
| ARG2 | ECM synthetic/degradation enzyme | 20107769 | [20107769](https://www.ncbi.nlm.nih.gov/pubmed/20107769) | [ARG2](https://www.genecards.org/Search/Keyword?queryString=ARG2) |
| ARHGEF1 | ECM Micro-enviornment associated | 22086927 | [22086927](https://www.ncbi.nlm.nih.gov/pubmed/22086927) | [ARHGEF1](https://www.genecards.org/Search/Keyword?queryString=ARHGEF1) |
| ARHGEF12 | ECM Micro-enviornment associated | 19056867 | [19056867](https://www.ncbi.nlm.nih.gov/pubmed/19056867) | [ARHGEF12](https://www.genecards.org/Search/Keyword?queryString=ARHGEF12) |
| ARSG | ECM synthetic/degradation enzyme | 18283100 | [18283100](https://www.ncbi.nlm.nih.gov/pubmed/18283100) | [ARSG](https://www.genecards.org/Search/Keyword?queryString=ARSG) |
| ARSH | ECM Micro-enviornment associated | 2104833 | [2104833](https://www.ncbi.nlm.nih.gov/pubmed/2104833) | [ARSH](https://www.genecards.org/Search/Keyword?queryString=ARSH) |
| ARSJ | ECM Micro-enviornment associated | 23144729 | [23144729](https://www.ncbi.nlm.nih.gov/pubmed/23144729) | [ARSJ](https://www.genecards.org/Search/Keyword?queryString=ARSJ) |
| ARSK | ECM Micro-enviornment associated | 23454643 | [23454643](https://www.ncbi.nlm.nih.gov/pubmed/23454643) | [ARSK](https://www.genecards.org/Search/Keyword?queryString=ARSK) |
| ARTN | Secreted Factors | 22159717 | [22159717](https://www.ncbi.nlm.nih.gov/pubmed/22159717 ) | [ARTN](https://www.genecards.org/Search/Keyword?queryString=ARTN) |
| ASPN | Proteoglycans | 23419153 | [23419153](https://www.ncbi.nlm.nih.gov/pubmed/23419153) | [ASPN](https://www.genecards.org/Search/Keyword?queryString=ASPN) |
| ASTL | ECM Regulators | 22159717 | [22159717](https://www.ncbi.nlm.nih.gov/pubmed/22159717 ) | [ASTL](https://www.genecards.org/Search/Keyword?queryString=ASTL) |
| ATHS | ECM Micro-enviornment associated | 8830938 | [8830938](https://www.ncbi.nlm.nih.gov/pubmed/8830938) | [ATHS](https://www.genecards.org/Search/Keyword?queryString=ATHS) |
| ATP1A3 | ECM Micro-enviornment associated | 24769233 | [24769233](https://www.ncbi.nlm.nih.gov/pubmed/24769233) | [ATP1A3](https://www.genecards.org/Search/Keyword?queryString=ATP1A3) |
| AZU1 | ECM Micro-enviornment associated | 11865065 | [11865065](https://www.ncbi.nlm.nih.gov/pubmed/11865065) | [AZU1](https://www.genecards.org/Search/Keyword?queryString=AZU1) |
| B2M | ECM Micro-enviornment associated | 18164932 | [18164932](https://www.ncbi.nlm.nih.gov/pubmed/18164932) | [B2M](https://www.genecards.org/Search/Keyword?queryString=B2M) |
| B3GALT6 | ECM synthetic/degradation enzyme | 11551958 | [11551958](https://www.ncbi.nlm.nih.gov/pubmed/11551958) | [B3GALT6](https://www.genecards.org/Search/Keyword?queryString=B3GALT6) |
| B3GAT1 | ECM synthetic/degradation enzyme | 8780385 | [8780385](https://www.ncbi.nlm.nih.gov/pubmed/8780385) | [B3GAT1](https://www.genecards.org/Search/Keyword?queryString=B3GAT1) |
| B3GAT2 | ECM synthetic/degradation enzyme | 12511570 | [12511570](https://www.ncbi.nlm.nih.gov/pubmed/12511570) | [B3GAT2](https://www.genecards.org/Search/Keyword?queryString=B3GAT2) |
| B3GAT3 | ECM synthetic/degradation enzyme | 10526176 | [10526176](https://www.ncbi.nlm.nih.gov/pubmed/10526176) | [B3GAT3](https://www.genecards.org/Search/Keyword?queryString=B3GAT3) |
| B4GALT7 | ECM synthetic/degradation enzyme | 10473568 | [10473568](https://www.ncbi.nlm.nih.gov/pubmed/10473568) | [B4GALT7](https://www.genecards.org/Search/Keyword?queryString=B4GALT7) |
| BCAN | Proteoglycans | 23419153 | [23419153](https://www.ncbi.nlm.nih.gov/pubmed/23419153) | [BCAN](https://www.genecards.org/Search/Keyword?queryString=BCAN) |
| BCAR1 | ECM Micro-enviornment associated | 10587647 | [10587647](https://www.ncbi.nlm.nih.gov/pubmed/10587647) | [BCAR1](https://www.genecards.org/Search/Keyword?queryString=BCAR1) |
| BDNF | Secreted Factors | 19267421 | [19267421](https://www.ncbi.nlm.nih.gov/pubmed/19267421) | [BDNF](https://www.genecards.org/Search/Keyword?queryString=BDNF) |
| BGLAP | ECM Glycoproteins | 18547474 | [18547474](https://www.ncbi.nlm.nih.gov/pubmed/18547474) | [BGLAP](https://www.genecards.org/Search/Keyword?queryString=BGLAP) |
| BGN | Proteoglycans | 23419153 | [23419153](https://www.ncbi.nlm.nih.gov/pubmed/23419153) | [BGN](https://www.genecards.org/Search/Keyword?queryString=BGN) |
| BMP1 | ECM Regulators | 17560775 | [17560775](https://www.ncbi.nlm.nih.gov/pubmed/17560775) | [BMP1](https://www.genecards.org/Search/Keyword?queryString=BMP1) |
| BMP10 | Secreted Factors | 26631724 | [26631724](https://www.ncbi.nlm.nih.gov/pubmed/26631724) | [BMP10](https://www.genecards.org/Search/Keyword?queryString=BMP10) |
| BMP15 | Secreted Factors | 23382188 | [23382188](https://www.ncbi.nlm.nih.gov/pubmed/23382188) | [BMP15](https://www.genecards.org/Search/Keyword?queryString=BMP15) |
| BMP2 | Secreted Factors | 10218963 | [10218963](https://www.ncbi.nlm.nih.gov/pubmed/10218963) | [BMP2](https://www.genecards.org/Search/Keyword?queryString=BMP2) |
| BMP3 | Secreted Factors | 25401122 | [25401122](https://www.ncbi.nlm.nih.gov/pubmed/25401122) | [BMP3](https://www.genecards.org/Search/Keyword?queryString=BMP3) |
| BMP4 | Secreted Factors | 25401122 | [25401122](https://www.ncbi.nlm.nih.gov/pubmed/25401122) | [BMP4](https://www.genecards.org/Search/Keyword?queryString=BMP4) |
| BMP5 | Secreted Factors | 25401122 | [25401122](https://www.ncbi.nlm.nih.gov/pubmed/25401122) | [BMP5](https://www.genecards.org/Search/Keyword?queryString=BMP5) |
| BMP6 | Secreted Factors | 25401122 | [25401122](https://www.ncbi.nlm.nih.gov/pubmed/25401122) | [BMP6](https://www.genecards.org/Search/Keyword?queryString=BMP6) |
| BMP7 | Secreted Factors | 25401122 | [25401122](https://www.ncbi.nlm.nih.gov/pubmed/25401122) | [BMP7](https://www.genecards.org/Search/Keyword?queryString=BMP7) |
| BMP8A | Secreted Factors | 19272164 | [19272164](https://www.ncbi.nlm.nih.gov/pubmed/19272164) | [BMP8A](https://www.genecards.org/Search/Keyword?queryString=BMP8A) |
| BMP8B | Secreted Factors | 22159717 | [22159717](https://www.ncbi.nlm.nih.gov/pubmed/22159717 ) | [BMP8B](https://www.genecards.org/Search/Keyword?queryString=BMP8B) |
| BMPER | ECM Glycoproteins | 18787191 | [18787191](https://www.ncbi.nlm.nih.gov/pubmed/18787191) | [BMPER](https://www.genecards.org/Search/Keyword?queryString=BMPER) |
| BMPR1B | ECM receptor | 15775969 | [15775969](https://www.ncbi.nlm.nih.gov/pubmed/15775969) | [BMPR1B](https://www.genecards.org/Search/Keyword?queryString=BMPR1B) |
| BMPR2 | ECM receptor | 11327700 | [11327700](https://www.ncbi.nlm.nih.gov/pubmed/11327700) | [BMPR2](https://www.genecards.org/Search/Keyword?queryString=BMPR2) |
| BRAF | ECM Micro-enviornment associated | 21451543 | [21451543](https://www.ncbi.nlm.nih.gov/pubmed/21451543) | [BRAF](https://www.genecards.org/Search/Keyword?queryString=BRAF) |
| BRINP2 | Secreted Factors | 22159717 | [22159717](https://www.ncbi.nlm.nih.gov/pubmed/22159717 ) | [BRINP2](https://www.genecards.org/Search/Keyword?queryString=BRINP2) |
| BRINP3 | Secreted Factors | 22159717 | [22159717](https://www.ncbi.nlm.nih.gov/pubmed/22159717 ) | [BRINP3](https://www.genecards.org/Search/Keyword?queryString=BRINP3) |
| BSPH1 | ECM Glycoproteins | 3606570 | [3606570](https://www.ncbi.nlm.nih.gov/pubmed/3606570) | [BSPH1](https://www.genecards.org/Search/Keyword?queryString=BSPH1) |
| BTC | Secreted Factors | 28829951 | [28829951](https://www.ncbi.nlm.nih.gov/pubmed/28829951) | [BTC](https://www.genecards.org/Search/Keyword?queryString=BTC) |
| BTRC | ECM Micro-enviornment associated | 16757476 | [16757476](https://www.ncbi.nlm.nih.gov/pubmed/16757476) | [BTRC](https://www.genecards.org/Search/Keyword?queryString=BTRC) |
| C17orf58 | ECM Regulators | 22159717 | [22159717](https://www.ncbi.nlm.nih.gov/pubmed/22159717 ) | [C17orf58](https://www.genecards.org/Search/Keyword?queryString=C17orf58) |
| C1QA | ECM-affiliated Proteins | 22159717 | [22159717](https://www.ncbi.nlm.nih.gov/pubmed/22159717 ) | [C1QA](https://www.genecards.org/Search/Keyword?queryString=C1QA) |
| C1QB | ECM-affiliated Proteins | 22159717 | [22159717](https://www.ncbi.nlm.nih.gov/pubmed/22159717 ) | [C1QB](https://www.genecards.org/Search/Keyword?queryString=C1QB) |
| C1QC | ECM-affiliated Proteins | 22159717 | [22159717](https://www.ncbi.nlm.nih.gov/pubmed/22159717 ) | [C1QC](https://www.genecards.org/Search/Keyword?queryString=C1QC) |
| C1QL1 | ECM-affiliated Proteins | 25424900 | [25424900](https://www.ncbi.nlm.nih.gov/pubmed/25424900) | [C1QL1](https://www.genecards.org/Search/Keyword?queryString=C1QL1) |
| C1QL2 | ECM-affiliated Proteins | 25424900 | [25424900](https://www.ncbi.nlm.nih.gov/pubmed/25424900) | [C1QL2](https://www.genecards.org/Search/Keyword?queryString=C1QL2) |
| C1QL3 | ECM-affiliated Proteins | 25424900 | [25424900](https://www.ncbi.nlm.nih.gov/pubmed/25424900) | [C1QL3](https://www.genecards.org/Search/Keyword?queryString=C1QL3) |
| C1QL4 | ECM-affiliated Proteins | 25424900 | [25424900](https://www.ncbi.nlm.nih.gov/pubmed/25424900) | [C1QL4](https://www.genecards.org/Search/Keyword?queryString=C1QL4) |
| C1QTNF1 | ECM-affiliated Proteins | 22159717 | [22159717](https://www.ncbi.nlm.nih.gov/pubmed/22159717 ) | [C1QTNF1](https://www.genecards.org/Search/Keyword?queryString=C1QTNF1) |
| C1QTNF2 | ECM-affiliated Proteins | 22159717 | [22159717](https://www.ncbi.nlm.nih.gov/pubmed/22159717 ) | [C1QTNF2](https://www.genecards.org/Search/Keyword?queryString=C1QTNF2) |
| C1QTNF3 | ECM-affiliated Proteins | 22159717 | [22159717](https://www.ncbi.nlm.nih.gov/pubmed/22159717 ) | [C1QTNF3](https://www.genecards.org/Search/Keyword?queryString=C1QTNF3) |
| C1QTNF4 | ECM-affiliated Proteins | 22159717 | [22159717](https://www.ncbi.nlm.nih.gov/pubmed/22159717 ) | [C1QTNF4](https://www.genecards.org/Search/Keyword?queryString=C1QTNF4) |
| C1QTNF5 | ECM-affiliated Proteins | 22159717 | [22159717](https://www.ncbi.nlm.nih.gov/pubmed/22159717 ) | [C1QTNF5](https://www.genecards.org/Search/Keyword?queryString=C1QTNF5) |
| C1QTNF6 | ECM-affiliated Proteins | 22159717 | [22159717](https://www.ncbi.nlm.nih.gov/pubmed/22159717 ) | [C1QTNF6](https://www.genecards.org/Search/Keyword?queryString=C1QTNF6) |
| C1QTNF7 | ECM-affiliated Proteins | 22159717 | [22159717](https://www.ncbi.nlm.nih.gov/pubmed/22159717 ) | [C1QTNF7](https://www.genecards.org/Search/Keyword?queryString=C1QTNF7) |
| C1QTNF8 | ECM-affiliated Proteins | 22159717 | [22159717](https://www.ncbi.nlm.nih.gov/pubmed/22159717 ) | [C1QTNF8](https://www.genecards.org/Search/Keyword?queryString=C1QTNF8) |
| C1QTNF9 | ECM-affiliated Proteins | 22159717 | [22159717](https://www.ncbi.nlm.nih.gov/pubmed/22159717 ) | [C1QTNF9](https://www.genecards.org/Search/Keyword?queryString=C1QTNF9) |
| C1QTNF9B | Secreted Factors | 22159717 | [22159717](https://www.ncbi.nlm.nih.gov/pubmed/22159717 ) | [C1QTNF9B](https://www.genecards.org/Search/Keyword?queryString=C1QTNF9B) |
| C4BPA | ECM Micro-enviornment associated | 18266273 | [18266273](https://www.ncbi.nlm.nih.gov/pubmed/18266273) | [C4BPA](https://www.genecards.org/Search/Keyword?queryString=C4BPA) |
| CABP1 | ECM Micro-enviornment associated | 22529935 | [22529935](https://www.ncbi.nlm.nih.gov/pubmed/22529935) | [CABP1](https://www.genecards.org/Search/Keyword?queryString=CABP1) |
| CABP2 | ECM Micro-enviornment associated | 22529935 | [22529935](https://www.ncbi.nlm.nih.gov/pubmed/22529935) | [CABP2](https://www.genecards.org/Search/Keyword?queryString=CABP2) |
| CADM1 | Cell adhesion molecule | 24465823 | [24465823](https://www.ncbi.nlm.nih.gov/pubmed/24465823) | [CADM1](https://www.genecards.org/Search/Keyword?queryString=CADM1) |
| CADM3 | Cell adhesion molecule | 12826663 | [12826663](https://www.ncbi.nlm.nih.gov/pubmed/12826663) | [CADM3](https://www.genecards.org/Search/Keyword?queryString=CADM3) |
| CADM4 | Cell adhesion molecule | 19565570 | [19565570](https://www.ncbi.nlm.nih.gov/pubmed/19565570) | [CADM4](https://www.genecards.org/Search/Keyword?queryString=CADM4) |
| CADPS | ECM Micro-enviornment associated | 26899371 | [26899371](https://www.ncbi.nlm.nih.gov/pubmed/26899371) | [CADPS](https://www.genecards.org/Search/Keyword?queryString=CADPS) |
| CALCRL | ECM Micro-enviornment associated | 19898635 | [19898635](https://www.ncbi.nlm.nih.gov/pubmed/19898635) | [CALCRL](https://www.genecards.org/Search/Keyword?queryString=CALCRL) |
| CALM1 | ECM Micro-enviornment associated | 23743201 | [23743201](https://www.ncbi.nlm.nih.gov/pubmed/23743201) | [CALM1](https://www.genecards.org/Search/Keyword?queryString=CALM1) |
| CALM2 | ECM Micro-enviornment associated | 29463791 | [29463791](https://www.ncbi.nlm.nih.gov/pubmed/29463791) | [CALM2](https://www.genecards.org/Search/Keyword?queryString=CALM2) |
| CAMK1D | ECM Micro-enviornment associated | 19383354 | [19383354](https://www.ncbi.nlm.nih.gov/pubmed/19383354) | [CAMK1D](https://www.genecards.org/Search/Keyword?queryString=CAMK1D) |
| CAMK2A | ECM Micro-enviornment associated | 12954639 | [12954639](https://www.ncbi.nlm.nih.gov/pubmed/12954639) | [CAMK2A](https://www.genecards.org/Search/Keyword?queryString=CAMK2A) |
| CAMK2B | ECM Micro-enviornment associated | 28130256 | [28130256](https://www.ncbi.nlm.nih.gov/pubmed/28130256) | [CAMK2B](https://www.genecards.org/Search/Keyword?queryString=CAMK2B) |
| CAMK2D | ECM Micro-enviornment associated | 28130256 | [28130256](https://www.ncbi.nlm.nih.gov/pubmed/28130256) | [CAMK2D](https://www.genecards.org/Search/Keyword?queryString=CAMK2D) |
| CAMK2G | ECM Micro-enviornment associated | 18248957 | [18248957](https://www.ncbi.nlm.nih.gov/pubmed/18248957) | [CAMK2G](https://www.genecards.org/Search/Keyword?queryString=CAMK2G) |
| CAMK2N2 | ECM Micro-enviornment associated | 12531901 | [12531901](https://www.ncbi.nlm.nih.gov/pubmed/12531901) | [CAMK2N2](https://www.genecards.org/Search/Keyword?queryString=CAMK2N2) |
| CAMP | ECM Micro-enviornment associated | 19858184 | [19858184](https://www.ncbi.nlm.nih.gov/pubmed/19858184) | [CAMP](https://www.genecards.org/Search/Keyword?queryString=CAMP) |
| CANT1 | ECM Micro-enviornment associated | 7513202 | [7513202](https://www.ncbi.nlm.nih.gov/pubmed/7513202) | [CANT1](https://www.genecards.org/Search/Keyword?queryString=CANT1) |
| CASK | ECM Micro-enviornment associated | 10460248 | [10460248](https://www.ncbi.nlm.nih.gov/pubmed/10460248) | [CASK](https://www.genecards.org/Search/Keyword?queryString=CASK) |
| CASP3 | ECM Micro-enviornment associated | 17406026 | [17406026](https://www.ncbi.nlm.nih.gov/pubmed/17406026) | [CASP3](https://www.genecards.org/Search/Keyword?queryString=CASP3) |
| CASR | ECM Micro-enviornment associated | 11062022 | [11062022](https://www.ncbi.nlm.nih.gov/pubmed/11062022) | [CASR](https://www.genecards.org/Search/Keyword?queryString=CASR) |
| CAT | ECM Micro-enviornment associated | 21423176 | [21423176](https://www.ncbi.nlm.nih.gov/pubmed/21423176) | [CAT](https://www.genecards.org/Search/Keyword?queryString=CAT) |
| CAV1 | ECM Micro-enviornment associated | 19887621 | [19887621](https://www.ncbi.nlm.nih.gov/pubmed/19887621) | [CAV1](https://www.genecards.org/Search/Keyword?queryString=CAV1) |
| CAV2 | ECM Micro-enviornment associated | 8552590 | [8552590](https://www.ncbi.nlm.nih.gov/pubmed/8552590) | [CAV2](https://www.genecards.org/Search/Keyword?queryString=CAV2) |
| CAV3 | ECM Micro-enviornment associated | 10988290 | [10988290](https://www.ncbi.nlm.nih.gov/pubmed/10988290) | [CAV3](https://www.genecards.org/Search/Keyword?queryString=CAV3) |
| CBL | ECM Micro-enviornment associated | 20717917 | [20717917](https://www.ncbi.nlm.nih.gov/pubmed/20717917) | [CBL](https://www.genecards.org/Search/Keyword?queryString=CBL) |
| CBLB | ECM Micro-enviornment associated | 12697763 | [12697763](https://www.ncbi.nlm.nih.gov/pubmed/12697763) | [CBLB](https://www.genecards.org/Search/Keyword?queryString=CBLB) |
| CBLC | ECM Micro-enviornment associated | 23376485 | [23376485](https://www.ncbi.nlm.nih.gov/pubmed/23376485) | [CBLC](https://www.genecards.org/Search/Keyword?queryString=CBLC) |
| CBLN1 | Secreted Factors | 29867379 | [29867379](https://www.ncbi.nlm.nih.gov/pubmed/29867379) | [CBLN1](https://www.genecards.org/Search/Keyword?queryString=CBLN1) |
| CBLN2 | Secreted Factors | 29867379 | [29867379](https://www.ncbi.nlm.nih.gov/pubmed/29867379) | [CBLN2](https://www.genecards.org/Search/Keyword?queryString=CBLN2) |
| CBLN3 | Secreted Factors | 29867379 | [29867379](https://www.ncbi.nlm.nih.gov/pubmed/29867379) | [CBLN3](https://www.genecards.org/Search/Keyword?queryString=CBLN3) |
| CBLN4 | Secreted Factors | 29867379 | [29867379](https://www.ncbi.nlm.nih.gov/pubmed/29867379) | [CBLN4](https://www.genecards.org/Search/Keyword?queryString=CBLN4) |
| CCBE1 | Secreted Factors | 19287381 | [19287381](https://www.ncbi.nlm.nih.gov/pubmed/19287381) | [CCBE1](https://www.genecards.org/Search/Keyword?queryString=CCBE1) |
| CCL1 | Secreted Factors | 22479563 | [22479563](https://www.ncbi.nlm.nih.gov/pubmed/22479563) | [CCL1](https://www.genecards.org/Search/Keyword?queryString=CCL1) |
| CCL11 | Secreted Factors | 22479563 | [22479563](https://www.ncbi.nlm.nih.gov/pubmed/22479563) | [CCL11](https://www.genecards.org/Search/Keyword?queryString=CCL11) |
| CCL13 | Secreted Factors | 22159717 | [22159717](https://www.ncbi.nlm.nih.gov/pubmed/22159717 ) | [CCL13](https://www.genecards.org/Search/Keyword?queryString=CCL13) |
| CCL14 | Secreted Factors | 22159717 | [22159717](https://www.ncbi.nlm.nih.gov/pubmed/22159717 ) | [CCL14](https://www.genecards.org/Search/Keyword?queryString=CCL14) |
| CCL15 | Secreted Factors | 22159717 | [22159717](https://www.ncbi.nlm.nih.gov/pubmed/22159717 ) | [CCL15](https://www.genecards.org/Search/Keyword?queryString=CCL15) |
| CCL16 | Secreted Factors | 22159717 | [22159717](https://www.ncbi.nlm.nih.gov/pubmed/22159717 ) | [CCL16](https://www.genecards.org/Search/Keyword?queryString=CCL16) |
| CCL17 | Secreted Factors | 22159717 | [22159717](https://www.ncbi.nlm.nih.gov/pubmed/22159717 ) | [CCL17](https://www.genecards.org/Search/Keyword?queryString=CCL17) |
| CCL18 | Secreted Factors | 22159717 | [22159717](https://www.ncbi.nlm.nih.gov/pubmed/22159717 ) | [CCL18](https://www.genecards.org/Search/Keyword?queryString=CCL18) |
| CCL19 | Secreted Factors | 22159717 | [22159717](https://www.ncbi.nlm.nih.gov/pubmed/22159717 ) | [CCL19](https://www.genecards.org/Search/Keyword?queryString=CCL19) |
| CCL2 | Secreted Factors | 22159717 | [22159717](https://www.ncbi.nlm.nih.gov/pubmed/22159717 ) | [CCL2](https://www.genecards.org/Search/Keyword?queryString=CCL2) |
| CCL20 | Secreted Factors | 22159717 | [22159717](https://www.ncbi.nlm.nih.gov/pubmed/22159717 ) | [CCL20](https://www.genecards.org/Search/Keyword?queryString=CCL20) |
| CCL21 | Secreted Factors | 22159717 | [22159717](https://www.ncbi.nlm.nih.gov/pubmed/22159717 ) | [CCL21](https://www.genecards.org/Search/Keyword?queryString=CCL21) |
| CCL22 | Secreted Factors | 22159717 | [22159717](https://www.ncbi.nlm.nih.gov/pubmed/22159717 ) | [CCL22](https://www.genecards.org/Search/Keyword?queryString=CCL22) |
| CCL23 | Secreted Factors | 22159717 | [22159717](https://www.ncbi.nlm.nih.gov/pubmed/22159717 ) | [CCL23](https://www.genecards.org/Search/Keyword?queryString=CCL23) |
| CCL24 | Secreted Factors | 22159717 | [22159717](https://www.ncbi.nlm.nih.gov/pubmed/22159717 ) | [CCL24](https://www.genecards.org/Search/Keyword?queryString=CCL24) |
| CCL25 | Secreted Factors | 22159717 | [22159717](https://www.ncbi.nlm.nih.gov/pubmed/22159717 ) | [CCL25](https://www.genecards.org/Search/Keyword?queryString=CCL25) |
| CCL26 | Secreted Factors | 22159717 | [22159717](https://www.ncbi.nlm.nih.gov/pubmed/22159717 ) | [CCL26](https://www.genecards.org/Search/Keyword?queryString=CCL26) |
| CCL27 | Secreted Factors | 22159717 | [22159717](https://www.ncbi.nlm.nih.gov/pubmed/22159717 ) | [CCL27](https://www.genecards.org/Search/Keyword?queryString=CCL27) |
| CCL28 | Secreted Factors | 22159717 | [22159717](https://www.ncbi.nlm.nih.gov/pubmed/22159717 ) | [CCL28](https://www.genecards.org/Search/Keyword?queryString=CCL28) |
| CCL3 | Secreted Factors | 28057004 | [28057004](https://www.ncbi.nlm.nih.gov/pubmed/28057004) | [CCL3](https://www.genecards.org/Search/Keyword?queryString=CCL3) |
| CCL3L3 | Secreted Factors | 22159717 | [22159717](https://www.ncbi.nlm.nih.gov/pubmed/22159717 ) | [CCL3L3](https://www.genecards.org/Search/Keyword?queryString=CCL3L3) |
| CCL4 | Secreted Factors | 28057004 | [28057004](https://www.ncbi.nlm.nih.gov/pubmed/28057004) | [CCL4](https://www.genecards.org/Search/Keyword?queryString=CCL4) |
| CCL4L1 | Secreted Factors | 22159717 | [22159717](https://www.ncbi.nlm.nih.gov/pubmed/22159717 ) | [CCL4L1](https://www.genecards.org/Search/Keyword?queryString=CCL4L1) |
| CCL4L2 | Secreted Factors | 22159717 | [22159717](https://www.ncbi.nlm.nih.gov/pubmed/22159717 ) | [CCL4L2](https://www.genecards.org/Search/Keyword?queryString=CCL4L2) |
| CCL5 | Secreted Factors | 22159717 | [22159717](https://www.ncbi.nlm.nih.gov/pubmed/22159717 ) | [CCL5](https://www.genecards.org/Search/Keyword?queryString=CCL5) |
| CCL7 | Secreted Factors | 28057004 | [28057004](https://www.ncbi.nlm.nih.gov/pubmed/28057004) | [CCL7](https://www.genecards.org/Search/Keyword?queryString=CCL7) |
| CCL8 | Secreted Factors | 28057004 | [28057004](https://www.ncbi.nlm.nih.gov/pubmed/28057004) | [CCL8](https://www.genecards.org/Search/Keyword?queryString=CCL8) |
| CCND1 | ECM Micro-enviornment associated | 23839032 | [23839032](https://www.ncbi.nlm.nih.gov/pubmed/23839032) | [CCND1](https://www.genecards.org/Search/Keyword?queryString=CCND1) |
| CCR1 | ECM Micro-enviornment associated | 18367676 | [18367676](https://www.ncbi.nlm.nih.gov/pubmed/18367676) | [CCR1](https://www.genecards.org/Search/Keyword?queryString=CCR1) |
| CCR5 | ECM Micro-enviornment associated | 10943863 | [10943863](https://www.ncbi.nlm.nih.gov/pubmed/10943863) | [CCR5](https://www.genecards.org/Search/Keyword?queryString=CCR5) |
| CD109 | ECM Regulators | 22694813 | [22694813](https://www.ncbi.nlm.nih.gov/pubmed/22694813) | [CD109](https://www.genecards.org/Search/Keyword?queryString=CD109) |
| CD209 | ECM-affiliated Proteins | 25940995 | [25940995](https://www.ncbi.nlm.nih.gov/pubmed/25940995) | [CD209](https://www.genecards.org/Search/Keyword?queryString=CD209) |
| CD36 | Cell adhesion molecule | 18808690 | [18808690](https://www.ncbi.nlm.nih.gov/pubmed/18808690) | [CD36](https://www.genecards.org/Search/Keyword?queryString=CD36) |
| CD4 | Cell adhesion molecule | 18078954 | [18078954](https://www.ncbi.nlm.nih.gov/pubmed/18078954) | [CD4](https://www.genecards.org/Search/Keyword?queryString=CD4) |
| CD44 | Cell adhesion molecule | 10950950 | [10950950](https://www.ncbi.nlm.nih.gov/pubmed/10950950) | [CD44](https://www.genecards.org/Search/Keyword?queryString=CD44) |
| CD47 | Cell adhesion molecule | 19636412 | [19636412](https://www.ncbi.nlm.nih.gov/pubmed/19636412) | [CD47](https://www.genecards.org/Search/Keyword?queryString=CD47) |
| CD58 | Cell adhesion molecule | 9292690 | [9292690](https://www.ncbi.nlm.nih.gov/pubmed/9292690) | [CD58](https://www.genecards.org/Search/Keyword?queryString=CD58) |
| CD63 | Cell adhesion molecule | 12705901 | [12705901](https://www.ncbi.nlm.nih.gov/pubmed/12705901) | [CD63](https://www.genecards.org/Search/Keyword?queryString=CD63) |
| CD86 | Cell adhesion molecule | 7664487 | [7664487](https://www.ncbi.nlm.nih.gov/pubmed/7664487) | [CD86](https://www.genecards.org/Search/Keyword?queryString=CD86) |
| CD9 | Cell adhesion molecule | 17203208 | [17203208](https://www.ncbi.nlm.nih.gov/pubmed/17203208) | [CD9](https://www.genecards.org/Search/Keyword?queryString=CD9) |
| CD93 | Cell adhesion molecule | 9136074 | [9136074](https://www.ncbi.nlm.nih.gov/pubmed/9136074) | [CD93](https://www.genecards.org/Search/Keyword?queryString=CD93) |
| CDC42 | ECM Micro-enviornment associated | 10587647 | [10587647](https://www.ncbi.nlm.nih.gov/pubmed/10587647) | [CDC42](https://www.genecards.org/Search/Keyword?queryString=CDC42) |
| CDCP2 | ECM Glycoproteins | 20501830 | [20501830](https://www.ncbi.nlm.nih.gov/pubmed/20501830) | [CDCP2](https://www.genecards.org/Search/Keyword?queryString=CDCP2) |
| CDH1 | Cell adhesion molecule | 21377268 | [21377268](https://www.ncbi.nlm.nih.gov/pubmed/21377268) | [CDH1](https://www.genecards.org/Search/Keyword?queryString=CDH1) |
| CDH10 | Cell adhesion molecule | 18084254 | [18084254](https://www.ncbi.nlm.nih.gov/pubmed/18084254) | [CDH10](https://www.genecards.org/Search/Keyword?queryString=CDH10) |
| CDH11 | Cell adhesion molecule | 23376485 | [23376485](https://www.ncbi.nlm.nih.gov/pubmed/23376485) | [CDH11](https://www.genecards.org/Search/Keyword?queryString=CDH11) |
| CDH17 | Cell adhesion molecule | 27035870 | [27035870](https://www.ncbi.nlm.nih.gov/pubmed/27035870) | [CDH17](https://www.genecards.org/Search/Keyword?queryString=CDH17) |
| CDH23 | Cell adhesion molecule | 11138009 | [11138009](https://www.ncbi.nlm.nih.gov/pubmed/11138009) | [CDH23](https://www.genecards.org/Search/Keyword?queryString=CDH23) |
| CDH24 | Cell adhesion molecule | 12734196 | [12734196](https://www.ncbi.nlm.nih.gov/pubmed/12734196) | [CDH24](https://www.genecards.org/Search/Keyword?queryString=CDH24) |
| CDH3 | Cell adhesion molecule | 9473155 | [9473155](https://www.ncbi.nlm.nih.gov/pubmed/9473155) | [CDH3](https://www.genecards.org/Search/Keyword?queryString=CDH3) |
| CDH4 | Cell adhesion molecule | 7982033 | [7982033](https://www.ncbi.nlm.nih.gov/pubmed/7982033) | [CDH4](https://www.genecards.org/Search/Keyword?queryString=CDH4) |
| CDH5 | Cell adhesion molecule | 25978380 | [25978380](https://www.ncbi.nlm.nih.gov/pubmed/25978380) | [CDH5](https://www.genecards.org/Search/Keyword?queryString=CDH5) |
| CDH6 | Cell adhesion molecule | 23376485 | [23376485](https://www.ncbi.nlm.nih.gov/pubmed/23376485) | [CDH6](https://www.genecards.org/Search/Keyword?queryString=CDH6) |
| CDH7 | Cell adhesion molecule | 10995570 | [10995570](https://www.ncbi.nlm.nih.gov/pubmed/10995570) | [CDH7](https://www.genecards.org/Search/Keyword?queryString=CDH7) |
| CDH8 | Cell adhesion molecule | 2059658 | [2059658](https://www.ncbi.nlm.nih.gov/pubmed/2059658) | [CDH8](https://www.genecards.org/Search/Keyword?queryString=CDH8) |
| CDH9 | Cell adhesion molecule | 2059658 | [2059658](https://www.ncbi.nlm.nih.gov/pubmed/2059658) | [CDH9](https://www.genecards.org/Search/Keyword?queryString=CDH9) |
| CDHR5 | Cell adhesion molecule | 19056867 | [19056867](https://www.ncbi.nlm.nih.gov/pubmed/19056867) | [CDHR5](https://www.genecards.org/Search/Keyword?queryString=CDHR5) |
| CDKN1A | ECM Micro-enviornment associated | 12517948 | [12517948](https://www.ncbi.nlm.nih.gov/pubmed/12517948) | [CDKN1A](https://www.genecards.org/Search/Keyword?queryString=CDKN1A) |
| CELA1 | ECM Regulators | 24949484 | [24949484](https://www.ncbi.nlm.nih.gov/pubmed/24949484) | [CELA1](https://www.genecards.org/Search/Keyword?queryString=CELA1) |
| CELA2A | ECM Regulators | 28071719 | [28071719](https://www.ncbi.nlm.nih.gov/pubmed/28071719) | [CELA2A](https://www.genecards.org/Search/Keyword?queryString=CELA2A) |
| CELA2B | ECM Regulators | 22159717 | [22159717](https://www.ncbi.nlm.nih.gov/pubmed/22159717 ) | [CELA2B](https://www.genecards.org/Search/Keyword?queryString=CELA2B) |
| CELA3A | ECM Regulators | 22159717 | [22159717](https://www.ncbi.nlm.nih.gov/pubmed/22159717 ) | [CELA3A](https://www.genecards.org/Search/Keyword?queryString=CELA3A) |
| CELA3B | ECM Regulators | 28071719 | [28071719](https://www.ncbi.nlm.nih.gov/pubmed/28071719) | [CELA3B](https://www.genecards.org/Search/Keyword?queryString=CELA3B) |
| CEMIP | ECM Micro-enviornment associated | 23509262 | [23509262](https://www.ncbi.nlm.nih.gov/pubmed/23509262) | [CEMIP](https://www.genecards.org/Search/Keyword?queryString=CEMIP) |
| CFC1 | Secreted Factors | 22159717 | [22159717](https://www.ncbi.nlm.nih.gov/pubmed/22159717 ) | [CFC1](https://www.genecards.org/Search/Keyword?queryString=CFC1) |
| CFC1B | Secreted Factors | 22159717 | [22159717](https://www.ncbi.nlm.nih.gov/pubmed/22159717 ) | [CFC1B](https://www.genecards.org/Search/Keyword?queryString=CFC1B) |
| CFH | ECM Micro-enviornment associated | 20855886 | [20855886](https://www.ncbi.nlm.nih.gov/pubmed/20855886) | [CFH](https://www.genecards.org/Search/Keyword?queryString=CFH) |
| CGA | ECM Micro-enviornment associated | 20339004 | [20339004](https://www.ncbi.nlm.nih.gov/pubmed/20339004) | [CGA](https://www.genecards.org/Search/Keyword?queryString=CGA) |
| CHAD | Proteoglycans | 23419153 | [23419153](https://www.ncbi.nlm.nih.gov/pubmed/23419153) | [CHAD](https://www.genecards.org/Search/Keyword?queryString=CHAD) |
| CHADL | Proteoglycans | 22261194 | [22261194](https://www.ncbi.nlm.nih.gov/pubmed/22261194) | [CHADL](https://www.genecards.org/Search/Keyword?queryString=CHADL) |
| CHAT | ECM synthetic/degradation enzyme | 19429183 | [19429183](https://www.ncbi.nlm.nih.gov/pubmed/19429183) | [CHAT](https://www.genecards.org/Search/Keyword?queryString=CHAT) |
| CHGA | ECM Micro-enviornment associated | 8218367 | [8218367](https://www.ncbi.nlm.nih.gov/pubmed/8218367) | [CHGA](https://www.genecards.org/Search/Keyword?queryString=CHGA) |
| CHPF | ECM synthetic/degradation enzyme | 12761225 | [12761225](https://www.ncbi.nlm.nih.gov/pubmed/12761225) | [CHPF](https://www.genecards.org/Search/Keyword?queryString=CHPF) |
| CHPF2 | ECM synthetic/degradation enzyme | 18316376 | [18316376](https://www.ncbi.nlm.nih.gov/pubmed/18316376) | [CHPF2](https://www.genecards.org/Search/Keyword?queryString=CHPF2) |
| CHRD | Secreted Factors | 22159717 | [22159717](https://www.ncbi.nlm.nih.gov/pubmed/22159717 ) | [CHRD](https://www.genecards.org/Search/Keyword?queryString=CHRD) |
| CHRDL1 | Secreted Factors | 22159717 | [22159717](https://www.ncbi.nlm.nih.gov/pubmed/22159717 ) | [CHRDL1](https://www.genecards.org/Search/Keyword?queryString=CHRDL1) |
| CHRDL2 | Secreted Factors | 22159717 | [22159717](https://www.ncbi.nlm.nih.gov/pubmed/22159717 ) | [CHRDL2](https://www.genecards.org/Search/Keyword?queryString=CHRDL2) |
| CHST1 | ECM synthetic/degradation enzyme | 9405439 | [9405439](https://www.ncbi.nlm.nih.gov/pubmed/9405439) | [CHST1](https://www.genecards.org/Search/Keyword?queryString=CHST1) |
| CHST11 | ECM synthetic/degradation enzyme | 12847091 | [12847091](https://www.ncbi.nlm.nih.gov/pubmed/12847091) | [CHST11](https://www.genecards.org/Search/Keyword?queryString=CHST11) |
| CHST12 | ECM synthetic/degradation enzyme | 10781601 | [10781601](https://www.ncbi.nlm.nih.gov/pubmed/10781601 ) | [CHST12](https://www.genecards.org/Search/Keyword?queryString=CHST12) |
| CHST3 | ECM synthetic/degradation enzyme | 9714738 | [9714738](https://www.ncbi.nlm.nih.gov/pubmed/9714738) | [CHST3](https://www.genecards.org/Search/Keyword?queryString=CHST3) |
| CHST5 | ECM synthetic/degradation enzyme | 10491328 | [10491328](https://www.ncbi.nlm.nih.gov/pubmed/10491328) | [CHST5](https://www.genecards.org/Search/Keyword?queryString=CHST5) |
| CHST6 | ECM synthetic/degradation enzyme | 15013869) | [15013869)](https://www.ncbi.nlm.nih.gov/pubmed/15013869)) | [CHST6](https://www.genecards.org/Search/Keyword?queryString=CHST6) |
| CHSY1 | ECM synthetic/degradation enzyme | 12761550 | [12761550](https://www.ncbi.nlm.nih.gov/pubmed/12761550) | [CHSY1](https://www.genecards.org/Search/Keyword?queryString=CHSY1) |
| CHSY3 | ECM synthetic/degradation enzyme | 12907687 | [12907687](https://www.ncbi.nlm.nih.gov/pubmed/12907687) | [CHSY3](https://www.genecards.org/Search/Keyword?queryString=CHSY3) |
| CIB1 | ECM Micro-enviornment associated | 17882488 | [17882488](https://www.ncbi.nlm.nih.gov/pubmed/17882488) | [CIB1](https://www.genecards.org/Search/Keyword?queryString=CIB1) |
| CILP | ECM Glycoproteins | 15533759 | [15533759](https://www.ncbi.nlm.nih.gov/pubmed/15533759) | [CILP](https://www.genecards.org/Search/Keyword?queryString=CILP) |
| CILP2 | ECM Glycoproteins | 23376485 | [23376485](https://www.ncbi.nlm.nih.gov/pubmed/23376485) | [CILP2](https://www.genecards.org/Search/Keyword?queryString=CILP2) |
| CKM | ECM Micro-enviornment associated | 10896681 | [10896681](https://www.ncbi.nlm.nih.gov/pubmed/10896681) | [CKM](https://www.genecards.org/Search/Keyword?queryString=CKM) |
| CLC | ECM-affiliated Proteins | 2160562 | [2160562](https://www.ncbi.nlm.nih.gov/pubmed/2160562) | [CLC](https://www.genecards.org/Search/Keyword?queryString=CLC) |
| CLCF1 | Secreted Factors | 23355913 | [23355913](https://www.ncbi.nlm.nih.gov/pubmed/23355913) | [CLCF1](https://www.genecards.org/Search/Keyword?queryString=CLCF1) |
| CLEC10A | ECM-affiliated Proteins | 23601247 | [23601247](https://www.ncbi.nlm.nih.gov/pubmed/23601247) | [CLEC10A](https://www.genecards.org/Search/Keyword?queryString=CLEC10A) |
| CLEC11A | ECM-affiliated Proteins | 22159717 | [22159717](https://www.ncbi.nlm.nih.gov/pubmed/22159717 ) | [CLEC11A](https://www.genecards.org/Search/Keyword?queryString=CLEC11A) |
| CLEC12A | ECM-affiliated Proteins | 22159717 | [22159717](https://www.ncbi.nlm.nih.gov/pubmed/22159717 ) | [CLEC12A](https://www.genecards.org/Search/Keyword?queryString=CLEC12A) |
| CLEC12B | ECM-affiliated Proteins | 22159717 | [22159717](https://www.ncbi.nlm.nih.gov/pubmed/22159717 ) | [CLEC12B](https://www.genecards.org/Search/Keyword?queryString=CLEC12B) |
| CLEC14A | ECM-affiliated Proteins | 22159717 | [22159717](https://www.ncbi.nlm.nih.gov/pubmed/22159717 ) | [CLEC14A](https://www.genecards.org/Search/Keyword?queryString=CLEC14A) |
| CLEC17A | ECM-affiliated Proteins | 22159717 | [22159717](https://www.ncbi.nlm.nih.gov/pubmed/22159717 ) | [CLEC17A](https://www.genecards.org/Search/Keyword?queryString=CLEC17A) |
| CLEC18A | ECM-affiliated Proteins | 22159717 | [22159717](https://www.ncbi.nlm.nih.gov/pubmed/22159717 ) | [CLEC18A](https://www.genecards.org/Search/Keyword?queryString=CLEC18A) |
| CLEC18B | ECM-affiliated Proteins | 22159717 | [22159717](https://www.ncbi.nlm.nih.gov/pubmed/22159717 ) | [CLEC18B](https://www.genecards.org/Search/Keyword?queryString=CLEC18B) |
| CLEC18C | ECM-affiliated Proteins | 22159717 | [22159717](https://www.ncbi.nlm.nih.gov/pubmed/22159717 ) | [CLEC18C](https://www.genecards.org/Search/Keyword?queryString=CLEC18C) |
| CLEC19A | ECM-affiliated Proteins | 22159717 | [22159717](https://www.ncbi.nlm.nih.gov/pubmed/22159717 ) | [CLEC19A](https://www.genecards.org/Search/Keyword?queryString=CLEC19A) |
| CLEC1A | ECM-affiliated Proteins | 22159717 | [22159717](https://www.ncbi.nlm.nih.gov/pubmed/22159717 ) | [CLEC1A](https://www.genecards.org/Search/Keyword?queryString=CLEC1A) |
| CLEC1B | ECM-affiliated Proteins | 22159717 | [22159717](https://www.ncbi.nlm.nih.gov/pubmed/22159717 ) | [CLEC1B](https://www.genecards.org/Search/Keyword?queryString=CLEC1B) |
| CLEC2A | ECM-affiliated Proteins | 22159717 | [22159717](https://www.ncbi.nlm.nih.gov/pubmed/22159717 ) | [CLEC2A](https://www.genecards.org/Search/Keyword?queryString=CLEC2A) |
| CLEC2B | ECM-affiliated Proteins | 22159717 | [22159717](https://www.ncbi.nlm.nih.gov/pubmed/22159717 ) | [CLEC2B](https://www.genecards.org/Search/Keyword?queryString=CLEC2B) |
| CLEC2D | ECM-affiliated Proteins | 22159717 | [22159717](https://www.ncbi.nlm.nih.gov/pubmed/22159717 ) | [CLEC2D](https://www.genecards.org/Search/Keyword?queryString=CLEC2D) |
| CLEC2L | ECM-affiliated Proteins | 22159717 | [22159717](https://www.ncbi.nlm.nih.gov/pubmed/22159717 ) | [CLEC2L](https://www.genecards.org/Search/Keyword?queryString=CLEC2L) |
| CLEC3A | ECM-affiliated Proteins | 22159717 | [22159717](https://www.ncbi.nlm.nih.gov/pubmed/22159717 ) | [CLEC3A](https://www.genecards.org/Search/Keyword?queryString=CLEC3A) |
| CLEC3B | ECM-affiliated Proteins | 22159717 | [22159717](https://www.ncbi.nlm.nih.gov/pubmed/22159717 ) | [CLEC3B](https://www.genecards.org/Search/Keyword?queryString=CLEC3B) |
| CLEC4A | ECM-affiliated Proteins | 22159717 | [22159717](https://www.ncbi.nlm.nih.gov/pubmed/22159717 ) | [CLEC4A](https://www.genecards.org/Search/Keyword?queryString=CLEC4A) |
| CLEC4C | ECM-affiliated Proteins | 22159717 | [22159717](https://www.ncbi.nlm.nih.gov/pubmed/22159717 ) | [CLEC4C](https://www.genecards.org/Search/Keyword?queryString=CLEC4C) |
| CLEC4D | ECM-affiliated Proteins | 22159717 | [22159717](https://www.ncbi.nlm.nih.gov/pubmed/22159717 ) | [CLEC4D](https://www.genecards.org/Search/Keyword?queryString=CLEC4D) |
| CLEC4E | ECM-affiliated Proteins | 22159717 | [22159717](https://www.ncbi.nlm.nih.gov/pubmed/22159717 ) | [CLEC4E](https://www.genecards.org/Search/Keyword?queryString=CLEC4E) |
| CLEC4F | ECM-affiliated Proteins | 22159717 | [22159717](https://www.ncbi.nlm.nih.gov/pubmed/22159717 ) | [CLEC4F](https://www.genecards.org/Search/Keyword?queryString=CLEC4F) |
| CLEC4G | ECM-affiliated Proteins | 22159717 | [22159717](https://www.ncbi.nlm.nih.gov/pubmed/22159717 ) | [CLEC4G](https://www.genecards.org/Search/Keyword?queryString=CLEC4G) |
| CLEC4M | ECM-affiliated Proteins | 22159717 | [22159717](https://www.ncbi.nlm.nih.gov/pubmed/22159717 ) | [CLEC4M](https://www.genecards.org/Search/Keyword?queryString=CLEC4M) |
| CLEC5A | ECM-affiliated Proteins | 22159717 | [22159717](https://www.ncbi.nlm.nih.gov/pubmed/22159717 ) | [CLEC5A](https://www.genecards.org/Search/Keyword?queryString=CLEC5A) |
| CLEC6A | ECM-affiliated Proteins | 22159717 | [22159717](https://www.ncbi.nlm.nih.gov/pubmed/22159717 ) | [CLEC6A](https://www.genecards.org/Search/Keyword?queryString=CLEC6A) |
| CLEC7A | ECM-affiliated Proteins | 22159717 | [22159717](https://www.ncbi.nlm.nih.gov/pubmed/22159717 ) | [CLEC7A](https://www.genecards.org/Search/Keyword?queryString=CLEC7A) |
| CLEC9A | ECM-affiliated Proteins | 22159717 | [22159717](https://www.ncbi.nlm.nih.gov/pubmed/22159717 ) | [CLEC9A](https://www.genecards.org/Search/Keyword?queryString=CLEC9A) |
| CLGN | ECM Micro-enviornment associated | 24769397 | [24769397](https://www.ncbi.nlm.nih.gov/pubmed/24769397) | [CLGN](https://www.genecards.org/Search/Keyword?queryString=CLGN) |
| CLIC4 | ECM Micro-enviornment associated | 15322081 | [15322081](https://www.ncbi.nlm.nih.gov/pubmed/15322081) | [CLIC4](https://www.genecards.org/Search/Keyword?queryString=CLIC4) |
| CLPP | ECM Micro-enviornment associated | 18378094 | [18378094](https://www.ncbi.nlm.nih.gov/pubmed/18378094) | [CLPP](https://www.genecards.org/Search/Keyword?queryString=CLPP) |
| CMA1 | ECM Micro-enviornment associated | 18958543 | [18958543](https://www.ncbi.nlm.nih.gov/pubmed/18958543) | [CMA1](https://www.genecards.org/Search/Keyword?queryString=CMA1) |
| CNMD | ECM | 10487209 | [10487209](https://www.ncbi.nlm.nih.gov/pubmed/10487209) | [CNMD](https://www.genecards.org/Search/Keyword?queryString=CNMD) |
| CNTF | Secreted Factors | 2199462 | [2199462](https://www.ncbi.nlm.nih.gov/pubmed/2199462) | [CNTF](https://www.genecards.org/Search/Keyword?queryString=CNTF) |
| CNTN1 | Cytoskeleton | 12700241 | [12700241](https://www.ncbi.nlm.nih.gov/pubmed/12700241) | [CNTN1](https://www.genecards.org/Search/Keyword?queryString=CNTN1) |
| CNTN2 | Cytoskeleton | 7593629 | [7593629](https://www.ncbi.nlm.nih.gov/pubmed/7593629) | [CNTN2](https://www.genecards.org/Search/Keyword?queryString=CNTN2) |
| CNTNAP1 | ECM Micro-enviornment associated | 8524223 | [8524223](https://www.ncbi.nlm.nih.gov/pubmed/8524223) | [CNTNAP1](https://www.genecards.org/Search/Keyword?queryString=CNTNAP1) |
| COCH | ECM Glycoproteins | 23376485 | [23376485](https://www.ncbi.nlm.nih.gov/pubmed/23376485) | [COCH](https://www.genecards.org/Search/Keyword?queryString=COCH) |
| COL10A1 | Collagens | 2037056 | [2037056](https://www.ncbi.nlm.nih.gov/pubmed/2037056) | [COL10A1](https://www.genecards.org/Search/Keyword?queryString=COL10A1) |
| COL11A1 | Collagens | 3182841 | [3182841](https://www.ncbi.nlm.nih.gov/pubmed/3182841) | [COL11A1](https://www.genecards.org/Search/Keyword?queryString=COL11A1) |
| COL11A2 | Collagens | 7559422 | [7559422](https://www.ncbi.nlm.nih.gov/pubmed/7559422) | [COL11A2](https://www.genecards.org/Search/Keyword?queryString=COL11A2) |
| COL12A1 | Collagens | 9143499 | [9143499](https://www.ncbi.nlm.nih.gov/pubmed/9143499) | [COL12A1](https://www.genecards.org/Search/Keyword?queryString=COL12A1) |
| COL13A1 | Collagens | 26626625 | [26626625](https://www.ncbi.nlm.nih.gov/pubmed/26626625) | [COL13A1](https://www.genecards.org/Search/Keyword?queryString=COL13A1) |
| COL14A1 | Collagens | 9427527 | [9427527](https://www.ncbi.nlm.nih.gov/pubmed/9427527) | [COL14A1](https://www.genecards.org/Search/Keyword?queryString=COL14A1) |
| COL15A1 | Collagens | 1427836 | [1427836](https://www.ncbi.nlm.nih.gov/pubmed/1427836) | [COL15A1](https://www.genecards.org/Search/Keyword?queryString=COL15A1) |
| COL16A1 | Collagens | 1631157 | [1631157](https://www.ncbi.nlm.nih.gov/pubmed/1631157) | [COL16A1](https://www.genecards.org/Search/Keyword?queryString=COL16A1) |
| COL17A1 | Collagens | 7916703 | [7916703](https://www.ncbi.nlm.nih.gov/pubmed/7916703) | [COL17A1](https://www.genecards.org/Search/Keyword?queryString=COL17A1) |
| COL18A1 | Collagens | 8188291 | [8188291](https://www.ncbi.nlm.nih.gov/pubmed/8188291) | [COL18A1](https://www.genecards.org/Search/Keyword?queryString=COL18A1) |
| COL19A1 | Collagens | 7916703 | [7916703](https://www.ncbi.nlm.nih.gov/pubmed/7916703) | [COL19A1](https://www.genecards.org/Search/Keyword?queryString=COL19A1) |
| COL1A1 | Collagens | 2857713 | [2857713](https://www.ncbi.nlm.nih.gov/pubmed/2857713) | [COL1A1](https://www.genecards.org/Search/Keyword?queryString=COL1A1) |
| COL1A2 | Collagens | 3857213 | [3857213](https://www.ncbi.nlm.nih.gov/pubmed/3857213) | [COL1A2](https://www.genecards.org/Search/Keyword?queryString=COL1A2) |
| COL20A1 | Collagens | 10819331 | [10819331](https://www.ncbi.nlm.nih.gov/pubmed/10819331) | [COL20A1](https://www.genecards.org/Search/Keyword?queryString=COL20A1) |
| COL21A1 | Collagens | 11566190 | [11566190](https://www.ncbi.nlm.nih.gov/pubmed/11566190) | [COL21A1](https://www.genecards.org/Search/Keyword?queryString=COL21A1) |
| COL22A1 | Collagens | 15016833 | [15016833](https://www.ncbi.nlm.nih.gov/pubmed/15016833) | [COL22A1](https://www.genecards.org/Search/Keyword?queryString=COL22A1) |
| COL23A1 | Collagens | 12644459 | [12644459](https://www.ncbi.nlm.nih.gov/pubmed/12644459) | [COL23A1](https://www.genecards.org/Search/Keyword?queryString=COL23A1) |
| COL24A1 | Collagens | 16373341 | [16373341](https://www.ncbi.nlm.nih.gov/pubmed/16373341) | [COL24A1](https://www.genecards.org/Search/Keyword?queryString=COL24A1) |
| COL25A1 | Collagens | 11927537 | [11927537](https://www.ncbi.nlm.nih.gov/pubmed/11927537) | [COL25A1](https://www.genecards.org/Search/Keyword?queryString=COL25A1) |
| COL26A1 | Collagens | 12145293 | [12145293](https://www.ncbi.nlm.nih.gov/pubmed/12145293) | [COL26A1](https://www.genecards.org/Search/Keyword?queryString=COL26A1) |
| COL27A1 | Collagens | 12714037 | [12714037](https://www.ncbi.nlm.nih.gov/pubmed/12714037) | [COL27A1](https://www.genecards.org/Search/Keyword?queryString=COL27A1) |
| COL28A1 | Collagens | 16330543 | [16330543](https://www.ncbi.nlm.nih.gov/pubmed/16330543) | [COL28A1](https://www.genecards.org/Search/Keyword?queryString=COL28A1) |
| COL2A1 | Collagens | 1677770 | [1677770](https://www.ncbi.nlm.nih.gov/pubmed/1677770) | [COL2A1](https://www.genecards.org/Search/Keyword?queryString=COL2A1) |
| COL3A1 | Collagens | 2834369 | [2834369](https://www.ncbi.nlm.nih.gov/pubmed/2834369) | [COL3A1](https://www.genecards.org/Search/Keyword?queryString=COL3A1) |
| COL4A1 | Collagens | 1639407 | [1639407](https://www.ncbi.nlm.nih.gov/pubmed/1639407) | [COL4A1](https://www.genecards.org/Search/Keyword?queryString=COL4A1) |
| COL4A2 | Collagens | 1639407 | [1639407](https://www.ncbi.nlm.nih.gov/pubmed/1639407) | [COL4A2](https://www.genecards.org/Search/Keyword?queryString=COL4A2) |
| COL4A3 | Collagens | 1639407 | [1639407](https://www.ncbi.nlm.nih.gov/pubmed/1639407) | [COL4A3](https://www.genecards.org/Search/Keyword?queryString=COL4A3) |
| COL4A3BP | ECM Micro-enviornment associated | 18772132 | [18772132](https://www.ncbi.nlm.nih.gov/pubmed/18772132) | [COL4A3BP](https://www.genecards.org/Search/Keyword?queryString=COL4A3BP) |
| COL4A4 | Collagens | 1639407 | [1639407](https://www.ncbi.nlm.nih.gov/pubmed/1639407) | [COL4A4](https://www.genecards.org/Search/Keyword?queryString=COL4A4) |
| COL4A5 | Collagens | 1639407 | [1639407](https://www.ncbi.nlm.nih.gov/pubmed/1639407) | [COL4A5](https://www.genecards.org/Search/Keyword?queryString=COL4A5) |
| COL4A6 | Collagens | 8356449 | [8356449](https://www.ncbi.nlm.nih.gov/pubmed/8356449) | [COL4A6](https://www.genecards.org/Search/Keyword?queryString=COL4A6) |
| COL5A1 | Collagens | 26910848 | [26910848](https://www.ncbi.nlm.nih.gov/pubmed/26910848) | [COL5A1](https://www.genecards.org/Search/Keyword?queryString=COL5A1) |
| COL5A2 | Collagens | 26910848 | [26910848](https://www.ncbi.nlm.nih.gov/pubmed/26910848) | [COL5A2](https://www.genecards.org/Search/Keyword?queryString=COL5A2) |
| COL5A3 | Collagens | 26910848 | [26910848](https://www.ncbi.nlm.nih.gov/pubmed/26910848) | [COL5A3](https://www.genecards.org/Search/Keyword?queryString=COL5A3) |
| COL6A1 | Collagens | 23869615 | [23869615](https://www.ncbi.nlm.nih.gov/pubmed/23869615) | [COL6A1](https://www.genecards.org/Search/Keyword?queryString=COL6A1) |
| COL6A2 | Collagens | 23869615 | [23869615](https://www.ncbi.nlm.nih.gov/pubmed/23869615) | [COL6A2](https://www.genecards.org/Search/Keyword?queryString=COL6A2) |
| COL6A3 | Collagens | 23869615 | [23869615](https://www.ncbi.nlm.nih.gov/pubmed/23869615) | [COL6A3](https://www.genecards.org/Search/Keyword?queryString=COL6A3) |
| COL6A4P1 | ECM Micro-enviornment associated | 18276594 | [18276594](https://www.ncbi.nlm.nih.gov/pubmed/18276594) | [COL6A4P1](https://www.genecards.org/Search/Keyword?queryString=COL6A4P1) |
| COL6A4P2 | ECM Micro-enviornment associated | 19486942 | [19486942](https://www.ncbi.nlm.nih.gov/pubmed/19486942) | [COL6A4P2](https://www.genecards.org/Search/Keyword?queryString=COL6A4P2) |
| COL6A5 | Collagens | 23869615 | [23869615](https://www.ncbi.nlm.nih.gov/pubmed/23869615) | [COL6A5](https://www.genecards.org/Search/Keyword?queryString=COL6A5) |
| COL6A6 | Collagens | 23869615 | [23869615](https://www.ncbi.nlm.nih.gov/pubmed/23869615) | [COL6A6](https://www.genecards.org/Search/Keyword?queryString=COL6A6) |
| COL7A1 | Collagens | 1871109 | [1871109](https://www.ncbi.nlm.nih.gov/pubmed/1871109) | [COL7A1](https://www.genecards.org/Search/Keyword?queryString=COL7A1) |
| COL8A1 | Collagens | 2029894 | [2029894](https://www.ncbi.nlm.nih.gov/pubmed/2029894) | [COL8A1](https://www.genecards.org/Search/Keyword?queryString=COL8A1) |
| COL8A2 | Collagens | 2029894 | [2029894](https://www.ncbi.nlm.nih.gov/pubmed/2029894) | [COL8A2](https://www.genecards.org/Search/Keyword?queryString=COL8A2) |
| COL9A1 | Collagens | 8586434 | [8586434](https://www.ncbi.nlm.nih.gov/pubmed/8586434) | [COL9A1](https://www.genecards.org/Search/Keyword?queryString=COL9A1) |
| COL9A2 | Collagens | 8586434 | [8586434](https://www.ncbi.nlm.nih.gov/pubmed/8586434) | [COL9A2](https://www.genecards.org/Search/Keyword?queryString=COL9A2) |
| COL9A3 | Collagens | 8586434 | [8586434](https://www.ncbi.nlm.nih.gov/pubmed/8586434) | [COL9A3](https://www.genecards.org/Search/Keyword?queryString=COL9A3) |
| COLEC10 | ECM-affiliated Proteins | 22159717 | [22159717](https://www.ncbi.nlm.nih.gov/pubmed/22159717 ) | [COLEC10](https://www.genecards.org/Search/Keyword?queryString=COLEC10) |
| COLEC11 | ECM-affiliated Proteins | 22159717 | [22159717](https://www.ncbi.nlm.nih.gov/pubmed/22159717 ) | [COLEC11](https://www.genecards.org/Search/Keyword?queryString=COLEC11) |
| COLEC12 | ECM-affiliated Proteins | 22159717 | [22159717](https://www.ncbi.nlm.nih.gov/pubmed/22159717 ) | [COLEC12](https://www.genecards.org/Search/Keyword?queryString=COLEC12) |
| COLGALT1 | ECM synthetic/degradation enzyme | 19075007 | [19075007](https://www.ncbi.nlm.nih.gov/pubmed/19075007) | [COLGALT1](https://www.genecards.org/Search/Keyword?queryString=COLGALT1) |
| COLGALT2 | ECM synthetic/degradation enzyme | 19075007 | [19075007](https://www.ncbi.nlm.nih.gov/pubmed/19075007) | [COLGALT2](https://www.genecards.org/Search/Keyword?queryString=COLGALT2) |
| COLQ | ECM Glycoproteins | 10679527 | [10679527](https://www.ncbi.nlm.nih.gov/pubmed/10679527) | [COLQ](https://www.genecards.org/Search/Keyword?queryString=COLQ) |
| COMP | ECM Glycoproteins | 23419153 | [23419153](https://www.ncbi.nlm.nih.gov/pubmed/23419153) | [COMP](https://www.genecards.org/Search/Keyword?queryString=COMP) |
| CPAMD8 | ECM Regulators | 21278247 | [21278247](https://www.ncbi.nlm.nih.gov/pubmed/21278247) | [CPAMD8](https://www.genecards.org/Search/Keyword?queryString=CPAMD8) |
| CPN2 | ECM Regulators | 22159717 | [22159717](https://www.ncbi.nlm.nih.gov/pubmed/22159717 ) | [CPN2](https://www.genecards.org/Search/Keyword?queryString=CPN2) |
| CRELD1 | ECM Glycoproteins | 12137942 | [12137942](https://www.ncbi.nlm.nih.gov/pubmed/12137942) | [CRELD1](https://www.genecards.org/Search/Keyword?queryString=CRELD1) |
| CRELD2 | ECM Glycoproteins | 23956175 | [23956175](https://www.ncbi.nlm.nih.gov/pubmed/23956175) | [CRELD2](https://www.genecards.org/Search/Keyword?queryString=CRELD2) |
| CRHBP | Secreted Factors | 22159717 | [22159717](https://www.ncbi.nlm.nih.gov/pubmed/22159717 ) | [CRHBP](https://www.genecards.org/Search/Keyword?queryString=CRHBP) |
| CRIM1 | ECM Glycoproteins | 26821812 | [26821812](https://www.ncbi.nlm.nih.gov/pubmed/26821812) | [CRIM1](https://www.genecards.org/Search/Keyword?queryString=CRIM1) |
| CRISPLD1 | ECM Glycoproteins | 21937732 | [21937732](https://www.ncbi.nlm.nih.gov/pubmed/21937732) | [CRISPLD1](https://www.genecards.org/Search/Keyword?queryString=CRISPLD1) |
| CRISPLD2 | ECM Glycoproteins | 29270355 | [29270355](https://www.ncbi.nlm.nih.gov/pubmed/29270355) | [CRISPLD2](https://www.genecards.org/Search/Keyword?queryString=CRISPLD2) |
| CRLF1 | Secreted Factors | 22159717 | [22159717](https://www.ncbi.nlm.nih.gov/pubmed/22159717 ) | [CRLF1](https://www.genecards.org/Search/Keyword?queryString=CRLF1) |
| CRLF3 | Secreted Factors | 22159717 | [22159717](https://www.ncbi.nlm.nih.gov/pubmed/22159717 ) | [CRLF3](https://www.genecards.org/Search/Keyword?queryString=CRLF3) |
| CRNN | Secreted Factors | 22159717 | [22159717](https://www.ncbi.nlm.nih.gov/pubmed/22159717 ) | [CRNN](https://www.genecards.org/Search/Keyword?queryString=CRNN) |
| CSF1 | Secreted Factors | 22159717 | [22159717](https://www.ncbi.nlm.nih.gov/pubmed/22159717 ) | [CSF1](https://www.genecards.org/Search/Keyword?queryString=CSF1) |
| CSF2 | Secreted Factors | 22159717 | [22159717](https://www.ncbi.nlm.nih.gov/pubmed/22159717 ) | [CSF2](https://www.genecards.org/Search/Keyword?queryString=CSF2) |
| CSF3 | Secreted Factors | 22159717 | [22159717](https://www.ncbi.nlm.nih.gov/pubmed/22159717 ) | [CSF3](https://www.genecards.org/Search/Keyword?queryString=CSF3) |
| CSGALNACT1 | ECM synthetic/degradation enzyme | 17145758 | [17145758](https://www.ncbi.nlm.nih.gov/pubmed/17145758) | [CSGALNACT1](https://www.genecards.org/Search/Keyword?queryString=CSGALNACT1) |
| CSGALNACT2 | ECM synthetic/degradation enzyme | 12446672 | [12446672](https://www.ncbi.nlm.nih.gov/pubmed/12446672) | [CSGALNACT2](https://www.genecards.org/Search/Keyword?queryString=CSGALNACT2) |
| CSH1 | Secreted Factors | 22159717 | [22159717](https://www.ncbi.nlm.nih.gov/pubmed/22159717 ) | [CSH1](https://www.genecards.org/Search/Keyword?queryString=CSH1) |
| CSH2 | Secreted Factors | 22159717 | [22159717](https://www.ncbi.nlm.nih.gov/pubmed/22159717 ) | [CSH2](https://www.genecards.org/Search/Keyword?queryString=CSH2) |
| CSHL1 | Secreted Factors | 22159717 | [22159717](https://www.ncbi.nlm.nih.gov/pubmed/22159717 ) | [CSHL1](https://www.genecards.org/Search/Keyword?queryString=CSHL1) |
| CSPG4 | ECM-affiliated Proteins | 28536635 | [28536635](https://www.ncbi.nlm.nih.gov/pubmed/28536635) | [CSPG4](https://www.genecards.org/Search/Keyword?queryString=CSPG4) |
| CSPG5 | ECM-affiliated Proteins | 25852466 | [25852466](https://www.ncbi.nlm.nih.gov/pubmed/25852466) | [CSPG5](https://www.genecards.org/Search/Keyword?queryString=CSPG5) |
| CST1 | ECM Regulators | 24357805 | [24357805](https://www.ncbi.nlm.nih.gov/pubmed/24357805) | [CST1](https://www.genecards.org/Search/Keyword?queryString=CST1) |
| CST11 | ECM Regulators | 22159717 | [22159717](https://www.ncbi.nlm.nih.gov/pubmed/22159717 ) | [CST11](https://www.genecards.org/Search/Keyword?queryString=CST11) |
| CST2 | ECM Regulators | 24357805 | [24357805](https://www.ncbi.nlm.nih.gov/pubmed/24357805) | [CST2](https://www.genecards.org/Search/Keyword?queryString=CST2) |
| CST3 | ECM Regulators | 24357805 | [24357805](https://www.ncbi.nlm.nih.gov/pubmed/24357805) | [CST3](https://www.genecards.org/Search/Keyword?queryString=CST3) |
| CST4 | ECM Regulators | 24357805 | [24357805](https://www.ncbi.nlm.nih.gov/pubmed/24357805) | [CST4](https://www.genecards.org/Search/Keyword?queryString=CST4) |
| CST5 | ECM Regulators | 22159717 | [22159717](https://www.ncbi.nlm.nih.gov/pubmed/22159717 ) | [CST5](https://www.genecards.org/Search/Keyword?queryString=CST5) |
| CST6 | ECM Regulators | 24357805 | [24357805](https://www.ncbi.nlm.nih.gov/pubmed/24357805) | [CST6](https://www.genecards.org/Search/Keyword?queryString=CST6) |
| CST7 | ECM Regulators | 24357805 | [24357805](https://www.ncbi.nlm.nih.gov/pubmed/24357805) | [CST7](https://www.genecards.org/Search/Keyword?queryString=CST7) |
| CST8 | ECM Regulators | 22159717 | [22159717](https://www.ncbi.nlm.nih.gov/pubmed/22159717 ) | [CST8](https://www.genecards.org/Search/Keyword?queryString=CST8) |
| CST9 | ECM Regulators | 22159717 | [22159717](https://www.ncbi.nlm.nih.gov/pubmed/22159717 ) | [CST9](https://www.genecards.org/Search/Keyword?queryString=CST9) |
| CST9L | ECM Regulators | 22159717 | [22159717](https://www.ncbi.nlm.nih.gov/pubmed/22159717 ) | [CST9L](https://www.genecards.org/Search/Keyword?queryString=CST9L) |
| CSTA | ECM Regulators | 27988214 | [27988214](https://www.ncbi.nlm.nih.gov/pubmed/27988214) | [CSTA](https://www.genecards.org/Search/Keyword?queryString=CSTA) |
| CSTB | ECM Regulators | 28071719 | [28071719](https://www.ncbi.nlm.nih.gov/pubmed/ 28071719) | [CSTB](https://www.genecards.org/Search/Keyword?queryString=CSTB) |
| CSTL1 | ECM Regulators | 22159717 | [22159717](https://www.ncbi.nlm.nih.gov/pubmed/22159717 ) | [CSTL1](https://www.genecards.org/Search/Keyword?queryString=CSTL1) |
| CTF1 | Secreted Factors | 27737648 | [27737648](https://www.ncbi.nlm.nih.gov/pubmed/27737648) | [CTF1](https://www.genecards.org/Search/Keyword?queryString=CTF1) |
| CTGF | ECM Glycoproteins | 19450452 | [19450452](https://www.ncbi.nlm.nih.gov/pubmed/19450452) | [CTGF](https://www.genecards.org/Search/Keyword?queryString=CTGF) |
| CTHRC1 | ECM Glycoproteins | 23658133 | [23658133](https://www.ncbi.nlm.nih.gov/pubmed/23658133) | [CTHRC1](https://www.genecards.org/Search/Keyword?queryString=CTHRC1) |
| CTNNB1 | ECM Micro-enviornment associated | 20372837 | [20372837](https://www.ncbi.nlm.nih.gov/pubmed/20372837) | [CTNNB1](https://www.genecards.org/Search/Keyword?queryString=CTNNB1) |
| CTSA | ECM Regulators | 27432266 | [27432266](https://www.ncbi.nlm.nih.gov/pubmed/27432266) | [CTSA](https://www.genecards.org/Search/Keyword?queryString=CTSA) |
| CTSB | ECM Regulators | 23844232 | [23844232](https://www.ncbi.nlm.nih.gov/pubmed/23844232) | [CTSB](https://www.genecards.org/Search/Keyword?queryString=CTSB) |
| CTSC | ECM Regulators | 24065739 | [24065739](https://www.ncbi.nlm.nih.gov/pubmed/24065739) | [CTSC](https://www.genecards.org/Search/Keyword?queryString=CTSC) |
| CTSD | ECM Regulators | 26831567 | [26831567](https://www.ncbi.nlm.nih.gov/pubmed/26831567) | [CTSD](https://www.genecards.org/Search/Keyword?queryString=CTSD) |
| CTSE | ECM Regulators | 23844232 | [23844232](https://www.ncbi.nlm.nih.gov/pubmed/23844232) | [CTSE](https://www.genecards.org/Search/Keyword?queryString=CTSE) |
| CTSF | ECM Regulators | 19077055 | [19077055](https://www.ncbi.nlm.nih.gov/pubmed/19077055) | [CTSF](https://www.genecards.org/Search/Keyword?queryString=CTSF) |
| CTSG | ECM Regulators | 28411251 | [28411251](https://www.ncbi.nlm.nih.gov/pubmed/28411251) | [CTSG](https://www.genecards.org/Search/Keyword?queryString=CTSG) |
| CTSH | ECM Regulators | 19077055 | [19077055](https://www.ncbi.nlm.nih.gov/pubmed/19077055) | [CTSH](https://www.genecards.org/Search/Keyword?queryString=CTSH) |
| CTSK | ECM Regulators | 28098926 | [28098926](https://www.ncbi.nlm.nih.gov/pubmed/28098926) | [CTSK](https://www.genecards.org/Search/Keyword?queryString=CTSK) |
| CTSL | ECM Regulators | 26299995 | [26299995](https://www.ncbi.nlm.nih.gov/pubmed/26299995) | [CTSL](https://www.genecards.org/Search/Keyword?queryString=CTSL) |
| CTSO | ECM Regulators | 22159717 | [22159717](https://www.ncbi.nlm.nih.gov/pubmed/22159717 ) | [CTSO](https://www.genecards.org/Search/Keyword?queryString=CTSO) |
| CTSS | ECM Regulators | 16825321 | [16825321](https://www.ncbi.nlm.nih.gov/pubmed/16825321) | [CTSS](https://www.genecards.org/Search/Keyword?queryString=CTSS) |
| CTSV | ECM Regulators | 22159717 | [22159717](https://www.ncbi.nlm.nih.gov/pubmed/22159717 ) | [CTSV](https://www.genecards.org/Search/Keyword?queryString=CTSV) |
| CTSW | ECM Regulators | 22159717 | [22159717](https://www.ncbi.nlm.nih.gov/pubmed/22159717 ) | [CTSW](https://www.genecards.org/Search/Keyword?queryString=CTSW) |
| CTSZ | ECM Regulators | 22159717 | [22159717](https://www.ncbi.nlm.nih.gov/pubmed/22159717 ) | [CTSZ](https://www.genecards.org/Search/Keyword?queryString=CTSZ) |
| CTTN | ECM Micro-enviornment associated | 9054437 | [9054437](https://www.ncbi.nlm.nih.gov/pubmed/ 9054437) | [CTTN](https://www.genecards.org/Search/Keyword?queryString=CTTN) |
| CX3CL1 | Secreted Factors | 18367676 | [18367676](https://www.ncbi.nlm.nih.gov/pubmed/18367676) | [CX3CL1](https://www.genecards.org/Search/Keyword?queryString=CX3CL1) |
| CXCL1 | Secreted Factors | 17702850 | [17702850](https://www.ncbi.nlm.nih.gov/pubmed/17702850) | [CXCL1](https://www.genecards.org/Search/Keyword?queryString=CXCL1) |
| CXCL10 | Secreted Factors | 23630573 | [23630573](https://www.ncbi.nlm.nih.gov/pubmed/23630573) | [CXCL10](https://www.genecards.org/Search/Keyword?queryString=CXCL10) |
| CXCL11 | Secreted Factors | 24205388 | [24205388](https://www.ncbi.nlm.nih.gov/pubmed/24205388) | [CXCL11](https://www.genecards.org/Search/Keyword?queryString=CXCL11) |
| CXCL12 | Secreted Factors | 22253872 | [22253872](https://www.ncbi.nlm.nih.gov/pubmed/22253872) | [CXCL12](https://www.genecards.org/Search/Keyword?queryString=CXCL12) |
| CXCL13 | Secreted Factors | 28881808 | [28881808](https://www.ncbi.nlm.nih.gov/pubmed/ 28881808) | [CXCL13](https://www.genecards.org/Search/Keyword?queryString=CXCL13) |
| CXCL14 | Secreted Factors | 27115465 | [27115465](https://www.ncbi.nlm.nih.gov/pubmed/ 27115465) | [CXCL14](https://www.genecards.org/Search/Keyword?queryString=CXCL14) |
| CXCL2 | Secreted Factors | 17702850 | [17702850](https://www.ncbi.nlm.nih.gov/pubmed/17702850) | [CXCL2](https://www.genecards.org/Search/Keyword?queryString=CXCL2) |
| CXCL3 | Secreted Factors | 16263140 | [16263140](https://www.ncbi.nlm.nih.gov/pubmed/16263140) | [CXCL3](https://www.genecards.org/Search/Keyword?queryString=CXCL3) |
| CXCL5 | Secreted Factors | 16263140 | [16263140](https://www.ncbi.nlm.nih.gov/pubmed/16263140) | [CXCL5](https://www.genecards.org/Search/Keyword?queryString=CXCL5) |
| CXCL6 | Secreted Factors | 16263140 | [16263140](https://www.ncbi.nlm.nih.gov/pubmed/16263140) | [CXCL6](https://www.genecards.org/Search/Keyword?queryString=CXCL6) |
| CXCL8 | Secreted Factors | 20124102 | [20124102](https://www.ncbi.nlm.nih.gov/pubmed/20124102) | [CXCL8](https://www.genecards.org/Search/Keyword?queryString=CXCL8) |
| CXCL9 | Secreted Factors | 27490802 | [27490802](https://www.ncbi.nlm.nih.gov/pubmed/27490802) | [CXCL9](https://www.genecards.org/Search/Keyword?queryString=CXCL9) |
| CXCR4 | ECM Micro-enviornment associated | 25808184 | [25808184](https://www.ncbi.nlm.nih.gov/pubmed/25808184) | [CXCR4](https://www.genecards.org/Search/Keyword?queryString=CXCR4) |
| CYR61 | ECM Glycoproteins | 9184077 | [9184077](https://www.ncbi.nlm.nih.gov/pubmed/9184077) | [CYR61](https://www.genecards.org/Search/Keyword?queryString=CYR61) |
| CYTL1 | ECM Micro-enviornment associated | 15292256 | [15292256](https://www.ncbi.nlm.nih.gov/pubmed/15292256) | [CYTL1](https://www.genecards.org/Search/Keyword?queryString=CYTL1) |
| DAG1 | ECM Micro-enviornment associated | 18341635 | [18341635](https://www.ncbi.nlm.nih.gov/pubmed/18341635) | [DAG1](https://www.genecards.org/Search/Keyword?queryString=DAG1) |
| DCHS1 | ECM Micro-enviornment associated | 24056717 | [24056717](https://www.ncbi.nlm.nih.gov/pubmed/24056717) | [DCHS1](https://www.genecards.org/Search/Keyword?queryString=DCHS1) |
| DCN | Proteoglycans | 23419153 | [23419153](https://www.ncbi.nlm.nih.gov/pubmed/23419153) | [DCN](https://www.genecards.org/Search/Keyword?queryString=DCN) |
| DCSTAMP | ECM Micro-enviornment associated | 11114299 | [11114299](https://www.ncbi.nlm.nih.gov/pubmed/11114299) | [DCSTAMP](https://www.genecards.org/Search/Keyword?queryString=DCSTAMP) |
| DDR2 | ECM Micro-enviornment associated | 18201965 | [18201965](https://www.ncbi.nlm.nih.gov/pubmed/18201965) | [DDR2](https://www.genecards.org/Search/Keyword?queryString=DDR2) |
| DDX26B | ECM Glycoproteins | 23956427 | [23956427](https://www.ncbi.nlm.nih.gov/pubmed/23956427) | [DDX26B](https://www.genecards.org/Search/Keyword?queryString=DDX26B) |
| DDX5 | ECM Micro-enviornment associated | 23979707 | [23979707](https://www.ncbi.nlm.nih.gov/pubmed/23979707) | [DDX5](https://www.genecards.org/Search/Keyword?queryString=DDX5) |
| DEFB1 | ECM Micro-enviornment associated | 17996844 | [17996844](https://www.ncbi.nlm.nih.gov/pubmed/17996844) | [DEFB1](https://www.genecards.org/Search/Keyword?queryString=DEFB1) |
| DEFB103B | ECM Micro-enviornment associated | 26539799 | [26539799](https://www.ncbi.nlm.nih.gov/pubmed/26539799) | [DEFB103B](https://www.genecards.org/Search/Keyword?queryString=DEFB103B) |
| DEFB4A | ECM Micro-enviornment associated | 26539799 | [26539799](https://www.ncbi.nlm.nih.gov/pubmed/26539799) | [DEFB4A](https://www.genecards.org/Search/Keyword?queryString=DEFB4A) |
| DHH | Secreted Factors | 22159717 | [22159717](https://www.ncbi.nlm.nih.gov/pubmed/22159717 ) | [DHH](https://www.genecards.org/Search/Keyword?queryString=DHH) |
| DMBT1 | ECM Glycoproteins | 22053071 | [22053071](https://www.ncbi.nlm.nih.gov/pubmed/22053071) | [DMBT1](https://www.genecards.org/Search/Keyword?queryString=DMBT1) |
| DMP1 | ECM Glycoproteins | 12646701 | [12646701](https://www.ncbi.nlm.nih.gov/pubmed/12646701) | [DMP1](https://www.genecards.org/Search/Keyword?queryString=DMP1) |
| DNM3 | ECM Micro-enviornment associated | 12631724 | [12631724](https://www.ncbi.nlm.nih.gov/pubmed/12631724) | [DNM3](https://www.genecards.org/Search/Keyword?queryString=DNM3) |
| DPH3 | ECM synthetic/degradation enzyme | 23185508 | [23185508](https://www.ncbi.nlm.nih.gov/pubmed/23185508) | [DPH3](https://www.genecards.org/Search/Keyword?queryString=DPH3) |
| DPP4 | ECM synthetic/degradation enzyme | 1362072 | [1362072](https://www.ncbi.nlm.nih.gov/pubmed/1362072) | [DPP4](https://www.genecards.org/Search/Keyword?queryString=DPP4) |
| DPT | ECM Glycoproteins | 23419153 | [23419153](https://www.ncbi.nlm.nih.gov/pubmed/23419153) | [DPT](https://www.genecards.org/Search/Keyword?queryString=DPT) |
| DROSHA | ECM Micro-enviornment associated | 8526932 | [8526932](https://www.ncbi.nlm.nih.gov/pubmed/8526932) | [DROSHA](https://www.genecards.org/Search/Keyword?queryString=DROSHA) |
| DSE | ECM synthetic/degradation enzyme | 16505484 | [16505484](https://www.ncbi.nlm.nih.gov/pubmed/16505484) | [DSE](https://www.genecards.org/Search/Keyword?queryString=DSE) |
| DSPP | ECM Glycoproteins | 12646701 | [12646701](https://www.ncbi.nlm.nih.gov/pubmed/12646701) | [DSPP](https://www.genecards.org/Search/Keyword?queryString=DSPP) |
| DST | ECM Micro-enviornment associated | 7818282 | [7818282](https://www.ncbi.nlm.nih.gov/pubmed/7818282) | [DST](https://www.genecards.org/Search/Keyword?queryString=DST) |
| EBI3 | Secreted Factors | 26355156 | [26355156](https://www.ncbi.nlm.nih.gov/pubmed/26355156) | [EBI3](https://www.genecards.org/Search/Keyword?queryString=EBI3) |
| ECM1 | ECM Glycoproteins | 18200062 | [18200062](https://www.ncbi.nlm.nih.gov/pubmed/18200062) | [ECM1](https://www.genecards.org/Search/Keyword?queryString=ECM1) |
| ECM2 | ECM Glycoproteins | 9345023 | [9345023](https://www.ncbi.nlm.nih.gov/pubmed/9345023) | [ECM2](https://www.genecards.org/Search/Keyword?queryString=ECM2) |
| EDA | Secreted Factors | 23419153 | [23419153](https://www.ncbi.nlm.nih.gov/pubmed/23419153) | [EDA](https://www.genecards.org/Search/Keyword?queryString=EDA) |
| EDIL3 | ECM Glycoproteins | 29182679 | [29182679](https://www.ncbi.nlm.nih.gov/pubmed/29182679) | [EDIL3](https://www.genecards.org/Search/Keyword?queryString=EDIL3) |
| EDN1 | ECM Micro-enviornment associated | 24756349 | [24756349](https://www.ncbi.nlm.nih.gov/pubmed/24756349) | [EDN1](https://www.genecards.org/Search/Keyword?queryString=EDN1) |
| EDNRA | ECM Micro-enviornment associated | 9918760 | [9918760](https://www.ncbi.nlm.nih.gov/pubmed/9918760) | [EDNRA](https://www.genecards.org/Search/Keyword?queryString=EDNRA) |
| EFEMP1 | ECM Glycoproteins | 23419153 | [23419153](https://www.ncbi.nlm.nih.gov/pubmed/23419153) | [EFEMP1](https://www.genecards.org/Search/Keyword?queryString=EFEMP1) |
| EFEMP2 | ECM Glycoproteins | 23419153 | [23419153](https://www.ncbi.nlm.nih.gov/pubmed/23419153) | [EFEMP2](https://www.genecards.org/Search/Keyword?queryString=EFEMP2) |
| EGF | Secreted Factors | 12297050 | [12297050](https://www.ncbi.nlm.nih.gov/pubmed/12297050) | [EGF](https://www.genecards.org/Search/Keyword?queryString=EGF) |
| EGFL6 | Secreted Factors | 20574786 | [20574786](https://www.ncbi.nlm.nih.gov/pubmed/20574786) | [EGFL6](https://www.genecards.org/Search/Keyword?queryString=EGFL6) |
| EGFL7 | Secreted Factors | 22160377 | [22160377](https://www.ncbi.nlm.nih.gov/pubmed/22160377) | [EGFL7](https://www.genecards.org/Search/Keyword?queryString=EGFL7) |
| EGFL8 | Secreted Factors | 22160377 | [22160377](https://www.ncbi.nlm.nih.gov/pubmed/22160377) | [EGFL8](https://www.genecards.org/Search/Keyword?queryString=EGFL8) |
| EGFLAM | ECM Glycoproteins | 21937732 | [21937732](https://www.ncbi.nlm.nih.gov/pubmed/21937732) | [EGFLAM](https://www.genecards.org/Search/Keyword?queryString=EGFLAM) |
| EGFR | ECM Micro-enviornment associated | 21423176 | [21423176](https://www.ncbi.nlm.nih.gov/pubmed/21423176) | [EGFR](https://www.genecards.org/Search/Keyword?queryString=EGFR) |
| EGLN1 | ECM Regulators | 24618895 | [24618895](https://www.ncbi.nlm.nih.gov/pubmed/24618895) | [EGLN1](https://www.genecards.org/Search/Keyword?queryString=EGLN1) |
| EGLN2 | ECM Regulators | 22159717 | [22159717](https://www.ncbi.nlm.nih.gov/pubmed/22159717 ) | [EGLN2](https://www.genecards.org/Search/Keyword?queryString=EGLN2) |
| EGLN3 | ECM Regulators | 22159717 | [22159717](https://www.ncbi.nlm.nih.gov/pubmed/22159717 ) | [EGLN3](https://www.genecards.org/Search/Keyword?queryString=EGLN3) |
| EIF4B | ECM Micro-enviornment associated | 25468996 | [25468996](https://www.ncbi.nlm.nih.gov/pubmed/25468996) | [EIF4B](https://www.genecards.org/Search/Keyword?queryString=EIF4B) |
| ELANE | ECM Regulators | 22159717 | [22159717](https://www.ncbi.nlm.nih.gov/pubmed/22159717 ) | [ELANE](https://www.genecards.org/Search/Keyword?queryString=ELANE) |
| ELFN1 | ECM-affiliated Proteins | 28615711 | [28615711](https://www.ncbi.nlm.nih.gov/pubmed/28615711) | [ELFN1](https://www.genecards.org/Search/Keyword?queryString=ELFN1) |
| ELFN2 | ECM-affiliated Proteins | 28255461 | [28255461](https://www.ncbi.nlm.nih.gov/pubmed/28255461) | [ELFN2](https://www.genecards.org/Search/Keyword?queryString=ELFN2) |
| ELK1 | ECM Micro-enviornment associated | 17724016 | [17724016](https://www.ncbi.nlm.nih.gov/pubmed/17724016) | [ELK1](https://www.genecards.org/Search/Keyword?queryString=ELK1) |
| ELN | ECM Glycoproteins | 23419153 | [23419153](https://www.ncbi.nlm.nih.gov/pubmed/23419153) | [ELN](https://www.genecards.org/Search/Keyword?queryString=ELN) |
| ELSPBP1 | ECM Glycoproteins | 27784933 | [27784933](https://www.ncbi.nlm.nih.gov/pubmed/27784933) | [ELSPBP1](https://www.genecards.org/Search/Keyword?queryString=ELSPBP1) |
| EMCN | ECM-affiliated Proteins | 29215001 | [29215001](https://www.ncbi.nlm.nih.gov/pubmed/29215001) | [EMCN](https://www.genecards.org/Search/Keyword?queryString=EMCN) |
| EMID1 | ECM Glycoproteins | 23419153 | [23419153](https://www.ncbi.nlm.nih.gov/pubmed/23419153) | [EMID1](https://www.genecards.org/Search/Keyword?queryString=EMID1) |
| EMILIN1 | ECM Glycoproteins | 23419153 | [23419153](https://www.ncbi.nlm.nih.gov/pubmed/23419153) | [EMILIN1](https://www.genecards.org/Search/Keyword?queryString=EMILIN1) |
| EMILIN2 | ECM Glycoproteins | 23419153 | [23419153](https://www.ncbi.nlm.nih.gov/pubmed/23419153) | [EMILIN2](https://www.genecards.org/Search/Keyword?queryString=EMILIN2) |
| EMILIN3 | ECM Glycoproteins | 23419153 | [23419153](https://www.ncbi.nlm.nih.gov/pubmed/23419153) | [EMILIN3](https://www.genecards.org/Search/Keyword?queryString=EMILIN3) |
| ENPP2 | ECM synthetic/degradation enzyme | 23150666 | [23150666](https://www.ncbi.nlm.nih.gov/pubmed/23150666) | [ENPP2](https://www.genecards.org/Search/Keyword?queryString=ENPP2) |
| EPGN | Secreted Factors | 22159717 | [22159717](https://www.ncbi.nlm.nih.gov/pubmed/22159717 ) | [EPGN](https://www.genecards.org/Search/Keyword?queryString=EPGN) |
| EPO | Secreted Factors | 24685986 | [24685986](https://www.ncbi.nlm.nih.gov/pubmed/24685986) | [EPO](https://www.genecards.org/Search/Keyword?queryString=EPO) |
| EPS15 | ECM Micro-enviornment associated | 17623661 | [17623661](https://www.ncbi.nlm.nih.gov/pubmed/17623661) | [EPS15](https://www.genecards.org/Search/Keyword?queryString=EPS15) |
| EPYC | Proteoglycans | 23419153 | [23419153](https://www.ncbi.nlm.nih.gov/pubmed/23419153) | [EPYC](https://www.genecards.org/Search/Keyword?queryString=EPYC) |
| ERBB2 | ECM Micro-enviornment associated | 25681438 | [25681438](https://www.ncbi.nlm.nih.gov/pubmed/25681438) | [ERBB2](https://www.genecards.org/Search/Keyword?queryString=ERBB2) |
| ERBB3 | ECM Micro-enviornment associated | 7556068 | [7556068](https://www.ncbi.nlm.nih.gov/pubmed/7556068) | [ERBB3](https://www.genecards.org/Search/Keyword?queryString=ERBB3) |
| ERBB4 | ECM Micro-enviornment associated | 15579589 | [15579589](https://www.ncbi.nlm.nih.gov/pubmed/15579589) | [ERBB4](https://www.genecards.org/Search/Keyword?queryString=ERBB4) |
| EREG | Secreted Factors | 21269401 | [21269401](https://www.ncbi.nlm.nih.gov/pubmed/21269401) | [EREG](https://www.genecards.org/Search/Keyword?queryString=EREG) |
| ESM1 | Proteoglycans | 20705756 | [20705756](https://www.ncbi.nlm.nih.gov/pubmed/20705756) | [ESM1](https://www.genecards.org/Search/Keyword?queryString=ESM1) |
| ESR1 | ECM Micro-enviornment associated | 20398004 | [20398004](https://www.ncbi.nlm.nih.gov/pubmed/20398004) | [ESR1](https://www.genecards.org/Search/Keyword?queryString=ESR1) |
| EXT1 | ECM synthetic/degradation enzyme | 11391482 | [11391482](https://www.ncbi.nlm.nih.gov/pubmed/11391482) | [EXT1](https://www.genecards.org/Search/Keyword?queryString=EXT1) |
| EXT2 | ECM synthetic/degradation enzyme | 17226760 | [17226760](https://www.ncbi.nlm.nih.gov/pubmed/17226760 ) | [EXT2](https://www.genecards.org/Search/Keyword?queryString=EXT2) |
| EXTL1 | ECM synthetic/degradation enzyme | 17237233 | [17237233](https://www.ncbi.nlm.nih.gov/pubmed/17237233) | [EXTL1](https://www.genecards.org/Search/Keyword?queryString=EXTL1) |
| EXTL2 | ECM synthetic/degradation enzyme | 15831490 | [15831490](https://www.ncbi.nlm.nih.gov/pubmed/15831490) | [EXTL2](https://www.genecards.org/Search/Keyword?queryString=EXTL2) |
| EXTL3 | ECM synthetic/degradation enzyme | 17761672 | [17761672](https://www.ncbi.nlm.nih.gov/pubmed/17761672) | [EXTL3](https://www.genecards.org/Search/Keyword?queryString=EXTL3) |
| EYS | ECM Glycoproteins | 26163349 | [26163349](https://www.ncbi.nlm.nih.gov/pubmed/26163349) | [EYS](https://www.genecards.org/Search/Keyword?queryString=EYS) |
| EZR | ECM Micro-enviornment associated | 10366421 | [10366421](https://www.ncbi.nlm.nih.gov/pubmed/10366421) | [EZR](https://www.genecards.org/Search/Keyword?queryString=EZR) |
| F10 | ECM Regulators | 21252089 | [21252089](https://www.ncbi.nlm.nih.gov/pubmed/21252089) | [F10](https://www.genecards.org/Search/Keyword?queryString=F10) |
| F12 | ECM Regulators | 25589788 | [25589788](https://www.ncbi.nlm.nih.gov/pubmed/25589788) | [F12](https://www.genecards.org/Search/Keyword?queryString=F12) |
| F13A1 | ECM Regulators | 7906563 | [7906563](https://www.ncbi.nlm.nih.gov/pubmed/7906563) | [F13A1](https://www.genecards.org/Search/Keyword?queryString=F13A1) |
| F13B | ECM Regulators | 22278598 | [22278598](https://www.ncbi.nlm.nih.gov/pubmed/22278598) | [F13B](https://www.genecards.org/Search/Keyword?queryString=F13B) |
| F2 | ECM Regulators | 19415820 | [19415820](https://www.ncbi.nlm.nih.gov/pubmed/19415820) | [F2](https://www.genecards.org/Search/Keyword?queryString=F2) |
| F2R | ECM Micro-enviornment associated | 20571025 | [20571025](https://www.ncbi.nlm.nih.gov/pubmed/20571025) | [F2R](https://www.genecards.org/Search/Keyword?queryString=F2R) |
| F5 | ECM Micro-enviornment associated | 18978274 | [18978274](https://www.ncbi.nlm.nih.gov/pubmed/18978274) | [F5](https://www.genecards.org/Search/Keyword?queryString=F5) |
| F7 | ECM Regulators | 19415820 | [19415820](https://www.ncbi.nlm.nih.gov/pubmed/19415820) | [F7](https://www.genecards.org/Search/Keyword?queryString=F7) |
| F9 | ECM Regulators | 19415820 | [19415820](https://www.ncbi.nlm.nih.gov/pubmed/19415820) | [F9](https://www.genecards.org/Search/Keyword?queryString=F9) |
| FAM20A | ECM Regulators | 26091039 | [26091039](https://www.ncbi.nlm.nih.gov/pubmed/26091039) | [FAM20A](https://www.genecards.org/Search/Keyword?queryString=FAM20A) |
| FAM20B | ECM Regulators | 26091039 | [26091039](https://www.ncbi.nlm.nih.gov/pubmed/26091039) | [FAM20B](https://www.genecards.org/Search/Keyword?queryString=FAM20B) |
| FAM20C | ECM Regulators | 26091039 | [26091039](https://www.ncbi.nlm.nih.gov/pubmed/26091039) | [FAM20C](https://www.genecards.org/Search/Keyword?queryString=FAM20C) |
| FAP | ECM Micro-enviornment associated | 21423176 | [21423176](https://www.ncbi.nlm.nih.gov/pubmed/21423176) | [FAP](https://www.genecards.org/Search/Keyword?queryString=FAP) |
| FAS | ECM Micro-enviornment associated | 11212252 | [11212252](https://www.ncbi.nlm.nih.gov/pubmed/11212252) | [FAS](https://www.genecards.org/Search/Keyword?queryString=FAS) |
| FASLG | Secreted Factors | 16687653 | [16687653](https://www.ncbi.nlm.nih.gov/pubmed/16687653) | [FASLG](https://www.genecards.org/Search/Keyword?queryString=FASLG) |
| FBLN1 | ECM Glycoproteins | 23419153 | [23419153](https://www.ncbi.nlm.nih.gov/pubmed/23419153) | [FBLN1](https://www.genecards.org/Search/Keyword?queryString=FBLN1) |
| FBLN2 | ECM Glycoproteins | 23419153 | [23419153](https://www.ncbi.nlm.nih.gov/pubmed/23419153) | [FBLN2](https://www.genecards.org/Search/Keyword?queryString=FBLN2) |
| FBLN5 | ECM Glycoproteins | 23419153 | [23419153](https://www.ncbi.nlm.nih.gov/pubmed/23419153) | [FBLN5](https://www.genecards.org/Search/Keyword?queryString=FBLN5) |
| FBLN7 | ECM Glycoproteins | 23419153 | [23419153](https://www.ncbi.nlm.nih.gov/pubmed/23419153) | [FBLN7](https://www.genecards.org/Search/Keyword?queryString=FBLN7) |
| FBN1 | ECM Glycoproteins | 23419153 | [23419153](https://www.ncbi.nlm.nih.gov/pubmed/23419153) | [FBN1](https://www.genecards.org/Search/Keyword?queryString=FBN1) |
| FBN2 | ECM Glycoproteins | 23419153 | [23419153](https://www.ncbi.nlm.nih.gov/pubmed/23419153) | [FBN2](https://www.genecards.org/Search/Keyword?queryString=FBN2) |
| FBN3 | ECM Glycoproteins | 23419153 | [23419153](https://www.ncbi.nlm.nih.gov/pubmed/23419153) | [FBN3](https://www.genecards.org/Search/Keyword?queryString=FBN3) |
| FCN1 | ECM-affiliated Proteins | 22159717 | [22159717](https://www.ncbi.nlm.nih.gov/pubmed/22159717 ) | [FCN1](https://www.genecards.org/Search/Keyword?queryString=FCN1) |
| FCN2 | ECM-affiliated Proteins | 22159717 | [22159717](https://www.ncbi.nlm.nih.gov/pubmed/22159717 ) | [FCN2](https://www.genecards.org/Search/Keyword?queryString=FCN2) |
| FCN3 | ECM-affiliated Proteins | 29170406 | [29170406](https://www.ncbi.nlm.nih.gov/pubmed/29170406) | [FCN3](https://www.genecards.org/Search/Keyword?queryString=FCN3) |
| FGA | ECM Glycoproteins | 26135677 | [26135677](https://www.ncbi.nlm.nih.gov/pubmed/26135677) | [FGA](https://www.genecards.org/Search/Keyword?queryString=FGA) |
| FGB | ECM Glycoproteins | 27988214 | [27988214](https://www.ncbi.nlm.nih.gov/pubmed/27988214) | [FGB](https://www.genecards.org/Search/Keyword?queryString=FGB) |
| FGF1 | Secreted Factors | 27858202 | [27858202](https://www.ncbi.nlm.nih.gov/pubmed/27858202) | [FGF1](https://www.genecards.org/Search/Keyword?queryString=FGF1) |
| FGF10 | Secreted Factors | 19755711 | [19755711](https://www.ncbi.nlm.nih.gov/pubmed/19755711) | [FGF10](https://www.genecards.org/Search/Keyword?queryString=FGF10) |
| FGF11 | Secreted Factors | 18058912 | [18058912](https://www.ncbi.nlm.nih.gov/pubmed/18058912) | [FGF11](https://www.genecards.org/Search/Keyword?queryString=FGF11) |
| FGF12 | Secreted Factors | 18058912 | [18058912](https://www.ncbi.nlm.nih.gov/pubmed/18058912) | [FGF12](https://www.genecards.org/Search/Keyword?queryString=FGF12) |
| FGF13 | Secreted Factors | 18058912 | [18058912](https://www.ncbi.nlm.nih.gov/pubmed/18058912) | [FGF13](https://www.genecards.org/Search/Keyword?queryString=FGF13) |
| FGF14 | Secreted Factors | 18058912 | [18058912](https://www.ncbi.nlm.nih.gov/pubmed/18058912) | [FGF14](https://www.genecards.org/Search/Keyword?queryString=FGF14) |
| FGF16 | Secreted Factors | 18058912 | [18058912](https://www.ncbi.nlm.nih.gov/pubmed/18058912) | [FGF16](https://www.genecards.org/Search/Keyword?queryString=FGF16) |
| FGF17 | Secreted Factors | 18058912 | [18058912](https://www.ncbi.nlm.nih.gov/pubmed/18058912) | [FGF17](https://www.genecards.org/Search/Keyword?queryString=FGF17) |
| FGF18 | Secreted Factors | 18058912 | [18058912](https://www.ncbi.nlm.nih.gov/pubmed/18058912) | [FGF18](https://www.genecards.org/Search/Keyword?queryString=FGF18) |
| FGF19 | Secreted Factors | 18058912 | [18058912](https://www.ncbi.nlm.nih.gov/pubmed/18058912) | [FGF19](https://www.genecards.org/Search/Keyword?queryString=FGF19) |
| FGF2 | Secreted Factors | 18058912 | [18058912](https://www.ncbi.nlm.nih.gov/pubmed/18058912) | [FGF2](https://www.genecards.org/Search/Keyword?queryString=FGF2) |
| FGF20 | Secreted Factors | 18058912 | [18058912](https://www.ncbi.nlm.nih.gov/pubmed/18058912) | [FGF20](https://www.genecards.org/Search/Keyword?queryString=FGF20) |
| FGF21 | Secreted Factors | 18058912 | [18058912](https://www.ncbi.nlm.nih.gov/pubmed/18058912) | [FGF21](https://www.genecards.org/Search/Keyword?queryString=FGF21) |
| FGF22 | Secreted Factors | 18058912 | [18058912](https://www.ncbi.nlm.nih.gov/pubmed/18058912) | [FGF22](https://www.genecards.org/Search/Keyword?queryString=FGF22) |
| FGF23 | Secreted Factors | 18058912 | [18058912](https://www.ncbi.nlm.nih.gov/pubmed/18058912) | [FGF23](https://www.genecards.org/Search/Keyword?queryString=FGF23) |
| FGF3 | Secreted Factors | 18058912 | [18058912](https://www.ncbi.nlm.nih.gov/pubmed/18058912) | [FGF3](https://www.genecards.org/Search/Keyword?queryString=FGF3) |
| FGF4 | Secreted Factors | 18058912 | [18058912](https://www.ncbi.nlm.nih.gov/pubmed/18058912) | [FGF4](https://www.genecards.org/Search/Keyword?queryString=FGF4) |
| FGF5 | Secreted Factors | 18058912 | [18058912](https://www.ncbi.nlm.nih.gov/pubmed/18058912) | [FGF5](https://www.genecards.org/Search/Keyword?queryString=FGF5) |
| FGF6 | Secreted Factors | 18058912 | [18058912](https://www.ncbi.nlm.nih.gov/pubmed/18058912) | [FGF6](https://www.genecards.org/Search/Keyword?queryString=FGF6) |
| FGF7 | Secreted Factors | 18058912 | [18058912](https://www.ncbi.nlm.nih.gov/pubmed/18058912) | [FGF7](https://www.genecards.org/Search/Keyword?queryString=FGF7) |
| FGF8 | Secreted Factors | 18058912 | [18058912](https://www.ncbi.nlm.nih.gov/pubmed/18058912) | [FGF8](https://www.genecards.org/Search/Keyword?queryString=FGF8) |
| FGF9 | Secreted Factors | 18058912 | [18058912](https://www.ncbi.nlm.nih.gov/pubmed/18058912) | [FGF9](https://www.genecards.org/Search/Keyword?queryString=FGF9) |
| FGFBP1 | Secreted Factors | 29158353 | [29158353](https://www.ncbi.nlm.nih.gov/pubmed/29158353) | [FGFBP1](https://www.genecards.org/Search/Keyword?queryString=FGFBP1) |
| FGFBP2 | Secreted Factors | 11509569 | [11509569](https://www.ncbi.nlm.nih.gov/pubmed/11509569) | [FGFBP2](https://www.genecards.org/Search/Keyword?queryString=FGFBP2) |
| FGFBP3 | Secreted Factors | 22159717 | [22159717](https://www.ncbi.nlm.nih.gov/pubmed/22159717 ) | [FGFBP3](https://www.genecards.org/Search/Keyword?queryString=FGFBP3) |
| FGFR1 | ECM Micro-enviornment associated | 19696444 | [19696444](https://www.ncbi.nlm.nih.gov/pubmed/19696444) | [FGFR1](https://www.genecards.org/Search/Keyword?queryString=FGFR1) |
| FGFR2 | ECM Micro-enviornment associated | 12162872 | [12162872](https://www.ncbi.nlm.nih.gov/pubmed/12162872) | [FGFR2](https://www.genecards.org/Search/Keyword?queryString=FGFR2) |
| FGFR3 | ECM Micro-enviornment associated | 25223521 | [25223521](https://www.ncbi.nlm.nih.gov/pubmed/25223521) | [FGFR3](https://www.genecards.org/Search/Keyword?queryString=FGFR3) |
| FGFR4 | ECM Micro-enviornment associated | 20798051 | [20798051](https://www.ncbi.nlm.nih.gov/pubmed/20798051) | [FGFR4](https://www.genecards.org/Search/Keyword?queryString=FGFR4) |
| FGG | ECM Glycoproteins | 27988214 | [27988214](https://www.ncbi.nlm.nih.gov/pubmed/27988214) | [FGG](https://www.genecards.org/Search/Keyword?queryString=FGG) |
| FGL1 | ECM Glycoproteins | 27007659 | [27007659](https://www.ncbi.nlm.nih.gov/pubmed/27007659) | [FGL1](https://www.genecards.org/Search/Keyword?queryString=FGL1) |
| FGL2 | ECM Glycoproteins | 28656005 | [28656005](https://www.ncbi.nlm.nih.gov/pubmed/28656005) | [FGL2](https://www.genecards.org/Search/Keyword?queryString=FGL2) |
| FIBP | ECM Micro-enviornment associated | 15781320 | [15781320](https://www.ncbi.nlm.nih.gov/pubmed/15781320) | [FIBP](https://www.genecards.org/Search/Keyword?queryString=FIBP) |
| FIGF | Secreted Factors | 19700746 | [19700746](https://www.ncbi.nlm.nih.gov/pubmed/19700746) | [FIGF](https://www.genecards.org/Search/Keyword?queryString=FIGF) |
| FKRP | ECM Micro-enviornment associated | 11592034 | [11592034](https://www.ncbi.nlm.nih.gov/pubmed/11592034) | [FKRP](https://www.genecards.org/Search/Keyword?queryString=FKRP) |
| FLG | Secreted Factors | 22164253 | [22164253](https://www.ncbi.nlm.nih.gov/pubmed/22164253) | [FLG](https://www.genecards.org/Search/Keyword?queryString=FLG) |
| FLG2 | Secreted Factors | 19384417 | [19384417](https://www.ncbi.nlm.nih.gov/pubmed/19384417) | [FLG2](https://www.genecards.org/Search/Keyword?queryString=FLG2) |
| FLNA | ECM Micro-enviornment associated | 20171211 | [20171211](https://www.ncbi.nlm.nih.gov/pubmed/20171211) | [FLNA](https://www.genecards.org/Search/Keyword?queryString=FLNA) |
| FLNB | ECM Micro-enviornment associated | 25925610 | [25925610](https://www.ncbi.nlm.nih.gov/pubmed/25925610) | [FLNB](https://www.genecards.org/Search/Keyword?queryString=FLNB) |
| FLNC | ECM Micro-enviornment associated | 21423176 | [21423176](https://www.ncbi.nlm.nih.gov/pubmed/21423176) | [FLNC](https://www.genecards.org/Search/Keyword?queryString=FLNC) |
| FLRT1 | ECM Micro-enviornment associated | 10644439 | [10644439](https://www.ncbi.nlm.nih.gov/pubmed/10644439) | [FLRT1](https://www.genecards.org/Search/Keyword?queryString=FLRT1) |
| FLRT3 | ECM Micro-enviornment associated | 10644439 | [10644439](https://www.ncbi.nlm.nih.gov/pubmed/10644439) | [FLRT3](https://www.genecards.org/Search/Keyword?queryString=FLRT3) |
| FLT3LG | Secreted Factors | 22159717 | [22159717](https://www.ncbi.nlm.nih.gov/pubmed/22159717 ) | [FLT3LG](https://www.genecards.org/Search/Keyword?queryString=FLT3LG) |
| FMOD | Proteoglycans | 27069062 | [27069062](https://www.ncbi.nlm.nih.gov/pubmed/27069062) | [FMOD](https://www.genecards.org/Search/Keyword?queryString=FMOD) |
| FN1 | ECM Glycoproteins | 23419153 | [23419153](https://www.ncbi.nlm.nih.gov/pubmed/23419153) | [FN1](https://www.genecards.org/Search/Keyword?queryString=FN1) |
| FNDC1 | ECM Glycoproteins | 27053524 | [27053524](https://www.ncbi.nlm.nih.gov/pubmed/27053524) | [FNDC1](https://www.genecards.org/Search/Keyword?queryString=FNDC1) |
| FNDC7 | ECM Glycoproteins | 28558659 | [28558659](https://www.ncbi.nlm.nih.gov/pubmed/28558659) | [FNDC7](https://www.genecards.org/Search/Keyword?queryString=FNDC7) |
| FNDC8 | ECM Glycoproteins | 17562024 | [17562024](https://www.ncbi.nlm.nih.gov/pubmed/17562024) | [FNDC8](https://www.genecards.org/Search/Keyword?queryString=FNDC8) |
| FOS | ECM Micro-enviornment associated | 1383501 | [1383501](https://www.ncbi.nlm.nih.gov/pubmed/1383501) | [FOS](https://www.genecards.org/Search/Keyword?queryString=FOS) |
| FOXL1 | ECM Micro-enviornment associated | 11555641 | [11555641](https://www.ncbi.nlm.nih.gov/pubmed/11555641) | [FOXL1](https://www.genecards.org/Search/Keyword?queryString=FOXL1) |
| FRAS1 | ECM Glycoproteins | 22159717 | [22159717](https://www.ncbi.nlm.nih.gov/pubmed/22159717) | [FRAS1](https://www.genecards.org/Search/Keyword?queryString=FRAS1) |
| FREM1 | ECM-affiliated Proteins | 15345741 | [15345741](https://www.ncbi.nlm.nih.gov/pubmed/15345741) | [FREM1](https://www.genecards.org/Search/Keyword?queryString=FREM1) |
| FREM2 | ECM-affiliated Proteins | 22159717 | [22159717](https://www.ncbi.nlm.nih.gov/pubmed/22159717 ) | [FREM2](https://www.genecards.org/Search/Keyword?queryString=FREM2) |
| FREM3 | ECM-affiliated Proteins | 22159717 | [22159717](https://www.ncbi.nlm.nih.gov/pubmed/22159717 ) | [FREM3](https://www.genecards.org/Search/Keyword?queryString=FREM3) |
| FRS2 | ECM Micro-enviornment associated | 20876804 | [20876804](https://www.ncbi.nlm.nih.gov/pubmed/20876804) | [FRS2](https://www.genecards.org/Search/Keyword?queryString=FRS2) |
| FRZB | Secreted Factors | 9118218 | [9118218](https://www.ncbi.nlm.nih.gov/pubmed/9118218) | [FRZB](https://www.genecards.org/Search/Keyword?queryString=FRZB) |
| FSCN1 | Cytoskeleton | 9362073 | [9362073](https://www.ncbi.nlm.nih.gov/pubmed/9362073) | [FSCN1](https://www.genecards.org/Search/Keyword?queryString=FSCN1) |
| FST | Secreted Factors | 19276183 | [19276183](https://www.ncbi.nlm.nih.gov/pubmed/19276183) | [FST](https://www.genecards.org/Search/Keyword?queryString=FST) |
| FSTL1 | Secreted Factors | 24641944 | [24641944](https://www.ncbi.nlm.nih.gov/pubmed/24641944) | [FSTL1](https://www.genecards.org/Search/Keyword?queryString=FSTL1) |
| FSTL3 | Secreted Factors | 26629006 | [26629006](https://www.ncbi.nlm.nih.gov/pubmed/26629006) | [FSTL3](https://www.genecards.org/Search/Keyword?queryString=FSTL3) |
| FZD1 | ECM Micro-enviornment associated | 22303445 | [22303445](https://www.ncbi.nlm.nih.gov/pubmed/22303445) | [FZD1](https://www.genecards.org/Search/Keyword?queryString=FZD1) |
| FZD10 | ECM Micro-enviornment associated | 29844390 | [29844390](https://www.ncbi.nlm.nih.gov/pubmed/29844390) | [FZD10](https://www.genecards.org/Search/Keyword?queryString=FZD10) |
| FZD2 | ECM Micro-enviornment associated | 18478089 | [18478089](https://www.ncbi.nlm.nih.gov/pubmed/18478089) | [FZD2](https://www.genecards.org/Search/Keyword?queryString=FZD2) |
| FZD3 | ECM Micro-enviornment associated | 26849959 | [26849959](https://www.ncbi.nlm.nih.gov/pubmed/26849959) | [FZD3](https://www.genecards.org/Search/Keyword?queryString=FZD3) |
| FZD4 | ECM Micro-enviornment associated | 23376485 | [23376485](https://www.ncbi.nlm.nih.gov/pubmed/23376485) | [FZD4](https://www.genecards.org/Search/Keyword?queryString=FZD4) |
| FZD5 | ECM Micro-enviornment associated | 26854061 | [26854061](https://www.ncbi.nlm.nih.gov/pubmed/26854061) | [FZD5](https://www.genecards.org/Search/Keyword?queryString=FZD5) |
| FZD6 | ECM Micro-enviornment associated | 26854061 | [26854061](https://www.ncbi.nlm.nih.gov/pubmed/26854061) | [FZD6](https://www.genecards.org/Search/Keyword?queryString=FZD6) |
| FZD7 | ECM Micro-enviornment associated | 26854061 | [26854061](https://www.ncbi.nlm.nih.gov/pubmed/26854061) | [FZD7](https://www.genecards.org/Search/Keyword?queryString=FZD7) |
| FZD8 | ECM Micro-enviornment associated | 26854061 | [26854061](https://www.ncbi.nlm.nih.gov/pubmed/26854061) | [FZD8](https://www.genecards.org/Search/Keyword?queryString=FZD8) |
| FZD9 | ECM Micro-enviornment associated | 26854061 | [26854061](https://www.ncbi.nlm.nih.gov/pubmed/26854061) | [FZD9](https://www.genecards.org/Search/Keyword?queryString=FZD9) |
| G6PC | ECM Micro-enviornment associated | 7992553 | [7992553](https://www.ncbi.nlm.nih.gov/pubmed/7992553) | [G6PC](https://www.genecards.org/Search/Keyword?queryString=G6PC) |
| GAB1 | ECM Micro-enviornment associated | 20723025 | [20723025](https://www.ncbi.nlm.nih.gov/pubmed/20723025) | [GAB1](https://www.genecards.org/Search/Keyword?queryString=GAB1) |
| GAD1 | ECM synthetic/degradation enzyme | 19429183 | [19429183](https://www.ncbi.nlm.nih.gov/pubmed/19429183) | [GAD1](https://www.genecards.org/Search/Keyword?queryString=GAD1) |
| GAL3ST3 | ECM synthetic/degradation enzyme | 11356829 | [11356829](https://www.ncbi.nlm.nih.gov/pubmed/11356829) | [GAL3ST3](https://www.genecards.org/Search/Keyword?queryString=GAL3ST3) |
| GAL3ST4 | ECM synthetic/degradation enzyme | 11333265 | [11333265](https://www.ncbi.nlm.nih.gov/pubmed/11333265) | [GAL3ST4](https://www.genecards.org/Search/Keyword?queryString=GAL3ST4) |
| GALE | ECM synthetic/degradation enzyme | 25201731 | [25201731](https://www.ncbi.nlm.nih.gov/pubmed/25201731) | [GALE](https://www.genecards.org/Search/Keyword?queryString=GALE) |
| GALNS | ECM synthetic/degradation enzyme | 1755850 | [1755850](https://www.ncbi.nlm.nih.gov/pubmed/1755850) | [GALNS](https://www.genecards.org/Search/Keyword?queryString=GALNS) |
| GAP43 | Cytoskeleton | 11433297 | [11433297](https://www.ncbi.nlm.nih.gov/pubmed/11433297) | [GAP43](https://www.genecards.org/Search/Keyword?queryString=GAP43) |
| GAS6 | ECM Glycoproteins | 26356564 | [26356564](https://www.ncbi.nlm.nih.gov/pubmed/26356564) | [GAS6](https://www.genecards.org/Search/Keyword?queryString=GAS6) |
| GC | ECM Micro-enviornment associated | 10438954 | [10438954](https://www.ncbi.nlm.nih.gov/pubmed/10438954) | [GC](https://www.genecards.org/Search/Keyword?queryString=GC) |
| GDF1 | Secreted Factors | 19276183 | [19276183](https://www.ncbi.nlm.nih.gov/pubmed/19276183) | [GDF1](https://www.genecards.org/Search/Keyword?queryString=GDF1) |
| GDF10 | Secreted Factors | 26502261 | [26502261](https://www.ncbi.nlm.nih.gov/pubmed/26502261) | [GDF10](https://www.genecards.org/Search/Keyword?queryString=GDF10) |
| GDF11 | Secreted Factors | 19276183 | [19276183](https://www.ncbi.nlm.nih.gov/pubmed/19276183) | [GDF11](https://www.genecards.org/Search/Keyword?queryString=GDF11) |
| GDF15 | Secreted Factors | 29724997 | [29724997](https://www.ncbi.nlm.nih.gov/pubmed/29724997) | [GDF15](https://www.genecards.org/Search/Keyword?queryString=GDF15) |
| GDF2 | Secreted Factors | 26550465 | [26550465](https://www.ncbi.nlm.nih.gov/pubmed/26550465) | [GDF2](https://www.genecards.org/Search/Keyword?queryString=GDF2) |
| GDF3 | Secreted Factors | 22159717 | [22159717](https://www.ncbi.nlm.nih.gov/pubmed/22159717 ) | [GDF3](https://www.genecards.org/Search/Keyword?queryString=GDF3) |
| GDF5 | Secreted Factors | 24561281 | [24561281](https://www.ncbi.nlm.nih.gov/pubmed/24561281) | [GDF5](https://www.genecards.org/Search/Keyword?queryString=GDF5) |
| GDF6 | Secreted Factors | 24618041 | [24618041](https://www.ncbi.nlm.nih.gov/pubmed/24618041) | [GDF6](https://www.genecards.org/Search/Keyword?queryString=GDF6) |
| GDF7 | Secreted Factors | 27075568 | [27075568](https://www.ncbi.nlm.nih.gov/pubmed/27075568) | [GDF7](https://www.genecards.org/Search/Keyword?queryString=GDF7) |
| GDF9 | Secreted Factors | 23382188 | [23382188](https://www.ncbi.nlm.nih.gov/pubmed/23382188) | [GDF9](https://www.genecards.org/Search/Keyword?queryString=GDF9) |
| GDNF | Secreted Factors | 17537792 | [17537792](https://www.ncbi.nlm.nih.gov/pubmed/17537792) | [GDNF](https://www.genecards.org/Search/Keyword?queryString=GDNF) |
| GH1 | Secreted Factors | 22159717 | [22159717](https://www.ncbi.nlm.nih.gov/pubmed/22159717 ) | [GH1](https://www.genecards.org/Search/Keyword?queryString=GH1) |
| GH2 | Secreted Factors | 22159717 | [22159717](https://www.ncbi.nlm.nih.gov/pubmed/22159717 ) | [GH2](https://www.genecards.org/Search/Keyword?queryString=GH2) |
| GLB1 | ECM synthetic/degradation enzyme | 22178079 | [22178079](https://www.ncbi.nlm.nih.gov/pubmed/22178079) | [GLB1](https://www.genecards.org/Search/Keyword?queryString=GLB1) |
| GLCE | ECM synthetic/degradation enzyme | 14718527 | [14718527](https://www.ncbi.nlm.nih.gov/pubmed/14718527) | [GLCE](https://www.genecards.org/Search/Keyword?queryString=GLCE) |
| GLDN | ECM Glycoproteins | 23419153 | [23419153](https://www.ncbi.nlm.nih.gov/pubmed/23419153) | [GLDN](https://www.genecards.org/Search/Keyword?queryString=GLDN) |
| GNPTAB | ECM synthetic/degradation enzyme | 19159218 | [19159218](https://www.ncbi.nlm.nih.gov/pubmed/19159218) | [GNPTAB](https://www.genecards.org/Search/Keyword?queryString=GNPTAB) |
| GPC1 | ECM-affiliated Proteins | 21554204 | [21554204](https://www.ncbi.nlm.nih.gov/pubmed/ 21554204) | [GPC1](https://www.genecards.org/Search/Keyword?queryString=GPC1) |
| GPC2 | ECM-affiliated Proteins | 21554204 | [21554204](https://www.ncbi.nlm.nih.gov/pubmed/ 21554204) | [GPC2](https://www.genecards.org/Search/Keyword?queryString=GPC2) |
| GPC3 | ECM-affiliated Proteins | 21554204 | [21554204](https://www.ncbi.nlm.nih.gov/pubmed/ 21554204) | [GPC3](https://www.genecards.org/Search/Keyword?queryString=GPC3) |
| GPC4 | ECM-affiliated Proteins | 21554204 | [21554204](https://www.ncbi.nlm.nih.gov/pubmed/ 21554204) | [GPC4](https://www.genecards.org/Search/Keyword?queryString=GPC4) |
| GPC5 | ECM-affiliated Proteins | 21554204 | [21554204](https://www.ncbi.nlm.nih.gov/pubmed/ 21554204) | [GPC5](https://www.genecards.org/Search/Keyword?queryString=GPC5) |
| GPC6 | ECM-affiliated Proteins | 21554204 | [21554204](https://www.ncbi.nlm.nih.gov/pubmed/ 21554204) | [GPC6](https://www.genecards.org/Search/Keyword?queryString=GPC6) |
| GPD1 | ECM synthetic/degradation enzyme | 8028517 | [8028517](https://www.ncbi.nlm.nih.gov/pubmed/8028517) | [GPD1](https://www.genecards.org/Search/Keyword?queryString=GPD1) |
| GPLD1 | ECM synthetic/degradation enzyme | 8161780 | [8161780](https://www.ncbi.nlm.nih.gov/pubmed/8161780) | [GPLD1](https://www.genecards.org/Search/Keyword?queryString=GPLD1) |
| GRB2 | ECM Micro-enviornment associated | 15182856 | [15182856](https://www.ncbi.nlm.nih.gov/pubmed/15182856) | [GRB2](https://www.genecards.org/Search/Keyword?queryString=GRB2) |
| GREM1 | ECM-affiliated Proteins | 21642622 | [21642622](https://www.ncbi.nlm.nih.gov/pubmed/21642622) | [GREM1](https://www.genecards.org/Search/Keyword?queryString=GREM1) |
| GRIFIN | ECM-affiliated Proteins | 9786891 | [9786891](https://www.ncbi.nlm.nih.gov/pubmed/9786891) | [GRIFIN](https://www.genecards.org/Search/Keyword?queryString=GRIFIN) |
| GRIP1 | ECM Micro-enviornment associated | 12458226 | [12458226](https://www.ncbi.nlm.nih.gov/pubmed/12458226) | [GRIP1](https://www.genecards.org/Search/Keyword?queryString=GRIP1) |
| GRIP2 | ECM Micro-enviornment associated | 12458226 | [12458226](https://www.ncbi.nlm.nih.gov/pubmed/12458226) | [GRIP2](https://www.genecards.org/Search/Keyword?queryString=GRIP2) |
| GYPC | ECM Micro-enviornment associated | 2818576 | [2818576](https://www.ncbi.nlm.nih.gov/pubmed/2818576) | [GYPC](https://www.genecards.org/Search/Keyword?queryString=GYPC) |
| GZMA | ECM Micro-enviornment associated | 10666226 | [10666226](https://www.ncbi.nlm.nih.gov/pubmed/10666226) | [GZMA](https://www.genecards.org/Search/Keyword?queryString=GZMA) |
| GZMB | ECM Micro-enviornment associated | 8258716 | [8258716](https://www.ncbi.nlm.nih.gov/pubmed/ 8258716) | [GZMB](https://www.genecards.org/Search/Keyword?queryString=GZMB) |
| GZMM | ECM Micro-enviornment associated | 15494398 | [15494398](https://www.ncbi.nlm.nih.gov/pubmed/15494398) | [GZMM](https://www.genecards.org/Search/Keyword?queryString=GZMM) |
| HABP2 | ECM Regulators | 30143058 | [30143058](https://www.ncbi.nlm.nih.gov/pubmed/30143058) | [HABP2](https://www.genecards.org/Search/Keyword?queryString=HABP2) |
| HAPLN1 | Proteoglycans | 30078576 | [30078576](https://www.ncbi.nlm.nih.gov/pubmed/30078576) | [HAPLN1](https://www.genecards.org/Search/Keyword?queryString=HAPLN1) |
| HAPLN2 | Proteoglycans | 27601046 | [27601046](https://www.ncbi.nlm.nih.gov/pubmed/27601046) | [HAPLN2](https://www.genecards.org/Search/Keyword?queryString=HAPLN2) |
| HAPLN3 | Proteoglycans | 21618406 | [21618406](https://www.ncbi.nlm.nih.gov/pubmed/21618406) | [HAPLN3](https://www.genecards.org/Search/Keyword?queryString=HAPLN3) |
| HAPLN4 | Proteoglycans | 27351915 | [27351915](https://www.ncbi.nlm.nih.gov/pubmed/27351915) | [HAPLN4](https://www.genecards.org/Search/Keyword?queryString=HAPLN4) |
| HAS1 | ECM synthetic/degradation enzyme | 23123404 | [23123404](https://www.ncbi.nlm.nih.gov/pubmed/23123404) | [HAS1](https://www.genecards.org/Search/Keyword?queryString=HAS1) |
| HAS2 | ECM synthetic/degradation enzyme | 19231585 | [19231585](https://www.ncbi.nlm.nih.gov/pubmed/19231585) | [HAS2](https://www.genecards.org/Search/Keyword?queryString=HAS2) |
| HBEGF | Secreted Factors | 24345808 | [24345808](https://www.ncbi.nlm.nih.gov/pubmed/24345808) | [HBEGF](https://www.genecards.org/Search/Keyword?queryString=HBEGF) |
| HCFC1 | Secreted Factors | 22159717 | [22159717](https://www.ncbi.nlm.nih.gov/pubmed/22159717 ) | [HCFC1](https://www.genecards.org/Search/Keyword?queryString=HCFC1) |
| HCFC2 | Secreted Factors | 22159717 | [22159717](https://www.ncbi.nlm.nih.gov/pubmed/22159717 ) | [HCFC2](https://www.genecards.org/Search/Keyword?queryString=HCFC2) |
| HCLS1 | ECM Micro-enviornment associated | 8305739 | [8305739](https://www.ncbi.nlm.nih.gov/pubmed/ 8305739) | [HCLS1](https://www.genecards.org/Search/Keyword?queryString=HCLS1) |
| HDC | ECM Micro-enviornment associated | 18234316 | [18234316](https://www.ncbi.nlm.nih.gov/pubmed/18234316) | [HDC](https://www.genecards.org/Search/Keyword?queryString=HDC) |
| HGF | Secreted Factors | 28094206 | [28094206](https://www.ncbi.nlm.nih.gov/pubmed/28094206) | [HGF](https://www.genecards.org/Search/Keyword?queryString=HGF) |
| HGFAC | Secreted Factors | 22159717 | [22159717](https://www.ncbi.nlm.nih.gov/pubmed/22159717 ) | [HGFAC](https://www.genecards.org/Search/Keyword?queryString=HGFAC) |
| HHIP | Secreted Factors | 23459001 | [23459001](https://www.ncbi.nlm.nih.gov/pubmed/23459001) | [HHIP](https://www.genecards.org/Search/Keyword?queryString=HHIP) |
| HIF1A | ECM Micro-enviornment associated | 19790048 | [19790048](https://www.ncbi.nlm.nih.gov/pubmed/19790048) | [HIF1A](https://www.genecards.org/Search/Keyword?queryString=HIF1A) |
| HMCN1 | ECM Glycoproteins | 23419153 | [23419153](https://www.ncbi.nlm.nih.gov/pubmed/23419153) | [HMCN1](https://www.genecards.org/Search/Keyword?queryString=HMCN1) |
| HMCN2 | ECM Glycoproteins | 23419153 | [23419153](https://www.ncbi.nlm.nih.gov/pubmed/23419153) | [HMCN2](https://www.genecards.org/Search/Keyword?queryString=HMCN2) |
| HMGB1 | ECM Micro-enviornment associated | 20007974 | [20007974](https://www.ncbi.nlm.nih.gov/pubmed/20007974) | [HMGB1](https://www.genecards.org/Search/Keyword?queryString=HMGB1) |
| HMMR | ECM Micro-enviornment associated | 1705114 | [1705114](https://www.ncbi.nlm.nih.gov/pubmed/1705114) | [HMMR](https://www.genecards.org/Search/Keyword?queryString=HMMR) |
| HMSD | ECM Regulators | 22159717 | [22159717](https://www.ncbi.nlm.nih.gov/pubmed/22159717 ) | [HMSD](https://www.genecards.org/Search/Keyword?queryString=HMSD) |
| HOXD10 | ECM associated regulatory factors | 14633614 | [14633614](https://www.ncbi.nlm.nih.gov/pubmed/14633614) | [HOXD10](https://www.genecards.org/Search/Keyword?queryString=HOXD10) |
| HPSE | ECM Regulators | 25849134 | [25849134](https://www.ncbi.nlm.nih.gov/pubmed/25849134) | [HPSE](https://www.genecards.org/Search/Keyword?queryString=HPSE) |
| HPSE2 | ECM Regulators | 26084486 | [26084486](https://www.ncbi.nlm.nih.gov/pubmed/26084486) | [HPSE2](https://www.genecards.org/Search/Keyword?queryString=HPSE2) |
| HPX | ECM-affiliated Proteins | 22159717 | [22159717](https://www.ncbi.nlm.nih.gov/pubmed/22159717 ) | [HPX](https://www.genecards.org/Search/Keyword?queryString=HPX) |
| HRAS | ECM associated regulatory factors | 21211511 | [21211511](https://www.ncbi.nlm.nih.gov/pubmed/21211511) | [HRAS](https://www.genecards.org/Search/Keyword?queryString=HRAS) |
| HRG | ECM Regulators | 16138928 | [16138928](https://www.ncbi.nlm.nih.gov/pubmed/16138928) | [HRG](https://www.genecards.org/Search/Keyword?queryString=HRG) |
| HRNR | Secreted Factors | 22159717 | [22159717](https://www.ncbi.nlm.nih.gov/pubmed/22159717 ) | [HRNR](https://www.genecards.org/Search/Keyword?queryString=HRNR) |
| HS2ST1 | ECM synthetic/degradation enzyme | 25594747 | [25594747](https://www.ncbi.nlm.nih.gov/pubmed/25594747) | [HS2ST1](https://www.genecards.org/Search/Keyword?queryString=HS2ST1) |
| HS3ST1 | ECM synthetic/degradation enzyme | 15096036 | [15096036](https://www.ncbi.nlm.nih.gov/pubmed/15096036) | [HS3ST1](https://www.genecards.org/Search/Keyword?queryString=HS3ST1) |
| HS3ST2 | ECM synthetic/degradation enzyme | 9988767 | [9988767](https://www.ncbi.nlm.nih.gov/pubmed/9988767) | [HS3ST2](https://www.genecards.org/Search/Keyword?queryString=HS3ST2) |
| HS3ST5 | ECM synthetic/degradation enzyme | 12138164 | [12138164](https://www.ncbi.nlm.nih.gov/pubmed/12138164) | [HS3ST5](https://www.genecards.org/Search/Keyword?queryString=HS3ST5) |
| HS6ST1 | ECM synthetic/degradation enzyme | 9535912 | [9535912](https://www.ncbi.nlm.nih.gov/pubmed/9535912) | [HS6ST1](https://www.genecards.org/Search/Keyword?queryString=HS6ST1) |
| HS6ST2 | ECM synthetic/degradation enzyme | 10644753 | [10644753](https://www.ncbi.nlm.nih.gov/pubmed/10644753) | [HS6ST2](https://www.genecards.org/Search/Keyword?queryString=HS6ST2) |
| HSPB2 | ECM Micro-enviornment associated | 28854361 | [28854361](https://www.ncbi.nlm.nih.gov/pubmed/28854361) | [HSPB2](https://www.genecards.org/Search/Keyword?queryString=HSPB2) |
| HSPC159 | ECM-affiliated Proteins | 18320588 | [18320588](https://www.ncbi.nlm.nih.gov/pubmed/18320588) | [HSPC159](https://www.genecards.org/Search/Keyword?queryString=HSPC159) |
| HSPG2 | Proteoglycans | 22261194 | [22261194](https://www.ncbi.nlm.nih.gov/pubmed/22261194) | [HSPG2](https://www.genecards.org/Search/Keyword?queryString=HSPG2) |
| HTRA1 | ECM Regulators | 22159717 | [22159717](https://www.ncbi.nlm.nih.gov/pubmed/22159717 ) | [HTRA1](https://www.genecards.org/Search/Keyword?queryString=HTRA1) |
| HTRA2 | ECM Micro-enviornment associated | 22265821 | [22265821](https://www.ncbi.nlm.nih.gov/pubmed/22265821) | [HTRA2](https://www.genecards.org/Search/Keyword?queryString=HTRA2) |
| HTRA3 | ECM Regulators | 22159717 | [22159717](https://www.ncbi.nlm.nih.gov/pubmed/22159717 ) | [HTRA3](https://www.genecards.org/Search/Keyword?queryString=HTRA3) |
| HTRA4 | ECM Regulators | 22159717 | [22159717](https://www.ncbi.nlm.nih.gov/pubmed/22159717 ) | [HTRA4](https://www.genecards.org/Search/Keyword?queryString=HTRA4) |
| HYAL1 | ECM Regulators | 15218248 | [15218248](https://www.ncbi.nlm.nih.gov/pubmed/15218248) | [HYAL1](https://www.genecards.org/Search/Keyword?queryString=HYAL1) |
| HYAL2 | ECM Regulators | 15218248 | [15218248](https://www.ncbi.nlm.nih.gov/pubmed/15218248) | [HYAL2](https://www.genecards.org/Search/Keyword?queryString=HYAL2) |
| HYAL3 | ECM Regulators | 15218248 | [15218248](https://www.ncbi.nlm.nih.gov/pubmed/15218248) | [HYAL3](https://www.genecards.org/Search/Keyword?queryString=HYAL3) |
| HYAL4 | ECM Regulators | 11731267 | [11731267](https://www.ncbi.nlm.nih.gov/pubmed/11731267) | [HYAL4](https://www.genecards.org/Search/Keyword?queryString=HYAL4) |
| IAPP | ECM | 25922077 | [25922077](https://www.ncbi.nlm.nih.gov/pubmed/25922077) | [IAPP](https://www.genecards.org/Search/Keyword?queryString=IAPP) |
| IBSP | ECM Glycoproteins | 12646701 | [12646701](https://www.ncbi.nlm.nih.gov/pubmed/12646701) | [IBSP](https://www.genecards.org/Search/Keyword?queryString=IBSP) |
| ICAM1 | ECM Micro-enviornment associated | 18454303 | [18454303](https://www.ncbi.nlm.nih.gov/pubmed/18454303) | [ICAM1](https://www.genecards.org/Search/Keyword?queryString=ICAM1) |
| IFNA1 | Secreted Factors | 28122987 | [28122987](https://www.ncbi.nlm.nih.gov/pubmed/28122987) | [IFNA1](https://www.genecards.org/Search/Keyword?queryString=IFNA1) |
| IFNA10 | Secreted Factors | 22159717 | [22159717](https://www.ncbi.nlm.nih.gov/pubmed/ 22159717  ) | [IFNA10](https://www.genecards.org/Search/Keyword?queryString=IFNA10) |
| IFNA13 | Secreted Factors | 22159717 | [22159717](https://www.ncbi.nlm.nih.gov/pubmed/ 22159717  ) | [IFNA13](https://www.genecards.org/Search/Keyword?queryString=IFNA13) |
| IFNA14 | Secreted Factors | 22159717 | [22159717](https://www.ncbi.nlm.nih.gov/pubmed/ 22159717  ) | [IFNA14](https://www.genecards.org/Search/Keyword?queryString=IFNA14) |
| IFNA16 | Secreted Factors | 22159717 | [22159717](https://www.ncbi.nlm.nih.gov/pubmed/ 22159717  ) | [IFNA16](https://www.genecards.org/Search/Keyword?queryString=IFNA16) |
| IFNA17 | Secreted Factors | 22159717 | [22159717](https://www.ncbi.nlm.nih.gov/pubmed/ 22159717  ) | [IFNA17](https://www.genecards.org/Search/Keyword?queryString=IFNA17) |
| IFNA2 | Secreted Factors | 22159717 | [22159717](https://www.ncbi.nlm.nih.gov/pubmed/ 22159717  ) | [IFNA2](https://www.genecards.org/Search/Keyword?queryString=IFNA2) |
| IFNA21 | Secreted Factors | 22159717 | [22159717](https://www.ncbi.nlm.nih.gov/pubmed/ 22159717  ) | [IFNA21](https://www.genecards.org/Search/Keyword?queryString=IFNA21) |
| IFNA4 | Secreted Factors | 22159717 | [22159717](https://www.ncbi.nlm.nih.gov/pubmed/ 22159717  ) | [IFNA4](https://www.genecards.org/Search/Keyword?queryString=IFNA4) |
| IFNA5 | Secreted Factors | 22159717 | [22159717](https://www.ncbi.nlm.nih.gov/pubmed/ 22159717  ) | [IFNA5](https://www.genecards.org/Search/Keyword?queryString=IFNA5) |
| IFNA6 | Secreted Factors | 22159717 | [22159717](https://www.ncbi.nlm.nih.gov/pubmed/ 22159717  ) | [IFNA6](https://www.genecards.org/Search/Keyword?queryString=IFNA6) |
| IFNA7 | Secreted Factors | 22159717 | [22159717](https://www.ncbi.nlm.nih.gov/pubmed/ 22159717  ) | [IFNA7](https://www.genecards.org/Search/Keyword?queryString=IFNA7) |
| IFNA8 | Secreted Factors | 22159717 | [22159717](https://www.ncbi.nlm.nih.gov/pubmed/ 22159717  ) | [IFNA8](https://www.genecards.org/Search/Keyword?queryString=IFNA8) |
| IFNB1 | Secreted Factors | 22159717 | [22159717](https://www.ncbi.nlm.nih.gov/pubmed/ 22159717  ) | [IFNB1](https://www.genecards.org/Search/Keyword?queryString=IFNB1) |
| IFNE | Secreted Factors | 22159717 | [22159717](https://www.ncbi.nlm.nih.gov/pubmed/ 22159717  ) | [IFNE](https://www.genecards.org/Search/Keyword?queryString=IFNE) |
| IFNG | Secreted Factors | 22159717 | [22159717](https://www.ncbi.nlm.nih.gov/pubmed/ 22159717  ) | [IFNG](https://www.genecards.org/Search/Keyword?queryString=IFNG) |
| IFNK | Secreted Factors | 22159717 | [22159717](https://www.ncbi.nlm.nih.gov/pubmed/ 22159717  ) | [IFNK](https://www.genecards.org/Search/Keyword?queryString=IFNK) |
| IFNW1 | Secreted Factors | 22159717 | [22159717](https://www.ncbi.nlm.nih.gov/pubmed/ 22159717  ) | [IFNW1](https://www.genecards.org/Search/Keyword?queryString=IFNW1) |
| IGF1 | Secreted Factors | 24856835 | [24856835](https://www.ncbi.nlm.nih.gov/pubmed/24856835) | [IGF1](https://www.genecards.org/Search/Keyword?queryString=IGF1) |
| IGF1R | ECM Micro-enviornment associated | 11884618 | [11884618](https://www.ncbi.nlm.nih.gov/pubmed/11884618) | [IGF1R](https://www.genecards.org/Search/Keyword?queryString=IGF1R) |
| IGF2 | Secreted Factors | 14624766 | [14624766](https://www.ncbi.nlm.nih.gov/pubmed/14624766) | [IGF2](https://www.genecards.org/Search/Keyword?queryString=IGF2) |
| IGF2R | ECM Micro-enviornment associated | 23347038 | [23347038](https://www.ncbi.nlm.nih.gov/pubmed/23347038) | [IGF2R](https://www.genecards.org/Search/Keyword?queryString=IGF2R) |
| IGFALS | ECM Glycoproteins | 21937732 | [21937732](https://www.ncbi.nlm.nih.gov/pubmed/21937732) | [IGFALS](https://www.genecards.org/Search/Keyword?queryString=IGFALS) |
| IGFBP1 | ECM Glycoproteins | 21937732 | [21937732](https://www.ncbi.nlm.nih.gov/pubmed/21937732) | [IGFBP1](https://www.genecards.org/Search/Keyword?queryString=IGFBP1) |
| IGFBP2 | ECM Glycoproteins | 21937732 | [21937732](https://www.ncbi.nlm.nih.gov/pubmed/21937732) | [IGFBP2](https://www.genecards.org/Search/Keyword?queryString=IGFBP2) |
| IGFBP3 | ECM Glycoproteins | 21937732 | [21937732](https://www.ncbi.nlm.nih.gov/pubmed/21937732) | [IGFBP3](https://www.genecards.org/Search/Keyword?queryString=IGFBP3) |
| IGFBP4 | ECM Glycoproteins | 21937732 | [21937732](https://www.ncbi.nlm.nih.gov/pubmed/21937732) | [IGFBP4](https://www.genecards.org/Search/Keyword?queryString=IGFBP4) |
| IGFBP5 | ECM Glycoproteins | 21937732 | [21937732](https://www.ncbi.nlm.nih.gov/pubmed/21937732) | [IGFBP5](https://www.genecards.org/Search/Keyword?queryString=IGFBP5) |
| IGFBP6 | ECM Glycoproteins | 21937732 | [21937732](https://www.ncbi.nlm.nih.gov/pubmed/21937732) | [IGFBP6](https://www.genecards.org/Search/Keyword?queryString=IGFBP6) |
| IGFBP7 | ECM Glycoproteins | 21937732 | [21937732](https://www.ncbi.nlm.nih.gov/pubmed/21937732) | [IGFBP7](https://www.genecards.org/Search/Keyword?queryString=IGFBP7) |
| IGFBPL1 | ECM Glycoproteins | 21937732 | [21937732](https://www.ncbi.nlm.nih.gov/pubmed/21937732) | [IGFBPL1](https://www.genecards.org/Search/Keyword?queryString=IGFBPL1) |
| IGSF10 | ECM Glycoproteins | 21937732 | [21937732](https://www.ncbi.nlm.nih.gov/pubmed/21937732) | [IGSF10](https://www.genecards.org/Search/Keyword?queryString=IGSF10) |
| IHH | Secreted Factors | 14623441 | [14623441](https://www.ncbi.nlm.nih.gov/pubmed/14623441) | [IHH](https://www.genecards.org/Search/Keyword?queryString=IHH) |
| IL10 | Secreted Factors | 14730222 | [14730222](https://www.ncbi.nlm.nih.gov/pubmed/14730222) | [IL10](https://www.genecards.org/Search/Keyword?queryString=IL10) |
| IL11 | Secreted Factors | 25808168 | [25808168](https://www.ncbi.nlm.nih.gov/pubmed/25808168) | [IL11](https://www.genecards.org/Search/Keyword?queryString=IL11) |
| IL12A | Secreted Factors | 22159717 | [22159717](https://www.ncbi.nlm.nih.gov/pubmed/ 22159717  ) | [IL12A](https://www.genecards.org/Search/Keyword?queryString=IL12A) |
| IL12B | Secreted Factors | 22159717 | [22159717](https://www.ncbi.nlm.nih.gov/pubmed/ 22159717  ) | [IL12B](https://www.genecards.org/Search/Keyword?queryString=IL12B) |
| IL13 | Secreted Factors | 27113293 | [27113293](https://www.ncbi.nlm.nih.gov/pubmed/27113293) | [IL13](https://www.genecards.org/Search/Keyword?queryString=IL13) |
| IL15 | Secreted Factors | 22510877 | [22510877](https://www.ncbi.nlm.nih.gov/pubmed/22510877) | [IL15](https://www.genecards.org/Search/Keyword?queryString=IL15) |
| IL16 | Secreted Factors | 29196464 | [29196464](https://www.ncbi.nlm.nih.gov/pubmed/29196464) | [IL16](https://www.genecards.org/Search/Keyword?queryString=IL16) |
| IL17A | Secreted Factors | 20409152 | [20409152](https://www.ncbi.nlm.nih.gov/pubmed/20409152) | [IL17A](https://www.genecards.org/Search/Keyword?queryString=IL17A) |
| IL17B | Secreted Factors | 20409152 | [20409152](https://www.ncbi.nlm.nih.gov/pubmed/20409152) | [IL17B](https://www.genecards.org/Search/Keyword?queryString=IL17B) |
| IL17C | Secreted Factors | 20409152 | [20409152](https://www.ncbi.nlm.nih.gov/pubmed/20409152) | [IL17C](https://www.genecards.org/Search/Keyword?queryString=IL17C) |
| IL17D | Secreted Factors | 20409152 | [20409152](https://www.ncbi.nlm.nih.gov/pubmed/20409152) | [IL17D](https://www.genecards.org/Search/Keyword?queryString=IL17D) |
| IL17F | Secreted Factors | 20409152 | [20409152](https://www.ncbi.nlm.nih.gov/pubmed/20409152) | [IL17F](https://www.genecards.org/Search/Keyword?queryString=IL17F) |
| IL18 | Secreted Factors | 18547159 | [18547159](https://www.ncbi.nlm.nih.gov/pubmed/18547159) | [IL18](https://www.genecards.org/Search/Keyword?queryString=IL18) |
| IL19 | Secreted Factors | 26199463 | [26199463](https://www.ncbi.nlm.nih.gov/pubmed/26199463) | [IL19](https://www.genecards.org/Search/Keyword?queryString=IL19) |
| IL1A | Secreted Factors | 17179173 | [17179173](https://www.ncbi.nlm.nih.gov/pubmed/17179173) | [IL1A](https://www.genecards.org/Search/Keyword?queryString=IL1A) |
| IL1B | Secreted Factors | 16264101 | [16264101](https://www.ncbi.nlm.nih.gov/pubmed/16264101) | [IL1B](https://www.genecards.org/Search/Keyword?queryString=IL1B) |
| IL1F10 | Secreted Factors | 22159717 | [22159717](https://www.ncbi.nlm.nih.gov/pubmed/ 22159717  ) | [IL1F10](https://www.genecards.org/Search/Keyword?queryString=IL1F10) |
| IL1F5 | Secreted Factors | 22159717 | [22159717](https://www.ncbi.nlm.nih.gov/pubmed/ 22159717  ) | [IL1F5](https://www.genecards.org/Search/Keyword?queryString=IL1F5) |
| IL1F6 | Secreted Factors | 22159717 | [22159717](https://www.ncbi.nlm.nih.gov/pubmed/ 22159717  ) | [IL1F6](https://www.genecards.org/Search/Keyword?queryString=IL1F6) |
| IL1F7 | Secreted Factors | 22159717 | [22159717](https://www.ncbi.nlm.nih.gov/pubmed/ 22159717  ) | [IL1F7](https://www.genecards.org/Search/Keyword?queryString=IL1F7) |
| IL1F8 | Secreted Factors | 22159717 | [22159717](https://www.ncbi.nlm.nih.gov/pubmed/ 22159717  ) | [IL1F8](https://www.genecards.org/Search/Keyword?queryString=IL1F8) |
| IL1F9 | Secreted Factors | 28805975 | [28805975](https://www.ncbi.nlm.nih.gov/pubmed/28805975) | [IL1F9](https://www.genecards.org/Search/Keyword?queryString=IL1F9) |
| IL1R1 | ECM Micro-enviornment associated | 12729621 | [12729621](https://www.ncbi.nlm.nih.gov/pubmed/12729621) | [IL1R1](https://www.genecards.org/Search/Keyword?queryString=IL1R1) |
| IL1RN | Secreted Factors | 22159717 | [22159717](https://www.ncbi.nlm.nih.gov/pubmed/ 22159717  ) | [IL1RN](https://www.genecards.org/Search/Keyword?queryString=IL1RN) |
| IL2 | Secreted Factors | 10651945 | [10651945](https://www.ncbi.nlm.nih.gov/pubmed/10651945) | [IL2](https://www.genecards.org/Search/Keyword?queryString=IL2) |
| IL20 | Secreted Factors | 24470401 | [24470401](https://www.ncbi.nlm.nih.gov/pubmed/24470401) | [IL20](https://www.genecards.org/Search/Keyword?queryString=IL20) |
| IL22 | Secreted Factors | 23223145 | [23223145](https://www.ncbi.nlm.nih.gov/pubmed/23223145) | [IL22](https://www.genecards.org/Search/Keyword?queryString=IL22) |
| IL23A | Secreted Factors | 22159717 | [22159717](https://www.ncbi.nlm.nih.gov/pubmed/ 22159717  ) | [IL23A](https://www.genecards.org/Search/Keyword?queryString=IL23A) |
| IL24 | Secreted Factors | 12843410 | [12843410](https://www.ncbi.nlm.nih.gov/pubmed/12843410) | [IL24](https://www.genecards.org/Search/Keyword?queryString=IL24) |
| IL25 | Secreted Factors | 28771607 | [28771607](https://www.ncbi.nlm.nih.gov/pubmed/28771607) | [IL25](https://www.genecards.org/Search/Keyword?queryString=IL25) |
| IL26 | Secreted Factors | 28852311 | [28852311](https://www.ncbi.nlm.nih.gov/pubmed/28852311) | [IL26](https://www.genecards.org/Search/Keyword?queryString=IL26) |
| IL3 | Secreted Factors | 14623967 | [14623967](https://www.ncbi.nlm.nih.gov/pubmed/14623967) | [IL3](https://www.genecards.org/Search/Keyword?queryString=IL3) |
| IL34 | Secreted Factors | 20504948 | [20504948](https://www.ncbi.nlm.nih.gov/pubmed/20504948) | [IL34](https://www.genecards.org/Search/Keyword?queryString=IL34) |
| IL4 | Secreted Factors | 15644118 | [15644118](https://www.ncbi.nlm.nih.gov/pubmed/15644118) | [IL4](https://www.genecards.org/Search/Keyword?queryString=IL4) |
| IL5 | Secreted Factors | 16210053 | [16210053](https://www.ncbi.nlm.nih.gov/pubmed/16210053) | [IL5](https://www.genecards.org/Search/Keyword?queryString=IL5) |
| IL6 | Secreted Factors | 24449804 | [24449804](https://www.ncbi.nlm.nih.gov/pubmed/24449804) | [IL6](https://www.genecards.org/Search/Keyword?queryString=IL6) |
| IL6R | ECM Micro-enviornment associated | 19790045 | [19790045](https://www.ncbi.nlm.nih.gov/pubmed/19790045) | [IL6R](https://www.genecards.org/Search/Keyword?queryString=IL6R) |
| IL6ST | ECM Micro-enviornment associated | 15890357 | [15890357](https://www.ncbi.nlm.nih.gov/pubmed/15890357) | [IL6ST](https://www.genecards.org/Search/Keyword?queryString=IL6ST) |
| IL7 | Secreted Factors | 16769608 | [16769608](https://www.ncbi.nlm.nih.gov/pubmed/16769608) | [IL7](https://www.genecards.org/Search/Keyword?queryString=IL7) |
| IL9 | Secreted Factors | 25646460 | [25646460](https://www.ncbi.nlm.nih.gov/pubmed/25646460) | [IL9](https://www.genecards.org/Search/Keyword?queryString=IL9) |
| IMPG1 | Proteoglycans | 29777959 | [29777959](https://www.ncbi.nlm.nih.gov/pubmed/29777959) | [IMPG1](https://www.genecards.org/Search/Keyword?queryString=IMPG1) |
| IMPG2 | Proteoglycans | 29777959 | [29777959](https://www.ncbi.nlm.nih.gov/pubmed/29777959) | [IMPG2](https://www.genecards.org/Search/Keyword?queryString=IMPG2) |
| INHA | Secreted Factors | 22159717 | [22159717](https://www.ncbi.nlm.nih.gov/pubmed/ 22159717  ) | [INHA](https://www.genecards.org/Search/Keyword?queryString=INHA) |
| INHBA | Secreted Factors | 21828177 | [21828177](https://www.ncbi.nlm.nih.gov/pubmed/21828177) | [INHBA](https://www.genecards.org/Search/Keyword?queryString=INHBA) |
| INHBB | Secreted Factors | 17881772 | [17881772](https://www.ncbi.nlm.nih.gov/pubmed/17881772) | [INHBB](https://www.genecards.org/Search/Keyword?queryString=INHBB) |
| INHBC | Secreted Factors | 22159717 | [22159717](https://www.ncbi.nlm.nih.gov/pubmed/ 22159717  ) | [INHBC](https://www.genecards.org/Search/Keyword?queryString=INHBC) |
| INHBE | Secreted Factors | 22159717 | [22159717](https://www.ncbi.nlm.nih.gov/pubmed/ 22159717  ) | [INHBE](https://www.genecards.org/Search/Keyword?queryString=INHBE) |
| INS | Secreted Factors | 9667398 | [9667398](https://www.ncbi.nlm.nih.gov/pubmed/9667398) | [INS](https://www.genecards.org/Search/Keyword?queryString=INS) |
| INS-IGF2 | Secreted Factors | 22159717 | [22159717](https://www.ncbi.nlm.nih.gov/pubmed/ 22159717  ) | [INS-IGF2](https://www.genecards.org/Search/Keyword?queryString=INS-IGF2) |
| INSL3 | Secreted Factors | 27059798 | [27059798](https://www.ncbi.nlm.nih.gov/pubmed/27059798) | [INSL3](https://www.genecards.org/Search/Keyword?queryString=INSL3) |
| INSL5 | Secreted Factors | 22159717 | [22159717](https://www.ncbi.nlm.nih.gov/pubmed/ 22159717  ) | [INSL5](https://www.genecards.org/Search/Keyword?queryString=INSL5) |
| INSL6 | Secreted Factors | 22159717 | [22159717](https://www.ncbi.nlm.nih.gov/pubmed/ 22159717  ) | [INSL6](https://www.genecards.org/Search/Keyword?queryString=INSL6) |
| IQGAP1 | ECM Micro-enviornment associated | 21423176 | [21423176](https://www.ncbi.nlm.nih.gov/pubmed/21423176) | [IQGAP1](https://www.genecards.org/Search/Keyword?queryString=IQGAP1) |
| IRS1 | ECM Micro-enviornment associated | 10428812 | [10428812](https://www.ncbi.nlm.nih.gov/pubmed/10428812) | [IRS1](https://www.genecards.org/Search/Keyword?queryString=IRS1) |
| ISM1 | Secreted Factors | 22159717 | [22159717](https://www.ncbi.nlm.nih.gov/pubmed/ 22159717  ) | [ISM1](https://www.genecards.org/Search/Keyword?queryString=ISM1) |
| ISM2 | Secreted Factors | 22159717 | [22159717](https://www.ncbi.nlm.nih.gov/pubmed/ 22159717  ) | [ISM2](https://www.genecards.org/Search/Keyword?queryString=ISM2) |
| ITFG1 | ECM receptor | 10490955 | [10490955](https://www.ncbi.nlm.nih.gov/pubmed/10490955) | [ITFG1](https://www.genecards.org/Search/Keyword?queryString=ITFG1) |
| ITGA2 | ECM receptor | 18990704 | [18990704](https://www.ncbi.nlm.nih.gov/pubmed/18990704) | [ITGA2](https://www.genecards.org/Search/Keyword?queryString=ITGA2) |
| ITGA2B | ECM receptor | 11728949 | [11728949](https://www.ncbi.nlm.nih.gov/pubmed/11728949) | [ITGA2B](https://www.genecards.org/Search/Keyword?queryString=ITGA2B) |
| ITGA3 | ECM receptor | 1655803 | [1655803](https://www.ncbi.nlm.nih.gov/pubmed/1655803) | [ITGA3](https://www.genecards.org/Search/Keyword?queryString=ITGA3) |
| ITGA5 | ECM receptor | 24286194 | [24286194](https://www.ncbi.nlm.nih.gov/pubmed/24286194) | [ITGA5](https://www.genecards.org/Search/Keyword?queryString=ITGA5) |
| ITGA6 | ECM receptor | 15679046 | [15679046](https://www.ncbi.nlm.nih.gov/pubmed/15679046) | [ITGA6](https://www.genecards.org/Search/Keyword?queryString=ITGA6) |
| ITGA7 | ECM receptor | 16293150 | [16293150](https://www.ncbi.nlm.nih.gov/pubmed/16293150) | [ITGA7](https://www.genecards.org/Search/Keyword?queryString=ITGA7) |
| ITGA8 | ECM receptor | 15579315 | [15579315](https://www.ncbi.nlm.nih.gov/pubmed/15579315) | [ITGA8](https://www.genecards.org/Search/Keyword?queryString=ITGA8) |
| ITGA9 | ECM receptor | 16005200 | [16005200](https://www.ncbi.nlm.nih.gov/pubmed/16005200) | [ITGA9](https://www.genecards.org/Search/Keyword?queryString=ITGA9) |
| ITGAM | ECM receptor | 7696347 | [7696347](https://www.ncbi.nlm.nih.gov/pubmed/7696347) | [ITGAM](https://www.genecards.org/Search/Keyword?queryString=ITGAM) |
| ITGAV | ECM receptor | 25663698 | [25663698](https://www.ncbi.nlm.nih.gov/pubmed/25663698) | [ITGAV](https://www.genecards.org/Search/Keyword?queryString=ITGAV) |
| ITGAX | ECM receptor | 22189006 | [22189006](https://www.ncbi.nlm.nih.gov/pubmed/22189006) | [ITGAX](https://www.genecards.org/Search/Keyword?queryString=ITGAX) |
| ITGB1 | ECM receptor | 22451694 | [22451694](https://www.ncbi.nlm.nih.gov/pubmed/22451694) | [ITGB1](https://www.genecards.org/Search/Keyword?queryString=ITGB1) |
| ITGB2 | ECM receptor | 27218821 | [27218821](https://www.ncbi.nlm.nih.gov/pubmed/27218821) | [ITGB2](https://www.genecards.org/Search/Keyword?queryString=ITGB2) |
| ITGB3 | ECM receptor | 17175151 | [17175151](https://www.ncbi.nlm.nih.gov/pubmed/17175151) | [ITGB3](https://www.genecards.org/Search/Keyword?queryString=ITGB3) |
| ITGB5 | ECM receptor | 18550570 | [18550570](https://www.ncbi.nlm.nih.gov/pubmed/18550570) | [ITGB5](https://www.genecards.org/Search/Keyword?queryString=ITGB5) |
| ITGB6 | ECM receptor | 8120056 | [8120056](https://www.ncbi.nlm.nih.gov/pubmed/8120056) | [ITGB6](https://www.genecards.org/Search/Keyword?queryString=ITGB6) |
| ITIH1 | ECM Regulators | 22821669 | [22821669](https://www.ncbi.nlm.nih.gov/pubmed/22821669) | [ITIH1](https://www.genecards.org/Search/Keyword?queryString=ITIH1) |
| ITIH2 | ECM Regulators | 22821669 | [22821669](https://www.ncbi.nlm.nih.gov/pubmed/22821669) | [ITIH2](https://www.genecards.org/Search/Keyword?queryString=ITIH2) |
| ITIH3 | ECM Regulators | 18226209 | [18226209](https://www.ncbi.nlm.nih.gov/pubmed/18226209) | [ITIH3](https://www.genecards.org/Search/Keyword?queryString=ITIH3) |
| ITIH4 | ECM Regulators | 18226209 | [18226209](https://www.ncbi.nlm.nih.gov/pubmed/18226209) | [ITIH4](https://www.genecards.org/Search/Keyword?queryString=ITIH4) |
| ITIH5 | ECM Regulators | 18226209 | [18226209](https://www.ncbi.nlm.nih.gov/pubmed/18226209) | [ITIH5](https://www.genecards.org/Search/Keyword?queryString=ITIH5) |
| ITIH6 | ECM Regulators | 18226209 | [18226209](https://www.ncbi.nlm.nih.gov/pubmed/18226209) | [ITIH6](https://www.genecards.org/Search/Keyword?queryString=ITIH6) |
| ITLN1 | ECM-affiliated Proteins | 25965823 | [25965823](https://www.ncbi.nlm.nih.gov/pubmed/25965823) | [ITLN1](https://www.genecards.org/Search/Keyword?queryString=ITLN1) |
| ITLN2 | ECM-affiliated Proteins | 22159717 | [22159717](https://www.ncbi.nlm.nih.gov/pubmed/ 22159717  ) | [ITLN2](https://www.genecards.org/Search/Keyword?queryString=ITLN2) |
| ITPR1 | ECM Micro-enviornment associated | 7945203 | [7945203](https://www.ncbi.nlm.nih.gov/pubmed/7945203) | [ITPR1](https://www.genecards.org/Search/Keyword?queryString=ITPR1) |
| ITPR2 | ECM Micro-enviornment associated | 7945203 | [7945203](https://www.ncbi.nlm.nih.gov/pubmed/7945203) | [ITPR2](https://www.genecards.org/Search/Keyword?queryString=ITPR2) |
| ITPR3 | ECM Micro-enviornment associated | 7945203 | [7945203](https://www.ncbi.nlm.nih.gov/pubmed/7945203) | [ITPR3](https://www.genecards.org/Search/Keyword?queryString=ITPR3) |
| JPH3 | ECM Micro-enviornment associated | 11145944 | [11145944](https://www.ncbi.nlm.nih.gov/pubmed/11145944) | [JPH3](https://www.genecards.org/Search/Keyword?queryString=JPH3) |
| JPH4 | ECM Micro-enviornment associated | 11114299 | [11114299](https://www.ncbi.nlm.nih.gov/pubmed/11114299) | [JPH4](https://www.genecards.org/Search/Keyword?queryString=JPH4) |
| JUN | ECM associated regulatory factors | 25533033 | [25533033](https://www.ncbi.nlm.nih.gov/pubmed/25533033) | [JUN](https://www.genecards.org/Search/Keyword?queryString=JUN) |
| KAL1 | ECM Glycoproteins | 24084442 | [24084442](https://www.ncbi.nlm.nih.gov/pubmed/24084442) | [KAL1](https://www.genecards.org/Search/Keyword?queryString=KAL1) |
| KAZALD1 | ECM Regulators | 26290530 | [26290530](https://www.ncbi.nlm.nih.gov/pubmed/26290530) | [KAZALD1](https://www.genecards.org/Search/Keyword?queryString=KAZALD1) |
| KCP | ECM Glycoproteins | 21937732 | [21937732](https://www.ncbi.nlm.nih.gov/pubmed/21937732) | [KCP](https://www.genecards.org/Search/Keyword?queryString=KCP) |
| KDR | ECM associated regulatory factors | 12918061 | [12918061](https://www.ncbi.nlm.nih.gov/pubmed/12918061) | [KDR](https://www.genecards.org/Search/Keyword?queryString=KDR) |
| KERA | Proteoglycans | 23419153 | [23419153](https://www.ncbi.nlm.nih.gov/pubmed/23419153) | [KERA](https://www.genecards.org/Search/Keyword?queryString=KERA) |
| KITLG | Secreted Factors | 25912341 | [25912341](https://www.ncbi.nlm.nih.gov/pubmed/25912341) | [KITLG](https://www.genecards.org/Search/Keyword?queryString=KITLG) |
| KNG1 | ECM Regulators | 22159717 | [22159717](https://www.ncbi.nlm.nih.gov/pubmed/ 22159717  ) | [KNG1](https://www.genecards.org/Search/Keyword?queryString=KNG1) |
| KRAS | ECM Micro-enviornment associated | 19320777 | [19320777](https://www.ncbi.nlm.nih.gov/pubmed/19320777) | [KRAS](https://www.genecards.org/Search/Keyword?queryString=KRAS) |
| KRT15 | ECM | 15009727 | [15009727](https://www.ncbi.nlm.nih.gov/pubmed/15009727) | [KRT15](https://www.genecards.org/Search/Keyword?queryString=KRT15) |
| KY | ECM Regulators | 22159717 | [22159717](https://www.ncbi.nlm.nih.gov/pubmed/ 22159717  ) | [KY](https://www.genecards.org/Search/Keyword?queryString=KY) |
| L1CAM | ECM Micro-enviornment associated | 22973895 | [22973895](https://www.ncbi.nlm.nih.gov/pubmed/22973895) | [L1CAM](https://www.genecards.org/Search/Keyword?queryString=L1CAM) |
| LAMA1 | ECM Glycoproteins | 23419153 | [23419153](https://www.ncbi.nlm.nih.gov/pubmed/23419153) | [LAMA1](https://www.genecards.org/Search/Keyword?queryString=LAMA1) |
| LAMA2 | ECM Glycoproteins | 23419153 | [23419153](https://www.ncbi.nlm.nih.gov/pubmed/23419153) | [LAMA2](https://www.genecards.org/Search/Keyword?queryString=LAMA2) |
| LAMA3 | ECM Glycoproteins | 23419153 | [23419153](https://www.ncbi.nlm.nih.gov/pubmed/23419153) | [LAMA3](https://www.genecards.org/Search/Keyword?queryString=LAMA3) |
| LAMA4 | ECM Glycoproteins | 23419153 | [23419153](https://www.ncbi.nlm.nih.gov/pubmed/23419153) | [LAMA4](https://www.genecards.org/Search/Keyword?queryString=LAMA4) |
| LAMA5 | ECM Glycoproteins | 23419153 | [23419153](https://www.ncbi.nlm.nih.gov/pubmed/23419153) | [LAMA5](https://www.genecards.org/Search/Keyword?queryString=LAMA5) |
| LAMB1 | ECM Glycoproteins | 23419153 | [23419153](https://www.ncbi.nlm.nih.gov/pubmed/23419153) | [LAMB1](https://www.genecards.org/Search/Keyword?queryString=LAMB1) |
| LAMB2 | ECM Glycoproteins | 23419153 | [23419153](https://www.ncbi.nlm.nih.gov/pubmed/23419153) | [LAMB2](https://www.genecards.org/Search/Keyword?queryString=LAMB2) |
| LAMB3 | ECM Glycoproteins | 23419153 | [23419153](https://www.ncbi.nlm.nih.gov/pubmed/23419153) | [LAMB3](https://www.genecards.org/Search/Keyword?queryString=LAMB3) |
| LAMB4 | ECM Glycoproteins | 23419153 | [23419153](https://www.ncbi.nlm.nih.gov/pubmed/23419153) | [LAMB4](https://www.genecards.org/Search/Keyword?queryString=LAMB4) |
| LAMC1 | ECM Glycoproteins | 23419153 | [23419153](https://www.ncbi.nlm.nih.gov/pubmed/23419153) | [LAMC1](https://www.genecards.org/Search/Keyword?queryString=LAMC1) |
| LAMC2 | ECM Glycoproteins | 23419153 | [23419153](https://www.ncbi.nlm.nih.gov/pubmed/23419153) | [LAMC2](https://www.genecards.org/Search/Keyword?queryString=LAMC2) |
| LAMC3 | ECM Glycoproteins | 23419153 | [23419153](https://www.ncbi.nlm.nih.gov/pubmed/23419153) | [LAMC3](https://www.genecards.org/Search/Keyword?queryString=LAMC3) |
| LDLR | ECM Micro-enviornment associated | 12598530 | [12598530](https://www.ncbi.nlm.nih.gov/pubmed/12598530) | [LDLR](https://www.genecards.org/Search/Keyword?queryString=LDLR) |
| LEFTY1 | Secreted Factors | 25037231 | [25037231](https://www.ncbi.nlm.nih.gov/pubmed/25037231) | [LEFTY1](https://www.genecards.org/Search/Keyword?queryString=LEFTY1) |
| LEFTY2 | Secreted Factors | 24147624 | [24147624](https://www.ncbi.nlm.nih.gov/pubmed/24147624) | [LEFTY2](https://www.genecards.org/Search/Keyword?queryString=LEFTY2) |
| LEP | Secreted Factors | 19391127 | [19391127](https://www.ncbi.nlm.nih.gov/pubmed/19391127) | [LEP](https://www.genecards.org/Search/Keyword?queryString=LEP) |
| LEPRE1 | ECM Regulators | 19862557 | [19862557](https://www.ncbi.nlm.nih.gov/pubmed/19862557) | [LEPRE1](https://www.genecards.org/Search/Keyword?queryString=LEPRE1) |
| LEPREL1 | ECM Regulators | 25645914 | [25645914](https://www.ncbi.nlm.nih.gov/pubmed/25645914) | [LEPREL1](https://www.genecards.org/Search/Keyword?queryString=LEPREL1) |
| LEPREL2 | ECM Regulators | 19436308 | [19436308](https://www.ncbi.nlm.nih.gov/pubmed/19436308) | [LEPREL2](https://www.genecards.org/Search/Keyword?queryString=LEPREL2) |
| LGALS1 | ECM-affiliated Proteins | 16840800 | [16840800](https://www.ncbi.nlm.nih.gov/pubmed/16840800) | [LGALS1](https://www.genecards.org/Search/Keyword?queryString=LGALS1) |
| LGALS12 | ECM-affiliated Proteins | 22159717 | [22159717](https://www.ncbi.nlm.nih.gov/pubmed/ 22159717  ) | [LGALS12](https://www.genecards.org/Search/Keyword?queryString=LGALS12) |
| LGALS13 | ECM-affiliated Proteins | 22159717 | [22159717](https://www.ncbi.nlm.nih.gov/pubmed/ 22159717  ) | [LGALS13](https://www.genecards.org/Search/Keyword?queryString=LGALS13) |
| LGALS14 | ECM-affiliated Proteins | 22159717 | [22159717](https://www.ncbi.nlm.nih.gov/pubmed/ 22159717  ) | [LGALS14](https://www.genecards.org/Search/Keyword?queryString=LGALS14) |
| LGALS16 | ECM-affiliated Proteins | 22159717 | [22159717](https://www.ncbi.nlm.nih.gov/pubmed/ 22159717  ) | [LGALS16](https://www.genecards.org/Search/Keyword?queryString=LGALS16) |
| LGALS2 | ECM-affiliated Proteins | 22159717 | [22159717](https://www.ncbi.nlm.nih.gov/pubmed/ 22159717  ) | [LGALS2](https://www.genecards.org/Search/Keyword?queryString=LGALS2) |
| LGALS3 | ECM-affiliated Proteins | 22159717 | [22159717](https://www.ncbi.nlm.nih.gov/pubmed/ 22159717  ) | [LGALS3](https://www.genecards.org/Search/Keyword?queryString=LGALS3) |
| LGALS4 | ECM-affiliated Proteins | 22159717 | [22159717](https://www.ncbi.nlm.nih.gov/pubmed/ 22159717  ) | [LGALS4](https://www.genecards.org/Search/Keyword?queryString=LGALS4) |
| LGALS7 | ECM-affiliated Proteins | 22159717 | [22159717](https://www.ncbi.nlm.nih.gov/pubmed/ 22159717  ) | [LGALS7](https://www.genecards.org/Search/Keyword?queryString=LGALS7) |
| LGALS8 | ECM-affiliated Proteins | 22159717 | [22159717](https://www.ncbi.nlm.nih.gov/pubmed/ 22159717  ) | [LGALS8](https://www.genecards.org/Search/Keyword?queryString=LGALS8) |
| LGALS9 | ECM-affiliated Proteins | 22159717 | [22159717](https://www.ncbi.nlm.nih.gov/pubmed/ 22159717  ) | [LGALS9](https://www.genecards.org/Search/Keyword?queryString=LGALS9) |
| LGALS9B | ECM-affiliated Proteins | 22159717 | [22159717](https://www.ncbi.nlm.nih.gov/pubmed/ 22159717  ) | [LGALS9B](https://www.genecards.org/Search/Keyword?queryString=LGALS9B) |
| LGALS9C | ECM-affiliated Proteins | 22159717 | [22159717](https://www.ncbi.nlm.nih.gov/pubmed/ 22159717  ) | [LGALS9C](https://www.genecards.org/Search/Keyword?queryString=LGALS9C) |
| LGI1 | ECM Glycoproteins | 23419153 | [23419153](https://www.ncbi.nlm.nih.gov/pubmed/23419153) | [LGI1](https://www.genecards.org/Search/Keyword?queryString=LGI1) |
| LGI2 | ECM Glycoproteins | 23419153 | [23419153](https://www.ncbi.nlm.nih.gov/pubmed/23419153) | [LGI2](https://www.genecards.org/Search/Keyword?queryString=LGI2) |
| LGI3 | ECM Glycoproteins | 23419153 | [23419153](https://www.ncbi.nlm.nih.gov/pubmed/23419153) | [LGI3](https://www.genecards.org/Search/Keyword?queryString=LGI3) |
| LGI4 | ECM Glycoproteins | 23419153 | [23419153](https://www.ncbi.nlm.nih.gov/pubmed/23419153) | [LGI4](https://www.genecards.org/Search/Keyword?queryString=LGI4) |
| LIF | Secreted Factors | 8335694 | [8335694](https://www.ncbi.nlm.nih.gov/pubmed/8335694) | [LIF](https://www.genecards.org/Search/Keyword?queryString=LIF) |
| LIPC | ECM Micro-enviornment associated | 9395455 | [9395455](https://www.ncbi.nlm.nih.gov/pubmed/9395455) | [LIPC](https://www.genecards.org/Search/Keyword?queryString=LIPC) |
| LIPE | ECM Micro-enviornment associated | 1714464 | [1714464](https://www.ncbi.nlm.nih.gov/pubmed/1714464) | [LIPE](https://www.genecards.org/Search/Keyword?queryString=LIPE) |
| LMAN1 | ECM-affiliated Proteins | 26150355 | [26150355](https://www.ncbi.nlm.nih.gov/pubmed/26150355) | [LMAN1](https://www.genecards.org/Search/Keyword?queryString=LMAN1) |
| LMAN1L | ECM-affiliated Proteins | 28675934 | [28675934](https://www.ncbi.nlm.nih.gov/pubmed/28675934) | [LMAN1L](https://www.genecards.org/Search/Keyword?queryString=LMAN1L) |
| LONP1 | ECM Micro-enviornment associated | 17498058 | [17498058](https://www.ncbi.nlm.nih.gov/pubmed/17498058) | [LONP1](https://www.genecards.org/Search/Keyword?queryString=LONP1) |
| LOX | ECM Regulators | 24348049 | [24348049](https://www.ncbi.nlm.nih.gov/pubmed/24348049) | [LOX](https://www.genecards.org/Search/Keyword?queryString=LOX) |
| LOXL1 | ECM Regulators | 24348049 | [24348049](https://www.ncbi.nlm.nih.gov/pubmed/24348049) | [LOXL1](https://www.genecards.org/Search/Keyword?queryString=LOXL1) |
| LOXL2 | ECM Regulators | 24348049 | [24348049](https://www.ncbi.nlm.nih.gov/pubmed/24348049) | [LOXL2](https://www.genecards.org/Search/Keyword?queryString=LOXL2) |
| LOXL3 | ECM Regulators | 24348049 | [24348049](https://www.ncbi.nlm.nih.gov/pubmed/24348049) | [LOXL3](https://www.genecards.org/Search/Keyword?queryString=LOXL3) |
| LOXL4 | ECM Regulators | 24348049 | [24348049](https://www.ncbi.nlm.nih.gov/pubmed/24348049) | [LOXL4](https://www.genecards.org/Search/Keyword?queryString=LOXL4) |
| LPA | ECM Regulators | 29677358 | [29677358](https://www.ncbi.nlm.nih.gov/pubmed/29677358) | [LPA](https://www.genecards.org/Search/Keyword?queryString=LPA) |
| LPL | ECM Micro-enviornment associated | 11212344 | [11212344](https://www.ncbi.nlm.nih.gov/pubmed/11212344) | [LPL](https://www.genecards.org/Search/Keyword?queryString=LPL) |
| LRG1 | ECM Glycoproteins | 23419153 | [23419153](https://www.ncbi.nlm.nih.gov/pubmed/23419153) | [LRG1](https://www.genecards.org/Search/Keyword?queryString=LRG1) |
| LRP1 | ECM Micro-enviornment associated | 21289173 | [21289173](https://www.ncbi.nlm.nih.gov/pubmed/21289173) | [LRP1](https://www.genecards.org/Search/Keyword?queryString=LRP1) |
| LRP4 | ECM Micro-enviornment associated | 21471202 | [21471202](https://www.ncbi.nlm.nih.gov/pubmed/21471202) | [LRP4](https://www.genecards.org/Search/Keyword?queryString=LRP4) |
| LRPAP1 | ECM Micro-enviornment associated | 21289173 | [21289173](https://www.ncbi.nlm.nih.gov/pubmed/21289173) | [LRPAP1](https://www.genecards.org/Search/Keyword?queryString=LRPAP1) |
| LRRTM4 | ECM Micro-enviornment associated | 23911103 | [23911103](https://www.ncbi.nlm.nih.gov/pubmed/23911103) | [LRRTM4](https://www.genecards.org/Search/Keyword?queryString=LRRTM4) |
| LTA | Secreted Factors | 22159717 | [22159717](https://www.ncbi.nlm.nih.gov/pubmed/ 22159717  ) | [LTA](https://www.genecards.org/Search/Keyword?queryString=LTA) |
| LTB | Secreted Factors | 22159717 | [22159717](https://www.ncbi.nlm.nih.gov/pubmed/ 22159717  ) | [LTB](https://www.genecards.org/Search/Keyword?queryString=LTB) |
| LTBP1 | ECM Glycoproteins | 23419153 | [23419153](https://www.ncbi.nlm.nih.gov/pubmed/23419153) | [LTBP1](https://www.genecards.org/Search/Keyword?queryString=LTBP1) |
| LTBP2 | ECM Glycoproteins | 23419153 | [23419153](https://www.ncbi.nlm.nih.gov/pubmed/23419153) | [LTBP2](https://www.genecards.org/Search/Keyword?queryString=LTBP2) |
| LTBP3 | ECM Glycoproteins | 23419153 | [23419153](https://www.ncbi.nlm.nih.gov/pubmed/23419153) | [LTBP3](https://www.genecards.org/Search/Keyword?queryString=LTBP3) |
| LTBP4 | ECM Glycoproteins | 23419153 | [23419153](https://www.ncbi.nlm.nih.gov/pubmed/23419153) | [LTBP4](https://www.genecards.org/Search/Keyword?queryString=LTBP4) |
| LTF | ECM | 16337314 | [16337314](https://www.ncbi.nlm.nih.gov/pubmed/16337314) | [LTF](https://www.genecards.org/Search/Keyword?queryString=LTF) |
| LUM | Proteoglycans | 24060754 | [24060754](https://www.ncbi.nlm.nih.gov/pubmed/24060754) | [LUM](https://www.genecards.org/Search/Keyword?queryString=LUM) |
| M6PR | ECM Micro-enviornment associated | 16046412 | [16046412](https://www.ncbi.nlm.nih.gov/pubmed/16046412) | [M6PR](https://www.genecards.org/Search/Keyword?queryString=M6PR) |
| MAG | Cytoskeleton | 7542351 | [7542351](https://www.ncbi.nlm.nih.gov/pubmed/7542351) | [MAG](https://www.genecards.org/Search/Keyword?queryString=MAG) |
| MAMDC2 | ECM Micro-enviornment associated | 28411251 | [28411251](https://www.ncbi.nlm.nih.gov/pubmed/28411251) | [MAMDC2](https://www.genecards.org/Search/Keyword?queryString=MAMDC2) |
| MAP2 | Cytoskeleton | 1647395 | [1647395](https://www.ncbi.nlm.nih.gov/pubmed/1647395) | [MAP2](https://www.genecards.org/Search/Keyword?queryString=MAP2) |
| MAP2K1 | ECM Micro-enviornment associated | 10753939 | [10753939](https://www.ncbi.nlm.nih.gov/pubmed/10753939) | [MAP2K1](https://www.genecards.org/Search/Keyword?queryString=MAP2K1) |
| MAP2K2 | ECM Micro-enviornment associated | 21615688 | [21615688](https://www.ncbi.nlm.nih.gov/pubmed/21615688) | [MAP2K2](https://www.genecards.org/Search/Keyword?queryString=MAP2K2) |
| MAPK1 | ECM Micro-enviornment associated | 19287189 | [19287189](https://www.ncbi.nlm.nih.gov/pubmed/19287189) | [MAPK1](https://www.genecards.org/Search/Keyword?queryString=MAPK1) |
| MAPK10 | ECM Micro-enviornment associated | 17656145 | [17656145](https://www.ncbi.nlm.nih.gov/pubmed/17656145) | [MAPK10](https://www.genecards.org/Search/Keyword?queryString=MAPK10) |
| MAPK11 | ECM Micro-enviornment associated | 10744763 | [10744763](https://www.ncbi.nlm.nih.gov/pubmed/10744763) | [MAPK11](https://www.genecards.org/Search/Keyword?queryString=MAPK11) |
| MAPK12 | ECM Micro-enviornment associated | 20231272 | [20231272](https://www.ncbi.nlm.nih.gov/pubmed/20231272) | [MAPK12](https://www.genecards.org/Search/Keyword?queryString=MAPK12) |
| MAPK13 | ECM Micro-enviornment associated | 12244047 | [12244047](https://www.ncbi.nlm.nih.gov/pubmed/12244047) | [MAPK13](https://www.genecards.org/Search/Keyword?queryString=MAPK13) |
| MAPK14 | ECM Micro-enviornment associated | 14766231 | [14766231](https://www.ncbi.nlm.nih.gov/pubmed/14766231) | [MAPK14](https://www.genecards.org/Search/Keyword?queryString=MAPK14) |
| MAPK3 | ECM Micro-enviornment associated | 10431817 | [10431817](https://www.ncbi.nlm.nih.gov/pubmed/10431817) | [MAPK3](https://www.genecards.org/Search/Keyword?queryString=MAPK3) |
| MAPK7 | ECM Micro-enviornment associated | 26617753 | [26617753](https://www.ncbi.nlm.nih.gov/pubmed/26617753) | [MAPK7](https://www.genecards.org/Search/Keyword?queryString=MAPK7) |
| MAPK8 | ECM Micro-enviornment associated | 26262877 | [26262877](https://www.ncbi.nlm.nih.gov/pubmed/26262877) | [MAPK8](https://www.genecards.org/Search/Keyword?queryString=MAPK8) |
| MASP1 | ECM Regulators | 23841802 | [23841802](https://www.ncbi.nlm.nih.gov/pubmed/23841802) | [MASP1](https://www.genecards.org/Search/Keyword?queryString=MASP1) |
| MASP2 | ECM Regulators | 25038892 | [25038892](https://www.ncbi.nlm.nih.gov/pubmed/25038892) | [MASP2](https://www.genecards.org/Search/Keyword?queryString=MASP2) |
| MATN1 | ECM Glycoproteins | 23419153 | [23419153](https://www.ncbi.nlm.nih.gov/pubmed/23419153) | [MATN1](https://www.genecards.org/Search/Keyword?queryString=MATN1) |
| MATN2 | ECM Glycoproteins | 23419153 | [23419153](https://www.ncbi.nlm.nih.gov/pubmed/23419153) | [MATN2](https://www.genecards.org/Search/Keyword?queryString=MATN2) |
| MATN3 | ECM Glycoproteins | 23419153 | [23419153](https://www.ncbi.nlm.nih.gov/pubmed/23419153) | [MATN3](https://www.genecards.org/Search/Keyword?queryString=MATN3) |
| MATN4 | ECM Glycoproteins | 23419153 | [23419153](https://www.ncbi.nlm.nih.gov/pubmed/23419153) | [MATN4](https://www.genecards.org/Search/Keyword?queryString=MATN4) |
| MBL2 | ECM-affiliated Proteins | 25038892 | [25038892](https://www.ncbi.nlm.nih.gov/pubmed/25038892) | [MBL2](https://www.genecards.org/Search/Keyword?queryString=MBL2) |
| MBL3P | ECM Micro-enviornment associated | 16177119 | [16177119](https://www.ncbi.nlm.nih.gov/pubmed/16177119) | [MBL3P](https://www.genecards.org/Search/Keyword?queryString=MBL3P) |
| MDK | Secreted Factors | 24102259 | [24102259](https://www.ncbi.nlm.nih.gov/pubmed/24102259) | [MDK](https://www.genecards.org/Search/Keyword?queryString=MDK) |
| MDM2 | ECM Micro-enviornment associated | 8883415 | [8883415](https://www.ncbi.nlm.nih.gov/pubmed/8883415) | [MDM2](https://www.genecards.org/Search/Keyword?queryString=MDM2) |
| MEGF10 | Secreted Factors | 18056409 | [18056409](https://www.ncbi.nlm.nih.gov/pubmed/18056409) | [MEGF10](https://www.genecards.org/Search/Keyword?queryString=MEGF10) |
| MEGF11 | Secreted Factors | 22159717 | [22159717](https://www.ncbi.nlm.nih.gov/pubmed/ 22159717  ) | [MEGF11](https://www.genecards.org/Search/Keyword?queryString=MEGF11) |
| MEGF6 | Secreted Factors | 22159717 | [22159717](https://www.ncbi.nlm.nih.gov/pubmed/ 22159717  ) | [MEGF6](https://www.genecards.org/Search/Keyword?queryString=MEGF6) |
| MEGF8 | Secreted Factors | 22159717 | [22159717](https://www.ncbi.nlm.nih.gov/pubmed/ 22159717  ) | [MEGF8](https://www.genecards.org/Search/Keyword?queryString=MEGF8) |
| MEGF9 | Secreted Factors | 22159717 | [22159717](https://www.ncbi.nlm.nih.gov/pubmed/ 22159717  ) | [MEGF9](https://www.genecards.org/Search/Keyword?queryString=MEGF9) |
| MEP1A | ECM Regulators | 7774936 | [7774936](https://www.ncbi.nlm.nih.gov/pubmed/7774936) | [MEP1A](https://www.genecards.org/Search/Keyword?queryString=MEP1A) |
| MEP1B | ECM Regulators | 7774936 | [7774936](https://www.ncbi.nlm.nih.gov/pubmed/7774936) | [MEP1B](https://www.genecards.org/Search/Keyword?queryString=MEP1B) |
| MEPE | ECM Glycoproteins | 12646701 | [12646701](https://www.ncbi.nlm.nih.gov/pubmed/12646701) | [MEPE](https://www.genecards.org/Search/Keyword?queryString=MEPE) |
| MET | ECM Micro-enviornment associated | 11073815 | [11073815](https://www.ncbi.nlm.nih.gov/pubmed/11073815) | [MET](https://www.genecards.org/Search/Keyword?queryString=MET) |
| MFAP1 | ECM Glycoproteins | 23419153 | [23419153](https://www.ncbi.nlm.nih.gov/pubmed/23419153) | [MFAP1](https://www.genecards.org/Search/Keyword?queryString=MFAP1) |
| MFAP2 | ECM Glycoproteins | 23419153 | [23419153](https://www.ncbi.nlm.nih.gov/pubmed/23419153) | [MFAP2](https://www.genecards.org/Search/Keyword?queryString=MFAP2) |
| MFAP3 | ECM Glycoproteins | 23419153 | [23419153](https://www.ncbi.nlm.nih.gov/pubmed/23419153) | [MFAP3](https://www.genecards.org/Search/Keyword?queryString=MFAP3) |
| MFAP4 | ECM Glycoproteins | 23419153 | [23419153](https://www.ncbi.nlm.nih.gov/pubmed/23419153) | [MFAP4](https://www.genecards.org/Search/Keyword?queryString=MFAP4) |
| MFAP5 | ECM Glycoproteins | 23419153 | [23419153](https://www.ncbi.nlm.nih.gov/pubmed/23419153) | [MFAP5](https://www.genecards.org/Search/Keyword?queryString=MFAP5) |
| MFGE8 | ECM Glycoproteins | 23419153 | [23419153](https://www.ncbi.nlm.nih.gov/pubmed/23419153) | [MFGE8](https://www.genecards.org/Search/Keyword?queryString=MFGE8) |
| MGP | ECM Glycoproteins | 23419153 | [23419153](https://www.ncbi.nlm.nih.gov/pubmed/23419153) | [MGP](https://www.genecards.org/Search/Keyword?queryString=MGP) |
| MMP1 | ECM Regulators | 10970876 | [10970876](https://www.ncbi.nlm.nih.gov/pubmed/10970876) | [MMP1](https://www.genecards.org/Search/Keyword?queryString=MMP1) |
| MMP10 | ECM Regulators | 10970876 | [10970876](https://www.ncbi.nlm.nih.gov/pubmed/10970876) | [MMP10](https://www.genecards.org/Search/Keyword?queryString=MMP10) |
| MMP11 | ECM Regulators | 10970876 | [10970876](https://www.ncbi.nlm.nih.gov/pubmed/10970876) | [MMP11](https://www.genecards.org/Search/Keyword?queryString=MMP11) |
| MMP12 | ECM Regulators | 10970876 | [10970876](https://www.ncbi.nlm.nih.gov/pubmed/10970876) | [MMP12](https://www.genecards.org/Search/Keyword?queryString=MMP12) |
| MMP13 | ECM Regulators | 10970876 | [10970876](https://www.ncbi.nlm.nih.gov/pubmed/10970876) | [MMP13](https://www.genecards.org/Search/Keyword?queryString=MMP13) |
| MMP14 | ECM Regulators | 10970876 | [10970876](https://www.ncbi.nlm.nih.gov/pubmed/10970876) | [MMP14](https://www.genecards.org/Search/Keyword?queryString=MMP14) |
| MMP15 | ECM Regulators | 10970876 | [10970876](https://www.ncbi.nlm.nih.gov/pubmed/10970876) | [MMP15](https://www.genecards.org/Search/Keyword?queryString=MMP15) |
| MMP16 | ECM Regulators | 10970876 | [10970876](https://www.ncbi.nlm.nih.gov/pubmed/10970876) | [MMP16](https://www.genecards.org/Search/Keyword?queryString=MMP16) |
| MMP17 | ECM Regulators | 10970876 | [10970876](https://www.ncbi.nlm.nih.gov/pubmed/10970876) | [MMP17](https://www.genecards.org/Search/Keyword?queryString=MMP17) |
| MMP19 | ECM Regulators | 10970876 | [10970876](https://www.ncbi.nlm.nih.gov/pubmed/10970876) | [MMP19](https://www.genecards.org/Search/Keyword?queryString=MMP19) |
| MMP2 | ECM Regulators | 10970876 | [10970876](https://www.ncbi.nlm.nih.gov/pubmed/10970876) | [MMP2](https://www.genecards.org/Search/Keyword?queryString=MMP2) |
| MMP20 | ECM Regulators | 10970876 | [10970876](https://www.ncbi.nlm.nih.gov/pubmed/10970876) | [MMP20](https://www.genecards.org/Search/Keyword?queryString=MMP20) |
| MMP21 | ECM Regulators | 10970876 | [10970876](https://www.ncbi.nlm.nih.gov/pubmed/10970876) | [MMP21](https://www.genecards.org/Search/Keyword?queryString=MMP21) |
| MMP23B | ECM Regulators | 21064033 | [21064033](https://www.ncbi.nlm.nih.gov/pubmed/21064033) | [MMP23B](https://www.genecards.org/Search/Keyword?queryString=MMP23B) |
| MMP24 | ECM Regulators | 10970876 | [10970876](https://www.ncbi.nlm.nih.gov/pubmed/10970876) | [MMP24](https://www.genecards.org/Search/Keyword?queryString=MMP24) |
| MMP25 | ECM Regulators | 10970876 | [10970876](https://www.ncbi.nlm.nih.gov/pubmed/10970876) | [MMP25](https://www.genecards.org/Search/Keyword?queryString=MMP25) |
| MMP26 | ECM Regulators | 10970876 | [10970876](https://www.ncbi.nlm.nih.gov/pubmed/10970876) | [MMP26](https://www.genecards.org/Search/Keyword?queryString=MMP26) |
| MMP27 | ECM Regulators | 10970876 | [10970876](https://www.ncbi.nlm.nih.gov/pubmed/10970876) | [MMP27](https://www.genecards.org/Search/Keyword?queryString=MMP27) |
| MMP28 | ECM Regulators | 22159717 | [22159717](https://www.ncbi.nlm.nih.gov/pubmed/ 22159717  ) | [MMP28](https://www.genecards.org/Search/Keyword?queryString=MMP28) |
| MMP3 | ECM Regulators | 10970876 | [10970876](https://www.ncbi.nlm.nih.gov/pubmed/10970876) | [MMP3](https://www.genecards.org/Search/Keyword?queryString=MMP3) |
| MMP7 | ECM Regulators | 10970876 | [10970876](https://www.ncbi.nlm.nih.gov/pubmed/10970876) | [MMP7](https://www.genecards.org/Search/Keyword?queryString=MMP7) |
| MMP8 | ECM Regulators | 10970876 | [10970876](https://www.ncbi.nlm.nih.gov/pubmed/10970876) | [MMP8](https://www.genecards.org/Search/Keyword?queryString=MMP8) |
| MMP9 | ECM Regulators | 10970876 | [10970876](https://www.ncbi.nlm.nih.gov/pubmed/10970876) | [MMP9](https://www.genecards.org/Search/Keyword?queryString=MMP9) |
| MMRN1 | ECM Glycoproteins | 23419153 | [23419153](https://www.ncbi.nlm.nih.gov/pubmed/23419153) | [MMRN1](https://www.genecards.org/Search/Keyword?queryString=MMRN1) |
| MMRN2 | ECM Glycoproteins | 23419153 | [23419153](https://www.ncbi.nlm.nih.gov/pubmed/23419153) | [MMRN2](https://www.genecards.org/Search/Keyword?queryString=MMRN2) |
| MMVP2 | ECM Micro-enviornment associated | 29352010 | [29352010](https://www.ncbi.nlm.nih.gov/pubmed/29352010) | [MMVP2](https://www.genecards.org/Search/Keyword?queryString=MMVP2) |
| MPDZ | ECM Micro-enviornment associated | 10967549 | [10967549](https://www.ncbi.nlm.nih.gov/pubmed/10967549) | [MPDZ](https://www.genecards.org/Search/Keyword?queryString=MPDZ) |
| MPO | ECM Micro-enviornment associated | 19788922 | [19788922](https://www.ncbi.nlm.nih.gov/pubmed/19788922) | [MPO](https://www.genecards.org/Search/Keyword?queryString=MPO) |
| MRAS | ECM Micro-enviornment associated | 27624141 | [27624141](https://www.ncbi.nlm.nih.gov/pubmed/27624141) | [MRAS](https://www.genecards.org/Search/Keyword?queryString=MRAS) |
| MRGPRX1 | ECM Micro-enviornment associated | 12714503 | [12714503](https://www.ncbi.nlm.nih.gov/pubmed/12714503) | [MRGPRX1](https://www.genecards.org/Search/Keyword?queryString=MRGPRX1) |
| MSN | Cytoskeleton | 10462524 | [10462524](https://www.ncbi.nlm.nih.gov/pubmed/10462524) | [MSN](https://www.genecards.org/Search/Keyword?queryString=MSN) |
| MSR1 | ECM Micro-enviornment associated | 12488451 | [12488451](https://www.ncbi.nlm.nih.gov/pubmed/12488451) | [MSR1](https://www.genecards.org/Search/Keyword?queryString=MSR1) |
| MST1 | Secreted Factors | 27765911 | [27765911](https://www.ncbi.nlm.nih.gov/pubmed/27765911) | [MST1](https://www.genecards.org/Search/Keyword?queryString=MST1) |
| MST1L | Secreted Factors | 22159717 | [22159717](https://www.ncbi.nlm.nih.gov/pubmed/22159717   ) | [MST1L](https://www.genecards.org/Search/Keyword?queryString=MST1L) |
| MSTN | Secreted Factors | 28012893 | [28012893](https://www.ncbi.nlm.nih.gov/pubmed/28012893) | [MSTN](https://www.genecards.org/Search/Keyword?queryString=MSTN) |
| MT3 | ECM Micro-enviornment associated | 16766169 | [16766169](https://www.ncbi.nlm.nih.gov/pubmed/16766169) | [MT3](https://www.genecards.org/Search/Keyword?queryString=MT3) |
| MTOR | ECM Micro-enviornment associated | 18434090 | [18434090](https://www.ncbi.nlm.nih.gov/pubmed/18434090) | [MTOR](https://www.genecards.org/Search/Keyword?queryString=MTOR) |
| MUC1 | ECM-affiliated Proteins | 23419153 | [23419153](https://www.ncbi.nlm.nih.gov/pubmed/23419153) | [MUC1](https://www.genecards.org/Search/Keyword?queryString=MUC1) |
| MUC12 | ECM-affiliated Proteins | 19904814 | [19904814](https://www.ncbi.nlm.nih.gov/pubmed/19904814) | [MUC12](https://www.genecards.org/Search/Keyword?queryString=MUC12) |
| MUC13 | ECM-affiliated Proteins | 19904814 | [19904814](https://www.ncbi.nlm.nih.gov/pubmed/19904814) | [MUC13](https://www.genecards.org/Search/Keyword?queryString=MUC13) |
| MUC15 | ECM-affiliated Proteins | 19904814 | [19904814](https://www.ncbi.nlm.nih.gov/pubmed/19904814) | [MUC15](https://www.genecards.org/Search/Keyword?queryString=MUC15) |
| MUC16 | ECM-affiliated Proteins | 23419153 | [23419153](https://www.ncbi.nlm.nih.gov/pubmed/23419153) | [MUC16](https://www.genecards.org/Search/Keyword?queryString=MUC16) |
| MUC17 | ECM-affiliated Proteins | 19904814 | [19904814](https://www.ncbi.nlm.nih.gov/pubmed/19904814) | [MUC17](https://www.genecards.org/Search/Keyword?queryString=MUC17) |
| MUC19 | ECM-affiliated Proteins | 19904814 | [19904814](https://www.ncbi.nlm.nih.gov/pubmed/19904814) | [MUC19](https://www.genecards.org/Search/Keyword?queryString=MUC19) |
| MUC2 | ECM-affiliated Proteins | 19904814 | [19904814](https://www.ncbi.nlm.nih.gov/pubmed/19904814) | [MUC2](https://www.genecards.org/Search/Keyword?queryString=MUC2) |
| MUC20 | ECM-affiliated Proteins | 19904814 | [19904814](https://www.ncbi.nlm.nih.gov/pubmed/19904814) | [MUC20](https://www.genecards.org/Search/Keyword?queryString=MUC20) |
| MUC21 | ECM-affiliated Proteins | 19904814 | [19904814](https://www.ncbi.nlm.nih.gov/pubmed/19904814) | [MUC21](https://www.genecards.org/Search/Keyword?queryString=MUC21) |
| MUC22 | ECM-affiliated Proteins | 24804170 | [24804170](https://www.ncbi.nlm.nih.gov/pubmed/24804170) | [MUC22](https://www.genecards.org/Search/Keyword?queryString=MUC22) |
| MUC3A | ECM-affiliated Proteins | 19904814 | [19904814](https://www.ncbi.nlm.nih.gov/pubmed/19904814) | [MUC3A](https://www.genecards.org/Search/Keyword?queryString=MUC3A) |
| MUC4 | ECM-affiliated Proteins | 23419153 | [23419153](https://www.ncbi.nlm.nih.gov/pubmed/23419153) | [MUC4](https://www.genecards.org/Search/Keyword?queryString=MUC4) |
| MUC5AC | ECM-affiliated Proteins | 23419153 | [23419153](https://www.ncbi.nlm.nih.gov/pubmed/23419153) | [MUC5AC](https://www.genecards.org/Search/Keyword?queryString=MUC5AC) |
| MUC5B | ECM-affiliated Proteins | 23419153 | [23419153](https://www.ncbi.nlm.nih.gov/pubmed/23419153) | [MUC5B](https://www.genecards.org/Search/Keyword?queryString=MUC5B) |
| MUC6 | ECM-affiliated Proteins | 19904814 | [19904814](https://www.ncbi.nlm.nih.gov/pubmed/19904814) | [MUC6](https://www.genecards.org/Search/Keyword?queryString=MUC6) |
| MUC7 | ECM-affiliated Proteins | 12646701 | [12646701](https://www.ncbi.nlm.nih.gov/pubmed/12646701) | [MUC7](https://www.genecards.org/Search/Keyword?queryString=MUC7) |
| MUC8 | ECM-affiliated Proteins | 19904814 | [19904814](https://www.ncbi.nlm.nih.gov/pubmed/19904814) | [MUC8](https://www.genecards.org/Search/Keyword?queryString=MUC8) |
| MUCL1 | ECM-affiliated Proteins | 23840300 | [23840300](https://www.ncbi.nlm.nih.gov/pubmed/23840300) | [MUCL1](https://www.genecards.org/Search/Keyword?queryString=MUCL1) |
| MUSK | ECM Micro-enviornment associated | 10366602 | [10366602](https://www.ncbi.nlm.nih.gov/pubmed/10366602) | [MUSK](https://www.genecards.org/Search/Keyword?queryString=MUSK) |
| MXRA5 | ECM Glycoproteins | 27599751 | [27599751](https://www.ncbi.nlm.nih.gov/pubmed/27599751) | [MXRA5](https://www.genecards.org/Search/Keyword?queryString=MXRA5) |
| MYC | ECM associated regulatory factors | 11311202 | [11311202](https://www.ncbi.nlm.nih.gov/pubmed/11311202) | [MYC](https://www.genecards.org/Search/Keyword?queryString=MYC) |
| MYD88 | ECM Micro-enviornment associated | 22095710 | [22095710](https://www.ncbi.nlm.nih.gov/pubmed/22095710) | [MYD88](https://www.genecards.org/Search/Keyword?queryString=MYD88) |
| MYDGF | ECM Micro-enviornment associated | 25581518 | [25581518](https://www.ncbi.nlm.nih.gov/pubmed/25581518) | [MYDGF](https://www.genecards.org/Search/Keyword?queryString=MYDGF) |
| NANOG | ECM associated regulatory factors | 22493428 | [22493428](https://www.ncbi.nlm.nih.gov/pubmed/22493428) | [NANOG](https://www.genecards.org/Search/Keyword?queryString=NANOG) |
| NCAM1 | ECM Micro-enviornment associated | 7513709 | [7513709](https://www.ncbi.nlm.nih.gov/pubmed/7513709) | [NCAM1](https://www.genecards.org/Search/Keyword?queryString=NCAM1) |
| NCAN | Proteoglycans | 23419153 | [23419153](https://www.ncbi.nlm.nih.gov/pubmed/23419153) | [NCAN](https://www.genecards.org/Search/Keyword?queryString=NCAN) |
| NCL | ECM associated regulatory factors | 8912641 | [8912641](https://www.ncbi.nlm.nih.gov/pubmed/8912641) | [NCL](https://www.genecards.org/Search/Keyword?queryString=NCL) |
| NDNF | ECM Glycoproteins | 24023955 | [24023955](https://www.ncbi.nlm.nih.gov/pubmed/24023955) | [NDNF](https://www.genecards.org/Search/Keyword?queryString=NDNF) |
| NDP | ECM Micro-enviornment associated | 15035989 | [15035989](https://www.ncbi.nlm.nih.gov/pubmed/15035989) | [NDP](https://www.genecards.org/Search/Keyword?queryString=NDP) |
| NDST1 | ECM synthetic/degradation enzyme | 9230113 | [9230113](https://www.ncbi.nlm.nih.gov/pubmed/9230113) | [NDST1](https://www.genecards.org/Search/Keyword?queryString=NDST1) |
| NDST2 | ECM synthetic/degradation enzyme | 16343444 | [16343444](https://www.ncbi.nlm.nih.gov/pubmed/16343444) | [NDST2](https://www.genecards.org/Search/Keyword?queryString=NDST2) |
| NDST3 | ECM synthetic/degradation enzyme | 9915799 | [9915799](https://www.ncbi.nlm.nih.gov/pubmed/9915799) | [NDST3](https://www.genecards.org/Search/Keyword?queryString=NDST3) |
| NDST4 | ECM synthetic/degradation enzyme | 11087757 | [11087757](https://www.ncbi.nlm.nih.gov/pubmed/11087757) | [NDST4](https://www.genecards.org/Search/Keyword?queryString=NDST4) |
| NELL1 | ECM Glycoproteins | 23419153 | [23419153](https://www.ncbi.nlm.nih.gov/pubmed/23419153) | [NELL1](https://www.genecards.org/Search/Keyword?queryString=NELL1) |
| NELL2 | ECM Glycoproteins | 23419153 | [23419153](https://www.ncbi.nlm.nih.gov/pubmed/23419153) | [NELL2](https://www.genecards.org/Search/Keyword?queryString=NELL2) |
| NES | ECM | 19429183 | [19429183](https://www.ncbi.nlm.nih.gov/pubmed/19429183) | [NES](https://www.genecards.org/Search/Keyword?queryString=NES) |
| NEU1 | ECM synthetic/degradation enzyme | 26993524 | [26993524](https://www.ncbi.nlm.nih.gov/pubmed/26993524) | [NEU1](https://www.genecards.org/Search/Keyword?queryString=NEU1) |
| NF1 | ECM | 11356864 | [11356864](https://www.ncbi.nlm.nih.gov/pubmed/11356864) | [NF1](https://www.genecards.org/Search/Keyword?queryString=NF1) |
| NGF | Secreted Factors | 21321391 | [21321391](https://www.ncbi.nlm.nih.gov/pubmed/21321391) | [NGF](https://www.genecards.org/Search/Keyword?queryString=NGF) |
| NGLY1 | ECM Regulators | 25605922 | [25605922](https://www.ncbi.nlm.nih.gov/pubmed/25605922) | [NGLY1](https://www.genecards.org/Search/Keyword?queryString=NGLY1) |
| NID1 | ECM Glycoproteins | 23419153 | [23419153](https://www.ncbi.nlm.nih.gov/pubmed/23419153) | [NID1](https://www.genecards.org/Search/Keyword?queryString=NID1) |
| NID2 | ECM Glycoproteins | 23419153 | [23419153](https://www.ncbi.nlm.nih.gov/pubmed/23419153) | [NID2](https://www.genecards.org/Search/Keyword?queryString=NID2) |
| NLRP12 | ECM Micro-enviornment associated | 19155499 | [19155499](https://www.ncbi.nlm.nih.gov/pubmed/19155499) | [NLRP12](https://www.genecards.org/Search/Keyword?queryString=NLRP12) |
| NODAL | Secreted Factors | 25672326 | [25672326](https://www.ncbi.nlm.nih.gov/pubmed/25672326) | [NODAL](https://www.genecards.org/Search/Keyword?queryString=NODAL) |
| NOG | ECM Micro-enviornment associated | 27508084 | [27508084](https://www.ncbi.nlm.nih.gov/pubmed/27508084) | [NOG](https://www.genecards.org/Search/Keyword?queryString=NOG) |
| NOS2 | ECM Micro-enviornment associated | 18597705 | [18597705](https://www.ncbi.nlm.nih.gov/pubmed/18597705) | [NOS2](https://www.genecards.org/Search/Keyword?queryString=NOS2) |
| NOV | ECM Glycoproteins | 23419153 | [23419153](https://www.ncbi.nlm.nih.gov/pubmed/23419153) | [NOV](https://www.genecards.org/Search/Keyword?queryString=NOV) |
| NPNT | ECM Glycoproteins | 23419153 | [23419153](https://www.ncbi.nlm.nih.gov/pubmed/23419153) | [NPNT](https://www.genecards.org/Search/Keyword?queryString=NPNT) |
| NRAS | ECM Micro-enviornment associated | 23000456 | [23000456](https://www.ncbi.nlm.nih.gov/pubmed/23000456) | [NRAS](https://www.genecards.org/Search/Keyword?queryString=NRAS) |
| NRG1 | Secreted Factors | 15528194 | [15528194](https://www.ncbi.nlm.nih.gov/pubmed/15528194) | [NRG1](https://www.genecards.org/Search/Keyword?queryString=NRG1) |
| NRG2 | Secreted Factors | 16732320 | [16732320](https://www.ncbi.nlm.nih.gov/pubmed/16732320) | [NRG2](https://www.genecards.org/Search/Keyword?queryString=NRG2) |
| NRG3 | Secreted Factors | 16732320 | [16732320](https://www.ncbi.nlm.nih.gov/pubmed/16732320) | [NRG3](https://www.genecards.org/Search/Keyword?queryString=NRG3) |
| NRG4 | Secreted Factors | 22159717 | [22159717](https://www.ncbi.nlm.nih.gov/pubmed/22159717) | [NRG4](https://www.genecards.org/Search/Keyword?queryString=NRG4) |
| NRTN | Secreted Factors | 22159717 | [22159717](https://www.ncbi.nlm.nih.gov/pubmed/22159717) | [NRTN](https://www.genecards.org/Search/Keyword?queryString=NRTN) |
| NTF3 | Secreted Factors | 28356268 | [28356268](https://www.ncbi.nlm.nih.gov/pubmed/28356268) | [NTF3](https://www.genecards.org/Search/Keyword?queryString=NTF3) |
| NTF4 | Secreted Factors | 15703015 | [15703015](https://www.ncbi.nlm.nih.gov/pubmed/15703015) | [NTF4](https://www.genecards.org/Search/Keyword?queryString=NTF4) |
| NTN1 | ECM Glycoproteins | 23419153 | [23419153](https://www.ncbi.nlm.nih.gov/pubmed/23419153) | [NTN1](https://www.genecards.org/Search/Keyword?queryString=NTN1) |
| NTN3 | ECM Glycoproteins | 23419153 | [23419153](https://www.ncbi.nlm.nih.gov/pubmed/23419153) | [NTN3](https://www.genecards.org/Search/Keyword?queryString=NTN3) |
| NTN4 | ECM Glycoproteins | 23419153 | [23419153](https://www.ncbi.nlm.nih.gov/pubmed/23419153) | [NTN4](https://www.genecards.org/Search/Keyword?queryString=NTN4) |
| NTN5 | ECM Glycoproteins | 23419153 | [23419153](https://www.ncbi.nlm.nih.gov/pubmed/23419153) | [NTN5](https://www.genecards.org/Search/Keyword?queryString=NTN5) |
| NTNG1 | ECM Glycoproteins | 23419153 | [23419153](https://www.ncbi.nlm.nih.gov/pubmed/23419153) | [NTNG1](https://www.genecards.org/Search/Keyword?queryString=NTNG1) |
| NTNG2 | ECM Glycoproteins | 23419153 | [23419153](https://www.ncbi.nlm.nih.gov/pubmed/23419153) | [NTNG2](https://www.genecards.org/Search/Keyword?queryString=NTNG2) |
| NUDT16L1 | ECM Micro-enviornment associated | 11805099 | [11805099](https://www.ncbi.nlm.nih.gov/pubmed/11805099) | [NUDT16L1](https://www.genecards.org/Search/Keyword?queryString=NUDT16L1) |
| NYX | Proteoglycans | 16261423 | [16261423](https://www.ncbi.nlm.nih.gov/pubmed/16261423) | [NYX](https://www.genecards.org/Search/Keyword?queryString=NYX) |
| OGFOD1 | ECM Regulators | 22159717 | [22159717](https://www.ncbi.nlm.nih.gov/pubmed/22159717) | [OGFOD1](https://www.genecards.org/Search/Keyword?queryString=OGFOD1) |
| OGFOD2 | ECM Regulators | 22159717 | [22159717](https://www.ncbi.nlm.nih.gov/pubmed/22159717) | [OGFOD2](https://www.genecards.org/Search/Keyword?queryString=OGFOD2) |
| OGN | Proteoglycans | 25410057 | [25410057](https://www.ncbi.nlm.nih.gov/pubmed/25410057) | [OGN](https://www.genecards.org/Search/Keyword?queryString=OGN) |
| OIT3 | ECM Glycoproteins | 23419153 | [23419153](https://www.ncbi.nlm.nih.gov/pubmed/23419153) | [OIT3](https://www.genecards.org/Search/Keyword?queryString=OIT3) |
| OMD | Proteoglycans | 22159717 | [22159717](https://www.ncbi.nlm.nih.gov/pubmed/22159717) | [OMD](https://www.genecards.org/Search/Keyword?queryString=OMD) |
| OPTC | Proteoglycans | 22159717 | [22159717](https://www.ncbi.nlm.nih.gov/pubmed/22159717) | [OPTC](https://www.genecards.org/Search/Keyword?queryString=OPTC) |
| OSM | Secreted Factors | 25622278 | [25622278](https://www.ncbi.nlm.nih.gov/pubmed/25622278) | [OSM](https://www.genecards.org/Search/Keyword?queryString=OSM) |
| OTOG | ECM Glycoproteins | 23419153 | [23419153](https://www.ncbi.nlm.nih.gov/pubmed/23419153) | [OTOG](https://www.genecards.org/Search/Keyword?queryString=OTOG) |
| OTOL1 | ECM Glycoproteins | 20856818 | [20856818](https://www.ncbi.nlm.nih.gov/pubmed/20856818) | [OTOL1](https://www.genecards.org/Search/Keyword?queryString=OTOL1) |
| OVGP1 | ECM-affiliated Proteins | 7819450 | [7819450](https://www.ncbi.nlm.nih.gov/pubmed/7819450) | [OVGP1](https://www.genecards.org/Search/Keyword?queryString=OVGP1) |
| OXT | ECM Micro-enviornment associated | 28238786 | [28238786](https://www.ncbi.nlm.nih.gov/pubmed/28238786) | [OXT](https://www.genecards.org/Search/Keyword?queryString=OXT) |
| P2RX3 | ECM associated regulatory factors | 28639246 | [28639246](https://www.ncbi.nlm.nih.gov/pubmed/28639246) | [P2RX3](https://www.genecards.org/Search/Keyword?queryString=P2RX3) |
| P3H1 | ECM Micro-enviornment associated | 17630507 | [17630507](https://www.ncbi.nlm.nih.gov/pubmed/17630507) | [P3H1](https://www.genecards.org/Search/Keyword?queryString=P3H1) |
| P3H3 | ECM Micro-enviornment associated | 21757687 | [21757687](https://www.ncbi.nlm.nih.gov/pubmed/21757687) | [P3H3](https://www.genecards.org/Search/Keyword?queryString=P3H3) |
| P4HA1 | ECM Regulators | 24827502 | [24827502](https://www.ncbi.nlm.nih.gov/pubmed/24827502) | [P4HA1](https://www.genecards.org/Search/Keyword?queryString=P4HA1) |
| P4HA2 | ECM Regulators | 24827502 | [24827502](https://www.ncbi.nlm.nih.gov/pubmed/24827502) | [P4HA2](https://www.genecards.org/Search/Keyword?queryString=P4HA2) |
| P4HA3 | ECM Regulators | 24827502 | [24827502](https://www.ncbi.nlm.nih.gov/pubmed/24827502) | [P4HA3](https://www.genecards.org/Search/Keyword?queryString=P4HA3) |
| P4HTM | ECM Regulators | 22159717 | [22159717](https://www.ncbi.nlm.nih.gov/pubmed/22159717) | [P4HTM](https://www.genecards.org/Search/Keyword?queryString=P4HTM) |
| PAK1 | ECM associated regulatory factors | 28534988 | [28534988](https://www.ncbi.nlm.nih.gov/pubmed/28534988) | [PAK1](https://www.genecards.org/Search/Keyword?queryString=PAK1) |
| PAMR1 | ECM Regulators | 24921011 | [24921011](https://www.ncbi.nlm.nih.gov/pubmed/24921011) | [PAMR1](https://www.genecards.org/Search/Keyword?queryString=PAMR1) |
| PAPLN | ECM Glycoproteins | 23419153 | [23419153](https://www.ncbi.nlm.nih.gov/pubmed/23419153) | [PAPLN](https://www.genecards.org/Search/Keyword?queryString=PAPLN) |
| PAPPA | ECM Regulators | 11836256 | [11836256](https://www.ncbi.nlm.nih.gov/pubmed/11836256) | [PAPPA](https://www.genecards.org/Search/Keyword?queryString=PAPPA) |
| PAPPA2 | ECM Regulators | 26417939 | [26417939](https://www.ncbi.nlm.nih.gov/pubmed/26417939) | [PAPPA2](https://www.genecards.org/Search/Keyword?queryString=PAPPA2) |
| PARM1 | ECM-affiliated Proteins | 22159717 | [22159717](https://www.ncbi.nlm.nih.gov/pubmed/22159717) | [PARM1](https://www.genecards.org/Search/Keyword?queryString=PARM1) |
| PBCRA1 | ECM Micro-enviornment associated | 9691169 | [9691169](https://www.ncbi.nlm.nih.gov/pubmed/9691169) | [PBCRA1](https://www.genecards.org/Search/Keyword?queryString=PBCRA1) |
| PCOLCE | ECM Glycoproteins | 23419153 | [23419153](https://www.ncbi.nlm.nih.gov/pubmed/23419153) | [PCOLCE](https://www.genecards.org/Search/Keyword?queryString=PCOLCE) |
| PCOLCE2 | ECM Glycoproteins | 23419153 | [23419153](https://www.ncbi.nlm.nih.gov/pubmed/23419153) | [PCOLCE2](https://www.genecards.org/Search/Keyword?queryString=PCOLCE2) |
| PCSK5 | ECM Regulators | 15911696 | [15911696](https://www.ncbi.nlm.nih.gov/pubmed/15911696) | [PCSK5](https://www.genecards.org/Search/Keyword?queryString=PCSK5) |
| PCSK6 | ECM Regulators | 12535616 | [12535616](https://www.ncbi.nlm.nih.gov/pubmed/12535616) | [PCSK6](https://www.genecards.org/Search/Keyword?queryString=PCSK6) |
| PDCD4 | ECM Micro-enviornment associated | 22087031 | [22087031](https://www.ncbi.nlm.nih.gov/pubmed/22087031) | [PDCD4](https://www.genecards.org/Search/Keyword?queryString=PDCD4) |
| PDGFA | Secreted Factors | 10567196 | [10567196](https://www.ncbi.nlm.nih.gov/pubmed/10567196) | [PDGFA](https://www.genecards.org/Search/Keyword?queryString=PDGFA) |
| PDGFB | Secreted Factors | 25678707 | [25678707](https://www.ncbi.nlm.nih.gov/pubmed/25678707) | [PDGFB](https://www.genecards.org/Search/Keyword?queryString=PDGFB) |
| PDGFC | Secreted Factors | 25678707 | [25678707](https://www.ncbi.nlm.nih.gov/pubmed/25678707) | [PDGFC](https://www.genecards.org/Search/Keyword?queryString=PDGFC) |
| PDGFD | Secreted Factors | 25678707 | [25678707](https://www.ncbi.nlm.nih.gov/pubmed/25678707) | [PDGFD](https://www.genecards.org/Search/Keyword?queryString=PDGFD) |
| PDGFRA | ECM Micro-enviornment associated | 10358027 | [10358027](https://www.ncbi.nlm.nih.gov/pubmed/10358027) | [PDGFRA](https://www.genecards.org/Search/Keyword?queryString=PDGFRA) |
| PDPK1 | ECM Micro-enviornment associated | 16551362 | [16551362](https://www.ncbi.nlm.nih.gov/pubmed/16551362) | [PDPK1](https://www.genecards.org/Search/Keyword?queryString=PDPK1) |
| PDX1 | ECM associated regulatory factors | 18468239 | [18468239](https://www.ncbi.nlm.nih.gov/pubmed/18468239) | [PDX1](https://www.genecards.org/Search/Keyword?queryString=PDX1) |
| PF4 | Secreted Factors | 20454664 | [20454664](https://www.ncbi.nlm.nih.gov/pubmed/20454664) | [PF4](https://www.genecards.org/Search/Keyword?queryString=PF4) |
| PF4V1 | Secreted Factors | 28057004 | [28057004](https://www.ncbi.nlm.nih.gov/pubmed/28057004) | [PF4V1](https://www.genecards.org/Search/Keyword?queryString=PF4V1) |
| PGF | Secreted Factors | 11870075 | [11870075](https://www.ncbi.nlm.nih.gov/pubmed/11870075) | [PGF](https://www.genecards.org/Search/Keyword?queryString=PGF) |
| PHEX | ECM Micro-enviornment associated | 12678920 | [12678920](https://www.ncbi.nlm.nih.gov/pubmed/12678920) | [PHEX](https://www.genecards.org/Search/Keyword?queryString=PHEX) |
| PI3 | ECM Regulators | 8476637 | [8476637](https://www.ncbi.nlm.nih.gov/pubmed/8476637) | [PI3](https://www.genecards.org/Search/Keyword?queryString=PI3) |
| PIK3CA | ECM Micro-enviornment associated | 25326806 | [25326806](https://www.ncbi.nlm.nih.gov/pubmed/25326806) | [PIK3CA](https://www.genecards.org/Search/Keyword?queryString=PIK3CA) |
| PIK3CB | ECM Micro-enviornment associated | 19682436 | [19682436](https://www.ncbi.nlm.nih.gov/pubmed/19682436) | [PIK3CB](https://www.genecards.org/Search/Keyword?queryString=PIK3CB) |
| PIK3CD | ECM Micro-enviornment associated | 18332138 | [18332138](https://www.ncbi.nlm.nih.gov/pubmed/18332138) | [PIK3CD](https://www.genecards.org/Search/Keyword?queryString=PIK3CD) |
| PIK3CG | ECM Micro-enviornment associated | 18434090 | [18434090](https://www.ncbi.nlm.nih.gov/pubmed/18434090) | [PIK3CG](https://www.genecards.org/Search/Keyword?queryString=PIK3CG) |
| PIK3IP1 | Secreted Factors | 22159717 | [22159717](https://www.ncbi.nlm.nih.gov/pubmed/22159717) | [PIK3IP1](https://www.genecards.org/Search/Keyword?queryString=PIK3IP1) |
| PIK3R1 | ECM Micro-enviornment associated | 19686810 | [19686810](https://www.ncbi.nlm.nih.gov/pubmed/19686810) | [PIK3R1](https://www.genecards.org/Search/Keyword?queryString=PIK3R1) |
| PIK3R2 | ECM Micro-enviornment associated | 22623428 | [22623428](https://www.ncbi.nlm.nih.gov/pubmed/22623428) | [PIK3R2](https://www.genecards.org/Search/Keyword?queryString=PIK3R2) |
| PLA2G10 | ECM Micro-enviornment associated | 14726411 | [14726411](https://www.ncbi.nlm.nih.gov/pubmed/14726411) | [PLA2G10](https://www.genecards.org/Search/Keyword?queryString=PLA2G10) |
| PLA2G2A | ECM Micro-enviornment associated | 12773489 | [12773489](https://www.ncbi.nlm.nih.gov/pubmed/12773489) | [PLA2G2A](https://www.genecards.org/Search/Keyword?queryString=PLA2G2A) |
| PLA2G2D | ECM Micro-enviornment associated | 9377118 | [9377118](https://www.ncbi.nlm.nih.gov/pubmed/9377118) | [PLA2G2D](https://www.genecards.org/Search/Keyword?queryString=PLA2G2D) |
| PLA2G3 | ECM Micro-enviornment associated | 10713052 | [10713052](https://www.ncbi.nlm.nih.gov/pubmed/10713052) | [PLA2G3](https://www.genecards.org/Search/Keyword?queryString=PLA2G3) |
| PLA2G4A | ECM Micro-enviornment associated | 16926552 | [16926552](https://www.ncbi.nlm.nih.gov/pubmed/16926552) | [PLA2G4A](https://www.genecards.org/Search/Keyword?queryString=PLA2G4A) |
| PLAT | ECM Regulators | 23360524 | [23360524](https://www.ncbi.nlm.nih.gov/pubmed/23360524) | [PLAT](https://www.genecards.org/Search/Keyword?queryString=PLAT) |
| PLAU | ECM Regulators | 21423176 | [21423176](https://www.ncbi.nlm.nih.gov/pubmed/21423176) | [PLAU](https://www.genecards.org/Search/Keyword?queryString=PLAU) |
| PLAUR | ECM Micro-enviornment associated | 25456503 | [25456503](https://www.ncbi.nlm.nih.gov/pubmed/25456503) | [PLAUR](https://www.genecards.org/Search/Keyword?queryString=PLAUR) |
| PLCB1 | ECM Micro-enviornment associated | 9802880 | [9802880](https://www.ncbi.nlm.nih.gov/pubmed/9802880) | [PLCB1](https://www.genecards.org/Search/Keyword?queryString=PLCB1) |
| PLCB4 | ECM Micro-enviornment associated | 1654337 | [1654337](https://www.ncbi.nlm.nih.gov/pubmed/1654337) | [PLCB4](https://www.genecards.org/Search/Keyword?queryString=PLCB4) |
| PLCE1 | ECM Micro-enviornment associated | 24316392 | [24316392](https://www.ncbi.nlm.nih.gov/pubmed/24316392) | [PLCE1](https://www.genecards.org/Search/Keyword?queryString=PLCE1) |
| PLCG1 | ECM Micro-enviornment associated | 10430888 | [10430888](https://www.ncbi.nlm.nih.gov/pubmed/10430888) | [PLCG1](https://www.genecards.org/Search/Keyword?queryString=PLCG1) |
| PLCG2 | ECM Micro-enviornment associated | 10469124 | [10469124](https://www.ncbi.nlm.nih.gov/pubmed/10469124) | [PLCG2](https://www.genecards.org/Search/Keyword?queryString=PLCG2) |
| PLG | ECM Regulators | 22159717 | [22159717](https://www.ncbi.nlm.nih.gov/pubmed/22159717) | [PLG](https://www.genecards.org/Search/Keyword?queryString=PLG) |
| PLOD1 | ECM Regulators | 24285264 | [24285264](https://www.ncbi.nlm.nih.gov/pubmed/24285264) | [PLOD1](https://www.genecards.org/Search/Keyword?queryString=PLOD1) |
| PLOD2 | ECM Regulators | 24285264 | [24285264](https://www.ncbi.nlm.nih.gov/pubmed/24285264) | [PLOD2](https://www.genecards.org/Search/Keyword?queryString=PLOD2) |
| PLOD3 | ECM Regulators | 24285264 | [24285264](https://www.ncbi.nlm.nih.gov/pubmed/24285264) | [PLOD3](https://www.genecards.org/Search/Keyword?queryString=PLOD3) |
| PLXDC1 | ECM-affiliated Proteins | 25535841 | [25535841](https://www.ncbi.nlm.nih.gov/pubmed/25535841) | [PLXDC1](https://www.genecards.org/Search/Keyword?queryString=PLXDC1) |
| PLXDC2 | ECM-affiliated Proteins | 25535841 | [25535841](https://www.ncbi.nlm.nih.gov/pubmed/25535841) | [PLXDC2](https://www.genecards.org/Search/Keyword?queryString=PLXDC2) |
| PLXNA1 | ECM-affiliated Proteins | 20512151 | [20512151](https://www.ncbi.nlm.nih.gov/pubmed/20512151) | [PLXNA1](https://www.genecards.org/Search/Keyword?queryString=PLXNA1) |
| PLXNA2 | ECM-affiliated Proteins | 22783168 | [22783168](https://www.ncbi.nlm.nih.gov/pubmed/22783168) | [PLXNA2](https://www.genecards.org/Search/Keyword?queryString=PLXNA2) |
| PLXNA3 | ECM-affiliated Proteins | 19909241 | [19909241](https://www.ncbi.nlm.nih.gov/pubmed/19909241) | [PLXNA3](https://www.genecards.org/Search/Keyword?queryString=PLXNA3) |
| PLXNA4 | ECM-affiliated Proteins | 19909241 | [19909241](https://www.ncbi.nlm.nih.gov/pubmed/19909241) | [PLXNA4](https://www.genecards.org/Search/Keyword?queryString=PLXNA4) |
| PLXNB1 | ECM-affiliated Proteins | 19909241 | [19909241](https://www.ncbi.nlm.nih.gov/pubmed/19909241) | [PLXNB1](https://www.genecards.org/Search/Keyword?queryString=PLXNB1) |
| PLXNB2 | ECM-affiliated Proteins | 27988214 | [27988214](https://www.ncbi.nlm.nih.gov/pubmed/27988214) | [PLXNB2](https://www.genecards.org/Search/Keyword?queryString=PLXNB2) |
| PLXNB3 | ECM-affiliated Proteins | 22159717 | [22159717](https://www.ncbi.nlm.nih.gov/pubmed/22159717) | [PLXNB3](https://www.genecards.org/Search/Keyword?queryString=PLXNB3) |
| PLXNC1 | ECM-affiliated Proteins | 24064357 | [24064357](https://www.ncbi.nlm.nih.gov/pubmed/24064357) | [PLXNC1](https://www.genecards.org/Search/Keyword?queryString=PLXNC1) |
| PLXND1 | ECM-affiliated Proteins | 20880496 | [20880496](https://www.ncbi.nlm.nih.gov/pubmed/20880496) | [PLXND1](https://www.genecards.org/Search/Keyword?queryString=PLXND1) |
| PODN | Proteoglycans | 21937732 | [21937732](https://www.ncbi.nlm.nih.gov/pubmed/21937732) | [PODN](https://www.genecards.org/Search/Keyword?queryString=PODN) |
| PODNL1 | Proteoglycans | 26290530 | [26290530](https://www.ncbi.nlm.nih.gov/pubmed/26290530) | [PODNL1](https://www.genecards.org/Search/Keyword?queryString=PODNL1) |
| POMGNT2 | ECM synthetic/degradation enzyme | 17113869 | [17113869](https://www.ncbi.nlm.nih.gov/pubmed/17113869) | [POMGNT2](https://www.genecards.org/Search/Keyword?queryString=POMGNT2) |
| POMZP3 | ECM Glycoproteins | 22159717 | [22159717](https://www.ncbi.nlm.nih.gov/pubmed/22159717  ) | [POMZP3](https://www.genecards.org/Search/Keyword?queryString=POMZP3) |
| POSTN | ECM Glycoproteins | 27307601 | [27307601](https://www.ncbi.nlm.nih.gov/pubmed/27307601) | [POSTN](https://www.genecards.org/Search/Keyword?queryString=POSTN) |
| PPARA | ECM Micro-enviornment associated | 19217440 | [19217440](https://www.ncbi.nlm.nih.gov/pubmed/19217440) | [PPARA](https://www.genecards.org/Search/Keyword?queryString=PPARA) |
| PPARD | ECM Micro-enviornment associated | 19538467 | [19538467](https://www.ncbi.nlm.nih.gov/pubmed/19538467) | [PPARD](https://www.genecards.org/Search/Keyword?queryString=PPARD) |
| PPARG | ECM Micro-enviornment associated | 16795079 | [16795079](https://www.ncbi.nlm.nih.gov/pubmed/16795079) | [PPARG](https://www.genecards.org/Search/Keyword?queryString=PPARG) |
| PPBP | Secreted Factors | 22159717 | [22159717](https://www.ncbi.nlm.nih.gov/pubmed/22159717) | [PPBP](https://www.genecards.org/Search/Keyword?queryString=PPBP) |
| PPP1CA | ECM Micro-enviornment associated | 25468996 | [25468996](https://www.ncbi.nlm.nih.gov/pubmed/25468996) | [PPP1CA](https://www.genecards.org/Search/Keyword?queryString=PPP1CA) |
| PPP1CB | ECM Micro-enviornment associated | 21423176 | [21423176](https://www.ncbi.nlm.nih.gov/pubmed/21423176) | [PPP1CB](https://www.genecards.org/Search/Keyword?queryString=PPP1CB) |
| PPP1CC | ECM Micro-enviornment associated | 21423176 | [21423176](https://www.ncbi.nlm.nih.gov/pubmed/21423176) | [PPP1CC](https://www.genecards.org/Search/Keyword?queryString=PPP1CC) |
| PPP1R12A | ECM Micro-enviornment associated | 21423176 | [21423176](https://www.ncbi.nlm.nih.gov/pubmed/21423176) | [PPP1R12A](https://www.genecards.org/Search/Keyword?queryString=PPP1R12A) |
| PPP1R12B | ECM Micro-enviornment associated | 9269769 | [9269769](https://www.ncbi.nlm.nih.gov/pubmed/9269769) | [PPP1R12B](https://www.genecards.org/Search/Keyword?queryString=PPP1R12B) |
| PPP1R12C | ECM Micro-enviornment associated | 11399775 | [11399775](https://www.ncbi.nlm.nih.gov/pubmed/11399775) | [PPP1R12C](https://www.genecards.org/Search/Keyword?queryString=PPP1R12C) |
| PRB1 | ECM | 9487127 | [9487127](https://www.ncbi.nlm.nih.gov/pubmed/9487127) | [PRB1](https://www.genecards.org/Search/Keyword?queryString=PRB1) |
| PRB4 | ECM | 9487127 | [9487127](https://www.ncbi.nlm.nih.gov/pubmed/9487127) | [PRB4](https://www.genecards.org/Search/Keyword?queryString=PRB4) |
| PRELP | Proteoglycans | 26477596 | [26477596](https://www.ncbi.nlm.nih.gov/pubmed/26477596) | [PRELP](https://www.genecards.org/Search/Keyword?queryString=PRELP) |
| PRG2 | Proteoglycans | 28071719 | [28071719](https://www.ncbi.nlm.nih.gov/pubmed/28071719) | [PRG2](https://www.genecards.org/Search/Keyword?queryString=PRG2) |
| PRG3 | Proteoglycans | 21937732 | [21937732](https://www.ncbi.nlm.nih.gov/pubmed/21937732) | [PRG3](https://www.genecards.org/Search/Keyword?queryString=PRG3) |
| PRG4 | Proteoglycans | 21937732 | [21937732](https://www.ncbi.nlm.nih.gov/pubmed/21937732) | [PRG4](https://www.genecards.org/Search/Keyword?queryString=PRG4) |
| PRKACA | ECM associated regulatory factors | 19368997 | [19368997](https://www.ncbi.nlm.nih.gov/pubmed/19368997) | [PRKACA](https://www.genecards.org/Search/Keyword?queryString=PRKACA) |
| PRKACB | ECM associated regulatory factors | 23376485 | [23376485](https://www.ncbi.nlm.nih.gov/pubmed/23376485) | [PRKACB](https://www.genecards.org/Search/Keyword?queryString=PRKACB) |
| PRKCA | ECM associated regulatory factors | 9553124 | [9553124](https://www.ncbi.nlm.nih.gov/pubmed/9553124) | [PRKCA](https://www.genecards.org/Search/Keyword?queryString=PRKCA) |
| PRKCB | ECM associated regulatory factors | 9244383 | [9244383](https://www.ncbi.nlm.nih.gov/pubmed/9244383) | [PRKCB](https://www.genecards.org/Search/Keyword?queryString=PRKCB) |
| PRKCG | ECM associated regulatory factors | 9244383 | [9244383](https://www.ncbi.nlm.nih.gov/pubmed/9244383) | [PRKCG](https://www.genecards.org/Search/Keyword?queryString=PRKCG) |
| PRL | Secreted Factors | 15778982 | [15778982](https://www.ncbi.nlm.nih.gov/pubmed/15778982) | [PRL](https://www.genecards.org/Search/Keyword?queryString=PRL) |
| PRNP | ECM | 24970228 | [24970228](https://www.ncbi.nlm.nih.gov/pubmed/24970228) | [PRNP](https://www.genecards.org/Search/Keyword?queryString=PRNP) |
| PROL1 | ECM-affiliated Proteins | 12646701 | [12646701](https://www.ncbi.nlm.nih.gov/pubmed/12646701) | [PROL1](https://www.genecards.org/Search/Keyword?queryString=PROL1) |
| PRSS1 | ECM Regulators | 28071719 | [28071719](https://www.ncbi.nlm.nih.gov/pubmed/28071719) | [PRSS1](https://www.genecards.org/Search/Keyword?queryString=PRSS1) |
| PRSS12 | ECM Regulators | 25368556 | [25368556](https://www.ncbi.nlm.nih.gov/pubmed/25368556) | [PRSS12](https://www.genecards.org/Search/Keyword?queryString=PRSS12) |
| PRSS2 | ECM Regulators | 18787072 | [18787072](https://www.ncbi.nlm.nih.gov/pubmed/18787072) | [PRSS2](https://www.genecards.org/Search/Keyword?queryString=PRSS2) |
| PRSS3 | ECM Regulators | 4695067 | [4695067](https://www.ncbi.nlm.nih.gov/pubmed/4695067) | [PRSS3](https://www.genecards.org/Search/Keyword?queryString=PRSS3) |
| PRTN3 | ECM synthetic/degradation enzyme | 20551380 | [20551380](https://www.ncbi.nlm.nih.gov/pubmed/20551380) | [PRTN3](https://www.genecards.org/Search/Keyword?queryString=PRTN3) |
| PSG1 | ECM | 21193412 | [21193412](https://www.ncbi.nlm.nih.gov/pubmed/21193412) | [PSG1](https://www.genecards.org/Search/Keyword?queryString=PSG1) |
| PSPN | Secreted Factors | 21200028 | [21200028](https://www.ncbi.nlm.nih.gov/pubmed/21200028) | [PSPN](https://www.genecards.org/Search/Keyword?queryString=PSPN) |
| PTCH1 | ECM | 19287498 | [19287498](https://www.ncbi.nlm.nih.gov/pubmed/19287498) | [PTCH1](https://www.genecards.org/Search/Keyword?queryString=PTCH1) |
| PTGS2 | ECM synthetic/degradation enzyme | 18378414 | [18378414](https://www.ncbi.nlm.nih.gov/pubmed/18378414) | [PTGS2](https://www.genecards.org/Search/Keyword?queryString=PTGS2) |
| PTH | ECM Micro-enviornment associated | 27530924 | [27530924](https://www.ncbi.nlm.nih.gov/pubmed/27530924) | [PTH](https://www.genecards.org/Search/Keyword?queryString=PTH) |
| PTK2 | ECM Micro-enviornment associated | 10655584 | [10655584](https://www.ncbi.nlm.nih.gov/pubmed/10655584) | [PTK2](https://www.genecards.org/Search/Keyword?queryString=PTK2) |
| PTK2B | ECM Micro-enviornment associated | 18765415 | [18765415](https://www.ncbi.nlm.nih.gov/pubmed/18765415) | [PTK2B](https://www.genecards.org/Search/Keyword?queryString=PTK2B) |
| PTN | Secreted Factors | 26399645 | [26399645](https://www.ncbi.nlm.nih.gov/pubmed/26399645) | [PTN](https://www.genecards.org/Search/Keyword?queryString=PTN) |
| PTPN11 | ECM Micro-enviornment associated | 20472558 | [20472558](https://www.ncbi.nlm.nih.gov/pubmed/20472558) | [PTPN11](https://www.genecards.org/Search/Keyword?queryString=PTPN11) |
| PTPN6 | ECM Micro-enviornment associated | 19056867 | [19056867](https://www.ncbi.nlm.nih.gov/pubmed/19056867) | [PTPN6](https://www.genecards.org/Search/Keyword?queryString=PTPN6) |
| PTPRB | ECM Micro-enviornment associated | 16955703 | [16955703](https://www.ncbi.nlm.nih.gov/pubmed/16955703) | [PTPRB](https://www.genecards.org/Search/Keyword?queryString=PTPRB) |
| PTPRF | ECM Micro-enviornment associated | 19199708 | [19199708](https://www.ncbi.nlm.nih.gov/pubmed/19199708) | [PTPRF](https://www.genecards.org/Search/Keyword?queryString=PTPRF) |
| PTPRS | ECM Micro-enviornment associated | 21454754 | [21454754](https://www.ncbi.nlm.nih.gov/pubmed/21454754) | [PTPRS](https://www.genecards.org/Search/Keyword?queryString=PTPRS) |
| PTPRT | ECM Micro-enviornment associated | 18644975 | [18644975](https://www.ncbi.nlm.nih.gov/pubmed/18644975) | [PTPRT](https://www.genecards.org/Search/Keyword?queryString=PTPRT) |
| PTPRZ1 | ECM Micro-enviornment associated | 12700241 | [12700241](https://www.ncbi.nlm.nih.gov/pubmed/12700241) | [PTPRZ1](https://www.genecards.org/Search/Keyword?queryString=PTPRZ1) |
| PXDN | ECM Glycoproteins | 21937732 | [21937732](https://www.ncbi.nlm.nih.gov/pubmed/21937732) | [PXDN](https://www.genecards.org/Search/Keyword?queryString=PXDN) |
| PXDNL | ECM Glycoproteins | 24253521 | [24253521](https://www.ncbi.nlm.nih.gov/pubmed/24253521) | [PXDNL](https://www.genecards.org/Search/Keyword?queryString=PXDNL) |
| PXN | Cytoskeleton | 26895766 | [26895766](https://www.ncbi.nlm.nih.gov/pubmed/26895766) | [PXN](https://www.genecards.org/Search/Keyword?queryString=PXN) |
| PXYLP1 | ECM Micro-enviornment associated | 24425863 | [24425863](https://www.ncbi.nlm.nih.gov/pubmed/24425863) | [PXYLP1](https://www.genecards.org/Search/Keyword?queryString=PXYLP1) |
| PZP | ECM Regulators | 28071719 | [28071719](https://www.ncbi.nlm.nih.gov/pubmed/28071719) | [PZP](https://www.genecards.org/Search/Keyword?queryString=PZP) |
| RAC1 | ECM Micro-enviornment associated | 21642434 | [21642434](https://www.ncbi.nlm.nih.gov/pubmed/21642434) | [RAC1](https://www.genecards.org/Search/Keyword?queryString=RAC1) |
| RAF1 | ECM Micro-enviornment associated | 11459784 | [11459784](https://www.ncbi.nlm.nih.gov/pubmed/11459784) | [RAF1](https://www.genecards.org/Search/Keyword?queryString=RAF1) |
| RDX | ECM | 10462524 | [10462524](https://www.ncbi.nlm.nih.gov/pubmed/10462524) | [RDX](https://www.genecards.org/Search/Keyword?queryString=RDX) |
| REG1A | ECM-affiliated Proteins | 19239705 | [19239705](https://www.ncbi.nlm.nih.gov/pubmed/19239705) | [REG1A](https://www.genecards.org/Search/Keyword?queryString=REG1A) |
| REG1B | ECM-affiliated Proteins | 28366542 | [28366542](https://www.ncbi.nlm.nih.gov/pubmed/28366542) | [REG1B](https://www.genecards.org/Search/Keyword?queryString=REG1B) |
| REG3A | ECM-affiliated Proteins | 19239705 | [19239705](https://www.ncbi.nlm.nih.gov/pubmed/19239705) | [REG3A](https://www.genecards.org/Search/Keyword?queryString=REG3A) |
| REG3G | ECM-affiliated Proteins | 19239705 | [19239705](https://www.ncbi.nlm.nih.gov/pubmed/19239705) | [REG3G](https://www.genecards.org/Search/Keyword?queryString=REG3G) |
| REG4 | ECM-affiliated Proteins | 27036049 | [27036049](https://www.ncbi.nlm.nih.gov/pubmed/ 27036049) | [REG4](https://www.genecards.org/Search/Keyword?queryString=REG4) |
| RELN | ECM Glycoproteins | 28345605 | [28345605](https://www.ncbi.nlm.nih.gov/pubmed/28345605) | [RELN](https://www.genecards.org/Search/Keyword?queryString=RELN) |
| RHOA | ECM Micro-enviornment associated | 19538537 | [19538537](https://www.ncbi.nlm.nih.gov/pubmed/19538537) | [RHOA](https://www.genecards.org/Search/Keyword?queryString=RHOA) |
| RNASE3 | ECM associated regulatory factors | 1493801 | [1493801](https://www.ncbi.nlm.nih.gov/pubmed/1493801) | [RNASE3](https://www.genecards.org/Search/Keyword?queryString=RNASE3) |
| RNF5 | ECM associated regulatory factors | 10967549 | [10967549](https://www.ncbi.nlm.nih.gov/pubmed/10967549) | [RNF5](https://www.genecards.org/Search/Keyword?queryString=RNF5) |
| ROCK1 | ECM Micro-enviornment associated | 18668558 | [18668558](https://www.ncbi.nlm.nih.gov/pubmed/18668558) | [ROCK1](https://www.genecards.org/Search/Keyword?queryString=ROCK1) |
| ROCK2 | ECM Micro-enviornment associated | 18668558 | [18668558](https://www.ncbi.nlm.nih.gov/pubmed/18668558) | [ROCK2](https://www.genecards.org/Search/Keyword?queryString=ROCK2) |
| RPL29 | ECM associated regulatory factors | 8397099 | [8397099](https://www.ncbi.nlm.nih.gov/pubmed/8397099) | [RPL29](https://www.genecards.org/Search/Keyword?queryString=RPL29) |
| RPS6 | ECM associated regulatory factors | 21989018 | [21989018](https://www.ncbi.nlm.nih.gov/pubmed/21989018) | [RPS6](https://www.genecards.org/Search/Keyword?queryString=RPS6) |
| RPS6KB1 | ECM associated regulatory factors | 15246963 | [15246963](https://www.ncbi.nlm.nih.gov/pubmed/15246963) | [RPS6KB1](https://www.genecards.org/Search/Keyword?queryString=RPS6KB1) |
| RPS6KB2 | ECM associated regulatory factors | 25843685 | [25843685](https://www.ncbi.nlm.nih.gov/pubmed/25843685) | [RPS6KB2](https://www.genecards.org/Search/Keyword?queryString=RPS6KB2) |
| RPTN | Secreted Factors | 26095047 | [26095047](https://www.ncbi.nlm.nih.gov/pubmed/26095047) | [RPTN](https://www.genecards.org/Search/Keyword?queryString=RPTN) |
| RRAS | ECM Micro-enviornment associated | 21423176 | [21423176](https://www.ncbi.nlm.nih.gov/pubmed/21423176) | [RRAS](https://www.genecards.org/Search/Keyword?queryString=RRAS) |
| RRAS2 | ECM Micro-enviornment associated | 21423176 | [21423176](https://www.ncbi.nlm.nih.gov/pubmed/21423176) | [RRAS2](https://www.genecards.org/Search/Keyword?queryString=RRAS2) |
| RSPO1 | ECM Glycoproteins | 21937732 | [21937732](https://www.ncbi.nlm.nih.gov/pubmed/21937732) | [RSPO1](https://www.genecards.org/Search/Keyword?queryString=RSPO1) |
| RSPO2 | ECM Glycoproteins | 21937732 | [21937732](https://www.ncbi.nlm.nih.gov/pubmed/21937732) | [RSPO2](https://www.genecards.org/Search/Keyword?queryString=RSPO2) |
| RSPO3 | ECM Glycoproteins | 21937732 | [21937732](https://www.ncbi.nlm.nih.gov/pubmed/21937732) | [RSPO3](https://www.genecards.org/Search/Keyword?queryString=RSPO3) |
| RSPO4 | ECM Glycoproteins | 21937732 | [21937732](https://www.ncbi.nlm.nih.gov/pubmed/21937732) | [RSPO4](https://www.genecards.org/Search/Keyword?queryString=RSPO4) |
| RTN4 | ECM | 17234155 | [17234155](https://www.ncbi.nlm.nih.gov/pubmed/17234155) | [RTN4](https://www.genecards.org/Search/Keyword?queryString=RTN4) |
| RTN4R | ECM receptor | 16712417 | [16712417](https://www.ncbi.nlm.nih.gov/pubmed/16712417) | [RTN4R](https://www.genecards.org/Search/Keyword?queryString=RTN4R) |
| RTN4RL1 | ECM receptor | 20463223 | [20463223](https://www.ncbi.nlm.nih.gov/pubmed/20463223) | [RTN4RL1](https://www.genecards.org/Search/Keyword?queryString=RTN4RL1) |
| RUNX1 | ECM associated regulatory factors | 20339004 | [20339004](https://www.ncbi.nlm.nih.gov/pubmed/20339004) | [RUNX1](https://www.genecards.org/Search/Keyword?queryString=RUNX1) |
| RUNX2 | ECM associated regulatory factors | 19259985 | [19259985](https://www.ncbi.nlm.nih.gov/pubmed/19259985) | [RUNX2](https://www.genecards.org/Search/Keyword?queryString=RUNX2) |
| S100A1 | Secreted Factors | 24833748 | [24833748](https://www.ncbi.nlm.nih.gov/pubmed/24833748) | [S100A1](https://www.genecards.org/Search/Keyword?queryString=S100A1) |
| S100A10 | Secreted Factors | 14570893 | [14570893](https://www.ncbi.nlm.nih.gov/pubmed/14570893) | [S100A10](https://www.genecards.org/Search/Keyword?queryString=S100A10) |
| S100A11 | Secreted Factors | 27988214 | [27988214](https://www.ncbi.nlm.nih.gov/pubmed/27988214) | [S100A11](https://www.genecards.org/Search/Keyword?queryString=S100A11) |
| S100A12 | Secreted Factors | 27988214 | [27988214](https://www.ncbi.nlm.nih.gov/pubmed/27988214) | [S100A12](https://www.genecards.org/Search/Keyword?queryString=S100A12) |
| S100A13 | Secreted Factors | 22159717 | [22159717](https://www.ncbi.nlm.nih.gov/pubmed/22159717) | [S100A13](https://www.genecards.org/Search/Keyword?queryString=S100A13) |
| S100A14 | Secreted Factors | 27988214 | [27988214](https://www.ncbi.nlm.nih.gov/pubmed/27988214) | [S100A14](https://www.genecards.org/Search/Keyword?queryString=S100A14) |
| S100A16 | Secreted Factors | 24660101 | [24660101](https://www.ncbi.nlm.nih.gov/pubmed/ 24660101) | [S100A16](https://www.genecards.org/Search/Keyword?queryString=S100A16) |
| S100A2 | Secreted Factors | 23996929 | [23996929](https://www.ncbi.nlm.nih.gov/pubmed/23996929) | [S100A2](https://www.genecards.org/Search/Keyword?queryString=S100A2) |
| S100A3 | Secreted Factors | 24660101 | [24660101](https://www.ncbi.nlm.nih.gov/pubmed/24660101) | [S100A3](https://www.genecards.org/Search/Keyword?queryString=S100A3) |
| S100A4 | Secreted Factors | 19956863 | [19956863](https://www.ncbi.nlm.nih.gov/pubmed/19956863) | [S100A4](https://www.genecards.org/Search/Keyword?queryString=S100A4) |
| S100A5 | Secreted Factors | 24660101 | [24660101](https://www.ncbi.nlm.nih.gov/pubmed/24660101) | [S100A5](https://www.genecards.org/Search/Keyword?queryString=S100A5) |
| S100A6 | Secreted Factors | 24660101 | [24660101](https://www.ncbi.nlm.nih.gov/pubmed/24660101) | [S100A6](https://www.genecards.org/Search/Keyword?queryString=S100A6) |
| S100A7 | Secreted Factors | 24660101 | [24660101](https://www.ncbi.nlm.nih.gov/pubmed/24660101) | [S100A7](https://www.genecards.org/Search/Keyword?queryString=S100A7) |
| S100A7A | Secreted Factors | 24660101 | [24660101](https://www.ncbi.nlm.nih.gov/pubmed/24660101) | [S100A7A](https://www.genecards.org/Search/Keyword?queryString=S100A7A) |
| S100A7L2 | Secreted Factors | 22159717 | [22159717](https://www.ncbi.nlm.nih.gov/pubmed/22159717) | [S100A7L2](https://www.genecards.org/Search/Keyword?queryString=S100A7L2) |
| S100A8 | Secreted Factors | 24660101 | [24660101](https://www.ncbi.nlm.nih.gov/pubmed/24660101) | [S100A8](https://www.genecards.org/Search/Keyword?queryString=S100A8) |
| S100A9 | Secreted Factors | 24660101 | [24660101](https://www.ncbi.nlm.nih.gov/pubmed/24660101) | [S100A9](https://www.genecards.org/Search/Keyword?queryString=S100A9) |
| S100B | Secreted Factors | 24660101 | [24660101](https://www.ncbi.nlm.nih.gov/pubmed/24660101) | [S100B](https://www.genecards.org/Search/Keyword?queryString=S100B) |
| S100G | Secreted Factors | 25049477 | [25049477](https://www.ncbi.nlm.nih.gov/pubmed/25049477) | [S100G](https://www.genecards.org/Search/Keyword?queryString=S100G) |
| S100P | Secreted Factors | 24660101 | [24660101](https://www.ncbi.nlm.nih.gov/pubmed/24660101) | [S100P](https://www.genecards.org/Search/Keyword?queryString=S100P) |
| S100Z | Secreted Factors | 24660101 | [24660101](https://www.ncbi.nlm.nih.gov/pubmed/24660101) | [S100Z](https://www.genecards.org/Search/Keyword?queryString=S100Z) |
| SBSPON | ECM Glycoproteins | 27988214 | [27988214](https://www.ncbi.nlm.nih.gov/pubmed/27988214) | [SBSPON](https://www.genecards.org/Search/Keyword?queryString=SBSPON) |
| SCG2 | ECM | 9473216 | [9473216](https://www.ncbi.nlm.nih.gov/pubmed/9473216) | [SCG2](https://www.genecards.org/Search/Keyword?queryString=SCG2) |
| SCG5 | ECM | 1619632 | [1619632](https://www.ncbi.nlm.nih.gov/pubmed/1619632) | [SCG5](https://www.genecards.org/Search/Keyword?queryString=SCG5) |
| SCUBE1 | Secreted Factors | 16753137 | [16753137](https://www.ncbi.nlm.nih.gov/pubmed/16753137) | [SCUBE1](https://www.genecards.org/Search/Keyword?queryString=SCUBE1) |
| SCUBE2 | Secreted Factors | 16753137 | [16753137](https://www.ncbi.nlm.nih.gov/pubmed/16753137) | [SCUBE2](https://www.genecards.org/Search/Keyword?queryString=SCUBE2) |
| SCUBE3 | Secreted Factors | 16753137 | [16753137](https://www.ncbi.nlm.nih.gov/pubmed/16753137) | [SCUBE3](https://www.genecards.org/Search/Keyword?queryString=SCUBE3) |
| SCX | ECM associated regulatory factors | 22796342 | [22796342](https://www.ncbi.nlm.nih.gov/pubmed/22796342) | [SCX](https://www.genecards.org/Search/Keyword?queryString=SCX) |
| SDC1 | ECM-affiliated Proteins | 26909794 | [26909794](https://www.ncbi.nlm.nih.gov/pubmed/26909794) | [SDC1](https://www.genecards.org/Search/Keyword?queryString=SDC1) |
| SDC2 | ECM-affiliated Proteins | 22900087 | [22900087](https://www.ncbi.nlm.nih.gov/pubmed/22900087) | [SDC2](https://www.genecards.org/Search/Keyword?queryString=SDC2) |
| SDC3 | ECM-affiliated Proteins | 23133440 | [23133440](https://www.ncbi.nlm.nih.gov/pubmed/23133440) | [SDC3](https://www.genecards.org/Search/Keyword?queryString=SDC3) |
| SDC4 | ECM-affiliated Proteins | 23133440 | [23133440](https://www.ncbi.nlm.nih.gov/pubmed/23133440) | [SDC4](https://www.genecards.org/Search/Keyword?queryString=SDC4) |
| SELE | ECM | 14525970 | [14525970](https://www.ncbi.nlm.nih.gov/pubmed/14525970) | [SELE](https://www.genecards.org/Search/Keyword?queryString=SELE) |
| SELL | ECM | 19088176 | [19088176](https://www.ncbi.nlm.nih.gov/pubmed/19088176) | [SELL](https://www.genecards.org/Search/Keyword?queryString=SELL) |
| SELP | ECM | 10861096 | [10861096](https://www.ncbi.nlm.nih.gov/pubmed/10861096) | [SELP](https://www.genecards.org/Search/Keyword?queryString=SELP) |
| SELPLG | ECM | 7505206 | [7505206](https://www.ncbi.nlm.nih.gov/pubmed/7505206) | [SELPLG](https://www.genecards.org/Search/Keyword?queryString=SELPLG) |
| SEMA3A | ECM-affiliated Proteins | 23028146 | [23028146](https://www.ncbi.nlm.nih.gov/pubmed/23028146) | [SEMA3A](https://www.genecards.org/Search/Keyword?queryString=SEMA3A) |
| SEMA3B | ECM-affiliated Proteins | 21933904 | [21933904](https://www.ncbi.nlm.nih.gov/pubmed/21933904) | [SEMA3B](https://www.genecards.org/Search/Keyword?queryString=SEMA3B) |
| SEMA3C | ECM-affiliated Proteins | 23666167 | [23666167](https://www.ncbi.nlm.nih.gov/pubmed/23666167) | [SEMA3C](https://www.genecards.org/Search/Keyword?queryString=SEMA3C) |
| SEMA3D | ECM-affiliated Proteins | 26828861 | [26828861](https://www.ncbi.nlm.nih.gov/pubmed/26828861) | [SEMA3D](https://www.genecards.org/Search/Keyword?queryString=SEMA3D) |
| SEMA3E | ECM-affiliated Proteins | 20385769 | [20385769](https://www.ncbi.nlm.nih.gov/pubmed/20385769) | [SEMA3E](https://www.genecards.org/Search/Keyword?queryString=SEMA3E) |
| SEMA3F | ECM-affiliated Proteins | 21933904 | [21933904](https://www.ncbi.nlm.nih.gov/pubmed/21933904) | [SEMA3F](https://www.genecards.org/Search/Keyword?queryString=SEMA3F) |
| SEMA3G | ECM-affiliated Proteins | 22562223 | [22562223](https://www.ncbi.nlm.nih.gov/pubmed/22562223) | [SEMA3G](https://www.genecards.org/Search/Keyword?queryString=SEMA3G) |
| SEMA4A | ECM-affiliated Proteins | 26303122 | [26303122](https://www.ncbi.nlm.nih.gov/pubmed/26303122) | [SEMA4A](https://www.genecards.org/Search/Keyword?queryString=SEMA4A) |
| SEMA4B | ECM-affiliated Proteins | 22132705 | [22132705](https://www.ncbi.nlm.nih.gov/pubmed/22132705) | [SEMA4B](https://www.genecards.org/Search/Keyword?queryString=SEMA4B) |
| SEMA4C | ECM-affiliated Proteins | 20959347 | [20959347](https://www.ncbi.nlm.nih.gov/pubmed/20959347) | [SEMA4C](https://www.genecards.org/Search/Keyword?queryString=SEMA4C) |
| SEMA4D | ECM-affiliated Proteins | 16702230 | [16702230](https://www.ncbi.nlm.nih.gov/pubmed/16702230) | [SEMA4D](https://www.genecards.org/Search/Keyword?queryString=SEMA4D) |
| SEMA4F | ECM-affiliated Proteins | 22159717 | [22159717](https://www.ncbi.nlm.nih.gov/pubmed/22159717) | [SEMA4F](https://www.genecards.org/Search/Keyword?queryString=SEMA4F) |
| SEMA4G | ECM-affiliated Proteins | 28839176 | [28839176](https://www.ncbi.nlm.nih.gov/pubmed/28839176) | [SEMA4G](https://www.genecards.org/Search/Keyword?queryString=SEMA4G) |
| SEMA5A | ECM-affiliated Proteins | 19850054 | [19850054](https://www.ncbi.nlm.nih.gov/pubmed/19850054) | [SEMA5A](https://www.genecards.org/Search/Keyword?queryString=SEMA5A) |
| SEMA5B | ECM-affiliated Proteins | 22817385 | [22817385](https://www.ncbi.nlm.nih.gov/pubmed/22817385) | [SEMA5B](https://www.genecards.org/Search/Keyword?queryString=SEMA5B) |
| SEMA6A | ECM-affiliated Proteins | 20631206 | [20631206](https://www.ncbi.nlm.nih.gov/pubmed/20631206) | [SEMA6A](https://www.genecards.org/Search/Keyword?queryString=SEMA6A) |
| SEMA6B | ECM-affiliated Proteins | 20631206 | [20631206](https://www.ncbi.nlm.nih.gov/pubmed/20631206) | [SEMA6B](https://www.genecards.org/Search/Keyword?queryString=SEMA6B) |
| SEMA6C | ECM-affiliated Proteins | 20631206 | [20631206](https://www.ncbi.nlm.nih.gov/pubmed/20631206) | [SEMA6C](https://www.genecards.org/Search/Keyword?queryString=SEMA6C) |
| SEMA6D | ECM-affiliated Proteins | 20631206 | [20631206](https://www.ncbi.nlm.nih.gov/pubmed/20631206) | [SEMA6D](https://www.genecards.org/Search/Keyword?queryString=SEMA6D) |
| SEMA7A | ECM-affiliated Proteins | 20631206 | [20631206](https://www.ncbi.nlm.nih.gov/pubmed/20631206) | [SEMA7A](https://www.genecards.org/Search/Keyword?queryString=SEMA7A) |
| SERPINA1 | ECM Regulators | 16737556 | [16737556](https://www.ncbi.nlm.nih.gov/pubmed/16737556) | [SERPINA1](https://www.genecards.org/Search/Keyword?queryString=SERPINA1) |
| SERPINA10 | ECM Regulators | 16737556 | [16737556](https://www.ncbi.nlm.nih.gov/pubmed/16737556) | [SERPINA10](https://www.genecards.org/Search/Keyword?queryString=SERPINA10) |
| SERPINA11 | ECM Regulators | 16737556 | [16737556](https://www.ncbi.nlm.nih.gov/pubmed/16737556) | [SERPINA11](https://www.genecards.org/Search/Keyword?queryString=SERPINA11) |
| SERPINA12 | ECM Regulators | 16737556 | [16737556](https://www.ncbi.nlm.nih.gov/pubmed/16737556) | [SERPINA12](https://www.genecards.org/Search/Keyword?queryString=SERPINA12) |
| SERPINA2 | ECM Regulators | 16737556 | [16737556](https://www.ncbi.nlm.nih.gov/pubmed/16737556) | [SERPINA2](https://www.genecards.org/Search/Keyword?queryString=SERPINA2) |
| SERPINA3 | ECM Regulators | 16737556 | [16737556](https://www.ncbi.nlm.nih.gov/pubmed/16737556) | [SERPINA3](https://www.genecards.org/Search/Keyword?queryString=SERPINA3) |
| SERPINA4 | ECM Regulators | 16737556 | [16737556](https://www.ncbi.nlm.nih.gov/pubmed/16737556) | [SERPINA4](https://www.genecards.org/Search/Keyword?queryString=SERPINA4) |
| SERPINA5 | ECM Regulators | 16737556 | [16737556](https://www.ncbi.nlm.nih.gov/pubmed/16737556) | [SERPINA5](https://www.genecards.org/Search/Keyword?queryString=SERPINA5) |
| SERPINA6 | ECM Regulators | 16737556 | [16737556](https://www.ncbi.nlm.nih.gov/pubmed/16737556) | [SERPINA6](https://www.genecards.org/Search/Keyword?queryString=SERPINA6) |
| SERPINA7 | ECM Regulators | 16737556 | [16737556](https://www.ncbi.nlm.nih.gov/pubmed/16737556) | [SERPINA7](https://www.genecards.org/Search/Keyword?queryString=SERPINA7) |
| SERPINA9 | ECM Regulators | 16737556 | [16737556](https://www.ncbi.nlm.nih.gov/pubmed/16737556) | [SERPINA9](https://www.genecards.org/Search/Keyword?queryString=SERPINA9) |
| SERPINB1 | ECM Regulators | 16737556 | [16737556](https://www.ncbi.nlm.nih.gov/pubmed/16737556) | [SERPINB1](https://www.genecards.org/Search/Keyword?queryString=SERPINB1) |
| SERPINB10 | ECM Regulators | 16737556 | [16737556](https://www.ncbi.nlm.nih.gov/pubmed/16737556) | [SERPINB10](https://www.genecards.org/Search/Keyword?queryString=SERPINB10) |
| SERPINB11 | ECM Regulators | 16737556 | [16737556](https://www.ncbi.nlm.nih.gov/pubmed/16737556) | [SERPINB11](https://www.genecards.org/Search/Keyword?queryString=SERPINB11) |
| SERPINB12 | ECM Regulators | 16737556 | [16737556](https://www.ncbi.nlm.nih.gov/pubmed/16737556) | [SERPINB12](https://www.genecards.org/Search/Keyword?queryString=SERPINB12) |
| SERPINB13 | ECM Regulators | 16737556 | [16737556](https://www.ncbi.nlm.nih.gov/pubmed/16737556) | [SERPINB13](https://www.genecards.org/Search/Keyword?queryString=SERPINB13) |
| SERPINB2 | ECM Regulators | 16737556 | [16737556](https://www.ncbi.nlm.nih.gov/pubmed/16737556) | [SERPINB2](https://www.genecards.org/Search/Keyword?queryString=SERPINB2) |
| SERPINB3 | ECM Regulators | 16737556 | [16737556](https://www.ncbi.nlm.nih.gov/pubmed/16737556) | [SERPINB3](https://www.genecards.org/Search/Keyword?queryString=SERPINB3) |
| SERPINB4 | ECM Regulators | 16737556 | [16737556](https://www.ncbi.nlm.nih.gov/pubmed/16737556) | [SERPINB4](https://www.genecards.org/Search/Keyword?queryString=SERPINB4) |
| SERPINB5 | ECM Regulators | 16737556 | [16737556](https://www.ncbi.nlm.nih.gov/pubmed/16737556) | [SERPINB5](https://www.genecards.org/Search/Keyword?queryString=SERPINB5) |
| SERPINB6 | ECM Regulators | 16737556 | [16737556](https://www.ncbi.nlm.nih.gov/pubmed/16737556) | [SERPINB6](https://www.genecards.org/Search/Keyword?queryString=SERPINB6) |
| SERPINB7 | ECM Regulators | 16737556 | [16737556](https://www.ncbi.nlm.nih.gov/pubmed/16737556) | [SERPINB7](https://www.genecards.org/Search/Keyword?queryString=SERPINB7) |
| SERPINB8 | ECM Regulators | 16737556 | [16737556](https://www.ncbi.nlm.nih.gov/pubmed/16737556) | [SERPINB8](https://www.genecards.org/Search/Keyword?queryString=SERPINB8) |
| SERPINB9 | ECM Regulators | 16737556 | [16737556](https://www.ncbi.nlm.nih.gov/pubmed/16737556) | [SERPINB9](https://www.genecards.org/Search/Keyword?queryString=SERPINB9) |
| SERPINC1 | ECM Regulators | 16737556 | [16737556](https://www.ncbi.nlm.nih.gov/pubmed/16737556) | [SERPINC1](https://www.genecards.org/Search/Keyword?queryString=SERPINC1) |
| SERPIND1 | ECM Regulators | 16737556 | [16737556](https://www.ncbi.nlm.nih.gov/pubmed/16737556) | [SERPIND1](https://www.genecards.org/Search/Keyword?queryString=SERPIND1) |
| SERPINE1 | ECM Regulators | 16737556 | [16737556](https://www.ncbi.nlm.nih.gov/pubmed/16737556) | [SERPINE1](https://www.genecards.org/Search/Keyword?queryString=SERPINE1) |
| SERPINE2 | ECM Regulators | 16737556 | [16737556](https://www.ncbi.nlm.nih.gov/pubmed/16737556) | [SERPINE2](https://www.genecards.org/Search/Keyword?queryString=SERPINE2) |
| SERPINE3 | ECM Regulators | 16737556 | [16737556](https://www.ncbi.nlm.nih.gov/pubmed/16737556) | [SERPINE3](https://www.genecards.org/Search/Keyword?queryString=SERPINE3) |
| SERPINF1 | ECM Regulators | 16737556 | [16737556](https://www.ncbi.nlm.nih.gov/pubmed/16737556) | [SERPINF1](https://www.genecards.org/Search/Keyword?queryString=SERPINF1) |
| SERPINF2 | ECM Regulators | 16737556 | [16737556](https://www.ncbi.nlm.nih.gov/pubmed/16737556) | [SERPINF2](https://www.genecards.org/Search/Keyword?queryString=SERPINF2) |
| SERPING1 | ECM Regulators | 16737556 | [16737556](https://www.ncbi.nlm.nih.gov/pubmed/16737556) | [SERPING1](https://www.genecards.org/Search/Keyword?queryString=SERPING1) |
| SERPINH1 | ECM Regulators | 16737556 | [16737556](https://www.ncbi.nlm.nih.gov/pubmed/16737556) | [SERPINH1](https://www.genecards.org/Search/Keyword?queryString=SERPINH1) |
| SERPINI1 | ECM Regulators | 16737556 | [16737556](https://www.ncbi.nlm.nih.gov/pubmed/16737556) | [SERPINI1](https://www.genecards.org/Search/Keyword?queryString=SERPINI1) |
| SERPINI2 | ECM Regulators | 16737556 | [16737556](https://www.ncbi.nlm.nih.gov/pubmed/16737556) | [SERPINI2](https://www.genecards.org/Search/Keyword?queryString=SERPINI2) |
| SFRP1 | Secreted Factors | 22728933 | [22728933](https://www.ncbi.nlm.nih.gov/pubmed/ 22728933) | [SFRP1](https://www.genecards.org/Search/Keyword?queryString=SFRP1) |
| SFRP2 | Secreted Factors | 22728933 | [22728933](https://www.ncbi.nlm.nih.gov/pubmed/ 22728933) | [SFRP2](https://www.genecards.org/Search/Keyword?queryString=SFRP2) |
| SFRP4 | Secreted Factors | 22728933 | [22728933](https://www.ncbi.nlm.nih.gov/pubmed/ 22728933) | [SFRP4](https://www.genecards.org/Search/Keyword?queryString=SFRP4) |
| SFRP5 | Secreted Factors | 22728933 | [22728933](https://www.ncbi.nlm.nih.gov/pubmed/ 22728933) | [SFRP5](https://www.genecards.org/Search/Keyword?queryString=SFRP5) |
| SFTA2 | ECM-affiliated Proteins | 22768197 | [22768197](https://www.ncbi.nlm.nih.gov/pubmed/22768197) | [SFTA2](https://www.genecards.org/Search/Keyword?queryString=SFTA2) |
| SFTA3 | ECM-affiliated Proteins | 28849102 | [28849102](https://www.ncbi.nlm.nih.gov/pubmed/28849102) | [SFTA3](https://www.genecards.org/Search/Keyword?queryString=SFTA3) |
| SFTPA1 | ECM-affiliated Proteins | 23419153 | [23419153](https://www.ncbi.nlm.nih.gov/pubmed/23419153) | [SFTPA1](https://www.genecards.org/Search/Keyword?queryString=SFTPA1) |
| SFTPA2 | ECM-affiliated Proteins | 29170406 | [29170406](https://www.ncbi.nlm.nih.gov/pubmed/29170406) | [SFTPA2](https://www.genecards.org/Search/Keyword?queryString=SFTPA2) |
| SFTPB | ECM-affiliated Proteins | 27736644 | [27736644](https://www.ncbi.nlm.nih.gov/pubmed/27736644) | [SFTPB](https://www.genecards.org/Search/Keyword?queryString=SFTPB) |
| SFTPC | ECM-affiliated Proteins | 27736644 | [27736644](https://www.ncbi.nlm.nih.gov/pubmed/27736644) | [SFTPC](https://www.genecards.org/Search/Keyword?queryString=SFTPC) |
| SFTPD | ECM-affiliated Proteins | 29170406 | [29170406](https://www.ncbi.nlm.nih.gov/pubmed/29170406) | [SFTPD](https://www.genecards.org/Search/Keyword?queryString=SFTPD) |
| SGCA | ECM | 10684260 | [10684260](https://www.ncbi.nlm.nih.gov/pubmed/10684260) | [SGCA](https://www.genecards.org/Search/Keyword?queryString=SGCA) |
| SGSH | ECM synthetic/degradation enzyme | 15146460 | [15146460](https://www.ncbi.nlm.nih.gov/pubmed/15146460) | [SGSH](https://www.genecards.org/Search/Keyword?queryString=SGSH) |
| SHH | Secreted Factors | 23313125 | [23313125](https://www.ncbi.nlm.nih.gov/pubmed/23313125) | [SHH](https://www.genecards.org/Search/Keyword?queryString=SHH) |
| SLC1A3 | ECM Micro-enviornment associated | 12684467 | [12684467](https://www.ncbi.nlm.nih.gov/pubmed/12684467) | [SLC1A3](https://www.genecards.org/Search/Keyword?queryString=SLC1A3) |
| SLC26A2 | ECM Micro-enviornment associated | 14692227 | [14692227](https://www.ncbi.nlm.nih.gov/pubmed/14692227) | [SLC26A2](https://www.genecards.org/Search/Keyword?queryString=SLC26A2) |
| SLC35B2 | ECM Micro-enviornment associated | 12716889 | [12716889](https://www.ncbi.nlm.nih.gov/pubmed/12716889) | [SLC35B2](https://www.genecards.org/Search/Keyword?queryString=SLC35B2) |
| SLC35B4 | ECM Micro-enviornment associated | 15911612 | [15911612](https://www.ncbi.nlm.nih.gov/pubmed/15911612) | [SLC35B4](https://www.genecards.org/Search/Keyword?queryString=SLC35B4) |
| SLC9A1 | ECM Micro-enviornment associated | 21423176 | [21423176](https://www.ncbi.nlm.nih.gov/pubmed/21423176) | [SLC9A1](https://www.genecards.org/Search/Keyword?queryString=SLC9A1) |
| SLIT1 | ECM Glycoproteins | 21937732 | [21937732](https://www.ncbi.nlm.nih.gov/pubmed/21937732) | [SLIT1](https://www.genecards.org/Search/Keyword?queryString=SLIT1) |
| SLIT2 | ECM Glycoproteins | 21937732 | [21937732](https://www.ncbi.nlm.nih.gov/pubmed/21937732) | [SLIT2](https://www.genecards.org/Search/Keyword?queryString=SLIT2) |
| SLIT3 | ECM Glycoproteins | 21937732 | [21937732](https://www.ncbi.nlm.nih.gov/pubmed/21937732) | [SLIT3](https://www.genecards.org/Search/Keyword?queryString=SLIT3) |
| SLPI | ECM Regulators | 21687692 | [21687692](https://www.ncbi.nlm.nih.gov/pubmed/21687692) | [SLPI](https://www.genecards.org/Search/Keyword?queryString=SLPI) |
| SMAD1 | ECM Micro-enviornment associated | 16226436 | [16226436](https://www.ncbi.nlm.nih.gov/pubmed/16226436) | [SMAD1](https://www.genecards.org/Search/Keyword?queryString=SMAD1) |
| SMAD2 | ECM Micro-enviornment associated | 22999704 | [22999704](https://www.ncbi.nlm.nih.gov/pubmed/22999704) | [SMAD2](https://www.genecards.org/Search/Keyword?queryString=SMAD2) |
| SMAD3 | ECM Micro-enviornment associated | 11241356 | [11241356](https://www.ncbi.nlm.nih.gov/pubmed/11241356) | [SMAD3](https://www.genecards.org/Search/Keyword?queryString=SMAD3) |
| SMAD5 | ECM Micro-enviornment associated | 16226436 | [16226436](https://www.ncbi.nlm.nih.gov/pubmed/16226436) | [SMAD5](https://www.genecards.org/Search/Keyword?queryString=SMAD5) |
| SMAD7 | ECM Micro-enviornment associated | 18593713 | [18593713](https://www.ncbi.nlm.nih.gov/pubmed/18593713) | [SMAD7](https://www.genecards.org/Search/Keyword?queryString=SMAD7) |
| SMC3 | ECM associated regulatory factors | 19444697 | [19444697](https://www.ncbi.nlm.nih.gov/pubmed/19444697) | [SMC3](https://www.genecards.org/Search/Keyword?queryString=SMC3) |
| SMO | ECM receptor | 19056867 | [19056867](https://www.ncbi.nlm.nih.gov/pubmed/19056867) | [SMO](https://www.genecards.org/Search/Keyword?queryString=SMO) |
| SMOC1 | ECM Glycoproteins | 21937732 | [21937732](https://www.ncbi.nlm.nih.gov/pubmed/21937732) | [SMOC1](https://www.genecards.org/Search/Keyword?queryString=SMOC1) |
| SMOC2 | ECM Glycoproteins | 21937732 | [21937732](https://www.ncbi.nlm.nih.gov/pubmed/21937732) | [SMOC2](https://www.genecards.org/Search/Keyword?queryString=SMOC2) |
| SMTN | ECM | 24043717 | [24043717](https://www.ncbi.nlm.nih.gov/pubmed/24043717) | [SMTN](https://www.genecards.org/Search/Keyword?queryString=SMTN) |
| SNED1 | ECM Glycoproteins | 21937732 | [21937732](https://www.ncbi.nlm.nih.gov/pubmed/21937732) | [SNED1](https://www.genecards.org/Search/Keyword?queryString=SNED1) |
| SOD1 | ECM Micro-enviornment associated | 7172448 | [7172448](https://www.ncbi.nlm.nih.gov/pubmed/7172448) | [SOD1](https://www.genecards.org/Search/Keyword?queryString=SOD1) |
| SOD3 | ECM Micro-enviornment associated | 20551380 | [20551380](https://www.ncbi.nlm.nih.gov/pubmed/20551380) | [SOD3](https://www.genecards.org/Search/Keyword?queryString=SOD3) |
| SOS1 | ECM associated regulatory factors | 21048031 | [21048031](https://www.ncbi.nlm.nih.gov/pubmed/21048031) | [SOS1](https://www.genecards.org/Search/Keyword?queryString=SOS1) |
| SOS2 | ECM associated regulatory factors | 25227104 | [25227104](https://www.ncbi.nlm.nih.gov/pubmed/25227104) | [SOS2](https://www.genecards.org/Search/Keyword?queryString=SOS2) |
| SOX9 | ECM associated regulatory factors | 15922909 | [15922909](https://www.ncbi.nlm.nih.gov/pubmed/15922909) | [SOX9](https://www.genecards.org/Search/Keyword?queryString=SOX9) |
| SP1 | ECM associated regulatory factors | 10506168 | [10506168](https://www.ncbi.nlm.nih.gov/pubmed/10506168) | [SP1](https://www.genecards.org/Search/Keyword?queryString=SP1) |
| SP3 | ECM associated regulatory factors | 16229994 | [16229994](https://www.ncbi.nlm.nih.gov/pubmed/16229994) | [SP3](https://www.genecards.org/Search/Keyword?queryString=SP3) |
| SPAM1 | ECM Regulators | 12932297 | [12932297](https://www.ncbi.nlm.nih.gov/pubmed/12932297) | [SPAM1](https://www.genecards.org/Search/Keyword?queryString=SPAM1) |
| SPARC | ECM Glycoproteins | 23419153 | [23419153](https://www.ncbi.nlm.nih.gov/pubmed/23419153) | [SPARC](https://www.genecards.org/Search/Keyword?queryString=SPARC) |
| SPARCL1 | ECM Glycoproteins | 21937732 | [21937732](https://www.ncbi.nlm.nih.gov/pubmed/21937732) | [SPARCL1](https://www.genecards.org/Search/Keyword?queryString=SPARCL1) |
| SPOCK1 | Proteoglycans | 23419153 | [23419153](https://www.ncbi.nlm.nih.gov/pubmed/23419153) | [SPOCK1](https://www.genecards.org/Search/Keyword?queryString=SPOCK1) |
| SPOCK2 | Proteoglycans | 23419153 | [23419153](https://www.ncbi.nlm.nih.gov/pubmed/23419153) | [SPOCK2](https://www.genecards.org/Search/Keyword?queryString=SPOCK2) |
| SPOCK3 | Proteoglycans | 23419153 | [23419153](https://www.ncbi.nlm.nih.gov/pubmed/23419153) | [SPOCK3](https://www.genecards.org/Search/Keyword?queryString=SPOCK3) |
| SPON1 | ECM Glycoproteins | 21937732 | [21937732](https://www.ncbi.nlm.nih.gov/pubmed/21937732) | [SPON1](https://www.genecards.org/Search/Keyword?queryString=SPON1) |
| SPON2 | ECM Glycoproteins | 21937732 | [21937732](https://www.ncbi.nlm.nih.gov/pubmed/21937732) | [SPON2](https://www.genecards.org/Search/Keyword?queryString=SPON2) |
| SPP1 | ECM Glycoproteins | 12646701 | [12646701](https://www.ncbi.nlm.nih.gov/pubmed/12646701) | [SPP1](https://www.genecards.org/Search/Keyword?queryString=SPP1) |
| SRC | ECM Micro-enviornment associated | 19056867 | [19056867](https://www.ncbi.nlm.nih.gov/pubmed/19056867) | [SRC](https://www.genecards.org/Search/Keyword?queryString=SRC) |
| SRF | ECM | 21842128 | [21842128](https://www.ncbi.nlm.nih.gov/pubmed/21842128) | [SRF](https://www.genecards.org/Search/Keyword?queryString=SRF) |
| SRGN | Proteoglycans | 26290530 | [26290530](https://www.ncbi.nlm.nih.gov/pubmed/26290530) | [SRGN](https://www.genecards.org/Search/Keyword?queryString=SRGN) |
| SRI | ECM | 15326289 | [15326289](https://www.ncbi.nlm.nih.gov/pubmed/15326289) | [SRI](https://www.genecards.org/Search/Keyword?queryString=SRI) |
| SRPX | ECM Glycoproteins | 21937732 | [21937732](https://www.ncbi.nlm.nih.gov/pubmed/21937732) | [SRPX](https://www.genecards.org/Search/Keyword?queryString=SRPX) |
| SRPX2 | ECM Glycoproteins | 21937732 | [21937732](https://www.ncbi.nlm.nih.gov/pubmed/21937732) | [SRPX2](https://www.genecards.org/Search/Keyword?queryString=SRPX2) |
| SSPO | ECM Glycoproteins | 21937732 | [21937732](https://www.ncbi.nlm.nih.gov/pubmed/21937732) | [SSPO](https://www.genecards.org/Search/Keyword?queryString=SSPO) |
| SST | ECM | 9437026 | [9437026](https://www.ncbi.nlm.nih.gov/pubmed/9437026) | [SST](https://www.genecards.org/Search/Keyword?queryString=SST) |
| ST14 | ECM Regulators | 27167193 | [27167193](https://www.ncbi.nlm.nih.gov/pubmed/27167193) | [ST14](https://www.genecards.org/Search/Keyword?queryString=ST14) |
| STAT3 | ECM associated regulatory factors | 16619044 | [16619044](https://www.ncbi.nlm.nih.gov/pubmed/16619044) | [STAT3](https://www.genecards.org/Search/Keyword?queryString=STAT3) |
| SULF1 | ECM Regulators | 19373441 | [19373441](https://www.ncbi.nlm.nih.gov/pubmed/19373441) | [SULF1](https://www.genecards.org/Search/Keyword?queryString=SULF1) |
| SULF2 | ECM Regulators | 19373441 | [19373441](https://www.ncbi.nlm.nih.gov/pubmed/19373441) | [SULF2](https://www.genecards.org/Search/Keyword?queryString=SULF2) |
| SUMF1 | ECM synthetic/degradation enzyme | 18832069 | [18832069](https://www.ncbi.nlm.nih.gov/pubmed/18832069) | [SUMF1](https://www.genecards.org/Search/Keyword?queryString=SUMF1) |
| SVEP1 | ECM Glycoproteins | 21937732 | [21937732](https://www.ncbi.nlm.nih.gov/pubmed/21937732) | [SVEP1](https://www.genecards.org/Search/Keyword?queryString=SVEP1) |
| SYT1 | ECM | 11438518 | [11438518](https://www.ncbi.nlm.nih.gov/pubmed/11438518) | [SYT1](https://www.genecards.org/Search/Keyword?queryString=SYT1) |
| TAT | ECM Micro-enviornment associated | 11024024 | [11024024](https://www.ncbi.nlm.nih.gov/pubmed/11024024) | [TAT](https://www.genecards.org/Search/Keyword?queryString=TAT) |
| TCF7L2 | ECM associated regulatory factors | 11931652 | [11931652](https://www.ncbi.nlm.nih.gov/pubmed/11931652) | [TCF7L2](https://www.genecards.org/Search/Keyword?queryString=TCF7L2) |
| TCHH | Secreted Factors | 29220522 | [29220522](https://www.ncbi.nlm.nih.gov/pubmed/29220522) | [TCHH](https://www.genecards.org/Search/Keyword?queryString=TCHH) |
| TCHHL1 | Secreted Factors | 29220522 | [29220522](https://www.ncbi.nlm.nih.gov/pubmed/29220522) | [TCHHL1](https://www.genecards.org/Search/Keyword?queryString=TCHHL1) |
| TDGF1 | Secreted Factors | 21937732 | [21937732](https://www.ncbi.nlm.nih.gov/pubmed/21937732) | [TDGF1](https://www.genecards.org/Search/Keyword?queryString=TDGF1) |
| TECTA | ECM Glycoproteins | 21937732 | [21937732](https://www.ncbi.nlm.nih.gov/pubmed/21937732) | [TECTA](https://www.genecards.org/Search/Keyword?queryString=TECTA) |
| TECTB | ECM Glycoproteins | 21937732 | [21937732](https://www.ncbi.nlm.nih.gov/pubmed/21937732) | [TECTB](https://www.genecards.org/Search/Keyword?queryString=TECTB) |
| TEK | ECM Micro-enviornment associated | 19293632 | [19293632](https://www.ncbi.nlm.nih.gov/pubmed/19293632) | [TEK](https://www.genecards.org/Search/Keyword?queryString=TEK) |
| TFAP4 | ECM associated regulatory factors | 23186163 | [23186163](https://www.ncbi.nlm.nih.gov/pubmed/23186163) | [TFAP4](https://www.genecards.org/Search/Keyword?queryString=TFAP4) |
| TGFA | Secreted Factors | 22864984 | [22864984](https://www.ncbi.nlm.nih.gov/pubmed/22864984) | [TGFA](https://www.genecards.org/Search/Keyword?queryString=TGFA) |
| TGFB1 | Secreted Factors | 8416992 | [8416992](https://www.ncbi.nlm.nih.gov/pubmed/8416992) | [TGFB1](https://www.genecards.org/Search/Keyword?queryString=TGFB1) |
| TGFB2 | Secreted Factors | 8416992 | [8416992](https://www.ncbi.nlm.nih.gov/pubmed/8416992) | [TGFB2](https://www.genecards.org/Search/Keyword?queryString=TGFB2) |
| TGFB3 | Secreted Factors | 21604386 | [21604386](https://www.ncbi.nlm.nih.gov/pubmed/21604386) | [TGFB3](https://www.genecards.org/Search/Keyword?queryString=TGFB3) |
| TGFBI | ECM Glycoproteins | 21937732 | [21937732](https://www.ncbi.nlm.nih.gov/pubmed/21937732) | [TGFBI](https://www.genecards.org/Search/Keyword?queryString=TGFBI) |
| TGFBR1 | Cell surface receptor | 16947635 | [16947635](https://www.ncbi.nlm.nih.gov/pubmed/16947635) | [TGFBR1](https://www.genecards.org/Search/Keyword?queryString=TGFBR1) |
| TGFBR3 | Cell surface receptor | 27784788 | [27784788](https://www.ncbi.nlm.nih.gov/pubmed/27784788) | [TGFBR3](https://www.genecards.org/Search/Keyword?queryString=TGFBR3) |
| TGIF1 | ECM Micro-enviornment associated | 18425852 | [18425852](https://www.ncbi.nlm.nih.gov/pubmed/18425852) | [TGIF1](https://www.genecards.org/Search/Keyword?queryString=TGIF1) |
| TGM1 | ECM Regulators | 18235007 | [18235007](https://www.ncbi.nlm.nih.gov/pubmed/18235007) | [TGM1](https://www.genecards.org/Search/Keyword?queryString=TGM1) |
| TGM2 | ECM Regulators | 18235007 | [18235007](https://www.ncbi.nlm.nih.gov/pubmed/18235007) | [TGM2](https://www.genecards.org/Search/Keyword?queryString=TGM2) |
| TGM3 | ECM Regulators | 18235007 | [18235007](https://www.ncbi.nlm.nih.gov/pubmed/18235007) | [TGM3](https://www.genecards.org/Search/Keyword?queryString=TGM3) |
| TGM4 | ECM Regulators | 18235007 | [18235007](https://www.ncbi.nlm.nih.gov/pubmed/18235007) | [TGM4](https://www.genecards.org/Search/Keyword?queryString=TGM4) |
| TGM5 | ECM Regulators | 18235007 | [18235007](https://www.ncbi.nlm.nih.gov/pubmed/18235007) | [TGM5](https://www.genecards.org/Search/Keyword?queryString=TGM5) |
| TGM6 | ECM Regulators | 18235007 | [18235007](https://www.ncbi.nlm.nih.gov/pubmed/18235007) | [TGM6](https://www.genecards.org/Search/Keyword?queryString=TGM6) |
| TGM7 | ECM Regulators | 18235007 | [18235007](https://www.ncbi.nlm.nih.gov/pubmed/18235007) | [TGM7](https://www.genecards.org/Search/Keyword?queryString=TGM7) |
| THBD | ECM | 1332436 | [1332436](https://www.ncbi.nlm.nih.gov/pubmed/1332436) | [THBD](https://www.genecards.org/Search/Keyword?queryString=THBD) |
| THBS1 | ECM Glycoproteins | 23419153 | [23419153](https://www.ncbi.nlm.nih.gov/pubmed/23419153) | [THBS1](https://www.genecards.org/Search/Keyword?queryString=THBS1) |
| THBS2 | ECM Glycoproteins | 21937732 | [21937732](https://www.ncbi.nlm.nih.gov/pubmed/21937732) | [THBS2](https://www.genecards.org/Search/Keyword?queryString=THBS2) |
| THBS3 | ECM Glycoproteins | 21937732 | [21937732](https://www.ncbi.nlm.nih.gov/pubmed/21937732) | [THBS3](https://www.genecards.org/Search/Keyword?queryString=THBS3) |
| THBS4 | ECM Glycoproteins | 21937732 | [21937732](https://www.ncbi.nlm.nih.gov/pubmed/21937732) | [THBS4](https://www.genecards.org/Search/Keyword?queryString=THBS4) |
| THPO | Secreted Factors | 26748484 | [26748484](https://www.ncbi.nlm.nih.gov/pubmed/26748484) | [THPO](https://www.genecards.org/Search/Keyword?queryString=THPO) |
| THSD4 | ECM Glycoproteins | 21937732 | [21937732](https://www.ncbi.nlm.nih.gov/pubmed/21937732) | [THSD4](https://www.genecards.org/Search/Keyword?queryString=THSD4) |
| TIAM1 | ECM Micro-enviornment associated | 20361982 | [20361982](https://www.ncbi.nlm.nih.gov/pubmed/20361982) | [TIAM1](https://www.genecards.org/Search/Keyword?queryString=TIAM1) |
| TICAM1 | ECM receptor cofactor | 22095710 | [22095710](https://www.ncbi.nlm.nih.gov/pubmed/22095710) | [TICAM1](https://www.genecards.org/Search/Keyword?queryString=TICAM1) |
| TIMP1 | ECM Regulators | 17202148 | [17202148](https://www.ncbi.nlm.nih.gov/pubmed/17202148) | [TIMP1](https://www.genecards.org/Search/Keyword?queryString=TIMP1) |
| TIMP2 | ECM Regulators | 17202148 | [17202148](https://www.ncbi.nlm.nih.gov/pubmed/17202148) | [TIMP2](https://www.genecards.org/Search/Keyword?queryString=TIMP2) |
| TIMP3 | ECM Regulators | 17202148 | [17202148](https://www.ncbi.nlm.nih.gov/pubmed/17202148) | [TIMP3](https://www.genecards.org/Search/Keyword?queryString=TIMP3) |
| TIMP4 | ECM Regulators | 17202148 | [17202148](https://www.ncbi.nlm.nih.gov/pubmed/17202148) | [TIMP4](https://www.genecards.org/Search/Keyword?queryString=TIMP4) |
| TINAG | ECM Glycoproteins | 21937732 | [21937732](https://www.ncbi.nlm.nih.gov/pubmed/21937732) | [TINAG](https://www.genecards.org/Search/Keyword?queryString=TINAG) |
| TINAGL1 | ECM Glycoproteins | 21937732 | [21937732](https://www.ncbi.nlm.nih.gov/pubmed/21937732) | [TINAGL1](https://www.genecards.org/Search/Keyword?queryString=TINAGL1) |
| TLL1 | ECM Regulators | 26902455 | [26902455](https://www.ncbi.nlm.nih.gov/pubmed/26902455) | [TLL1](https://www.genecards.org/Search/Keyword?queryString=TLL1) |
| TLL2 | ECM Regulators | 26902455 | [26902455](https://www.ncbi.nlm.nih.gov/pubmed/26902455) | [TLL2](https://www.genecards.org/Search/Keyword?queryString=TLL2) |
| TLR2 | ECM receptor | 19948979 | [19948979](https://www.ncbi.nlm.nih.gov/pubmed/19948979) | [TLR2](https://www.genecards.org/Search/Keyword?queryString=TLR2) |
| TLR4 | ECM receptor | 27124018 | [27124018](https://www.ncbi.nlm.nih.gov/pubmed/27124018) | [TLR4](https://www.genecards.org/Search/Keyword?queryString=TLR4) |
| TMEFF2 | ECM Micro-enviornment associated | 11668495 | [11668495](https://www.ncbi.nlm.nih.gov/pubmed/11668495) | [TMEFF2](https://www.genecards.org/Search/Keyword?queryString=TMEFF2) |
| TMEM2 | ECM Micro-enviornment associated | 28246172 | [28246172](https://www.ncbi.nlm.nih.gov/pubmed/28246172) | [TMEM2](https://www.genecards.org/Search/Keyword?queryString=TMEM2) |
| TMPRSS15 | ECM Regulators | 22159717 | [22159717](https://www.ncbi.nlm.nih.gov/pubmed/22159717) | [TMPRSS15](https://www.genecards.org/Search/Keyword?queryString=TMPRSS15) |
| TNC | ECM Glycoproteins | 23419153 | [23419153](https://www.ncbi.nlm.nih.gov/pubmed/23419153) | [TNC](https://www.genecards.org/Search/Keyword?queryString=TNC) |
| TNF | Secreted Factors | 1476204 | [1476204](https://www.ncbi.nlm.nih.gov/pubmed/1476204) | [TNF](https://www.genecards.org/Search/Keyword?queryString=TNF) |
| TNFAIP6 | ECM Glycoproteins | 21937732 | [21937732](https://www.ncbi.nlm.nih.gov/pubmed/21937732) | [TNFAIP6](https://www.genecards.org/Search/Keyword?queryString=TNFAIP6) |
| TNFSF10 | Secreted Factors | 26555706 | [26555706](https://www.ncbi.nlm.nih.gov/pubmed/26555706) | [TNFSF10](https://www.genecards.org/Search/Keyword?queryString=TNFSF10) |
| TNFSF11 | Secreted Factors | 28877641 | [28877641](https://www.ncbi.nlm.nih.gov/pubmed/28877641) | [TNFSF11](https://www.genecards.org/Search/Keyword?queryString=TNFSF11) |
| TNFSF12 | Secreted Factors | 22380695 | [22380695](https://www.ncbi.nlm.nih.gov/pubmed/22380695) | [TNFSF12](https://www.genecards.org/Search/Keyword?queryString=TNFSF12) |
| TNFSF13 | Secreted Factors | 27822476 | [27822476](https://www.ncbi.nlm.nih.gov/pubmed/27822476) | [TNFSF13](https://www.genecards.org/Search/Keyword?queryString=TNFSF13) |
| TNFSF13B | Secreted Factors | 17414611 | [17414611](https://www.ncbi.nlm.nih.gov/pubmed/17414611) | [TNFSF13B](https://www.genecards.org/Search/Keyword?queryString=TNFSF13B) |
| TNFSF14 | Secreted Factors | 24782592 | [24782592](https://www.ncbi.nlm.nih.gov/pubmed/24782592) | [TNFSF14](https://www.genecards.org/Search/Keyword?queryString=TNFSF14) |
| TNFSF15 | Secreted Factors | 23642711 | [23642711](https://www.ncbi.nlm.nih.gov/pubmed/ 23642711) | [TNFSF15](https://www.genecards.org/Search/Keyword?queryString=TNFSF15) |
| TNFSF18 | Secreted Factors | 17359498 | [17359498](https://www.ncbi.nlm.nih.gov/pubmed/17359498) | [TNFSF18](https://www.genecards.org/Search/Keyword?queryString=TNFSF18) |
| TNFSF4 | Secreted Factors | 26376865 | [26376865](https://www.ncbi.nlm.nih.gov/pubmed/26376865) | [TNFSF4](https://www.genecards.org/Search/Keyword?queryString=TNFSF4) |
| TNFSF8 | Secreted Factors | 26039874 | [26039874](https://www.ncbi.nlm.nih.gov/pubmed/26039874) | [TNFSF8](https://www.genecards.org/Search/Keyword?queryString=TNFSF8) |
| TNFSF9 | Secreted Factors | 26039874 | [26039874](https://www.ncbi.nlm.nih.gov/pubmed/26039874) | [TNFSF9](https://www.genecards.org/Search/Keyword?queryString=TNFSF9) |
| TNIP1 | ECM associated regulatory factors | 9923610 | [9923610](https://www.ncbi.nlm.nih.gov/pubmed/9923610) | [TNIP1](https://www.genecards.org/Search/Keyword?queryString=TNIP1) |
| TNK2 | ECM associated regulatory factors | 9763437 | [9763437](https://www.ncbi.nlm.nih.gov/pubmed/9763437) | [TNK2](https://www.genecards.org/Search/Keyword?queryString=TNK2) |
| TNN | ECM Glycoproteins | 23419153 | [23419153](https://www.ncbi.nlm.nih.gov/pubmed/23419153) | [TNN](https://www.genecards.org/Search/Keyword?queryString=TNN) |
| TNR | ECM Glycoproteins | 23419153 | [23419153](https://www.ncbi.nlm.nih.gov/pubmed/23419153) | [TNR](https://www.genecards.org/Search/Keyword?queryString=TNR) |
| TNXB | ECM Glycoproteins | 23419153 | [23419153](https://www.ncbi.nlm.nih.gov/pubmed/23419153) | [TNXB](https://www.genecards.org/Search/Keyword?queryString=TNXB) |
| TPH1 | ECM synthetic/degradation enzyme | 18234316 | [18234316](https://www.ncbi.nlm.nih.gov/pubmed/18234316) | [TPH1](https://www.genecards.org/Search/Keyword?queryString=TPH1) |
| TPO | Secreted Factors | 22664934 | [22664934](https://www.ncbi.nlm.nih.gov/pubmed/22664934) | [TPO](https://www.genecards.org/Search/Keyword?queryString=TPO) |
| TPSG1 | ECM synthetic/degradation enzyme | 17498058 | [17498058](https://www.ncbi.nlm.nih.gov/pubmed/17498058) | [TPSG1](https://www.genecards.org/Search/Keyword?queryString=TPSG1) |
| TRAPPC10 | Cytoskeleton | 19147749 | [19147749](https://www.ncbi.nlm.nih.gov/pubmed/19147749) | [TRAPPC10](https://www.genecards.org/Search/Keyword?queryString=TRAPPC10) |
| TRIB3 | ECM synthetic/degradation enzyme | 25603829 | [25603829](https://www.ncbi.nlm.nih.gov/pubmed/25603829) | [TRIB3](https://www.genecards.org/Search/Keyword?queryString=TRIB3) |
| TSKU | ECM Glycoproteins | 23419153 | [23419153](https://www.ncbi.nlm.nih.gov/pubmed/23419153) | [TSKU](https://www.genecards.org/Search/Keyword?queryString=TSKU) |
| TSPEAR | ECM Glycoproteins | 22159717 | [22159717](https://www.ncbi.nlm.nih.gov/pubmed/22159717 ) | [TSPEAR](https://www.genecards.org/Search/Keyword?queryString=TSPEAR) |
| TTF2 | ECM associated regulatory factors | 8723840 | [8723840](https://www.ncbi.nlm.nih.gov/pubmed/8723840) | [TTF2](https://www.genecards.org/Search/Keyword?queryString=TTF2) |
| TWIST1 | ECM associated regulatory factors | 20804746 | [20804746](https://www.ncbi.nlm.nih.gov/pubmed/20804746) | [TWIST1](https://www.genecards.org/Search/Keyword?queryString=TWIST1) |
| TWIST2 | ECM associated regulatory factors | 23557174 | [23557174](https://www.ncbi.nlm.nih.gov/pubmed/23557174) | [TWIST2](https://www.genecards.org/Search/Keyword?queryString=TWIST2) |
| UGDH | ECM synthetic/degradation enzyme | 10607914 | [10607914](https://www.ncbi.nlm.nih.gov/pubmed/10607914) | [UGDH](https://www.genecards.org/Search/Keyword?queryString=UGDH) |
| UXS1 | ECM synthetic/degradation enzyme | 19199708 | [19199708](https://www.ncbi.nlm.nih.gov/pubmed/19199708) | [UXS1](https://www.genecards.org/Search/Keyword?queryString=UXS1) |
| VANGL2 | Cytoskeleton | 29097183 | [29097183](https://www.ncbi.nlm.nih.gov/pubmed/29097183) | [VANGL2](https://www.genecards.org/Search/Keyword?queryString=VANGL2) |
| VAV2 | ECM associated regulatory factors | 16397238 | [16397238](https://www.ncbi.nlm.nih.gov/pubmed/16397238) | [VAV2](https://www.genecards.org/Search/Keyword?queryString=VAV2) |
| VCAN | Proteoglycans | 23419153 | [23419153](https://www.ncbi.nlm.nih.gov/pubmed/23419153) | [VCAN](https://www.genecards.org/Search/Keyword?queryString=VCAN) |
| VEGFA | Secreted Factors | 27822332 | [27822332](https://www.ncbi.nlm.nih.gov/pubmed/27822332) | [VEGFA](https://www.genecards.org/Search/Keyword?queryString=VEGFA) |
| VEGFB | Secreted Factors | 26773314 | [26773314](https://www.ncbi.nlm.nih.gov/pubmed/26773314) | [VEGFB](https://www.genecards.org/Search/Keyword?queryString=VEGFB) |
| VEGFC | Secreted Factors | 28788063 | [28788063](https://www.ncbi.nlm.nih.gov/pubmed/28788063) | [VEGFC](https://www.genecards.org/Search/Keyword?queryString=VEGFC) |
| VIM | Cytoskeleton | 23979707 | [23979707](https://www.ncbi.nlm.nih.gov/pubmed/23979707) | [VIM](https://www.genecards.org/Search/Keyword?queryString=VIM) |
| VIP | ECM Micro-enviornment associated | 18174366 | [18174366](https://www.ncbi.nlm.nih.gov/pubmed/18174366) | [VIP](https://www.genecards.org/Search/Keyword?queryString=VIP) |
| VIT | ECM Glycoproteins | 21937732 | [21937732](https://www.ncbi.nlm.nih.gov/pubmed/21937732) | [VIT](https://www.genecards.org/Search/Keyword?queryString=VIT) |
| VLDLR | ECM receptor | 10571240 | [10571240](https://www.ncbi.nlm.nih.gov/pubmed/10571240) | [VLDLR](https://www.genecards.org/Search/Keyword?queryString=VLDLR) |
| VTN | ECM Glycoproteins | 21937732 | [21937732](https://www.ncbi.nlm.nih.gov/pubmed/21937732) | [VTN](https://www.genecards.org/Search/Keyword?queryString=VTN) |
| VWA1 | ECM Glycoproteins | 21937732 | [21937732](https://www.ncbi.nlm.nih.gov/pubmed/21937732) | [VWA1](https://www.genecards.org/Search/Keyword?queryString=VWA1) |
| VWA2 | ECM Glycoproteins | 21937732 | [21937732](https://www.ncbi.nlm.nih.gov/pubmed/21937732) | [VWA2](https://www.genecards.org/Search/Keyword?queryString=VWA2) |
| VWA3A | ECM Glycoproteins | 22159717 | [22159717](https://www.ncbi.nlm.nih.gov/pubmed/22159717  ) | [VWA3A](https://www.genecards.org/Search/Keyword?queryString=VWA3A) |
| VWA3B | ECM Glycoproteins | 22159717 | [22159717](https://www.ncbi.nlm.nih.gov/pubmed/22159717  ) | [VWA3B](https://www.genecards.org/Search/Keyword?queryString=VWA3B) |
| VWA5A | ECM Glycoproteins | 22159717 | [22159717](https://www.ncbi.nlm.nih.gov/pubmed/22159717  ) | [VWA5A](https://www.genecards.org/Search/Keyword?queryString=VWA5A) |
| VWA5B1 | ECM Glycoproteins | 22159717 | [22159717](https://www.ncbi.nlm.nih.gov/pubmed/22159717  ) | [VWA5B1](https://www.genecards.org/Search/Keyword?queryString=VWA5B1) |
| VWA5B2 | ECM Glycoproteins | 27988214 | [27988214](https://www.ncbi.nlm.nih.gov/pubmed/27988214) | [VWA5B2](https://www.genecards.org/Search/Keyword?queryString=VWA5B2) |
| VWA7 | ECM Glycoproteins | 22159717 | [22159717](https://www.ncbi.nlm.nih.gov/pubmed/22159717 ) | [VWA7](https://www.genecards.org/Search/Keyword?queryString=VWA7) |
| VWA9 | ECM Glycoproteins | 26163349 | [26163349](https://www.ncbi.nlm.nih.gov/pubmed/26163349) | [VWA9](https://www.genecards.org/Search/Keyword?queryString=VWA9) |
| VWC2 | Secreted Factors | 21937732 | [21937732](https://www.ncbi.nlm.nih.gov/pubmed/21937732) | [VWC2](https://www.genecards.org/Search/Keyword?queryString=VWC2) |
| VWC2L | Secreted Factors | 22209847 | [22209847](https://www.ncbi.nlm.nih.gov/pubmed/22209847) | [VWC2L](https://www.genecards.org/Search/Keyword?queryString=VWC2L) |
| VWCE | ECM Glycoproteins | 22159717 | [22159717](https://www.ncbi.nlm.nih.gov/pubmed/22159717 ) | [VWCE](https://www.genecards.org/Search/Keyword?queryString=VWCE) |
| VWDE | ECM Glycoproteins | 29196464 | [29196464](https://www.ncbi.nlm.nih.gov/pubmed/29196464) | [VWDE](https://www.genecards.org/Search/Keyword?queryString=VWDE) |
| VWF | ECM Glycoproteins | 21937732 | [21937732](https://www.ncbi.nlm.nih.gov/pubmed/21937732) | [VWF](https://www.genecards.org/Search/Keyword?queryString=VWF) |
| WFIKKN1 | Secreted Factors | 22159717 | [22159717](https://www.ncbi.nlm.nih.gov/pubmed/22159717) | [WFIKKN1](https://www.genecards.org/Search/Keyword?queryString=WFIKKN1) |
| WFIKKN2 | Secreted Factors | 22159717 | [22159717](https://www.ncbi.nlm.nih.gov/pubmed/22159717) | [WFIKKN2](https://www.genecards.org/Search/Keyword?queryString=WFIKKN2) |
| WIF1 | Secreted Factors | 26854061 | [26854061](https://www.ncbi.nlm.nih.gov/pubmed/26854061) | [WIF1](https://www.genecards.org/Search/Keyword?queryString=WIF1) |
| WISP1 | ECM Glycoproteins | 21937732 | [21937732](https://www.ncbi.nlm.nih.gov/pubmed/21937732) | [WISP1](https://www.genecards.org/Search/Keyword?queryString=WISP1) |
| WISP2 | ECM Glycoproteins | 21937732 | [21937732](https://www.ncbi.nlm.nih.gov/pubmed/21937732) | [WISP2](https://www.genecards.org/Search/Keyword?queryString=WISP2) |
| WISP3 | ECM Glycoproteins | 21937732 | [21937732](https://www.ncbi.nlm.nih.gov/pubmed/21937732) | [WISP3](https://www.genecards.org/Search/Keyword?queryString=WISP3) |
| WNT1 | Secreted Factors | 21969569 | [21969569](https://www.ncbi.nlm.nih.gov/pubmed/21969569) | [WNT1](https://www.genecards.org/Search/Keyword?queryString=WNT1) |
| WNT10A | Secreted Factors | 25054240 | [25054240](https://www.ncbi.nlm.nih.gov/pubmed/25054240) | [WNT10A](https://www.genecards.org/Search/Keyword?queryString=WNT10A) |
| WNT10B | Secreted Factors | 25995040 | [25995040](https://www.ncbi.nlm.nih.gov/pubmed/25995040) | [WNT10B](https://www.genecards.org/Search/Keyword?queryString=WNT10B) |
| WNT11 | Secreted Factors | 25813538 | [25813538](https://www.ncbi.nlm.nih.gov/pubmed/25813538) | [WNT11](https://www.genecards.org/Search/Keyword?queryString=WNT11) |
| WNT16 | Secreted Factors | 21980461 | [21980461](https://www.ncbi.nlm.nih.gov/pubmed/21980461) | [WNT16](https://www.genecards.org/Search/Keyword?queryString=WNT16) |
| WNT2 | Secreted Factors | 22028906 | [22028906](https://www.ncbi.nlm.nih.gov/pubmed/22028906) | [WNT2](https://www.genecards.org/Search/Keyword?queryString=WNT2) |
| WNT2B | Secreted Factors | 26854061 | [26854061](https://www.ncbi.nlm.nih.gov/pubmed/26854061) | [WNT2B](https://www.genecards.org/Search/Keyword?queryString=WNT2B) |
| WNT3 | Secreted Factors | 21969569 | [21969569](https://www.ncbi.nlm.nih.gov/pubmed/ 21969569) | [WNT3](https://www.genecards.org/Search/Keyword?queryString=WNT3) |
| WNT3A | Secreted Factors | 26854061 | [26854061](https://www.ncbi.nlm.nih.gov/pubmed/26854061) | [WNT3A](https://www.genecards.org/Search/Keyword?queryString=WNT3A) |
| WNT4 | Secreted Factors | 25270402 | [25270402](https://www.ncbi.nlm.nih.gov/pubmed/25270402) | [WNT4](https://www.genecards.org/Search/Keyword?queryString=WNT4) |
| WNT5A | Secreted Factors | 25813538 | [25813538](https://www.ncbi.nlm.nih.gov/pubmed/25813538) | [WNT5A](https://www.genecards.org/Search/Keyword?queryString=WNT5A) |
| WNT5B | Secreted Factors | 26459057 | [26459057](https://www.ncbi.nlm.nih.gov/pubmed/26459057) | [WNT5B](https://www.genecards.org/Search/Keyword?queryString=WNT5B) |
| WNT6 | Secreted Factors | 26854061 | [26854061](https://www.ncbi.nlm.nih.gov/pubmed/26854061) | [WNT6](https://www.genecards.org/Search/Keyword?queryString=WNT6) |
| WNT7A | Secreted Factors | 26838601 | [26838601](https://www.ncbi.nlm.nih.gov/pubmed/26838601) | [WNT7A](https://www.genecards.org/Search/Keyword?queryString=WNT7A) |
| WNT7B | Secreted Factors | 19690384 | [19690384](https://www.ncbi.nlm.nih.gov/pubmed/19690384) | [WNT7B](https://www.genecards.org/Search/Keyword?queryString=WNT7B) |
| WNT8A | Secreted Factors | 23207593 | [23207593](https://www.ncbi.nlm.nih.gov/pubmed/23207593) | [WNT8A](https://www.genecards.org/Search/Keyword?queryString=WNT8A) |
| WNT8B | Secreted Factors | 16412233 | [16412233](https://www.ncbi.nlm.nih.gov/pubmed/16412233) | [WNT8B](https://www.genecards.org/Search/Keyword?queryString=WNT8B) |
| WNT9A | Secreted Factors | 27908786 | [27908786](https://www.ncbi.nlm.nih.gov/pubmed/27908786) | [WNT9A](https://www.genecards.org/Search/Keyword?queryString=WNT9A) |
| WNT9B | Secreted Factors | 19961844 | [19961844](https://www.ncbi.nlm.nih.gov/pubmed/19961844) | [WNT9B](https://www.genecards.org/Search/Keyword?queryString=WNT9B) |
| XCL1 | Secreted Factors | 25497737 | [25497737](https://www.ncbi.nlm.nih.gov/pubmed/25497737) | [XCL1](https://www.genecards.org/Search/Keyword?queryString=XCL1) |
| XCL2 | Secreted Factors | 25497737 | [25497737](https://www.ncbi.nlm.nih.gov/pubmed/25497737) | [XCL2](https://www.genecards.org/Search/Keyword?queryString=XCL2) |
| XDH | ECM synthetic/degradation enzyme | 16502470 | [16502470](https://www.ncbi.nlm.nih.gov/pubmed/16502470) | [XDH](https://www.genecards.org/Search/Keyword?queryString=XDH) |
| XYLT1 | ECM synthetic/degradation enzyme | 11099377 | [11099377](https://www.ncbi.nlm.nih.gov/pubmed/11099377) | [XYLT1](https://www.genecards.org/Search/Keyword?queryString=XYLT1) |
| XYLT2 | ECM synthetic/degradation enzyme | 11099377 | [11099377](https://www.ncbi.nlm.nih.gov/pubmed/11099377) | [XYLT2](https://www.genecards.org/Search/Keyword?queryString=XYLT2) |
| ZFP91 | Secreted Factors | 25423310 | [25423310](https://www.ncbi.nlm.nih.gov/pubmed/25423310) | [ZFP91](https://www.genecards.org/Search/Keyword?queryString=ZFP91) |
| ZMPSTE24 | ECM synthetic/degradation enzyme | 19056867 | [19056867](https://www.ncbi.nlm.nih.gov/pubmed/19056867) | [ZMPSTE24](https://www.genecards.org/Search/Keyword?queryString=ZMPSTE24) |
| ZP1 | ECM Glycoproteins | 21937732 | [21937732](https://www.ncbi.nlm.nih.gov/pubmed/21937732) | [ZP1](https://www.genecards.org/Search/Keyword?queryString=ZP1) |
| ZP2 | ECM Glycoproteins | 21937732 | [21937732](https://www.ncbi.nlm.nih.gov/pubmed/21937732) | [ZP2](https://www.genecards.org/Search/Keyword?queryString=ZP2) |
| ZP3 | ECM Glycoproteins | 21937732 | [21937732](https://www.ncbi.nlm.nih.gov/pubmed/21937732) | [ZP3](https://www.genecards.org/Search/Keyword?queryString=ZP3) |
| ZP4 | ECM Glycoproteins | 21937732 | [21937732](https://www.ncbi.nlm.nih.gov/pubmed/21937732) | [ZP4](https://www.genecards.org/Search/Keyword?queryString=ZP4) |
| ZPLD1 | ECM Glycoproteins | 21937732 | [21937732](https://www.ncbi.nlm.nih.gov/pubmed/21937732) | [ZPLD1](https://www.genecards.org/Search/Keyword?queryString=ZPLD1) |
