# Supplementary material for: Computational analysis for identification of the extracellular matrix molecules involved in endometrial cancer progression
Source: PLoS One. 2020 Apr 21;15(4):e0231594. doi: 10.1371/journal.pone.0231594 (PMC7173926; doi:10.1371/journal.pone.0231594)
Supplement: S2 Table — (DOCX) [file pone.0231594.s003.docx]

| **S2 Table. Tumorigenesis PAN-ECM DEGs (831 genes)** | | | | | | |
| --- | --- | --- | --- | --- | --- | --- |
| **Gene Symbol** | **Ensembl_gene_id** | **log2FoldChange** | **lfcSE** | **stat** | **p value** | **padj** |
| **A2M** | ENSG00000175899 | -1.87 | 0.20804 | -8.9964 | 2.33E-19 | *3.81E-18* |
| **A2ML1** | ENSG00000166535 | 2.06 | 0.44096 | 4.66359 | 3.11E-06 | *1.18E-05* |
| **ABI3BP** | ENSG00000154175 | -3.53 | 0.33085 | -10.668 | 1.44E-26 | *4.37E-25* |
| **ACTN1** | ENSG00000072110 | -1.36 | 0.14592 | -9.3331 | 1.03E-20 | *1.87E-19* |
| **ADAM11** | ENSG00000073670 | -1.73 | 0.32784 | -5.2636 | 1.41E-07 | *6.51E-07* |
| **ADAM12** | ENSG00000148848 | 1.37 | 0.32344 | 4.24248 | 2.21E-05 | *7.39E-05* |
| **ADAM15** | ENSG00000143537 | 0.62 | 0.17129 | 3.60878 | 3.08E-04 | *8.52E-04* |
| **ADAM2** | ENSG00000104755 | 4.88 | 0.79662 | 6.12508 | 9.06E-10 | *5.44E-09* |
| **ADAM22** | ENSG00000008277 | -2.38 | 0.22871 | -10.385 | 2.91E-25 | *7.81E-24* |
| **ADAM28** | ENSG00000042980 | 1.16 | 0.30617 | 3.79777 | 1.46E-04 | *4.28E-04* |
| **ADAM29** | ENSG00000168594 | -2.10 | 0.61039 | -3.4477 | 5.65E-04 | *1.49E-03* |
| **ADAM32** | ENSG00000197140 | 1.50 | 0.29099 | 5.14553 | 2.67E-07 | *1.18E-06* |
| **ADAM33** | ENSG00000149451 | -2.54 | 0.41096 | -6.1868 | 6.14E-10 | *3.77E-09* |
| **ADAM8** | ENSG00000151651 | 1.37 | 0.22373 | 6.10462 | 1.03E-09 | *6.14E-09* |
| **ADAMDEC1** | ENSG00000134028 | 2.93 | 0.38137 | 7.68486 | 1.53E-14 | *1.57E-13* |
| **ADAMTS1** | ENSG00000154734 | -2.36 | 0.25431 | -9.2712 | 1.84E-20 | *3.30E-19* |
| **ADAMTS10** | ENSG00000142303 | -0.92 | 0.29163 | -3.1646 | 1.55E-03 | *3.77E-03* |
| **ADAMTS14** | ENSG00000138316 | 2.43 | 0.32687 | 7.44119 | 9.98E-14 | *9.42E-13* |
| **ADAMTS15** | ENSG00000166106 | 1.66 | 0.36136 | 4.60407 | 4.14E-06 | *1.54E-05* |
| **ADAMTS18** | ENSG00000140873 | 1.37 | 0.47817 | 2.87393 | 4.05E-03 | *9.08E-03* |
| **ADAMTS3** | ENSG00000156140 | -2.22 | 0.27324 | -8.1191 | 4.70E-16 | *5.53E-15* |
| **ADAMTS4** | ENSG00000158859 | -2.43 | 0.24907 | -9.7762 | 1.43E-22 | *3.11E-21* |
| **ADAMTS5** | ENSG00000154736 | -3.04 | 0.31475 | -9.646 | 5.11E-22 | *1.04E-20* |
| **ADAMTS6** | ENSG00000049192 | 1.41 | 0.40147 | 3.51157 | 4.45E-04 | *1.20E-03* |
| **ADAMTS7** | ENSG00000136378 | 1.53 | 0.28581 | 5.34994 | 8.80E-08 | *4.16E-07* |
| **ADAMTSL1** | ENSG00000178031 | -2.45 | 0.35755 | -6.8569 | 7.03E-12 | *5.42E-11* |
| **ADAMTSL2** | ENSG00000197859 | 1.41 | 0.39775 | 3.53513 | 4.08E-04 | *1.10E-03* |
| **ADAMTSL3** | ENSG00000156218 | -1.97 | 0.43549 | -4.5126 | 6.40E-06 | *2.32E-05* |
| **ADAMTSL4** | ENSG00000143382 | -2.36 | 0.26039 | -9.0763 | 1.12E-19 | *1.89E-18* |
| **ADAMTSL5** | ENSG00000185761 | -3.04 | 0.36314 | -8.3706 | 5.73E-17 | *7.38E-16* |
| **ADCY10** | ENSG00000143199 | 0.91 | 0.30816 | 2.96586 | 3.02E-03 | *6.93E-03* |
| **ADGRL3** | ENSG00000150471 | -1.25 | 0.42801 | -2.918 | 3.52E-03 | *7.99E-03* |
| **ADIPOQ** | ENSG00000181092 | -2.48 | 0.81045 | -3.063 | 2.19E-03 | *5.18E-03* |
| **AEBP1** | ENSG00000106624 | -2.29 | 0.27045 | -8.4511 | 2.89E-17 | *3.82E-16* |
| **AGRN** | ENSG00000188157 | 1.14 | 0.19342 | 5.88523 | 3.97E-09 | *2.21E-08* |
| **AGTR1** | ENSG00000144891 | -4.45 | 0.5217 | -8.5236 | 1.55E-17 | *2.12E-16* |
| **AKT1** | ENSG00000142208 | 0.60 | 0.10735 | 5.59921 | 2.15E-08 | *1.10E-07* |
| **AKT3** | ENSG00000117020 | -3.50 | 0.23378 | -14.988 | 8.84E-51 | *1.26E-48* |
| **AMBN** | ENSG00000178522 | 7.21 | 1.11587 | 6.45946 | 1.05E-10 | *7.07E-10* |
| **AMBP** | ENSG00000106927 | 4.36 | 0.49917 | 8.73497 | 2.44E-18 | *3.58E-17* |
| **AMH** | ENSG00000104899 | 4.56 | 0.40448 | 11.2831 | 1.59E-29 | *6.03E-28* |
| **ANGPT1** | ENSG00000154188 | -2.40 | 0.34461 | -6.9782 | 2.99E-12 | *2.39E-11* |
| **ANGPT4** | ENSG00000101280 | -2.62 | 0.51742 | -5.0634 | 4.12E-07 | *1.78E-06* |
| **ANGPTL1** | ENSG00000116194 | -4.00 | 0.48386 | -8.2754 | 1.28E-16 | *1.60E-15* |
| **ANGPTL2** | ENSG00000136859 | -1.76 | 0.21766 | -8.0883 | 6.05E-16 | *7.07E-15* |
| **ANGPTL4** | ENSG00000167772 | 1.91 | 0.34589 | 5.53067 | 3.19E-08 | *1.60E-07* |
| **ANGPTL5** | ENSG00000187151 | -5.23 | 0.54757 | -9.5558 | 1.23E-21 | *2.43E-20* |
| **ANGPTL6** | ENSG00000130812 | 0.87 | 0.22405 | 3.88953 | 1.00E-04 | *3.03E-04* |
| **ANGPTL7** | ENSG00000171819 | -2.52 | 0.5628 | -4.4749 | 7.65E-06 | *2.74E-05* |
| **ANK1** | ENSG00000029534 | 2.06 | 0.39228 | 5.2546 | 1.48E-07 | *6.81E-07* |
| **ANK2** | ENSG00000145362 | -2.10 | 0.32681 | -6.4135 | 1.42E-10 | *9.41E-10* |
| **ANXA13** | ENSG00000104537 | -3.79 | 0.53621 | -7.0636 | 1.62E-12 | *1.34E-11* |
| **ANXA2** | ENSG00000182718 | 1.20 | 0.18072 | 6.63183 | 3.32E-11 | *2.36E-10* |
| **ANXA3** | ENSG00000138772 | 0.74 | 0.2114 | 3.50794 | 4.52E-04 | *1.21E-03* |
| **ANXA6** | ENSG00000197043 | -1.75 | 0.20599 | -8.4717 | 2.42E-17 | *3.23E-16* |
| **ANXA8** | ENSG00000265190 | 2.06 | 0.57697 | 3.57172 | 3.55E-04 | *9.72E-04* |
| **ANXA9** | ENSG00000143412 | -0.93 | 0.24882 | -3.7558 | 1.73E-04 | *5.00E-04* |
| **APOA5** | ENSG00000110243 | 2.60 | 0.70066 | 3.71722 | 2.01E-04 | *5.77E-04* |
| **APOC1** | ENSG00000130208 | 2.26 | 0.35962 | 6.28834 | 3.21E-10 | *2.03E-09* |
| **APOL4** | ENSG00000100336 | 1.95 | 0.26099 | 7.45383 | 9.07E-14 | *8.61E-13* |
| **APOLD1** | ENSG00000178878 | -3.49 | 0.24459 | -14.282 | 2.85E-46 | *3.27E-44* |
| **ARG1** | ENSG00000118520 | -1.19 | 0.28533 | -4.1552 | 3.25E-05 | *1.06E-04* |
| **ARG2** | ENSG00000081181 | 0.88 | 0.25286 | 3.47389 | 5.13E-04 | *1.36E-03* |
| **ARSG** | ENSG00000141337 | -0.61 | 0.17713 | -3.4628 | 5.35E-04 | *1.42E-03* |
| **ARSH** | ENSG00000205667 | 3.93 | 0.39503 | 9.93773 | 2.85E-23 | *6.62E-22* |
| **ARTN** | ENSG00000117407 | 2.35 | 0.22649 | 10.3652 | 3.57E-25 | *9.50E-24* |
| **ASPN** | ENSG00000106819 | -4.03 | 0.393 | -10.244 | 1.26E-24 | *3.22E-23* |
| **ASTL** | ENSG00000188886 | 1.54 | 0.38633 | 3.97352 | 7.08E-05 | *2.19E-04* |
| **ATP1A3** | ENSG00000105409 | 1.30 | 0.36141 | 3.60873 | 3.08E-04 | *8.52E-04* |
| **B3GAT3** | ENSG00000149541 | 1.15 | 0.15701 | 7.29848 | 2.91E-13 | *2.61E-12* |
| **B4GALT7** | ENSG00000027847 | 1.08 | 0.15186 | 7.12903 | 1.01E-12 | *8.52E-12* |
| **BCAN** | ENSG00000132692 | -2.27 | 0.38145 | -5.9534 | 2.63E-09 | *1.49E-08* |
| **BDNF** | ENSG00000176697 | 2.92 | 0.39012 | 7.48302 | 7.26E-14 | *6.94E-13* |
| **BGLAP** | ENSG00000242252 | 0.73 | 0.2537 | 2.86609 | 4.16E-03 | *9.28E-03* |
| **BMP5** | ENSG00000112175 | 3.87 | 0.75659 | 5.11901 | 3.07E-07 | *1.35E-06* |
| **BMP8A** | ENSG00000183682 | -1.06 | 0.27297 | -3.8763 | 1.06E-04 | *3.19E-04* |
| **BMP8B** | ENSG00000116985 | 0.68 | 0.23648 | 2.86153 | 4.22E-03 | *9.40E-03* |
| **BMPER** | ENSG00000164619 | -2.53 | 0.48159 | -5.2587 | 1.45E-07 | *6.67E-07* |
| **BMPR1B** | ENSG00000138696 | 1.04 | 0.32287 | 3.2335 | 1.22E-03 | *3.03E-03* |
| **BMPR2** | ENSG00000204217 | -1.06 | 0.14072 | -7.5151 | 5.69E-14 | *5.49E-13* |
| **BRINP2** | ENSG00000198797 | 1.68 | 0.55027 | 3.04754 | 2.31E-03 | *5.43E-03* |
| **BRINP3** | ENSG00000162670 | -2.88 | 0.63721 | -4.5137 | 6.37E-06 | *2.31E-05* |
| **C1QB** | ENSG00000173369 | 1.13 | 0.3299 | 3.438 | 5.86E-04 | *1.54E-03* |
| **C1QC** | ENSG00000159189 | 1.03 | 0.29696 | 3.45345 | 5.53E-04 | *1.46E-03* |
| **C1QL1** | ENSG00000131094 | 1.62 | 0.49456 | 3.27214 | 1.07E-03 | *2.68E-03* |
| **C1QL4** | ENSG00000186897 | 4.78 | 0.47353 | 10.1007 | 5.48E-24 | *1.34E-22* |
| **C1QTNF1** | ENSG00000173918 | -2.54 | 0.24103 | -10.519 | 7.03E-26 | *2.00E-24* |
| **C1QTNF2** | ENSG00000145861 | -3.31 | 0.26336 | -12.575 | 2.89E-36 | *1.69E-34* |
| **C1QTNF4** | ENSG00000172247 | -1.62 | 0.33507 | -4.841 | 1.29E-06 | *5.19E-06* |
| **C1QTNF6** | ENSG00000133466 | 1.69 | 0.23203 | 7.26593 | 3.70E-13 | *3.27E-12* |
| **C1QTNF7** | ENSG00000163145 | -3.50 | 0.32091 | -10.918 | 9.41E-28 | *3.11E-26* |
| **C1QTNF9** | ENSG00000240654 | -1.87 | 0.43485 | -4.3106 | 1.63E-05 | *5.55E-05* |
| **C4BPA** | ENSG00000123838 | 2.64 | 0.55794 | 4.72833 | 2.26E-06 | *8.77E-06* |
| **CABP1** | ENSG00000157782 | -1.43 | 0.24769 | -5.7838 | 7.30E-09 | *3.94E-08* |
| **CADM1** | ENSG00000182985 | 1.26 | 0.2362 | 5.354 | 8.60E-08 | *4.07E-07* |
| **CADM3** | ENSG00000162706 | -3.20 | 0.50924 | -6.2935 | 3.10E-10 | *1.97E-09* |
| **CADM4** | ENSG00000105767 | 1.53 | 0.16602 | 9.24047 | 2.45E-20 | *4.37E-19* |
| **CALCRL** | ENSG00000064989 | -2.28 | 0.29564 | -7.7217 | 1.15E-14 | *1.19E-13* |
| **CAMK2A** | ENSG00000070808 | -5.00 | 0.3493 | -14.319 | 1.66E-46 | *1.95E-44* |
| **CAMK2D** | ENSG00000145349 | -0.74 | 0.18117 | -4.11 | 3.96E-05 | *1.27E-04* |
| **CAMK2G** | ENSG00000148660 | -1.56 | 0.12026 | -13.013 | 1.04E-38 | *7.33E-37* |
| **CAMK2N2** | ENSG00000163888 | 1.76 | 0.35536 | 4.96609 | 6.83E-07 | *2.85E-06* |
| **CAMP** | ENSG00000164047 | 4.59 | 0.58699 | 7.82737 | 4.98E-15 | *5.35E-14* |
| **CANT1** | ENSG00000171302 | 0.78 | 0.13521 | 5.75764 | 8.53E-09 | *4.56E-08* |
| **CASP3** | ENSG00000164305 | 1.00 | 0.10009 | 9.95124 | 2.49E-23 | *5.81E-22* |
| **CAV1** | ENSG00000105974 | -3.81 | 0.21726 | -17.537 | 7.53E-69 | *2.37E-66* |
| **CAV2** | ENSG00000105971 | -2.49 | 0.22121 | -11.275 | 1.74E-29 | *6.56E-28* |
| **CAV3** | ENSG00000182533 | -1.85 | 0.56427 | -3.2767 | 1.05E-03 | *2.64E-03* |
| **CBL** | ENSG00000110395 | -0.68 | 0.14605 | -4.6456 | 3.39E-06 | *1.28E-05* |
| **CBLC** | ENSG00000142273 | 2.64 | 0.24811 | 10.644 | 1.86E-26 | *5.58E-25* |
| **CBLN1** | ENSG00000102924 | -2.17 | 0.40392 | -5.3724 | 7.77E-08 | *3.70E-07* |
| **CBLN2** | ENSG00000141668 | 2.31 | 0.63453 | 3.63307 | 2.80E-04 | *7.81E-04* |
| **CBLN3** | ENSG00000139899 | -1.17 | 0.17757 | -6.5929 | 4.31E-11 | *3.04E-10* |
| **CBLN4** | ENSG00000054803 | -3.43 | 0.4231 | -8.1169 | 4.78E-16 | *5.63E-15* |
| **CCBE1** | ENSG00000183287 | -3.54 | 0.46145 | -7.6713 | 1.70E-14 | *1.73E-13* |
| **CCL11** | ENSG00000172156 | -1.74 | 0.54936 | -3.1668 | 1.54E-03 | *3.75E-03* |
| **CCL14** | ENSG00000276409 | -5.89 | 0.50158 | -11.741 | 7.90E-32 | *3.51E-30* |
| **CCL16** | ENSG00000275152 | -3.56 | 0.43892 | -8.1115 | 5.00E-16 | *5.87E-15* |
| **CCL17** | ENSG00000102970 | 1.93 | 0.39249 | 4.92902 | 8.26E-07 | *3.41E-06* |
| **CCL18** | ENSG00000275385 | 4.12 | 0.45158 | 9.12601 | 7.11E-20 | *1.21E-18* |
| **CCL19** | ENSG00000172724 | -1.57 | 0.46965 | -3.3489 | 8.11E-04 | *2.08E-03* |
| **CCL2** | ENSG00000108691 | -1.77 | 0.29429 | -6.0259 | 1.68E-09 | *9.80E-09* |
| **CCL20** | ENSG00000115009 | 4.22 | 0.44828 | 9.41832 | 4.58E-21 | *8.66E-20* |
| **CCL21** | ENSG00000137077 | -3.34 | 0.52172 | -6.4039 | 1.51E-10 | *9.97E-10* |
| **CCL22** | ENSG00000102962 | 1.78 | 0.34933 | 5.10361 | 3.33E-07 | *1.45E-06* |
| **CCL23** | ENSG00000274736 | -4.09 | 0.4027 | -10.162 | 2.92E-24 | *7.27E-23* |
| **CCL24** | ENSG00000106178 | 2.69 | 0.48296 | 5.57708 | 2.45E-08 | *1.24E-07* |
| **CCL25** | ENSG00000131142 | 1.76 | 0.43842 | 4.01435 | 5.96E-05 | *1.86E-04* |
| **CCL26** | ENSG00000006606 | -3.08 | 0.42264 | -7.2912 | 3.07E-13 | *2.75E-12* |
| **CCL28** | ENSG00000151882 | 1.54 | 0.29324 | 5.24505 | 1.56E-07 | *7.15E-07* |
| **CCL3** | ENSG00000277632 | 1.05 | 0.32376 | 3.24889 | 1.16E-03 | *2.88E-03* |
| **CCL7** | ENSG00000108688 | 1.53 | 0.50717 | 3.01104 | 2.60E-03 | *6.06E-03* |
| **CD109** | ENSG00000156535 | -1.26 | 0.34973 | -3.6081 | 3.09E-04 | *8.54E-04* |
| **CD36** | ENSG00000135218 | -1.63 | 0.41559 | -3.9193 | 8.88E-05 | *2.70E-04* |
| **CD44** | ENSG00000026508 | -1.25 | 0.23771 | -5.2585 | 1.45E-07 | *6.68E-07* |
| **CD86** | ENSG00000114013 | 0.82 | 0.2672 | 3.06872 | 2.15E-03 | *5.09E-03* |
| **CD93** | ENSG00000125810 | -2.05 | 0.2102 | -9.7503 | 1.84E-22 | *3.96E-21* |
| **CDH1** | ENSG00000039068 | 1.50 | 0.2054 | 7.28032 | 3.33E-13 | *2.96E-12* |
| **CDH11** | ENSG00000140937 | -1.55 | 0.26377 | -5.8856 | 3.97E-09 | *2.21E-08* |
| **CDH23** | ENSG00000107736 | -2.20 | 0.35078 | -6.2618 | 3.81E-10 | *2.40E-09* |
| **CDH24** | ENSG00000139880 | 0.92 | 0.22419 | 4.11325 | 3.90E-05 | *1.25E-04* |
| **CDH3** | ENSG00000062038 | 0.90 | 0.29502 | 3.04607 | 2.32E-03 | *5.45E-03* |
| **CDH4** | ENSG00000179242 | -2.19 | 0.41403 | -5.2958 | 1.18E-07 | *5.51E-07* |
| **CDH5** | ENSG00000179776 | -1.29 | 0.21237 | -6.0677 | 1.30E-09 | *7.65E-09* |
| **CDH8** | ENSG00000150394 | -1.68 | 0.40218 | -4.1851 | 2.85E-05 | *9.36E-05* |
| **CDHR5** | ENSG00000099834 | 1.45 | 0.45622 | 3.18437 | 1.45E-03 | *3.54E-03* |
| **CELA3A** | ENSG00000142789 | 3.42 | 0.6464 | 5.28416 | 1.26E-07 | *5.85E-07* |
| **CELA3B** | ENSG00000219073 | 2.05 | 0.5012 | 4.08342 | 4.44E-05 | *1.41E-04* |
| **CGA** | ENSG00000135346 | 2.91 | 0.74107 | 3.92745 | 8.59E-05 | *2.62E-04* |
| **CHADL** | ENSG00000100399 | 0.94 | 0.248 | 3.79839 | 1.46E-04 | *4.27E-04* |
| **CHAT** | ENSG00000070748 | 3.05 | 0.87836 | 3.47555 | 5.10E-04 | *1.36E-03* |
| **CHGA** | ENSG00000100604 | 4.21 | 0.63583 | 6.61736 | 3.66E-11 | *2.60E-10* |
| **CHPF** | ENSG00000123989 | 1.04 | 0.20462 | 5.06009 | 4.19E-07 | *1.81E-06* |
| **CHPF2** | ENSG00000033100 | 1.11 | 0.11271 | 9.81624 | 9.59E-23 | *2.13E-21* |
| **CHRD** | ENSG00000090539 | -1.70 | 0.3095 | -5.4979 | 3.84E-08 | *1.90E-07* |
| **CHRDL1** | ENSG00000101938 | -5.64 | 0.52291 | -10.785 | 4.06E-27 | *1.28E-25* |
| **CHRDL2** | ENSG00000054938 | -5.39 | 0.5302 | -10.159 | 3.03E-24 | *7.53E-23* |
| **CHST3** | ENSG00000122863 | -0.74 | 0.21908 | -3.3852 | 7.11E-04 | *1.84E-03* |
| **CHST6** | ENSG00000183196 | 2.62 | 0.34642 | 7.55585 | 4.16E-14 | *4.07E-13* |
| **CHSY3** | ENSG00000198108 | -1.18 | 0.25423 | -4.6323 | 3.62E-06 | *1.36E-05* |
| **CIB1** | ENSG00000185043 | 1.20 | 0.1859 | 6.46491 | 1.01E-10 | *6.83E-10* |
| **CILP** | ENSG00000138615 | -3.65 | 0.40961 | -8.9099 | 5.11E-19 | *8.05E-18* |
| **CILP2** | ENSG00000160161 | 3.16 | 0.39788 | 7.9367 | 2.08E-15 | *2.32E-14* |
| **CKM** | ENSG00000104879 | 2.54 | 0.36256 | 6.99356 | 2.68E-12 | *2.16E-11* |
| **CLEC14A** | ENSG00000176435 | -2.11 | 0.21489 | -9.8015 | 1.11E-22 | *2.45E-21* |
| **CLEC18A** | ENSG00000157322 | 1.19 | 0.38594 | 3.08697 | 2.02E-03 | *4.81E-03* |
| **CLEC18B** | ENSG00000140839 | 2.86 | 0.41707 | 6.86863 | 6.48E-12 | *5.02E-11* |
| **CLEC18C** | ENSG00000157335 | 2.03 | 0.55549 | 3.6554 | 2.57E-04 | *7.21E-04* |
| **CLEC1A** | ENSG00000150048 | -2.48 | 0.21405 | -11.573 | 5.62E-31 | *2.35E-29* |
| **CLEC2B** | ENSG00000110852 | -1.86 | 0.24433 | -7.5989 | 2.99E-14 | *2.97E-13* |
| **CLEC3A** | ENSG00000166509 | -2.53 | 0.86743 | -2.9164 | 3.54E-03 | *8.02E-03* |
| **CLEC3B** | ENSG00000163815 | -3.98 | 0.34261 | -11.608 | 3.73E-31 | *1.58E-29* |
| **CLEC4G** | ENSG00000182566 | -2.81 | 0.37745 | -7.4448 | 9.71E-14 | *9.19E-13* |
| **CLEC4M** | ENSG00000104938 | -4.73 | 0.55493 | -8.5153 | 1.66E-17 | *2.27E-16* |
| **CLEC5A** | ENSG00000258227 | 1.49 | 0.28669 | 5.19662 | 2.03E-07 | *9.14E-07* |
| **CLEC9A** | ENSG00000197992 | -2.22 | 0.35938 | -6.1695 | 6.85E-10 | *4.18E-09* |
| **CLIC4** | ENSG00000169504 | -2.16 | 0.18477 | -11.714 | 1.08E-31 | *4.79E-30* |
| **CLPP** | ENSG00000125656 | 0.79 | 0.15029 | 5.25624 | 1.47E-07 | *6.76E-07* |
| **CMA1** | ENSG00000092009 | -4.77 | 0.53321 | -8.9429 | 3.79E-19 | *6.05E-18* |
| **CNTF** | ENSG00000242689 | -1.05 | 0.29132 | -3.6125 | 3.03E-04 | *8.40E-04* |
| **CNTN1** | ENSG00000018236 | -1.36 | 0.39297 | -3.4518 | 5.57E-04 | *1.47E-03* |
| **CNTNAP1** | ENSG00000108797 | -1.99 | 0.21886 | -9.1004 | 9.00E-20 | *1.52E-18* |
| **COCH** | ENSG00000100473 | 2.43 | 0.35721 | 6.79687 | 1.07E-11 | *8.07E-11* |
| **COL10A1** | ENSG00000123500 | 1.42 | 0.46889 | 3.02824 | 2.46E-03 | *5.76E-03* |
| **COL11A1** | ENSG00000060718 | 2.44 | 0.46913 | 5.20739 | 1.92E-07 | *8.65E-07* |
| **COL11A2** | ENSG00000204248 | 1.46 | 0.32608 | 4.46233 | 8.11E-06 | *2.89E-05* |
| **COL12A1** | ENSG00000111799 | -0.97 | 0.30225 | -3.2115 | 1.32E-03 | *3.25E-03* |
| **COL14A1** | ENSG00000187955 | -2.86 | 0.32978 | -8.6708 | 4.29E-18 | *6.18E-17* |
| **COL15A1** | ENSG00000204291 | -2.06 | 0.24237 | -8.5183 | 1.62E-17 | *2.21E-16* |
| **COL16A1** | ENSG00000084636 | -2.32 | 0.24109 | -9.629 | 6.03E-22 | *1.23E-20* |
| **COL17A1** | ENSG00000065618 | 2.82 | 0.41752 | 6.75612 | 1.42E-11 | *1.06E-10* |
| **COL19A1** | ENSG00000082293 | -1.18 | 0.46905 | -2.5098 | 1.21E-02 | *9.41E-03* |
| **COL1A2** | ENSG00000164692 | -0.82 | 0.2784 | -2.9319 | 3.37E-03 | *7.67E-03* |
| **COL20A1** | ENSG00000101203 | -2.10 | 0.46969 | -4.479 | 7.50E-06 | *2.69E-05* |
| **COL25A1** | ENSG00000188517 | 1.54 | 0.50538 | 3.04195 | 2.35E-03 | *5.52E-03* |
| **COL26A1** | ENSG00000160963 | 4.72 | 0.45049 | 10.4779 | 1.09E-25 | *3.06E-24* |
| **COL2A1** | ENSG00000139219 | 5.52 | 0.59928 | 9.21624 | 3.08E-20 | *5.43E-19* |
| **COL3A1** | ENSG00000168542 | -1.10 | 0.28885 | -3.8207 | 1.33E-04 | *3.94E-04* |
| **COL4A4** | ENSG00000081052 | -1.88 | 0.38601 | -4.8656 | 1.14E-06 | *4.62E-06* |
| **COL4A5** | ENSG00000188153 | -0.99 | 0.25255 | -3.9352 | 8.31E-05 | *2.54E-04* |
| **COL4A6** | ENSG00000197565 | -4.46 | 0.35253 | -12.647 | 1.16E-36 | *6.90E-35* |
| **COL5A1** | ENSG00000130635 | -1.20 | 0.26085 | -4.6195 | 3.85E-06 | *1.44E-05* |
| **COL5A3** | ENSG00000080573 | -0.99 | 0.21645 | -4.5814 | 4.62E-06 | *1.71E-05* |
| **COL6A1** | ENSG00000142156 | -0.98 | 0.28088 | -3.478 | 5.05E-04 | *1.34E-03* |
| **COL6A2** | ENSG00000142173 | -1.45 | 0.28886 | -5.0158 | 5.28E-07 | *2.24E-06* |
| **COL6A3** | ENSG00000163359 | -2.20 | 0.26425 | -8.3213 | 8.70E-17 | *1.10E-15* |
| **COL6A6** | ENSG00000206384 | -2.89 | 0.43409 | -6.655 | 2.83E-11 | *2.04E-10* |
| **COL7A1** | ENSG00000114270 | 1.16 | 0.25084 | 4.64068 | 3.47E-06 | *1.31E-05* |
| **COL8A2** | ENSG00000171812 | -1.13 | 0.33697 | -3.3443 | 8.25E-04 | *2.11E-03* |
| **COL9A1** | ENSG00000112280 | 3.51 | 0.54459 | 6.44751 | 1.14E-10 | *7.61E-10* |
| **COL9A2** | ENSG00000049089 | 2.30 | 0.30371 | 7.57002 | 3.73E-14 | *3.67E-13* |
| **COL9A3** | ENSG00000092758 | 1.59 | 0.44156 | 3.61078 | 3.05E-04 | *8.45E-04* |
| **COLEC10** | ENSG00000184374 | 1.59 | 0.42931 | 3.7014 | 2.14E-04 | *6.11E-04* |
| **COLEC12** | ENSG00000158270 | -2.48 | 0.35445 | -6.9928 | 2.69E-12 | *2.17E-11* |
| **COLGALT1** | ENSG00000130309 | 0.86 | 0.12968 | 6.66121 | 2.72E-11 | *1.96E-10* |
| **COLQ** | ENSG00000206561 | -0.95 | 0.21946 | -4.3506 | 1.36E-05 | *4.68E-05* |
| **COMP** | ENSG00000105664 | 1.85 | 0.51448 | 3.59971 | 3.19E-04 | *8.79E-04* |
| **CRELD2** | ENSG00000184164 | 1.40 | 0.16389 | 8.55085 | 1.22E-17 | *1.69E-16* |
| **CRHBP** | ENSG00000145708 | -4.00 | 0.3901 | -10.243 | 1.27E-24 | *3.24E-23* |
| **CRIM1** | ENSG00000150938 | -1.99 | 0.18706 | -10.626 | 2.25E-26 | *6.66E-25* |
| **CRISPLD2** | ENSG00000103196 | -3.06 | 0.23001 | -13.296 | 2.45E-40 | *1.95E-38* |
| **CRLF1** | ENSG00000006016 | 1.32 | 0.42237 | 3.13287 | 1.73E-03 | *4.17E-03* |
| **CSGALNACT1** | ENSG00000147408 | -2.34 | 0.23928 | -9.7876 | 1.27E-22 | *2.79E-21* |
| **CSGALNACT2** | ENSG00000169826 | -0.81 | 0.16041 | -5.0395 | 4.67E-07 | *2.00E-06* |
| **CSPG4** | ENSG00000173546 | -1.89 | 0.25064 | -7.5341 | 4.92E-14 | *4.78E-13* |
| **CSPG5** | ENSG00000114646 | 3.25 | 0.33761 | 9.62712 | 6.14E-22 | *1.25E-20* |
| **CST1** | ENSG00000170373 | 6.09 | 0.67146 | 9.07397 | 1.15E-19 | *1.93E-18* |
| **CST2** | ENSG00000170369 | 4.18 | 0.57746 | 7.23987 | 4.49E-13 | *3.92E-12* |
| **CST3** | ENSG00000101439 | 0.71 | 0.22215 | 3.19168 | 1.41E-03 | *3.46E-03* |
| **CST4** | ENSG00000101441 | 6.22 | 0.73478 | 8.46632 | 2.53E-17 | *3.37E-16* |
| **CSTA** | ENSG00000121552 | 1.00 | 0.31719 | 3.14606 | 1.65E-03 | *4.00E-03* |
| **CSTB** | ENSG00000160213 | 1.83 | 0.19042 | 9.602 | 7.84E-22 | *1.58E-20* |
| **CSTL1** | ENSG00000125823 | 2.39 | 0.6198 | 3.86148 | 1.13E-04 | *3.37E-04* |
| **CTF1** | ENSG00000150281 | -1.21 | 0.20843 | -5.8025 | 6.53E-09 | *3.55E-08* |
| **CTGF** | ENSG00000118523 | -2.55 | 0.26013 | -9.7963 | 1.17E-22 | *2.57E-21* |
| **CTHRC1** | ENSG00000164932 | 2.18 | 0.33532 | 6.48743 | 8.73E-11 | *5.94E-10* |
| **CTSA** | ENSG00000064601 | 1.01 | 0.13866 | 7.31243 | 2.62E-13 | *2.37E-12* |
| **CTSB** | ENSG00000164733 | 1.30 | 0.18855 | 6.89777 | 5.28E-12 | *4.13E-11* |
| **CTSC** | ENSG00000109861 | 1.14 | 0.17061 | 6.65469 | 2.84E-11 | *2.04E-10* |
| **CTSD** | ENSG00000117984 | 0.71 | 0.1786 | 3.97115 | 7.15E-05 | *2.21E-04* |
| **CTSE** | ENSG00000196188 | 3.00 | 0.52753 | 5.67754 | 1.37E-08 | *7.13E-08* |
| **CTSF** | ENSG00000174080 | -1.65 | 0.25558 | -6.4549 | 1.08E-10 | *7.28E-10* |
| **CTSG** | ENSG00000100448 | -4.06 | 0.53005 | -7.6534 | 1.96E-14 | *1.98E-13* |
| **CTSH** | ENSG00000103811 | 0.87 | 0.23708 | 3.67266 | 2.40E-04 | *6.78E-04* |
| **CTSK** | ENSG00000143387 | -2.37 | 0.25382 | -9.3254 | 1.11E-20 | *2.01E-19* |
| **CTSL** | ENSG00000135047 | -0.78 | 0.1746 | -4.4874 | 7.21E-06 | *2.60E-05* |
| **CTSO** | ENSG00000256043 | -1.55 | 0.17068 | -9.053 | 1.39E-19 | *2.31E-18* |
| **CTSS** | ENSG00000163131 | 0.75 | 0.24911 | 3.02876 | 2.46E-03 | *5.75E-03* |
| **CTSV** | ENSG00000136943 | 5.15 | 0.38272 | 13.468 | 2.41E-41 | *2.04E-39* |
| **CTSZ** | ENSG00000101160 | 1.15 | 0.17893 | 6.40794 | 1.48E-10 | *9.74E-10* |
| **CXCL1** | ENSG00000163739 | 2.38 | 0.49596 | 4.79636 | 1.62E-06 | *6.40E-06* |
| **CXCL10** | ENSG00000169245 | 1.81 | 0.37356 | 4.83806 | 1.31E-06 | *5.26E-06* |
| **CXCL11** | ENSG00000169248 | 1.40 | 0.4269 | 3.28271 | 1.03E-03 | *2.59E-03* |
| **CXCL12** | ENSG00000107562 | -4.07 | 0.31952 | -12.727 | 4.18E-37 | *2.57E-35* |
| **CXCL13** | ENSG00000156234 | 2.81 | 0.52658 | 5.34345 | 9.12E-08 | *4.30E-07* |
| **CXCL14** | ENSG00000145824 | 3.02 | 0.49206 | 6.13713 | 8.40E-10 | *5.06E-09* |
| **CXCL2** | ENSG00000081041 | -1.35 | 0.41992 | -3.2089 | 1.33E-03 | *3.27E-03* |
| **CXCL3** | ENSG00000163734 | 1.81 | 0.40144 | 4.49948 | 6.81E-06 | *2.46E-05* |
| **CXCL5** | ENSG00000163735 | 3.66 | 0.53648 | 6.81904 | 9.16E-12 | *6.97E-11* |
| **CXCL8** | ENSG00000169429 | 2.25 | 0.38141 | 5.89115 | 3.84E-09 | *2.14E-08* |
| **CXCL9** | ENSG00000138755 | 2.23 | 0.44906 | 4.96083 | 7.02E-07 | *2.92E-06* |
| **CXCR4** | ENSG00000121966 | 0.70 | 0.23362 | 3.00383 | 2.67E-03 | *6.20E-03* |
| **CYR61** | ENSG00000142871 | -3.25 | 0.23994 | -13.528 | 1.06E-41 | *9.24E-40* |
| **DCHS1** | ENSG00000166341 | -2.09 | 0.26095 | -8.0052 | 1.19E-15 | *1.36E-14* |
| **DCN** | ENSG00000011465 | -4.44 | 0.32309 | -13.741 | 5.74E-43 | *5.42E-41* |
| **DCSTAMP** | ENSG00000164935 | 1.28 | 0.35534 | 3.60207 | 3.16E-04 | *8.72E-04* |
| **DDR2** | ENSG00000162733 | -3.44 | 0.25863 | -13.293 | 2.54E-40 | *2.00E-38* |
| **DEFB1** | ENSG00000164825 | 2.79 | 0.48535 | 5.74501 | 9.19E-09 | *4.90E-08* |
| **DEFB4A** | ENSG00000171711 | 4.47 | 0.67199 | 6.64898 | 2.95E-11 | *2.12E-10* |
| **DHH** | ENSG00000139549 | -1.38 | 0.32597 | -4.2372 | 2.26E-05 | *7.56E-05* |
| **DMBT1** | ENSG00000187908 | 3.05 | 0.55728 | 5.46686 | 4.58E-08 | *2.25E-07* |
| **DNM3** | ENSG00000197959 | -1.10 | 0.23677 | -4.6443 | 3.41E-06 | *1.29E-05* |
| **DPT** | ENSG00000143196 | -6.28 | 0.45753 | -13.736 | 6.21E-43 | *5.83E-41* |
| **DSE** | ENSG00000111817 | -1.18 | 0.19742 | -5.9607 | 2.51E-09 | *1.43E-08* |
| **DST** | ENSG00000151914 | -1.53 | 0.30228 | -5.0641 | 4.10E-07 | *1.77E-06* |
| **ECM2** | ENSG00000106823 | -3.77 | 0.29963 | -12.576 | 2.87E-36 | *1.68E-34* |
| **EDIL3** | ENSG00000164176 | -2.87 | 0.29381 | -9.7765 | 1.42E-22 | *3.10E-21* |
| **EDN1** | ENSG00000078401 | -1.03 | 0.23454 | -4.3743 | 1.22E-05 | *4.23E-05* |
| **EDNRA** | ENSG00000151617 | -2.94 | 0.24579 | -11.963 | 5.57E-33 | *2.65E-31* |
| **EFEMP1** | ENSG00000115380 | -3.45 | 0.34329 | -10.052 | 9.03E-24 | *2.18E-22* |
| **EFEMP2** | ENSG00000172638 | -0.84 | 0.27178 | -3.1086 | 1.88E-03 | *4.50E-03* |
| **EGFL6** | ENSG00000198759 | 1.92 | 0.36716 | 5.2306 | 1.69E-07 | *7.70E-07* |
| **EGFL7** | ENSG00000172889 | -1.19 | 0.30326 | -3.9348 | 8.33E-05 | *2.54E-04* |
| **EGFLAM** | ENSG00000164318 | -1.24 | 0.22849 | -5.4478 | 5.10E-08 | *2.49E-07* |
| **EGFR** | ENSG00000146648 | -0.77 | 0.19615 | -3.9183 | 8.92E-05 | *2.71E-04* |
| **EGLN3** | ENSG00000129521 | 1.97 | 0.25138 | 7.85466 | 4.01E-15 | *4.34E-14* |
| **EIF4B** | ENSG00000063046 | -1.03 | 0.11996 | -8.5817 | 9.35E-18 | *1.30E-16* |
| **EMCN** | ENSG00000164035 | -3.66 | 0.2892 | -12.659 | 9.99E-37 | *5.98E-35* |
| **EMID1** | ENSG00000186998 | 1.82 | 0.31402 | 5.80566 | 6.41E-09 | *3.49E-08* |
| **EMILIN1** | ENSG00000138080 | -2.86 | 0.28106 | -10.166 | 2.80E-24 | *6.99E-23* |
| **EMILIN3** | ENSG00000183798 | -1.84 | 0.38467 | -4.7848 | 1.71E-06 | *6.76E-06* |
| **ENPP2** | ENSG00000136960 | -2.37 | 0.29053 | -8.1672 | 3.16E-16 | *3.78E-15* |
| **EPO** | ENSG00000130427 | -1.60 | 0.40235 | -3.9753 | 7.03E-05 | *2.17E-04* |
| **EPS15** | ENSG00000085832 | -0.87 | 0.10918 | -8.0027 | 1.22E-15 | *1.39E-14* |
| **EPYC** | ENSG00000083782 | 2.39 | 0.57446 | 4.16468 | 3.12E-05 | *1.02E-04* |
| **ERBB2** | ENSG00000141736 | 0.60 | 0.20344 | 2.93737 | 3.31E-03 | *7.54E-03* |
| **ERBB3** | ENSG00000065361 | 1.68 | 0.18847 | 8.89597 | 5.79E-19 | *9.06E-18* |
| **EREG** | ENSG00000124882 | 2.84 | 0.52469 | 5.41884 | 6.00E-08 | *2.91E-07* |
| **ESM1** | ENSG00000164283 | 2.64 | 0.30961 | 8.52115 | 1.58E-17 | *2.16E-16* |
| **EXTL1** | ENSG00000158008 | -3.02 | 0.31873 | -9.4774 | 2.61E-21 | *5.02E-20* |
| **EXTL2** | ENSG00000162694 | -0.68 | 0.15703 | -4.3585 | 1.31E-05 | *4.52E-05* |
| **EYS** | ENSG00000188107 | -0.79 | 0.19092 | -4.1141 | 3.89E-05 | *1.25E-04* |
| **EZR** | ENSG00000092820 | 1.14 | 0.18016 | 6.33172 | 2.42E-10 | *1.56E-09* |
| **F10** | ENSG00000126218 | -2.94 | 0.3593 | -8.1763 | 2.93E-16 | *3.52E-15* |
| **F12** | ENSG00000131187 | 3.64 | 0.33682 | 10.8055 | 3.24E-27 | *1.03E-25* |
| **F2** | ENSG00000180210 | 2.89 | 0.53371 | 5.40916 | 6.33E-08 | *3.06E-07* |
| **FAM20B** | ENSG00000116199 | -0.61 | 0.112 | -5.455 | 4.90E-08 | *2.40E-07* |
| **FAP** | ENSG00000078098 | -2.45 | 0.29603 | -8.2714 | 1.32E-16 | *1.65E-15* |
| **FAS** | ENSG00000026103 | -0.82 | 0.20878 | -3.9435 | 8.03E-05 | *2.46E-04* |
| **FASLG** | ENSG00000117560 | 1.07 | 0.35014 | 3.0428 | 2.34E-03 | *5.51E-03* |
| **FBLN1** | ENSG00000077942 | 1.13 | 0.36017 | 3.138 | 1.70E-03 | *4.10E-03* |
| **FBLN5** | ENSG00000140092 | -2.06 | 0.28838 | -7.1351 | 9.67E-13 | *8.17E-12* |
| **FBN1** | ENSG00000166147 | -2.20 | 0.27039 | -8.1391 | 3.98E-16 | *4.72E-15* |
| **FBN2** | ENSG00000138829 | 3.38 | 0.44903 | 7.52586 | 5.24E-14 | *5.08E-13* |
| **FCN3** | ENSG00000142748 | 1.01 | 0.26044 | 3.87039 | 1.09E-04 | *3.26E-04* |
| **FGF10** | ENSG00000070193 | -3.98 | 0.61391 | -6.4761 | 9.41E-11 | *6.37E-10* |
| **FGF13** | ENSG00000129682 | -1.54 | 0.33481 | -4.599 | 4.24E-06 | *1.58E-05* |
| **FGF16** | ENSG00000196468 | -2.79 | 0.57871 | -4.8242 | 1.41E-06 | *5.62E-06* |
| **FGF18** | ENSG00000156427 | 2.99 | 0.3127 | 9.55188 | 1.27E-21 | *2.51E-20* |
| **FGF19** | ENSG00000162344 | 5.05 | 0.65145 | 7.75513 | 8.83E-15 | *9.21E-14* |
| **FGF2** | ENSG00000138685 | -3.57 | 0.31498 | -11.347 | 7.65E-30 | *2.95E-28* |
| **FGF20** | ENSG00000078579 | 3.73 | 0.73588 | 5.06865 | 4.01E-07 | *1.73E-06* |
| **FGF21** | ENSG00000105550 | 3.69 | 0.80088 | 4.60699 | 4.09E-06 | *1.52E-05* |
| **FGF3** | ENSG00000186895 | 7.36 | 0.98293 | 7.49204 | 6.78E-14 | *6.50E-13* |
| **FGF4** | ENSG00000075388 | 6.73 | 1.04291 | 6.44923 | 1.12E-10 | *7.54E-10* |
| **FGF7** | ENSG00000140285 | -4.48 | 0.45844 | -9.7676 | 1.55E-22 | *3.36E-21* |
| **FGF8** | ENSG00000107831 | 4.63 | 0.61955 | 7.48068 | 7.39E-14 | *7.06E-13* |
| **FGFBP1** | ENSG00000137440 | 3.39 | 0.46001 | 7.36503 | 1.77E-13 | *1.62E-12* |
| **FGFR3** | ENSG00000068078 | 2.11 | 0.40526 | 5.198 | 2.01E-07 | *9.08E-07* |
| **FGFR4** | ENSG00000160867 | 1.83 | 0.34631 | 5.29741 | 1.17E-07 | *5.47E-07* |
| **FGL1** | ENSG00000104760 | 1.59 | 0.5114 | 3.1102 | 1.87E-03 | *4.47E-03* |
| **FGL2** | ENSG00000127951 | -2.18 | 0.31912 | -6.8332 | 8.30E-12 | *6.35E-11* |
| **FIBP** | ENSG00000172500 | 0.99 | 0.11432 | 8.66834 | 4.38E-18 | *6.30E-17* |
| **FLNA** | ENSG00000196924 | -2.98 | 0.24326 | -12.251 | 1.65E-34 | *8.64E-33* |
| **FLNC** | ENSG00000128591 | -4.81 | 0.38449 | -12.499 | 7.59E-36 | *4.36E-34* |
| **FLT3LG** | ENSG00000090554 | -1.15 | 0.2393 | -4.8259 | 1.39E-06 | *5.57E-06* |
| **FMOD** | ENSG00000122176 | -1.27 | 0.32882 | -3.8673 | 1.10E-04 | *3.30E-04* |
| **FN1** | ENSG00000115414 | -1.05 | 0.3271 | -3.2215 | 1.28E-03 | *3.15E-03* |
| **FOS** | ENSG00000170345 | -3.03 | 0.32165 | -9.4101 | 4.96E-21 | *9.34E-20* |
| **FRAS1** | ENSG00000138759 | 1.85 | 0.259 | 7.15274 | 8.51E-13 | *7.24E-12* |
| **FREM1** | ENSG00000164946 | -2.34 | 0.31089 | -7.5398 | 4.71E-14 | *4.58E-13* |
| **FREM2** | ENSG00000150893 | 2.21 | 0.35835 | 6.17205 | 6.74E-10 | *4.12E-09* |
| **FSCN1** | ENSG00000075618 | 0.80 | 0.26031 | 3.05434 | 2.26E-03 | *5.31E-03* |
| **FSTL1** | ENSG00000163430 | -1.13 | 0.20536 | -5.5163 | 3.46E-08 | *1.72E-07* |
| **FSTL3** | ENSG00000070404 | -1.43 | 0.26241 | -5.4682 | 4.55E-08 | *2.23E-07* |
| **FZD10** | ENSG00000111432 | 2.48 | 0.34785 | 7.13991 | 9.34E-13 | *7.91E-12* |
| **FZD2** | ENSG00000180340 | 1.59 | 0.21739 | 7.33499 | 2.22E-13 | *2.01E-12* |
| **FZD3** | ENSG00000104290 | 0.77 | 0.1713 | 4.49541 | 6.94E-06 | *2.51E-05* |
| **FZD4** | ENSG00000174804 | -2.27 | 0.1898 | -11.974 | 4.86E-33 | *2.32E-31* |
| **FZD5** | ENSG00000163251 | 1.89 | 0.2151 | 8.78342 | 1.59E-18 | *2.37E-17* |
| **FZD7** | ENSG00000155760 | -1.91 | 0.36967 | -5.1628 | 2.43E-07 | *1.08E-06* |
| **FZD8** | ENSG00000177283 | 1.55 | 0.32158 | 4.81559 | 1.47E-06 | *5.85E-06* |
| **FZD9** | ENSG00000188763 | 1.49 | 0.31571 | 4.73235 | 2.22E-06 | *8.61E-06* |
| **GAB1** | ENSG00000109458 | -1.80 | 0.15428 | -11.647 | 2.37E-31 | *1.02E-29* |
| **GAD1** | ENSG00000128683 | 5.24 | 0.44903 | 11.6632 | 1.96E-31 | *8.53E-30* |
| **GAL3ST4** | ENSG00000197093 | -0.97 | 0.20379 | -4.773 | 1.82E-06 | *7.14E-06* |
| **GALE** | ENSG00000117308 | 2.26 | 0.15924 | 14.2055 | 8.47E-46 | *9.48E-44* |
| **GALNS** | ENSG00000141012 | 0.76 | 0.14572 | 5.24674 | 1.55E-07 | *7.09E-07* |
| **GAS6** | ENSG00000183087 | -2.82 | 0.28277 | -9.958 | 2.33E-23 | *5.44E-22* |
| **GDF10** | ENSG00000266524 | -2.01 | 0.52707 | -3.8148 | 1.36E-04 | *4.02E-04* |
| **GDF11** | ENSG00000135414 | 1.49 | 0.21879 | 6.78844 | 1.13E-11 | *8.53E-11* |
| **GDF15** | ENSG00000130513 | 3.87 | 0.32724 | 11.8271 | 2.83E-32 | *1.30E-30* |
| **GDF5** | ENSG00000125965 | 3.84 | 0.40144 | 9.57761 | 9.93E-22 | *1.98E-20* |
| **GDF6** | ENSG00000156466 | -2.56 | 0.5246 | -4.8802 | 1.06E-06 | *4.32E-06* |
| **GDF7** | ENSG00000143869 | -1.54 | 0.37855 | -4.0728 | 4.64E-05 | *1.47E-04* |
| **GLB1** | ENSG00000170266 | 1.37 | 0.15337 | 8.90403 | 5.39E-19 | *8.46E-18* |
| **GPC2** | ENSG00000213420 | 2.38 | 0.30092 | 7.91479 | 2.48E-15 | *2.75E-14* |
| **GPC6** | ENSG00000183098 | -1.05 | 0.34699 | -3.0144 | 2.57E-03 | *6.00E-03* |
| **GPD1** | ENSG00000167588 | 1.04 | 0.31203 | 3.33879 | 8.41E-04 | *2.15E-03* |
| **GPLD1** | ENSG00000112293 | -1.40 | 0.21357 | -6.5473 | 5.86E-11 | *4.07E-10* |
| **GREM1** | ENSG00000166923 | -2.92 | 0.3777 | -7.7398 | 9.96E-15 | *1.03E-13* |
| **GRIP1** | ENSG00000155974 | 1.01 | 0.22494 | 4.47894 | 7.50E-06 | *2.69E-05* |
| **GRIP2** | ENSG00000144596 | -1.02 | 0.32023 | -3.1707 | 1.52E-03 | *3.70E-03* |
| **GYPC** | ENSG00000136732 | -2.40 | 0.27065 | -8.8844 | 6.43E-19 | *1.00E-17* |
| **GZMA** | ENSG00000145649 | 1.07 | 0.33273 | 3.2216 | 1.27E-03 | *3.15E-03* |
| **GZMB** | ENSG00000100453 | 2.34 | 0.39158 | 5.96884 | 2.39E-09 | *1.37E-08* |
| **HABP2** | ENSG00000148702 | 2.24 | 0.51936 | 4.30437 | 1.67E-05 | *5.69E-05* |
| **HAPLN1** | ENSG00000145681 | 5.73 | 0.32966 | 17.3935 | 9.25E-68 | *2.73E-65* |
| **HAPLN2** | ENSG00000132702 | -2.30 | 0.44794 | -5.1284 | 2.92E-07 | *1.29E-06* |
| **HAPLN3** | ENSG00000140511 | 2.28 | 0.27523 | 8.28269 | 1.20E-16 | *1.51E-15* |
| **HAS1** | ENSG00000105509 | -3.76 | 0.41906 | -8.9678 | 3.02E-19 | *4.87E-18* |
| **HBEGF** | ENSG00000113070 | -2.23 | 0.19919 | -11.22 | 3.27E-29 | *1.21E-27* |
| **HCFC2** | ENSG00000111727 | -1.65 | 0.13626 | -12.137 | 6.74E-34 | *3.41E-32* |
| **HCLS1** | ENSG00000180353 | 0.99 | 0.26569 | 3.72912 | 1.92E-04 | *5.52E-04* |
| **HGF** | ENSG00000019991 | -2.38 | 0.31822 | -7.4734 | 7.81E-14 | *7.45E-13* |
| **HGFAC** | ENSG00000109758 | 2.33 | 0.31379 | 7.41693 | 1.20E-13 | *1.12E-12* |
| **HHIP** | ENSG00000164161 | 1.68 | 0.4217 | 3.97591 | 7.01E-05 | *2.17E-04* |
| **HMCN1** | ENSG00000143341 | 1.20 | 0.35169 | 3.41545 | 6.37E-04 | *1.66E-03* |
| **HMCN2** | ENSG00000148357 | -3.21 | 0.35652 | -8.9962 | 2.34E-19 | *3.81E-18* |
| **HMMR** | ENSG00000072571 | 3.48 | 0.23983 | 14.4925 | 1.35E-47 | *1.67E-45* |
| **HOXD10** | ENSG00000128710 | -1.59 | 0.30834 | -5.1521 | 2.58E-07 | *1.14E-06* |
| **HPSE** | ENSG00000173083 | -1.08 | 0.28121 | -3.844 | 1.21E-04 | *3.60E-04* |
| **HPSE2** | ENSG00000172987 | -2.21 | 0.53955 | -4.1046 | 4.05E-05 | *1.30E-04* |
| **HPX** | ENSG00000110169 | 1.20 | 0.30738 | 3.91343 | 9.10E-05 | *2.76E-04* |
| **HRAS** | ENSG00000174775 | 1.35 | 0.1832 | 7.35058 | 1.97E-13 | *1.79E-12* |
| **HRG** | ENSG00000113905 | 2.44 | 0.62801 | 3.88026 | 1.04E-04 | *3.14E-04* |
| **HS3ST5** | ENSG00000249853 | 1.83 | 0.51676 | 3.54173 | 3.98E-04 | *1.08E-03* |
| **HS6ST1** | ENSG00000136720 | 1.33 | 0.18235 | 7.26779 | 3.65E-13 | *3.23E-12* |
| **HS6ST2** | ENSG00000171004 | -1.29 | 0.33013 | -3.9191 | 8.89E-05 | *2.70E-04* |
| **HSPB2** | ENSG00000170276 | -3.14 | 0.34329 | -9.1408 | 6.20E-20 | *1.06E-18* |
| **HSPG2** | ENSG00000142798 | -1.05 | 0.28142 | -3.7295 | 1.92E-04 | *5.51E-04* |
| **HTRA1** | ENSG00000166033 | -0.83 | 0.22153 | -3.7664 | 1.66E-04 | *4.81E-04* |
| **HTRA3** | ENSG00000170801 | -1.60 | 0.29772 | -5.3722 | 7.78E-08 | *3.70E-07* |
| **HTRA4** | ENSG00000169495 | 1.66 | 0.34739 | 4.77312 | 1.81E-06 | *7.13E-06* |
| **HYAL1** | ENSG00000114378 | -1.68 | 0.27083 | -6.1972 | 5.75E-10 | *3.54E-09* |
| **HYAL3** | ENSG00000186792 | 1.44 | 0.19141 | 7.50553 | 6.12E-14 | *5.89E-13* |
| **IBSP** | ENSG00000029559 | 3.22 | 0.50848 | 6.33006 | 2.45E-10 | *1.57E-09* |
| **IFNG** | ENSG00000111537 | 2.38 | 0.51005 | 4.66559 | 3.08E-06 | *1.17E-05* |
| **IGF1R** | ENSG00000140443 | -0.69 | 0.23285 | -2.9632 | 3.04E-03 | *6.99E-03* |
| **IGFBP2** | ENSG00000115457 | 1.58 | 0.31585 | 4.99081 | 6.01E-07 | *2.53E-06* |
| **IGFBP4** | ENSG00000141753 | -1.40 | 0.29881 | -4.6718 | 2.99E-06 | *1.14E-05* |
| **IGFBP5** | ENSG00000115461 | -3.09 | 0.32067 | -9.6441 | 5.20E-22 | *1.06E-20* |
| **IGFBP6** | ENSG00000167779 | -3.17 | 0.34255 | -9.2552 | 2.14E-20 | *3.82E-19* |
| **IGFBP7** | ENSG00000163453 | -1.18 | 0.22056 | -5.3507 | 8.76E-08 | *4.14E-07* |
| **IGFBPL1** | ENSG00000137142 | 2.19 | 0.46414 | 4.7266 | 2.28E-06 | *8.84E-06* |
| **IGSF10** | ENSG00000152580 | -2.65 | 0.36415 | -7.2807 | 3.32E-13 | *2.96E-12* |
| **IHH** | ENSG00000163501 | 2.89 | 0.56176 | 5.14191 | 2.72E-07 | *1.20E-06* |
| **IL10** | ENSG00000136634 | 1.13 | 0.38151 | 2.95143 | 3.16E-03 | *7.23E-03* |
| **IL11** | ENSG00000095752 | 2.52 | 0.35218 | 7.15853 | 8.15E-13 | *6.94E-12* |
| **IL16** | ENSG00000172349 | -0.77 | 0.25576 | -3.002 | 2.68E-03 | *6.23E-03* |
| **IL17C** | ENSG00000124391 | 2.33 | 0.4722 | 4.94464 | 7.63E-07 | *3.16E-06* |
| **IL17D** | ENSG00000172458 | -1.30 | 0.18108 | -7.1801 | 6.96E-13 | *5.96E-12* |
| **IL18** | ENSG00000150782 | 1.28 | 0.26673 | 4.79373 | 1.64E-06 | *6.48E-06* |
| **IL19** | ENSG00000142224 | 4.92 | 0.51841 | 9.49385 | 2.23E-21 | *4.32E-20* |
| **IL1A** | ENSG00000115008 | 1.49 | 0.42886 | 3.46326 | 5.34E-04 | *1.41E-03* |
| **IL1RN** | ENSG00000136689 | 2.37 | 0.34298 | 6.89629 | 5.34E-12 | *4.17E-11* |
| **IL23A** | ENSG00000110944 | 1.94 | 0.29136 | 6.66474 | 2.65E-11 | *1.91E-10* |
| **IL34** | ENSG00000157368 | -1.15 | 0.25557 | -4.5118 | 6.43E-06 | *2.33E-05* |
| **IL5** | ENSG00000113525 | -1.53 | 0.41515 | -3.6802 | 2.33E-04 | *6.59E-04* |
| **IL6** | ENSG00000136244 | -2.88 | 0.41205 | -6.9984 | 2.59E-12 | *2.09E-11* |
| **IL6R** | ENSG00000160712 | -1.00 | 0.24686 | -4.0607 | 4.89E-05 | *1.55E-04* |
| **IL6ST** | ENSG00000134352 | -1.48 | 0.16959 | -8.7541 | 2.06E-18 | *3.05E-17* |
| **IL7** | ENSG00000104432 | -0.85 | 0.28842 | -2.9482 | 3.20E-03 | *7.30E-03* |
| **IMPG2** | ENSG00000081148 | -3.78 | 0.38187 | -9.8983 | 4.23E-23 | *9.67E-22* |
| **INHBA** | ENSG00000122641 | -1.02 | 0.32228 | -3.1563 | 1.60E-03 | *3.87E-03* |
| **INHBE** | ENSG00000139269 | 1.30 | 0.38538 | 3.38343 | 7.16E-04 | *1.85E-03* |
| **IQGAP1** | ENSG00000140575 | -1.04 | 0.13191 | -7.9203 | 2.37E-15 | *2.63E-14* |
| **IRS1** | ENSG00000169047 | -1.60 | 0.25378 | -6.3026 | 2.93E-10 | *1.87E-09* |
| **ISM1** | ENSG00000101230 | 1.27 | 0.32074 | 3.95991 | 7.50E-05 | *2.31E-04* |
| **ISM2** | ENSG00000100593 | -1.86 | 0.35576 | -5.2152 | 1.84E-07 | *8.32E-07* |
| **ITGA2B** | ENSG00000005961 | 0.86 | 0.29184 | 2.93006 | 3.39E-03 | *7.71E-03* |
| **ITGA5** | ENSG00000161638 | -1.13 | 0.18443 | -6.1427 | 8.11E-10 | *4.90E-09* |
| **ITGA7** | ENSG00000135424 | -2.93 | 0.27024 | -10.825 | 2.62E-27 | *8.39E-26* |
| **ITGA8** | ENSG00000077943 | -3.48 | 0.26329 | -13.2 | 8.75E-40 | *6.65E-38* |
| **ITGA9** | ENSG00000144668 | -1.23 | 0.2833 | -4.3311 | 1.48E-05 | *5.08E-05* |
| **ITGAV** | ENSG00000138448 | -1.13 | 0.20346 | -5.5387 | 3.05E-08 | *1.53E-07* |
| **ITGAX** | ENSG00000140678 | 1.31 | 0.25984 | 5.02327 | 5.08E-07 | *2.16E-06* |
| **ITGB1** | ENSG00000150093 | -1.10 | 0.19931 | -5.5434 | 2.97E-08 | *1.49E-07* |
| **ITGB3** | ENSG00000259207 | -1.54 | 0.41113 | -3.7562 | 1.73E-04 | *5.00E-04* |
| **ITGB6** | ENSG00000115221 | 2.12 | 0.33196 | 6.38819 | 1.68E-10 | *1.10E-09* |
| **ITIH2** | ENSG00000151655 | 2.00 | 0.48412 | 4.1282 | 3.66E-05 | *1.18E-04* |
| **ITIH3** | ENSG00000162267 | -1.44 | 0.31435 | -4.5728 | 4.81E-06 | *1.78E-05* |
| **ITIH6** | ENSG00000102313 | 2.57 | 0.68139 | 3.7649 | 1.67E-04 | *4.84E-04* |
| **ITLN1** | ENSG00000179914 | -1.70 | 0.5257 | -3.2321 | 1.23E-03 | *3.04E-03* |
| **ITLN2** | ENSG00000158764 | 2.13 | 0.60787 | 3.50229 | 4.61E-04 | *1.24E-03* |
| **ITPR1** | ENSG00000150995 | -3.31 | 0.23626 | -13.998 | 1.60E-44 | *1.66E-42* |
| **ITPR3** | ENSG00000096433 | 1.38 | 0.24917 | 5.54545 | 2.93E-08 | *1.47E-07* |
| **JPH4** | ENSG00000092051 | -5.00 | 0.36373 | -13.743 | 5.60E-43 | *5.31E-41* |
| **JUN** | ENSG00000177606 | -1.49 | 0.20671 | -7.2063 | 5.75E-13 | *4.97E-12* |
| **KAZALD1** | ENSG00000107821 | 2.61 | 0.30181 | 8.63797 | 5.72E-18 | *8.12E-17* |
| **KCP** | ENSG00000135253 | 1.75 | 0.39981 | 4.37949 | 1.19E-05 | *4.14E-05* |
| **KDR** | ENSG00000128052 | -2.52 | 0.20807 | -12.097 | 1.09E-33 | *5.48E-32* |
| **KITLG** | ENSG00000049130 | -1.20 | 0.30728 | -3.8995 | 9.64E-05 | *2.91E-04* |
| **KRT15** | ENSG00000171346 | 3.28 | 0.39474 | 8.30311 | 1.01E-16 | *1.28E-15* |
| **KY** | ENSG00000174611 | -3.16 | 0.375 | -8.4375 | 3.24E-17 | *4.27E-16* |
| **LAMA1** | ENSG00000101680 | 3.71 | 0.3763 | 9.85886 | 6.28E-23 | *1.41E-21* |
| **LAMA2** | ENSG00000196569 | -3.10 | 0.30661 | -10.122 | 4.43E-24 | *1.09E-22* |
| **LAMA4** | ENSG00000112769 | -2.68 | 0.2196 | -12.207 | 2.87E-34 | *1.49E-32* |
| **LAMA5** | ENSG00000130702 | 0.79 | 0.18607 | 4.26627 | 1.99E-05 | *6.69E-05* |
| **LAMB2** | ENSG00000172037 | -0.81 | 0.18707 | -4.3563 | 1.32E-05 | *4.57E-05* |
| **LAMB4** | ENSG00000091128 | 2.13 | 0.2996 | 7.12589 | 1.03E-12 | *8.70E-12* |
| **LAMC2** | ENSG00000058085 | 1.29 | 0.26389 | 4.87452 | 1.09E-06 | *4.43E-06* |
| **LAMC3** | ENSG00000050555 | 1.79 | 0.32693 | 5.47493 | 4.38E-08 | *2.15E-07* |
| **LEFTY1** | ENSG00000243709 | 6.52 | 0.55269 | 11.8055 | 3.66E-32 | *1.67E-30* |
| **LEFTY2** | ENSG00000143768 | -4.16 | 0.54453 | -7.6454 | 2.08E-14 | *2.10E-13* |
| **LGALS7** | ENSG00000205076 | 3.78 | 0.80737 | 4.68806 | 2.76E-06 | *1.06E-05* |
| **LGALS9** | ENSG00000168961 | 1.52 | 0.23644 | 6.42136 | 1.35E-10 | *8.97E-10* |
| **LGALS9B** | ENSG00000170298 | 2.55 | 0.52094 | 4.89206 | 9.98E-07 | *4.08E-06* |
| **LGALS9C** | ENSG00000171916 | 2.25 | 0.46357 | 4.85355 | 1.21E-06 | *4.89E-06* |
| **LGI1** | ENSG00000108231 | -1.80 | 0.5231 | -3.4442 | 5.73E-04 | *1.51E-03* |
| **LGI2** | ENSG00000153012 | -3.55 | 0.34747 | -10.226 | 1.52E-24 | *3.86E-23* |
| **LGI3** | ENSG00000168481 | -3.41 | 0.48018 | -7.0961 | 1.28E-12 | *1.07E-11* |
| **LGI4** | ENSG00000153902 | -3.94 | 0.28369 | -13.902 | 6.18E-44 | *6.19E-42* |
| **LIF** | ENSG00000128342 | 1.46 | 0.31203 | 4.66494 | 3.09E-06 | *1.17E-05* |
| **LMAN1L** | ENSG00000140506 | 3.55 | 0.869 | 4.08429 | 4.42E-05 | *1.41E-04* |
| **LONP1** | ENSG00000196365 | 1.12 | 0.15907 | 7.05518 | 1.72E-12 | *1.42E-11* |
| **LOXL1** | ENSG00000129038 | 0.70 | 0.23575 | 2.9616 | 3.06E-03 | *7.02E-03* |
| **LOXL2** | ENSG00000134013 | 1.07 | 0.24105 | 4.42265 | 9.75E-06 | *3.44E-05* |
| **LOXL3** | ENSG00000115318 | -0.66 | 0.18197 | -3.653 | 2.59E-04 | *7.27E-04* |
| **LOXL4** | ENSG00000138131 | -1.91 | 0.33799 | -5.6417 | 1.68E-08 | *8.68E-08* |
| **LRG1** | ENSG00000171236 | 1.30 | 0.31519 | 4.11889 | 3.81E-05 | *1.23E-04* |
| **LRP1** | ENSG00000123384 | -1.80 | 0.2255 | -7.9877 | 1.37E-15 | *1.56E-14* |
| **LRP4** | ENSG00000134569 | 2.96 | 0.4468 | 6.63536 | 3.24E-11 | *2.31E-10* |
| **LTA** | ENSG00000226979 | 1.96 | 0.32809 | 5.98767 | 2.13E-09 | *1.22E-08* |
| **LTB** | ENSG00000227507 | 2.86 | 0.35759 | 8.00455 | 1.20E-15 | *1.37E-14* |
| **LTBP1** | ENSG00000049323 | -0.93 | 0.23446 | -3.9695 | 7.20E-05 | *2.23E-04* |
| **LTBP2** | ENSG00000119681 | -1.62 | 0.2368 | -6.8292 | 8.54E-12 | *6.53E-11* |
| **LTBP3** | ENSG00000168056 | -0.95 | 0.2262 | -4.1854 | 2.85E-05 | *9.36E-05* |
| **LTBP4** | ENSG00000090006 | -3.06 | 0.30952 | -9.8855 | 4.81E-23 | *1.09E-21* |
| **LTF** | ENSG00000012223 | 3.72 | 0.60852 | 6.11714 | 9.53E-10 | *5.69E-09* |
| **LUM** | ENSG00000139329 | -1.65 | 0.30152 | -5.4618 | 4.71E-08 | *2.31E-07* |
| **MAG** | ENSG00000105695 | -4.30 | 0.5925 | -7.2525 | 4.09E-13 | *3.59E-12* |
| **MAMDC2** | ENSG00000165072 | -5.50 | 0.40093 | -13.729 | 6.85E-43 | *6.37E-41* |
| **MAP2K2** | ENSG00000126934 | 0.83 | 0.153 | 5.45441 | 4.91E-08 | *2.40E-07* |
| **MAPK10** | ENSG00000109339 | -2.49 | 0.3145 | -7.9098 | 2.58E-15 | *2.85E-14* |
| **MAPK11** | ENSG00000185386 | -0.90 | 0.25758 | -3.5065 | 4.54E-04 | *1.22E-03* |
| **MAPK13** | ENSG00000156711 | 1.92 | 0.17112 | 11.2381 | 2.65E-29 | *9.86E-28* |
| **MAPK14** | ENSG00000112062 | -0.81 | 0.09518 | -8.4939 | 2.00E-17 | *2.69E-16* |
| **MAPK3** | ENSG00000102882 | -0.90 | 0.14854 | -6.0875 | 1.15E-09 | *6.79E-09* |
| **MASP1** | ENSG00000127241 | -4.75 | 0.40714 | -11.677 | 1.67E-31 | *7.30E-30* |
| **MASP2** | ENSG00000009724 | -1.46 | 0.23307 | -6.2498 | 4.11E-10 | *2.58E-09* |
| **MATN1** | ENSG00000162510 | 1.04 | 0.3359 | 3.10482 | 1.90E-03 | *4.55E-03* |
| **MATN2** | ENSG00000132561 | -1.70 | 0.31406 | -5.4047 | 6.49E-08 | *3.13E-07* |
| **MATN4** | ENSG00000124159 | 1.92 | 0.39276 | 4.87789 | 1.07E-06 | *4.37E-06* |
| **MDK** | ENSG00000110492 | 2.12 | 0.22913 | 9.25453 | 2.15E-20 | *3.84E-19* |
| **MEGF10** | ENSG00000145794 | -1.87 | 0.38438 | -4.8601 | 1.17E-06 | *4.75E-06* |
| **MEGF9** | ENSG00000106780 | -0.63 | 0.18016 | -3.4811 | 4.99E-04 | *1.33E-03* |
| **MEP1A** | ENSG00000112818 | 1.82 | 0.49041 | 3.7068 | 2.10E-04 | *5.99E-04* |
| **MET** | ENSG00000105976 | 0.96 | 0.27931 | 3.43352 | 5.96E-04 | *1.56E-03* |
| **MFAP2** | ENSG00000117122 | 1.95 | 0.29734 | 6.56887 | 5.07E-11 | *3.55E-10* |
| **MFAP4** | ENSG00000166482 | -3.28 | 0.42315 | -7.7536 | 8.93E-15 | *9.32E-14* |
| **MFAP5** | ENSG00000197614 | -3.16 | 0.47892 | -6.5951 | 4.25E-11 | *3.00E-10* |
| **MFGE8** | ENSG00000140545 | -0.92 | 0.23832 | -3.8635 | 1.12E-04 | *3.34E-04* |
| **MGP** | ENSG00000111341 | -3.14 | 0.28583 | -10.974 | 5.09E-28 | *1.71E-26* |
| **MMP1** | ENSG00000196611 | 4.57 | 0.42973 | 10.6388 | 1.97E-26 | *5.88E-25* |
| **MMP10** | ENSG00000166670 | 3.71 | 0.52657 | 7.04093 | 1.91E-12 | *1.56E-11* |
| **MMP11** | ENSG00000099953 | 2.75 | 0.28699 | 9.59817 | 8.14E-22 | *1.64E-20* |
| **MMP12** | ENSG00000262406 | 4.06 | 0.41491 | 9.79279 | 1.21E-22 | *2.66E-21* |
| **MMP13** | ENSG00000137745 | 4.07 | 0.69842 | 5.83166 | 5.49E-09 | *3.01E-08* |
| **MMP15** | ENSG00000102996 | 1.56 | 0.17054 | 9.13905 | 6.30E-20 | *1.08E-18* |
| **MMP16** | ENSG00000156103 | -1.39 | 0.33154 | -4.1856 | 2.84E-05 | *9.35E-05* |
| **MMP19** | ENSG00000123342 | -1.77 | 0.24124 | -7.3544 | 1.92E-13 | *1.75E-12* |
| **MMP21** | ENSG00000154485 | -1.11 | 0.31909 | -3.4633 | 5.34E-04 | *1.41E-03* |
| **MMP23B** | ENSG00000189409 | -1.75 | 0.36927 | -4.7433 | 2.10E-06 | *8.19E-06* |
| **MMP24** | ENSG00000125966 | -1.56 | 0.22373 | -6.9696 | 3.18E-12 | *2.54E-11* |
| **MMP25** | ENSG00000008516 | 1.27 | 0.29183 | 4.34195 | 1.41E-05 | *4.85E-05* |
| **MMP26** | ENSG00000167346 | 2.47 | 0.82243 | 3.00118 | 2.69E-03 | *6.24E-03* |
| **MMP28** | ENSG00000271447 | -4.03 | 0.31046 | -12.987 | 1.45E-38 | *1.02E-36* |
| **MMP3** | ENSG00000149968 | 2.45 | 0.47808 | 5.1298 | 2.90E-07 | *1.28E-06* |
| **MMP7** | ENSG00000137673 | 1.39 | 0.46754 | 2.9725 | 2.95E-03 | *6.80E-03* |
| **MMP8** | ENSG00000118113 | 3.47 | 0.69516 | 4.9935 | 5.93E-07 | *2.50E-06* |
| **MMP9** | ENSG00000100985 | 3.83 | 0.35276 | 10.8453 | 2.10E-27 | *6.79E-26* |
| **MMRN1** | ENSG00000138722 | -3.12 | 0.34098 | -9.1368 | 6.43E-20 | *1.10E-18* |
| **MMRN2** | ENSG00000173269 | -2.53 | 0.20722 | -12.222 | 2.36E-34 | *1.23E-32* |
| **MPDZ** | ENSG00000107186 | -1.94 | 0.1883 | -10.328 | 5.25E-25 | *1.38E-23* |
| **MRAS** | ENSG00000158186 | -1.44 | 0.20752 | -6.9306 | 4.19E-12 | *3.30E-11* |
| **MSN** | ENSG00000147065 | -0.86 | 0.16098 | -5.3342 | 9.59E-08 | *4.52E-07* |
| **MST1** | ENSG00000173531 | 1.14 | 0.24455 | 4.67458 | 2.95E-06 | *1.12E-05* |
| **MST1L** | ENSG00000186715 | 1.14 | 0.3291 | 3.45861 | 5.43E-04 | *1.44E-03* |
| **MSTN** | ENSG00000138379 | -1.32 | 0.40983 | -3.2109 | 1.32E-03 | *3.25E-03* |
| **MT3** | ENSG00000087250 | 3.76 | 0.46967 | 8.00628 | 1.18E-15 | *1.35E-14* |
| **MTOR** | ENSG00000198793 | 0.87 | 0.12593 | 6.87727 | 6.10E-12 | *4.74E-11* |
| **MUC1** | ENSG00000185499 | 1.81 | 0.28943 | 6.2365 | 4.47E-10 | *2.79E-09* |
| **MUC13** | ENSG00000173702 | 2.62 | 0.50888 | 5.15423 | 2.55E-07 | *1.13E-06* |
| **MUC16** | ENSG00000181143 | 1.58 | 0.40564 | 3.8998 | 9.63E-05 | *2.91E-04* |
| **MUC2** | ENSG00000198788 | 4.36 | 0.49987 | 8.72133 | 2.75E-18 | *4.01E-17* |
| **MUC20** | ENSG00000176945 | 1.09 | 0.33151 | 3.2767 | 1.05E-03 | *2.64E-03* |
| **MUC21** | ENSG00000204544 | 3.27 | 0.59308 | 5.51794 | 3.43E-08 | *1.71E-07* |
| **MUC5AC** | ENSG00000215182 | 6.62 | 0.6001 | 11.038 | 2.50E-28 | *8.60E-27* |
| **MUC5B** | ENSG00000117983 | 4.57 | 0.59277 | 7.70926 | 1.27E-14 | *1.30E-13* |
| **MUC7** | ENSG00000171195 | 5.86 | 0.91784 | 6.37992 | 1.77E-10 | *1.16E-09* |
| **MUCL1** | ENSG00000172551 | 3.04 | 0.69733 | 4.35581 | 1.33E-05 | *4.58E-05* |
| **MYC** | ENSG00000136997 | -1.50 | 0.24804 | -6.0324 | 1.62E-09 | *9.43E-09* |
| **MYD88** | ENSG00000172936 | 0.72 | 0.11488 | 6.30353 | 2.91E-10 | *1.85E-09* |
| **MYDGF** | ENSG00000074842 | 1.31 | 0.16286 | 8.04785 | 8.43E-16 | *9.74E-15* |
| **NCAM1** | ENSG00000149294 | -4.28 | 0.40616 | -10.546 | 5.27E-26 | *1.51E-24* |
| **NDNF** | ENSG00000173376 | -2.64 | 0.36422 | -7.2387 | 4.53E-13 | *3.95E-12* |
| **NDP** | ENSG00000124479 | -1.47 | 0.36198 | -4.0617 | 4.87E-05 | *1.54E-04* |
| **NDST4** | ENSG00000138653 | -3.29 | 0.65397 | -5.0252 | 5.03E-07 | *2.14E-06* |
| **NELL1** | ENSG00000165973 | 1.86 | 0.60961 | 3.05737 | 2.23E-03 | *5.26E-03* |
| **NELL2** | ENSG00000184613 | -1.89 | 0.42032 | -4.4919 | 7.06E-06 | *2.55E-05* |
| **NES** | ENSG00000132688 | -1.79 | 0.24275 | -7.3692 | 1.72E-13 | *1.57E-12* |
| **NEU1** | ENSG00000204386 | 1.41 | 0.13386 | 10.5135 | 7.49E-26 | *2.12E-24* |
| **NGF** | ENSG00000134259 | -2.89 | 0.32509 | -8.8923 | 5.99E-19 | *9.35E-18* |
| **NID1** | ENSG00000116962 | -1.35 | 0.25365 | -5.3053 | 1.12E-07 | *5.25E-07* |
| **NID2** | ENSG00000087303 | -1.05 | 0.31922 | -3.3038 | 9.54E-04 | *2.42E-03* |
| **NOG** | ENSG00000183691 | 3.08 | 0.38597 | 7.96859 | 1.60E-15 | *1.81E-14* |
| **NPNT** | ENSG00000168743 | -1.16 | 0.32888 | -3.5305 | 4.15E-04 | *1.12E-03* |
| **NRAS** | ENSG00000213281 | 0.84 | 0.15887 | 5.31177 | 1.09E-07 | *5.08E-07* |
| **NRG2** | ENSG00000158458 | -1.36 | 0.37595 | -3.6205 | 2.94E-04 | *8.18E-04* |
| **NRG3** | ENSG00000185737 | -3.87 | 0.55719 | -6.9462 | 3.75E-12 | *2.97E-11* |
| **NRTN** | ENSG00000171119 | 2.92 | 0.35339 | 8.24977 | 1.59E-16 | *1.96E-15* |
| **NTN4** | ENSG00000074527 | -1.58 | 0.24914 | -6.3455 | 2.22E-10 | *1.43E-09* |
| **NUDT16L1** | ENSG00000168101 | 0.83 | 0.13557 | 6.14031 | 8.24E-10 | *4.96E-09* |
| **OGN** | ENSG00000106809 | -5.52 | 0.57462 | -9.6013 | 7.89E-22 | *1.59E-20* |
| **OMD** | ENSG00000127083 | -4.66 | 0.55033 | -8.4736 | 2.38E-17 | *3.19E-16* |
| **OSM** | ENSG00000099985 | 0.80 | 0.27359 | 2.92168 | 3.48E-03 | *7.90E-03* |
| **OTOG** | ENSG00000188162 | 3.34 | 0.54098 | 6.18004 | 6.41E-10 | *3.92E-09* |
| **OVGP1** | ENSG00000085465 | -2.43 | 0.60984 | -3.9866 | 6.70E-05 | *2.08E-04* |
| **P3H1** | ENSG00000117385 | 0.94 | 0.14017 | 6.72653 | 1.74E-11 | *1.28E-10* |
| **P3H3** | ENSG00000110811 | -1.11 | 0.30608 | -3.6179 | 2.97E-04 | *8.25E-04* |
| **P4HA1** | ENSG00000122884 | 0.69 | 0.17059 | 4.02251 | 5.76E-05 | *1.80E-04* |
| **P4HA3** | ENSG00000149380 | -0.91 | 0.24455 | -3.7132 | 2.05E-04 | *5.85E-04* |
| **PAK1** | ENSG00000149269 | 0.71 | 0.09405 | 7.5195 | 5.50E-14 | *5.32E-13* |
| **PAMR1** | ENSG00000149090 | -1.56 | 0.32253 | -4.8236 | 1.41E-06 | *5.63E-06* |
| **PAPLN** | ENSG00000100767 | -0.84 | 0.28044 | -2.9987 | 2.71E-03 | *6.29E-03* |
| **PAPPA** | ENSG00000182752 | -2.71 | 0.35927 | -7.5528 | 4.26E-14 | *4.16E-13* |
| **PARM1** | ENSG00000169116 | -1.48 | 0.32149 | -4.6162 | 3.91E-06 | *1.46E-05* |
| **PCOLCE2** | ENSG00000163710 | -2.53 | 0.44345 | -5.71 | 1.13E-08 | *5.95E-08* |
| **PCSK5** | ENSG00000099139 | -0.93 | 0.30858 | -3.0295 | 2.45E-03 | *5.73E-03* |
| **PDCD4** | ENSG00000150593 | -0.95 | 0.14692 | -6.4446 | 1.16E-10 | *7.75E-10* |
| **PDGFC** | ENSG00000145431 | -1.15 | 0.17145 | -6.7132 | 1.90E-11 | *1.40E-10* |
| **PDGFD** | ENSG00000170962 | -1.80 | 0.26093 | -6.8964 | 5.33E-12 | *4.17E-11* |
| **PDGFRA** | ENSG00000134853 | -2.78 | 0.30619 | -9.095 | 9.46E-20 | *1.60E-18* |
| **PDPK1** | ENSG00000140992 | -0.69 | 0.1119 | -6.1609 | 7.23E-10 | *4.40E-09* |
| **PDX1** | ENSG00000139515 | 5.51 | 0.82131 | 6.70722 | 1.98E-11 | *1.45E-10* |
| **PF4V1** | ENSG00000109272 | 4.66 | 0.6724 | 6.93228 | 4.14E-12 | *3.27E-11* |
| **PHEX** | ENSG00000102174 | -1.51 | 0.34813 | -4.3492 | 1.37E-05 | *4.71E-05* |
| **PI3** | ENSG00000124102 | 4.92 | 0.50404 | 9.7622 | 1.64E-22 | *3.53E-21* |
| **PIK3CA** | ENSG00000121879 | -0.75 | 0.18931 | -3.9368 | 8.26E-05 | *2.52E-04* |
| **PIK3IP1** | ENSG00000100100 | -1.37 | 0.16086 | -8.536 | 1.39E-17 | *1.91E-16* |
| **PIK3R2** | ENSG00000105647 | 1.65 | 0.21557 | 7.65242 | 1.97E-14 | *1.99E-13* |
| **PLA2G10** | ENSG00000069764 | 2.46 | 0.37819 | 6.50477 | 7.78E-11 | *5.33E-10* |
| **PLA2G2A** | ENSG00000188257 | -3.54 | 0.58126 | -6.0911 | 1.12E-09 | *6.64E-09* |
| **PLA2G2D** | ENSG00000117215 | 2.35 | 0.47915 | 4.90732 | 9.23E-07 | *3.79E-06* |
| **PLA2G3** | ENSG00000100078 | 5.37 | 0.38002 | 14.135 | 2.31E-45 | *2.52E-43* |
| **PLAT** | ENSG00000104368 | -1.51 | 0.31846 | -4.7306 | 2.24E-06 | *8.67E-06* |
| **PLAU** | ENSG00000122861 | 1.13 | 0.25045 | 4.52936 | 5.92E-06 | *2.16E-05* |
| **PLAUR** | ENSG00000011422 | 1.58 | 0.22703 | 6.97484 | 3.06E-12 | *2.45E-11* |
| **PLG** | ENSG00000122194 | -1.70 | 0.58571 | -2.9032 | 3.69E-03 | *8.34E-03* |
| **PLOD1** | ENSG00000083444 | 1.46 | 0.14594 | 10.0261 | 1.17E-23 | *2.80E-22* |
| **PLOD2** | ENSG00000152952 | 0.75 | 0.23488 | 3.20215 | 1.36E-03 | *3.34E-03* |
| **PLXDC1** | ENSG00000161381 | -1.81 | 0.2092 | -8.6384 | 5.70E-18 | *8.09E-17* |
| **PLXDC2** | ENSG00000120594 | -1.65 | 0.24894 | -6.6311 | 3.33E-11 | *2.37E-10* |
| **PLXNA1** | ENSG00000114554 | 0.78 | 0.18112 | 4.31173 | 1.62E-05 | *5.53E-05* |
| **PLXNA3** | ENSG00000130827 | 0.64 | 0.15724 | 4.06391 | 4.83E-05 | *1.53E-04* |
| **PLXNB1** | ENSG00000164050 | 0.68 | 0.16733 | 4.06706 | 4.76E-05 | *1.51E-04* |
| **PLXNC1** | ENSG00000136040 | -1.06 | 0.24356 | -4.3473 | 1.38E-05 | *4.74E-05* |
| **PLXND1** | ENSG00000004399 | -0.95 | 0.19094 | -4.9611 | 7.01E-07 | *2.92E-06* |
| **PODN** | ENSG00000174348 | -3.99 | 0.3815 | -10.464 | 1.26E-25 | *3.50E-24* |
| **POMGNT2** | ENSG00000144647 | -1.12 | 0.17157 | -6.5042 | 7.81E-11 | *5.34E-10* |
| **POSTN** | ENSG00000133110 | -2.72 | 0.37408 | -7.2819 | 3.29E-13 | *2.93E-12* |
| **PPARA** | ENSG00000186951 | -0.69 | 0.13169 | -5.2572 | 1.46E-07 | *6.72E-07* |
| **PPBP** | ENSG00000163736 | 1.84 | 0.60383 | 3.0541 | 2.26E-03 | *5.32E-03* |
| **PPP1CA** | ENSG00000172531 | 1.54 | 0.11556 | 13.3208 | 1.75E-40 | *1.42E-38* |
| **PPP1R12A** | ENSG00000058272 | -1.59 | 0.13528 | -11.787 | 4.54E-32 | *2.06E-30* |
| **PPP1R12B** | ENSG00000077157 | -4.55 | 0.25475 | -17.853 | 2.72E-71 | *9.94E-69* |
| **PPP1R12C** | ENSG00000125503 | -1.16 | 0.11664 | -9.9057 | 3.93E-23 | *9.02E-22* |
| **PRELP** | ENSG00000188783 | -4.74 | 0.35981 | -13.176 | 1.21E-39 | *9.09E-38* |
| **PRG4** | ENSG00000116690 | -1.65 | 0.29256 | -5.6399 | 1.70E-08 | *8.76E-08* |
| **PRKACB** | ENSG00000142875 | -1.31 | 0.1735 | -7.5687 | 3.77E-14 | *3.70E-13* |
| **PRKCA** | ENSG00000154229 | -1.81 | 0.2366 | -7.6656 | 1.78E-14 | *1.81E-13* |
| **PRKCB** | ENSG00000166501 | -1.84 | 0.30405 | -6.0509 | 1.44E-09 | *8.45E-09* |
| **PRL** | ENSG00000172179 | -1.52 | 0.46097 | -3.2883 | 1.01E-03 | *2.54E-03* |
| **PRNP** | ENSG00000171867 | -2.33 | 0.26371 | -8.8371 | 9.83E-19 | *1.50E-17* |
| **PRSS1** | ENSG00000204983 | 3.87 | 0.67275 | 5.75151 | 8.84E-09 | *4.72E-08* |
| **PRSS2** | ENSG00000275896 | 5.12 | 0.49649 | 10.3224 | 5.58E-25 | *1.47E-23* |
| **PRSS3** | ENSG00000010438 | 2.75 | 0.49682 | 5.53892 | 3.04E-08 | *1.53E-07* |
| **PTH** | ENSG00000152266 | 2.70 | 0.65669 | 4.11271 | 3.91E-05 | *1.26E-04* |
| **PTN** | ENSG00000105894 | -1.15 | 0.38759 | -2.96 | 3.08E-03 | *7.05E-03* |
| **PTPN11** | ENSG00000179295 | -0.70 | 0.13549 | -5.1604 | 2.46E-07 | *1.10E-06* |
| **PTPN6** | ENSG00000111679 | 1.71 | 0.14878 | 11.4928 | 1.43E-30 | *5.84E-29* |
| **PTPRB** | ENSG00000127329 | -2.19 | 0.22686 | -9.6572 | 4.58E-22 | *9.43E-21* |
| **PTPRF** | ENSG00000142949 | 0.60 | 0.1701 | 3.54221 | 3.97E-04 | *1.08E-03* |
| **PTPRT** | ENSG00000196090 | -2.71 | 0.67385 | -4.0268 | 5.65E-05 | *1.77E-04* |
| **PXN** | ENSG00000089159 | -0.66 | 0.11213 | -5.8963 | 3.72E-09 | *2.07E-08* |
| **PZP** | ENSG00000126838 | 1.63 | 0.53411 | 3.0484 | 2.30E-03 | *5.41E-03* |
| **RDX** | ENSG00000137710 | -0.61 | 0.15188 | -4.0142 | 5.97E-05 | *1.87E-04* |
| **REG4** | ENSG00000134193 | 3.37 | 0.62159 | 5.42866 | 5.68E-08 | *2.76E-07* |
| **RELN** | ENSG00000189056 | -1.98 | 0.55997 | -3.5325 | 4.12E-04 | *1.11E-03* |
| **ROCK1** | ENSG00000067900 | -1.07 | 0.14209 | -7.5065 | 6.07E-14 | *5.85E-13* |
| **ROCK2** | ENSG00000134318 | -1.38 | 0.1273 | -10.835 | 2.35E-27 | *7.56E-26* |
| **RPS6KB2** | ENSG00000175634 | 1.08 | 0.11949 | 9.04121 | 1.55E-19 | *2.57E-18* |
| **RPTN** | ENSG00000215853 | 7.30 | 1.16025 | 6.29004 | 3.17E-10 | *2.01E-09* |
| **RRAS** | ENSG00000126458 | -1.54 | 0.20209 | -7.6197 | 2.54E-14 | *2.54E-13* |
| **RSPO1** | ENSG00000169218 | -5.06 | 0.48686 | -10.383 | 2.95E-25 | *7.90E-24* |
| **RSPO2** | ENSG00000147655 | 2.15 | 0.58218 | 3.69342 | 2.21E-04 | *6.29E-04* |
| **RSPO3** | ENSG00000146374 | -3.89 | 0.47899 | -8.1307 | 4.27E-16 | *5.04E-15* |
| **RSPO4** | ENSG00000101282 | 1.73 | 0.59657 | 2.90136 | 3.72E-03 | *8.38E-03* |
| **RTN4** | ENSG00000115310 | -0.83 | 0.11167 | -7.4737 | 7.80E-14 | *7.44E-13* |
| **RUNX1** | ENSG00000159216 | 1.53 | 0.19957 | 7.67872 | 1.61E-14 | *1.64E-13* |
| **RUNX2** | ENSG00000124813 | 1.45 | 0.3332 | 4.34761 | 1.38E-05 | *4.74E-05* |
| **S100A11** | ENSG00000163191 | 2.38 | 0.19926 | 11.9238 | 8.90E-33 | *4.19E-31* |
| **S100A14** | ENSG00000189334 | 1.84 | 0.40117 | 4.57496 | 4.76E-06 | *1.76E-05* |
| **S100A16** | ENSG00000188643 | 0.87 | 0.28101 | 3.1063 | 1.89E-03 | *4.53E-03* |
| **S100A2** | ENSG00000196754 | 4.50 | 0.39751 | 11.323 | 1.01E-29 | *3.85E-28* |
| **S100A3** | ENSG00000188015 | 2.23 | 0.41484 | 5.37087 | 7.84E-08 | *3.73E-07* |
| **S100A7** | ENSG00000143556 | 5.31 | 0.75668 | 7.01552 | 2.29E-12 | *1.86E-11* |
| **S100A7A** | ENSG00000184330 | 4.30 | 1.07208 | 4.01441 | 5.96E-05 | *1.86E-04* |
| **S100A8** | ENSG00000143546 | 2.90 | 0.47775 | 6.07021 | 1.28E-09 | *7.53E-09* |
| **S100A9** | ENSG00000163220 | 3.27 | 0.45774 | 7.14886 | 8.75E-13 | *7.43E-12* |
| **S100G** | ENSG00000169906 | 3.54 | 0.83465 | 4.24234 | 2.21E-05 | *7.40E-05* |
| **S100P** | ENSG00000163993 | 4.42 | 0.46264 | 9.55495 | 1.24E-21 | *2.44E-20* |
| **SCG2** | ENSG00000171951 | -1.13 | 0.37891 | -2.9904 | 2.79E-03 | *6.45E-03* |
| **SCUBE3** | ENSG00000146197 | 1.48 | 0.39583 | 3.72787 | 1.93E-04 | *5.55E-04* |
| **SCX** | ENSG00000260428 | 2.56 | 0.35181 | 7.26757 | 3.66E-13 | *3.23E-12* |
| **SDC1** | ENSG00000115884 | 3.82 | 0.25265 | 15.1391 | 8.94E-52 | *1.32E-49* |
| **SDC2** | ENSG00000169439 | -0.95 | 0.23655 | -3.9993 | 6.35E-05 | *1.98E-04* |
| **SDC3** | ENSG00000162512 | -0.64 | 0.18679 | -3.4134 | 6.41E-04 | *1.67E-03* |
| **SELE** | ENSG00000007908 | -5.15 | 0.38513 | -13.376 | 8.40E-41 | *6.92E-39* |
| **SELP** | ENSG00000174175 | -3.38 | 0.31781 | -10.637 | 2.00E-26 | *5.96E-25* |
| **SEMA3A** | ENSG00000075213 | 1.24 | 0.3884 | 3.20191 | 1.37E-03 | *3.35E-03* |
| **SEMA3E** | ENSG00000170381 | 2.18 | 0.4925 | 4.4207 | 9.84E-06 | *3.47E-05* |
| **SEMA3F** | ENSG00000001617 | 0.98 | 0.20968 | 4.66031 | 3.16E-06 | *1.20E-05* |
| **SEMA3G** | ENSG00000010319 | -1.02 | 0.26898 | -3.7899 | 1.51E-04 | *4.41E-04* |
| **SEMA4A** | ENSG00000196189 | 1.41 | 0.19454 | 7.24637 | 4.28E-13 | *3.75E-12* |
| **SEMA4B** | ENSG00000185033 | 1.26 | 0.20402 | 6.15938 | 7.30E-10 | *4.44E-09* |
| **SEMA4D** | ENSG00000187764 | 0.88 | 0.11419 | 7.68636 | 1.51E-14 | *1.55E-13* |
| **SEMA5B** | ENSG00000082684 | 1.34 | 0.40869 | 3.2773 | 1.05E-03 | *2.63E-03* |
| **SEMA6B** | ENSG00000167680 | -1.05 | 0.23702 | -4.4136 | 1.02E-05 | *3.58E-05* |
| **SEMA6D** | ENSG00000137872 | -2.26 | 0.32379 | -6.988 | 2.79E-12 | *2.24E-11* |
| **SEMA7A** | ENSG00000138623 | 0.70 | 0.21351 | 3.26495 | 1.09E-03 | *2.74E-03* |
| **SERPINA1** | ENSG00000197249 | 3.01 | 0.4067 | 7.41106 | 1.25E-13 | *1.17E-12* |
| **SERPINA10** | ENSG00000140093 | -1.65 | 0.57813 | -2.8571 | 4.27E-03 | *9.52E-03* |
| **SERPINA11** | ENSG00000186910 | 4.81 | 0.7429 | 6.47357 | 9.57E-11 | *6.47E-10* |
| **SERPINA4** | ENSG00000100665 | 2.70 | 0.54811 | 4.92227 | 8.55E-07 | *3.52E-06* |
| **SERPINA5** | ENSG00000188488 | 1.43 | 0.47197 | 3.02921 | 2.45E-03 | *5.74E-03* |
| **SERPINB11** | ENSG00000206072 | 3.14 | 0.9919 | 3.1618 | 1.57E-03 | *3.81E-03* |
| **SERPINB13** | ENSG00000197641 | 2.97 | 0.74845 | 3.96696 | 7.28E-05 | *2.25E-04* |
| **SERPINB2** | ENSG00000197632 | 2.36 | 0.63724 | 3.7105 | 2.07E-04 | *5.91E-04* |
| **SERPINB3** | ENSG00000057149 | 3.07 | 0.72574 | 4.2323 | 2.31E-05 | *7.72E-05* |
| **SERPINB5** | ENSG00000206075 | 3.39 | 0.46105 | 7.35475 | 1.91E-13 | *1.74E-12* |
| **SERPINB7** | ENSG00000166396 | 2.32 | 0.67612 | 3.42607 | 6.12E-04 | *1.60E-03* |
| **SERPINC1** | ENSG00000117601 | 1.22 | 0.36186 | 3.36048 | 7.78E-04 | *2.00E-03* |
| **SERPINE1** | ENSG00000106366 | -1.31 | 0.30585 | -4.2695 | 1.96E-05 | *6.61E-05* |
| **SERPINF1** | ENSG00000132386 | -2.21 | 0.27754 | -7.9481 | 1.89E-15 | *2.12E-14* |
| **SERPING1** | ENSG00000149131 | -1.47 | 0.24052 | -6.1213 | 9.28E-10 | *5.55E-09* |
| **SERPINH1** | ENSG00000149257 | 0.79 | 0.17767 | 4.42102 | 9.82E-06 | *3.46E-05* |
| **SFRP1** | ENSG00000104332 | -2.73 | 0.34558 | -7.9078 | 2.62E-15 | *2.90E-14* |
| **SFRP4** | ENSG00000106483 | -2.05 | 0.50854 | -4.0391 | 5.37E-05 | *1.69E-04* |
| **SFTA2** | ENSG00000196260 | 4.62 | 0.52631 | 8.77969 | 1.64E-18 | *2.45E-17* |
| **SFTPA2** | ENSG00000185303 | 2.68 | 0.51126 | 5.24645 | 1.55E-07 | *7.10E-07* |
| **SFTPB** | ENSG00000168878 | 2.76 | 0.59299 | 4.65236 | 3.28E-06 | *1.24E-05* |
| **SGCA** | ENSG00000108823 | -4.59 | 0.39556 | -11.615 | 3.45E-31 | *1.47E-29* |
| **SGSH** | ENSG00000181523 | 0.64 | 0.14608 | 4.39881 | 1.09E-05 | *3.81E-05* |
| **SHH** | ENSG00000164690 | 3.33 | 0.44246 | 7.52973 | 5.08E-14 | *4.93E-13* |
| **SLC26A2** | ENSG00000155850 | 1.78 | 0.28098 | 6.32882 | 2.47E-10 | *1.59E-09* |
| **SLC35B2** | ENSG00000157593 | 0.75 | 0.11647 | 6.43767 | 1.21E-10 | *8.10E-10* |
| **SLC9A1** | ENSG00000090020 | 0.62 | 0.13937 | 4.46229 | 8.11E-06 | *2.89E-05* |
| **SLIT1** | ENSG00000187122 | 1.25 | 0.40463 | 3.0937 | 1.98E-03 | *4.71E-03* |
| **SLIT2** | ENSG00000145147 | -1.48 | 0.33665 | -4.4005 | 1.08E-05 | *3.78E-05* |
| **SLIT3** | ENSG00000184347 | -2.57 | 0.27366 | -9.3759 | 6.86E-21 | *1.27E-19* |
| **SLPI** | ENSG00000124107 | 2.28 | 0.399 | 5.72154 | 1.06E-08 | *5.59E-08* |
| **SMAD1** | ENSG00000170365 | -0.95 | 0.19202 | -4.9552 | 7.22E-07 | *3.00E-06* |
| **SMAD3** | ENSG00000166949 | -1.19 | 0.1495 | -7.9478 | 1.90E-15 | *2.12E-14* |
| **SMAD5** | ENSG00000113658 | -0.98 | 0.13196 | -7.4621 | 8.51E-14 | *8.10E-13* |
| **SMOC2** | ENSG00000112562 | -3.12 | 0.28333 | -11.027 | 2.84E-28 | *9.72E-27* |
| **SMTN** | ENSG00000183963 | -2.60 | 0.19567 | -13.284 | 2.86E-40 | *2.25E-38* |
| **SNED1** | ENSG00000162804 | -1.90 | 0.26421 | -7.1753 | 7.22E-13 | *6.17E-12* |
| **SOD3** | ENSG00000109610 | -1.14 | 0.32839 | -3.4745 | 5.12E-04 | *1.36E-03* |
| **SOS2** | ENSG00000100485 | -0.92 | 0.11586 | -7.9125 | 2.52E-15 | *2.80E-14* |
| **SOX9** | ENSG00000125398 | 0.99 | 0.3074 | 3.23224 | 1.23E-03 | *3.04E-03* |
| **SP3** | ENSG00000172845 | -0.62 | 0.13521 | -4.6066 | 4.09E-06 | *1.53E-05* |
| **SPARC** | ENSG00000113140 | -1.23 | 0.21511 | -5.7141 | 1.10E-08 | *5.82E-08* |
| **SPARCL1** | ENSG00000152583 | -4.33 | 0.27804 | -15.578 | 1.03E-54 | *1.77E-52* |
| **SPOCK3** | ENSG00000196104 | -2.12 | 0.62863 | -3.3742 | 7.40E-04 | *1.91E-03* |
| **SPP1** | ENSG00000118785 | 3.46 | 0.36435 | 9.49583 | 2.18E-21 | *4.24E-20* |
| **SRF** | ENSG00000112658 | -1.22 | 0.12873 | -9.4434 | 3.61E-21 | *6.88E-20* |
| **SRGN** | ENSG00000122862 | -0.86 | 0.24277 | -3.5456 | 3.92E-04 | *1.06E-03* |
| **SRPX** | ENSG00000101955 | -3.80 | 0.34212 | -11.099 | 1.27E-28 | *4.42E-27* |
| **SST** | ENSG00000157005 | 3.72 | 0.65399 | 5.68387 | 1.32E-08 | *6.88E-08* |
| **ST14** | ENSG00000149418 | 1.87 | 0.2082 | 8.96788 | 3.02E-19 | *4.87E-18* |
| **SULF2** | ENSG00000196562 | -1.21 | 0.27652 | -4.3811 | 1.18E-05 | *4.11E-05* |
| **SVEP1** | ENSG00000165124 | -2.67 | 0.28478 | -9.3906 | 5.97E-21 | *1.12E-19* |
| **SYT1** | ENSG00000067715 | -3.47 | 0.42686 | -8.1392 | 3.98E-16 | *4.72E-15* |
| **TCF7L2** | ENSG00000148737 | -1.00 | 0.18775 | -5.3432 | 9.13E-08 | *4.31E-07* |
| **TCHH** | ENSG00000159450 | 2.26 | 0.39637 | 5.69219 | 1.25E-08 | *6.57E-08* |
| **TCHHL1** | ENSG00000182898 | 4.39 | 1.10378 | 3.97485 | 7.04E-05 | *2.18E-04* |
| **TEK** | ENSG00000120156 | -2.47 | 0.25896 | -9.5344 | 1.51E-21 | *2.96E-20* |
| **TGFA** | ENSG00000163235 | 1.75 | 0.29445 | 5.93806 | 2.88E-09 | *1.63E-08* |
| **TGFB2** | ENSG00000092969 | -1.13 | 0.34915 | -3.2427 | 1.18E-03 | *2.94E-03* |
| **TGFB3** | ENSG00000119699 | -1.47 | 0.22519 | -6.5109 | 7.47E-11 | *5.12E-10* |
| **TGFBI** | ENSG00000120708 | 1.06 | 0.34439 | 3.07011 | 2.14E-03 | *5.06E-03* |
| **TGFBR1** | ENSG00000106799 | -0.90 | 0.11727 | -7.7111 | 1.25E-14 | *1.28E-13* |
| **TGFBR3** | ENSG00000069702 | -3.42 | 0.29535 | -11.577 | 5.36E-31 | *2.24E-29* |
| **TGIF1** | ENSG00000177426 | 0.62 | 0.12807 | 4.80367 | 1.56E-06 | *6.18E-06* |
| **TGM1** | ENSG00000092295 | -1.48 | 0.30937 | -4.7751 | 1.80E-06 | *7.07E-06* |
| **TGM2** | ENSG00000198959 | -1.86 | 0.26614 | -6.9846 | 2.86E-12 | *2.30E-11* |
| **TGM3** | ENSG00000125780 | 1.34 | 0.43633 | 3.06081 | 2.21E-03 | *5.21E-03* |
| **TGM5** | ENSG00000104055 | 4.37 | 0.44956 | 9.72997 | 2.25E-22 | *4.80E-21* |
| **TGM7** | ENSG00000159495 | 5.66 | 0.57089 | 9.92133 | 3.36E-23 | *7.75E-22* |
| **THBD** | ENSG00000178726 | -2.52 | 0.29171 | -8.6534 | 5.00E-18 | *7.13E-17* |
| **THBS1** | ENSG00000137801 | -2.78 | 0.28937 | -9.6118 | 7.13E-22 | *1.44E-20* |
| **THBS2** | ENSG00000186340 | -2.14 | 0.32841 | -6.5156 | 7.24E-11 | *4.98E-10* |
| **THBS4** | ENSG00000113296 | -2.19 | 0.40527 | -5.3989 | 6.71E-08 | *3.23E-07* |
| **TIAM1** | ENSG00000156299 | -1.57 | 0.22541 | -6.9834 | 2.88E-12 | *2.31E-11* |
| **TIMP2** | ENSG00000035862 | -2.60 | 0.22359 | -11.626 | 3.04E-31 | *1.30E-29* |
| **TIMP3** | ENSG00000100234 | -3.15 | 0.40519 | -7.7728 | 7.68E-15 | *8.06E-14* |
| **TIMP4** | ENSG00000157150 | -1.97 | 0.50662 | -3.8891 | 1.01E-04 | *3.03E-04* |
| **TINAG** | ENSG00000137251 | 3.60 | 1.00002 | 3.59593 | 3.23E-04 | *8.91E-04* |
| **TLL1** | ENSG00000038295 | -2.59 | 0.36711 | -7.0459 | 1.84E-12 | *1.51E-11* |
| **TLL2** | ENSG00000095587 | 1.44 | 0.34955 | 4.11548 | 3.86E-05 | *1.24E-04* |
| **TLR2** | ENSG00000137462 | 0.77 | 0.20352 | 3.78146 | 1.56E-04 | *4.56E-04* |
| **TLR4** | ENSG00000136869 | -1.00 | 0.29836 | -3.3532 | 7.99E-04 | *2.05E-03* |
| **TMEM2** | ENSG00000135048 | 0.82 | 0.21528 | 3.81347 | 1.37E-04 | *4.04E-04* |
| **TMPRSS15** | ENSG00000154646 | 2.33 | 0.77237 | 3.01936 | 2.53E-03 | *5.91E-03* |
| **TNF** | ENSG00000232810 | 2.55 | 0.37337 | 6.83562 | 8.17E-12 | *6.25E-11* |
| **TNFSF11** | ENSG00000120659 | 4.88 | 0.4882 | 10.0019 | 1.49E-23 | *3.55E-22* |
| **TNFSF12** | ENSG00000239697 | -1.39 | 0.19303 | -7.226 | 4.97E-13 | *4.32E-12* |
| **TNFSF14** | ENSG00000125735 | 1.29 | 0.39588 | 3.25069 | 1.15E-03 | *2.87E-03* |
| **TNFSF15** | ENSG00000181634 | 1.14 | 0.25943 | 4.40541 | 1.06E-05 | *3.71E-05* |
| **TNFSF18** | ENSG00000120337 | 1.93 | 0.43912 | 4.38448 | 1.16E-05 | *4.05E-05* |
| **TNFSF9** | ENSG00000125657 | 1.20 | 0.36412 | 3.28254 | 1.03E-03 | *2.59E-03* |
| **TNR** | ENSG00000116147 | 1.62 | 0.51723 | 3.12969 | 1.75E-03 | *4.21E-03* |
| **TNXB** | ENSG00000168477 | -4.66 | 0.40405 | -11.541 | 8.22E-31 | *3.39E-29* |
| **TPH1** | ENSG00000129167 | 1.79 | 0.53467 | 3.34557 | 8.21E-04 | *2.11E-03* |
| **TPO** | ENSG00000115705 | -2.49 | 0.50017 | -4.9781 | 6.42E-07 | *2.69E-06* |
| **TPSG1** | ENSG00000116176 | -3.71 | 0.32391 | -11.461 | 2.08E-30 | *8.27E-29* |
| **TRIB3** | ENSG00000101255 | 2.19 | 0.30264 | 7.25166 | 4.12E-13 | *3.61E-12* |
| **TSKU** | ENSG00000182704 | 1.11 | 0.23311 | 4.74418 | 2.09E-06 | *8.15E-06* |
| **TTF2** | ENSG00000116830 | 1.07 | 0.14266 | 7.5247 | 5.28E-14 | *5.12E-13* |
| **TWIST2** | ENSG00000233608 | -1.35 | 0.39615 | -3.4174 | 6.32E-04 | *1.65E-03* |
| **UGDH** | ENSG00000109814 | 0.95 | 0.1872 | 5.07202 | 3.94E-07 | *1.70E-06* |
| **VANGL2** | ENSG00000162738 | 1.39 | 0.20106 | 6.92592 | 4.33E-12 | *3.41E-11* |
| **VEGFC** | ENSG00000150630 | -1.13 | 0.21984 | -5.1557 | 2.53E-07 | *1.12E-06* |
| **VIP** | ENSG00000146469 | -3.05 | 0.33977 | -8.9744 | 2.85E-19 | *4.61E-18* |
| **VIT** | ENSG00000205221 | -4.56 | 0.46073 | -9.9012 | 4.11E-23 | *9.42E-22* |
| **VTN** | ENSG00000109072 | -2.91 | 0.35663 | -8.1461 | 3.76E-16 | *4.47E-15* |
| **VWA1** | ENSG00000179403 | 0.93 | 0.21449 | 4.32442 | 1.53E-05 | *5.23E-05* |
| **VWA3B** | ENSG00000168658 | -1.52 | 0.4382 | -3.479 | 5.03E-04 | *1.34E-03* |
| **VWA5B2** | ENSG00000145198 | 2.51 | 0.47723 | 5.25181 | 1.51E-07 | *6.90E-07* |
| **VWA7** | ENSG00000204396 | -2.27 | 0.31226 | -7.2728 | 3.52E-13 | *3.12E-12* |
| **VWC2** | ENSG00000188730 | -4.92 | 0.50992 | -9.6552 | 4.67E-22 | *9.60E-21* |
| **VWF** | ENSG00000110799 | -1.36 | 0.24384 | -5.5841 | 2.35E-08 | *1.19E-07* |
| **WFIKKN1** | ENSG00000127578 | 0.95 | 0.30664 | 3.10157 | 1.92E-03 | *4.60E-03* |
| **WFIKKN2** | ENSG00000173714 | -2.37 | 0.4012 | -5.912 | 3.38E-09 | *1.90E-08* |
| **WIF1** | ENSG00000156076 | 2.75 | 0.63347 | 4.341 | 1.42E-05 | *4.87E-05* |
| **WISP1** | ENSG00000104415 | -2.20 | 0.27165 | -8.0875 | 6.09E-16 | *7.10E-15* |
| **WISP2** | ENSG00000064205 | -5.41 | 0.37819 | -14.314 | 1.78E-46 | *2.07E-44* |
| **WNT10B** | ENSG00000169884 | 1.09 | 0.36185 | 3.02481 | 2.49E-03 | *5.81E-03* |
| **WNT11** | ENSG00000085741 | 2.20 | 0.43655 | 5.0371 | 4.73E-07 | *2.02E-06* |
| **WNT16** | ENSG00000002745 | 1.60 | 0.49854 | 3.2166 | 1.30E-03 | *3.20E-03* |
| **WNT2B** | ENSG00000134245 | -2.59 | 0.27273 | -9.4826 | 2.48E-21 | *4.78E-20* |
| **WNT3A** | ENSG00000154342 | 1.75 | 0.43774 | 4.00428 | 6.22E-05 | *1.94E-04* |
| **WNT4** | ENSG00000162552 | -1.44 | 0.35964 | -3.9999 | 6.34E-05 | *1.97E-04* |
| **WNT7A** | ENSG00000154764 | 3.64 | 0.51967 | 7.01332 | 2.33E-12 | *1.89E-11* |
| **WNT9B** | ENSG00000158955 | -3.38 | 0.47256 | -7.1543 | 8.41E-13 | *7.16E-12* |
| **XCL1** | ENSG00000143184 | 1.69 | 0.39904 | 4.22753 | 2.36E-05 | *7.86E-05* |
| **XDH** | ENSG00000158125 | 4.09 | 0.47941 | 8.52754 | 1.49E-17 | *2.05E-16* |
| **XYLT1** | ENSG00000103489 | -2.18 | 0.30588 | -7.1289 | 1.01E-12 | *8.53E-12* |
| **ZP3** | ENSG00000188372 | 0.78 | 0.26496 | 2.95623 | 3.11E-03 | *7.13E-03* |

log2FoldChange, log2 fold change between the groups

lfcSE, standard error of the log2FoldChange estimate

stat, Wald statistics

p value, Wald test p-value

padj, Benjamini-Hochberg adjusted p-value
