# Supplementary material for: Computational analysis for identification of the extracellular matrix molecules involved in endometrial cancer progression
Source: PLoS One. 2020 Apr 21;15(4):e0231594. doi: 10.1371/journal.pone.0231594 (PMC7173926; doi:10.1371/journal.pone.0231594)
Supplement: S3 Table — (DOCX) [file pone.0231594.s004.docx]

| **S3 Table. Metastasis PAN-ECM DEGs (241)** | | | | | | |
| --- | --- | --- | --- | --- | --- | --- |
| **Gene Symbol** | **Ensembl_gene_id** | **log2FoldChange** | **lfcSE** | **stat** | **pvalue** | **padj** |
| **AMELX** | ENSG00000125363 | 1.28 | 0.402210852 | 3.183236075 | 1.46E-03 | *8.12E-03* |
| **COL10A1** | ENSG00000123500 | 1.42 | 0.224699344 | 6.321712908 | 2.59E-10 | *2.04E-08* |
| **COL11A1** | ENSG00000060718 | 0.99 | 0.214389175 | 4.601640074 | 4.19E-06 | *7.13E-05* |
| **COL11A2** | ENSG00000204248 | 0.98 | 0.156610672 | 6.286209739 | 3.25E-10 | *2.51E-08* |
| **COL19A1** | ENSG00000082293 | 0.62 | 0.217432273 | 2.839706979 | 4.52E-03 | *9.00E-03* |
| **COL20A1** | ENSG00000101203 | 1.51 | 0.238592334 | 6.34902734 | 2.17E-10 | *1.77E-08* |
| **COL22A1** | ENSG00000169436 | 0.88 | 0.217128316 | 4.053094843 | 5.05E-05 | *5.47E-04* |
| **COL23A1** | ENSG00000050767 | 0.82 | 0.188635049 | 4.370222209 | 1.24E-05 | *1.76E-04* |
| **COL24A1** | ENSG00000171502 | 0.75 | 0.155511898 | 4.845089243 | 1.27E-06 | *2.62E-05* |
| **COL26A1** | ENSG00000160963 | 0.88 | 0.204858152 | 4.315467081 | 1.59E-05 | *2.15E-04* |
| **COL28A1** | ENSG00000215018 | -0.59 | 0.158693823 | -3.739711194 | 1.84E-04 | *1.55E-03* |
| **COL4A3** | ENSG00000169031 | 2.07 | 0.222967919 | 9.303587363 | 1.36E-20 | *1.96E-17* |
| **COL4A4** | ENSG00000081052 | 1.67 | 0.191277632 | 8.705328027 | 3.17E-18 | *2.23E-15* |
| **COL6A3** | ENSG00000163359 | 0.80 | 0.130907883 | 6.142637956 | 8.12E-10 | *5.54E-08* |
| **COL6A5** | ENSG00000172752 | 0.99 | 0.251615145 | 3.918809858 | 8.90E-05 | *8.62E-04* |
| **COL6A6** | ENSG00000206384 | 1.26 | 0.204576173 | 6.162764393 | 7.15E-10 | *5.01E-08* |
| **COL8A1** | ENSG00000144810 | 0.99 | 0.1544445 | 6.399957926 | 1.55E-10 | *1.35E-08* |
| **COL8A2** | ENSG00000171812 | 0.91 | 0.158607461 | 5.757456499 | 8.54E-09 | *4.15E-07* |
| **COL9A1** | ENSG00000112280 | 1.90 | 0.269753292 | 7.02807478 | 2.09E-12 | *2.88E-10* |
| **COMP** | ENSG00000105664 | 2.00 | 0.237049906 | 8.456010606 | 2.77E-17 | *1.45E-14* |
| **DMBT1** | ENSG00000187908 | -1.43 | 0.252369151 | -5.683510123 | 1.32E-08 | *5.94E-07* |
| **DPT** | ENSG00000143196 | -1.04 | 0.20010657 | -5.190825987 | 2.09E-07 | *6.00E-06* |
| **ELN** | ENSG00000049540 | 1.26 | 0.172314589 | 7.294648987 | 2.99E-13 | *5.15E-11* |
| **ELSPBP1** | ENSG00000169393 | 2.20 | 0.461487644 | 4.758040394 | 1.95E-06 | *3.79E-05* |
| **EYS** | ENSG00000188107 | 0.67 | 0.113304862 | 5.894931604 | 3.75E-09 | *2.07E-07* |
| **FBLN1** | ENSG00000077942 | -0.68 | 0.169888631 | -4.001353985 | 6.30E-05 | *6.50E-04* |
| **FBN3** | ENSG00000142449 | 1.00 | 0.224773999 | 4.432018872 | 9.34E-06 | *1.38E-04* |
| **FN1** | ENSG00000115414 | 0.79 | 0.15309466 | 5.142104872 | 2.72E-07 | *7.43E-06* |
| **IGFALS** | ENSG00000099769 | 1.09 | 0.178048706 | 6.147692582 | 7.86E-10 | *5.40E-08* |
| **IGFBP1** | ENSG00000146678 | 1.70 | 0.26583977 | 6.400759786 | 1.55E-10 | *1.34E-08* |
| **IGSF10** | ENSG00000152580 | 0.69 | 0.173776421 | 3.970756429 | 7.16E-05 | *7.19E-04* |
| **IMPG1** | ENSG00000112706 | 0.73 | 0.151634761 | 4.782555678 | 1.73E-06 | *3.43E-05* |
| **IMPG2** | ENSG00000081148 | 0.66 | 0.154526996 | 4.269258713 | 1.96E-05 | *2.55E-04* |
| **KCP** | ENSG00000135253 | 0.70 | 0.179784598 | 3.869973889 | 1.09E-04 | *1.02E-03* |
| **LAMA2** | ENSG00000196569 | 0.68 | 0.149935811 | 4.503673338 | 6.68E-06 | *1.05E-04* |
| **LGI2** | ENSG00000153012 | 1.07 | 0.157596364 | 6.783970575 | 1.17E-11 | *1.35E-09* |
| **MATN1** | ENSG00000162510 | -0.65 | 0.152464129 | -4.287815724 | 1.80E-05 | *2.38E-04* |
| **MATN2** | ENSG00000132561 | -0.68 | 0.135773562 | -5.033086419 | 4.83E-07 | *1.20E-05* |
| **MATN4** | ENSG00000124159 | 0.62 | 0.179090528 | 3.464266506 | 5.32E-04 | *3.62E-03* |
| **NELL2** | ENSG00000184613 | 0.92 | 0.193327443 | 4.78081612 | 1.75E-06 | *3.45E-05* |
| **NTN3** | ENSG00000162068 | 0.88 | 0.18226152 | 4.836999953 | 1.32E-06 | *2.72E-05* |
| **NTNG1** | ENSG00000162631 | 0.87 | 0.230848425 | 3.774466688 | 1.60E-04 | *1.39E-03* |
| **NYX** | ENSG00000188937 | 0.93 | 0.261351459 | 3.569321503 | 3.58E-04 | *2.63E-03* |
| **OGN** | ENSG00000106809 | -0.98 | 0.26075294 | -3.766638512 | 1.65E-04 | *1.42E-03* |
| **PAPLN** | ENSG00000100767 | 0.85 | 0.132680973 | 6.411617799 | 1.44E-10 | *1.26E-08* |
| **PCOLCE2** | ENSG00000163710 | 0.83 | 0.195748259 | 4.215742622 | 2.49E-05 | *3.09E-04* |
| **POSTN** | ENSG00000133110 | 0.60 | 0.171709186 | 3.494489147 | 4.75E-04 | *3.31E-03* |
| **PRG2** | ENSG00000186652 | 0.91 | 0.250591671 | 3.645887426 | 2.66E-04 | *2.07E-03* |
| **PXDN** | ENSG00000130508 | 0.82 | 0.157727556 | 5.212381268 | 1.86E-07 | *5.44E-06* |
| **RELN** | ENSG00000189056 | 1.13 | 0.249764621 | 4.528862684 | 5.93E-06 | *9.53E-05* |
| **RSPO4** | ENSG00000101282 | 1.28 | 0.263005627 | 4.871784128 | 1.11E-06 | *2.34E-05* |
| **SLIT1** | ENSG00000187122 | 0.59 | 0.184649414 | 3.192077292 | 1.41E-03 | *7.91E-03* |
| **SRPX** | ENSG00000101955 | 0.82 | 0.156272121 | 5.251501391 | 1.51E-07 | *4.55E-06* |
| **THBS2** | ENSG00000186340 | 0.65 | 0.149827823 | 4.360882494 | 1.30E-05 | *1.82E-04* |
| **TNFAIP6** | ENSG00000123610 | 0.81 | 0.154755795 | 5.265105757 | 1.40E-07 | *4.32E-06* |
| **TSPEAR** | ENSG00000175894 | 1.73 | 0.223633759 | 7.730589999 | 1.07E-14 | *2.79E-12* |
| **VCAN** | ENSG00000038427 | 0.74 | 0.148098288 | 5.022445391 | 5.10E-07 | *1.25E-05* |
| **VWA2** | ENSG00000165816 | -0.65 | 0.180099027 | -3.625227844 | 2.89E-04 | *2.21E-03* |
| **VWA3A** | ENSG00000175267 | -1.00 | 0.218002587 | -4.579501657 | 4.66E-06 | *7.81E-05* |
| **VWA3B** | ENSG00000168658 | -0.96 | 0.195237483 | -4.908821533 | 9.16E-07 | *2.02E-05* |
| **ZPLD1** | ENSG00000170044 | 1.25 | 0.238710075 | 5.216472061 | 1.82E-07 | *5.35E-06* |
| **ADAM18** | ENSG00000168619 | 1.57 | 0.351140203 | 4.467778521 | 7.90E-06 | *1.20E-04* |
| **ADAM28** | ENSG00000042980 | -0.72 | 0.142516261 | -5.03682564 | 4.73E-07 | *1.18E-05* |
| **ADAM33** | ENSG00000149451 | 1.05 | 0.191442664 | 5.463102083 | 4.68E-08 | *1.72E-06* |
| **ADAMTS1** | ENSG00000154734 | 0.61 | 0.124036681 | 4.930771817 | 8.19E-07 | *1.84E-05* |
| **ADAMTS16** | ENSG00000145536 | 1.65 | 0.21587149 | 7.631088215 | 2.33E-14 | *5.56E-12* |
| **ADAMTS19** | ENSG00000145808 | -0.70 | 0.215519951 | -3.234146014 | 1.22E-03 | *7.04E-03* |
| **ADAMTS3** | ENSG00000156140 | 1.41 | 0.142980944 | 9.847917293 | 7.00E-23 | *2.25E-19* |
| **ADAMTS6** | ENSG00000049192 | -1.09 | 0.187773319 | -5.80376503 | 6.48E-09 | *3.27E-07* |
| **ADAMTS8** | ENSG00000134917 | -1.47 | 0.213214727 | -6.873134707 | 6.28E-12 | *7.86E-10* |
| **ADAMTS9** | ENSG00000163638 | 0.60 | 0.118281805 | 5.063742004 | 4.11E-07 | *1.05E-05* |
| **ADAMTSL2** | ENSG00000197859 | -0.70 | 0.180333181 | -3.889043115 | 1.01E-04 | *9.50E-04* |
| **ADAMTSL3** | ENSG00000156218 | -0.69 | 0.18767613 | -3.669975334 | 2.43E-04 | *1.92E-03* |
| **ADAMTSL4** | ENSG00000143382 | 0.59 | 0.123242113 | 4.756639396 | 1.97E-06 | *3.81E-05* |
| **ANGPT4** | ENSG00000101280 | 1.06 | 0.234578308 | 4.523940571 | 6.07E-06 | *9.72E-05* |
| **ANGPTL3** | ENSG00000132855 | 0.84 | 0.180398985 | 4.663463797 | 3.11E-06 | *5.55E-05* |
| **ANGPTL7** | ENSG00000171819 | 1.30 | 0.222349773 | 5.86868162 | 4.39E-09 | *2.35E-07* |
| **BMP3** | ENSG00000152785 | 1.26 | 0.214249988 | 5.859198359 | 4.65E-09 | *2.46E-07* |
| **BMP7** | ENSG00000101144 | 0.94 | 0.201647059 | 4.657014648 | 3.21E-06 | *5.70E-05* |
| **C1QL1** | ENSG00000131094 | 1.27 | 0.228108498 | 5.569045977 | 2.56E-08 | *1.03E-06* |
| **C1QL2** | ENSG00000144119 | 1.04 | 0.312031602 | 3.321575408 | 8.95E-04 | *5.49E-03* |
| **C1QL4** | ENSG00000186897 | 0.90 | 0.213834147 | 4.19193252 | 2.77E-05 | *3.37E-04* |
| **C1QTNF8** | ENSG00000184471 | -2.34 | 0.380782252 | -6.14483353 | 8.00E-10 | *5.47E-08* |
| **C1QTNF9B** | ENSG00000205863 | 0.76 | 0.1778847 | 4.252991931 | 2.11E-05 | *2.69E-04* |
| **CCL11** | ENSG00000172156 | 0.82 | 0.23373277 | 3.513727648 | 4.42E-04 | *3.14E-03* |
| **CCL24** | ENSG00000106178 | -1.37 | 0.206628656 | -6.642482701 | 3.08E-11 | *3.26E-09* |
| **CCL7** | ENSG00000108688 | 0.84 | 0.213732309 | 3.930075363 | 8.49E-05 | *8.31E-04* |
| **CELA3A** | ENSG00000142789 | -1.07 | 0.256600865 | -4.184796513 | 2.85E-05 | *3.45E-04* |
| **CELA3B** | ENSG00000219073 | -0.88 | 0.211955628 | -4.163677249 | 3.13E-05 | *3.72E-04* |
| **CHRD** | ENSG00000090539 | 0.94 | 0.153169658 | 6.107394376 | 1.01E-09 | *6.69E-08* |
| **CLC** | ENSG00000105205 | 1.18 | 0.352626249 | 3.339332362 | 8.40E-04 | *5.22E-03* |
| **CLEC3B** | ENSG00000163815 | -0.62 | 0.149127029 | -4.154749563 | 3.26E-05 | *3.83E-04* |
| **CPAMD8** | ENSG00000160111 | 0.62 | 0.167543 | 3.67187126 | 2.41E-04 | *1.91E-03* |
| **CRHBP** | ENSG00000145708 | 0.64 | 0.168703158 | 3.814501473 | 1.36E-04 | *1.22E-03* |
| **CRLF1** | ENSG00000006016 | 0.63 | 0.199303338 | 3.142721432 | 1.67E-03 | *9.06E-03* |
| **CSH2** | ENSG00000213218 | 1.75 | 0.528849925 | 3.308811412 | 9.37E-04 | *5.69E-03* |
| **CST1** | ENSG00000170373 | -1.15 | 0.311798027 | -3.682145711 | 2.31E-04 | *1.85E-03* |
| **CST4** | ENSG00000101441 | -1.96 | 0.3263501 | -6.009065332 | 1.87E-09 | *1.13E-07* |
| **CTSV** | ENSG00000136943 | -0.77 | 0.170910234 | -4.479858962 | 7.47E-06 | *1.14E-04* |
| **EGFL6** | ENSG00000198759 | 0.66 | 0.177444375 | 3.70358289 | 2.13E-04 | *1.73E-03* |
| **ELANE** | ENSG00000197561 | -0.70 | 0.220080806 | -3.184285666 | 1.45E-03 | *8.10E-03* |
| **ELFN2** | ENSG00000166897 | 1.60 | 0.230981495 | 6.930245853 | 4.20E-12 | *5.50E-10* |
| **EREG** | ENSG00000124882 | 0.84 | 0.24550169 | 3.424757599 | 6.15E-04 | *4.08E-03* |
| **F2** | ENSG00000180210 | 0.77 | 0.23268753 | 3.326860897 | 8.78E-04 | *5.41E-03* |
| **FAM20A** | ENSG00000108950 | 0.67 | 0.156363204 | 4.288899683 | 1.80E-05 | *2.37E-04* |
| **FGF11** | ENSG00000161958 | 0.95 | 0.166820089 | 5.689053495 | 1.28E-08 | *5.79E-07* |
| **FGF12** | ENSG00000114279 | 1.13 | 0.177851576 | 6.362745714 | 1.98E-10 | *1.65E-08* |
| **FGF14** | ENSG00000102466 | -1.33 | 0.237817937 | -5.577247641 | 2.44E-08 | *9.85E-07* |
| **FGF22** | ENSG00000070388 | -0.63 | 0.17559937 | -3.580123167 | 3.43E-04 | *2.54E-03* |
| **FLG** | ENSG00000143631 | 0.95 | 0.221368604 | 4.284278687 | 1.83E-05 | *2.41E-04* |
| **FLG2** | ENSG00000143520 | 1.17 | 0.317328281 | 3.683920722 | 2.30E-04 | *1.84E-03* |
| **FREM1** | ENSG00000164946 | 0.62 | 0.146921175 | 4.201690625 | 2.65E-05 | *3.25E-04* |
| **FSTL3** | ENSG00000070404 | 0.60 | 0.122331754 | 4.889381678 | 1.01E-06 | *2.19E-05* |
| **GDF3** | ENSG00000184344 | -0.87 | 0.196289446 | -4.450014569 | 8.59E-06 | *1.29E-04* |
| **GDF5** | ENSG00000125965 | -1.05 | 0.18081128 | -5.791213182 | 6.99E-09 | *3.49E-07* |
| **GDF6** | ENSG00000156466 | 1.05 | 0.237102838 | 4.441567429 | 8.93E-06 | *1.33E-04* |
| **GPC3** | ENSG00000147257 | 1.07 | 0.196079934 | 5.47828656 | 4.29E-08 | *1.59E-06* |
| **GPC5** | ENSG00000179399 | 1.90 | 0.244603606 | 7.774284522 | 7.59E-15 | *2.05E-12* |
| **GREM1** | ENSG00000166923 | 0.77 | 0.167987948 | 4.601593578 | 4.19E-06 | *7.13E-05* |
| **HGF** | ENSG00000019991 | 0.60 | 0.152344431 | 3.920329417 | 8.84E-05 | *8.59E-04* |
| **HHIP** | ENSG00000164161 | -0.62 | 0.194504591 | -3.18164769 | 1.46E-03 | *8.16E-03* |
| **HMSD** | ENSG00000221887 | 0.71 | 0.216704848 | 3.265338305 | 1.09E-03 | *6.42E-03* |
| **HPSE** | ENSG00000173083 | 0.66 | 0.130809448 | 5.012366588 | 5.38E-07 | *1.30E-05* |
| **HRNR** | ENSG00000197915 | 0.67 | 0.195928553 | 3.414210067 | 6.40E-04 | *4.21E-03* |
| **IFNB1** | ENSG00000171855 | 1.10 | 0.287325836 | 3.833603729 | 1.26E-04 | *1.14E-03* |
| **IFNE** | ENSG00000184995 | 0.86 | 0.255627271 | 3.360546042 | 7.78E-04 | *4.90E-03* |
| **IGF1** | ENSG00000017427 | -0.69 | 0.183422964 | -3.742118693 | 1.82E-04 | *1.54E-03* |
| **IGF2** | ENSG00000167244 | 1.73 | 0.240420208 | 7.214313861 | 5.42E-13 | *8.52E-11* |
| **IHH** | ENSG00000163501 | -1.52 | 0.269599699 | -5.656315496 | 1.55E-08 | *6.72E-07* |
| **IL11** | ENSG00000095752 | 1.21 | 0.164472277 | 7.38338661 | 1.54E-13 | *3.02E-11* |
| **IL13** | ENSG00000169194 | -0.75 | 0.223063516 | -3.368633012 | 7.55E-04 | *4.79E-03* |
| **IL19** | ENSG00000142224 | -0.89 | 0.230027331 | -3.871531321 | 1.08E-04 | *1.01E-03* |
| **IL2** | ENSG00000109471 | -1.51 | 0.301216848 | -5.022363999 | 5.10E-07 | *1.25E-05* |
| **IL5** | ENSG00000113525 | 1.00 | 0.206958311 | 4.850861886 | 1.23E-06 | *2.56E-05* |
| **IL6** | ENSG00000136244 | 1.51 | 0.187363009 | 8.048611458 | 8.37E-16 | *2.92E-13* |
| **INHBC** | ENSG00000175189 | 0.63 | 0.160184849 | 3.947861426 | 7.89E-05 | *7.81E-04* |
| **INHBE** | ENSG00000139269 | 0.62 | 0.182738208 | 3.368993264 | 7.54E-04 | *4.79E-03* |
| **ISM2** | ENSG00000100593 | 0.78 | 0.167530631 | 4.629665135 | 3.66E-06 | *6.37E-05* |
| **ITIH2** | ENSG00000151655 | -0.86 | 0.230059259 | -3.720779363 | 1.99E-04 | *1.64E-03* |
| **ITLN1** | ENSG00000179914 | 0.87 | 0.241342634 | 3.599655406 | 3.19E-04 | *2.39E-03* |
| **KNG1** | ENSG00000113889 | 2.22 | 0.290091798 | 7.653530732 | 1.96E-14 | *4.88E-12* |
| **LGALS14** | ENSG00000006659 | 1.19 | 0.359131606 | 3.320453801 | 8.99E-04 | *5.51E-03* |
| **LIF** | ENSG00000128342 | 1.24 | 0.162171901 | 7.632700774 | 2.30E-14 | *5.56E-12* |
| **LMAN1L** | ENSG00000140506 | -1.15 | 0.351418965 | -3.283431062 | 1.03E-03 | *6.09E-03* |
| **LOXL4** | ENSG00000138131 | 0.59 | 0.154628338 | 3.801706351 | 1.44E-04 | *1.27E-03* |
| **MASP1** | ENSG00000127241 | -0.62 | 0.178774033 | -3.482722443 | 4.96E-04 | *3.43E-03* |
| **MEGF10** | ENSG00000145794 | 1.03 | 0.180260926 | 5.706039523 | 1.16E-08 | *5.35E-07* |
| **MMP1** | ENSG00000196611 | 1.05 | 0.196674825 | 5.32243947 | 1.02E-07 | *3.29E-06* |
| **MMP10** | ENSG00000166670 | 1.14 | 0.247072117 | 4.618251034 | 3.87E-06 | *6.67E-05* |
| **MMP13** | ENSG00000137745 | 1.12 | 0.261834017 | 4.264414334 | 2.00E-05 | *2.59E-04* |
| **MMP26** | ENSG00000167346 | -3.07 | 0.380482875 | -8.073805582 | 6.81E-16 | *2.43E-13* |
| **MUC13** | ENSG00000173702 | -0.88 | 0.241706379 | -3.627877771 | 2.86E-04 | *2.19E-03* |
| **MUC5AC** | ENSG00000215182 | -0.90 | 0.259160904 | -3.458478783 | 5.43E-04 | *3.69E-03* |
| **MUC5B** | ENSG00000117983 | -1.13 | 0.276412988 | -4.102351807 | 4.09E-05 | *4.62E-04* |
| **MUC6** | ENSG00000184956 | -0.91 | 0.262103528 | -3.467731406 | 5.25E-04 | *3.59E-03* |
| **NRG3** | ENSG00000185737 | 1.82 | 0.265514966 | 6.845433962 | 7.62E-12 | *9.19E-10* |
| **NTF3** | ENSG00000185652 | 0.62 | 0.18800273 | 3.289573572 | 1.00E-03 | *5.98E-03* |
| **NTF4** | ENSG00000225950 | 0.67 | 0.213523566 | 3.138298781 | 1.70E-03 | *9.17E-03* |
| **OVGP1** | ENSG00000085465 | -1.81 | 0.248640432 | -7.288668923 | 3.13E-13 | *5.36E-11* |
| **PF4V1** | ENSG00000109272 | -1.07 | 0.259288868 | -4.126582377 | 3.68E-05 | *4.23E-04* |
| **PGF** | ENSG00000119630 | 0.75 | 0.128772575 | 5.793479284 | 6.89E-09 | *3.46E-07* |
| **PLG** | ENSG00000122194 | -1.64 | 0.257937161 | -6.343017126 | 2.25E-10 | *1.83E-08* |
| **PRL** | ENSG00000172179 | 0.96 | 0.216817048 | 4.428851123 | 9.47E-06 | *1.40E-04* |
| **PRSS1** | ENSG00000204983 | 1.34 | 0.297240715 | 4.522236404 | 6.12E-06 | *9.77E-05* |
| **PRSS3** | ENSG00000010438 | 1.01 | 0.222953988 | 4.510891055 | 6.46E-06 | *1.02E-04* |
| **PZP** | ENSG00000126838 | -1.76 | 0.240983998 | -7.320204456 | 2.48E-13 | *4.45E-11* |
| **REG3A** | ENSG00000172016 | -2.15 | 0.519803018 | -4.143167422 | 3.43E-05 | *3.99E-04* |
| **S100A3** | ENSG00000188015 | -0.61 | 0.179331694 | -3.374513065 | 7.39E-04 | *4.71E-03* |
| **S100A5** | ENSG00000196420 | 1.58 | 0.202547067 | 7.793788591 | 6.50E-15 | *1.81E-12* |
| **S100A7** | ENSG00000143556 | -1.33 | 0.318837995 | -4.163631954 | 3.13E-05 | *3.72E-04* |
| **SEMA3E** | ENSG00000170381 | -0.84 | 0.233727425 | -3.587700139 | 3.34E-04 | *2.48E-03* |
| **SEMA6D** | ENSG00000137872 | 0.73 | 0.153617038 | 4.762170246 | 1.92E-06 | *3.73E-05* |
| **SERPINA11** | ENSG00000186910 | -1.87 | 0.355863892 | -5.253205415 | 1.49E-07 | *4.52E-06* |
| **SERPINA3** | ENSG00000196136 | -1.27 | 0.263734407 | -4.832820231 | 1.35E-06 | *2.77E-05* |
| **SERPINA5** | ENSG00000188488 | -1.18 | 0.216197525 | -5.457315343 | 4.83E-08 | *1.77E-06* |
| **SERPINA6** | ENSG00000170099 | -1.60 | 0.300207493 | -5.333264147 | 9.65E-08 | *3.14E-06* |
| **SERPIND1** | ENSG00000099937 | -1.16 | 0.22466999 | -5.173461358 | 2.30E-07 | *6.47E-06* |
| **SFRP4** | ENSG00000106483 | -0.94 | 0.226411768 | -4.153200027 | 3.28E-05 | *3.85E-04* |
| **SFTPA1** | ENSG00000122852 | 1.11 | 0.354311662 | 3.128693796 | 1.76E-03 | *9.41E-03* |
| **SFTPB** | ENSG00000168878 | 1.08 | 0.269757237 | 3.992185695 | 6.55E-05 | *6.70E-04* |
| **TDGF1** | ENSG00000241186 | -0.88 | 0.190445573 | -4.644132463 | 3.42E-06 | *6.01E-05* |
| **TGM1** | ENSG00000092295 | 0.72 | 0.144186902 | 4.968490791 | 6.75E-07 | *1.57E-05* |
| **THPO** | ENSG00000090534 | 1.21 | 0.205357691 | 5.899448809 | 3.65E-09 | *2.02E-07* |
| **TLL1** | ENSG00000038295 | 0.63 | 0.15852868 | 3.944131699 | 8.01E-05 | *7.91E-04* |
| **TMPRSS15** | ENSG00000154646 | 0.99 | 0.31095853 | 3.19819773 | 1.38E-03 | *7.78E-03* |
| **TNF** | ENSG00000232810 | 0.61 | 0.178435784 | 3.43955955 | 5.83E-04 | *3.91E-03* |
| **TNFSF10** | ENSG00000121858 | 0.65 | 0.126162387 | 5.118451809 | 3.08E-07 | *8.26E-06* |
| **TNFSF14** | ENSG00000125735 | -0.79 | 0.177980668 | -4.461783299 | 8.13E-06 | *1.23E-04* |
| **TNFSF8** | ENSG00000106952 | -0.96 | 0.177392349 | -5.406690149 | 6.42E-08 | *2.23E-06* |
| **TPO** | ENSG00000115705 | 0.94 | 0.23469373 | 4.025836762 | 5.68E-05 | *6.01E-04* |
| **VWC2L** | ENSG00000174453 | 0.96 | 0.295022273 | 3.23768972 | 1.21E-03 | *6.97E-03* |
| **WFIKKN2** | ENSG00000173714 | 0.92 | 0.208628111 | 4.427244473 | 9.54E-06 | *1.41E-04* |
| **WIF1** | ENSG00000156076 | -1.06 | 0.294597214 | -3.581618662 | 3.41E-04 | *2.53E-03* |
| **WNT10A** | ENSG00000135925 | 1.14 | 0.202023533 | 5.663493238 | 1.48E-08 | *6.52E-07* |
| **WNT3** | ENSG00000108379 | 0.84 | 0.144576836 | 5.842771569 | 5.13E-09 | *2.67E-07* |
| **WNT7A** | ENSG00000154764 | 0.97 | 0.235057512 | 4.135927261 | 3.54E-05 | *4.09E-04* |
| **AGTR1** | ENSG00000144891 | 1.49 | 0.212381094 | 7.025932669 | 2.13E-12 | *2.91E-10* |
| **ATP1A3** | ENSG00000105409 | 1.17 | 0.177154977 | 6.631597435 | 3.32E-11 | *3.48E-09* |
| **AZU1** | ENSG00000172232 | -1.55 | 0.246975292 | -6.287803603 | 3.22E-10 | *2.50E-08* |
| **B3GAT2** | ENSG00000112309 | 0.73 | 0.122698533 | 5.949312491 | 2.69E-09 | *1.55E-07* |
| **BMPR1B** | ENSG00000138696 | -0.59 | 0.14752794 | -3.991280094 | 6.57E-05 | *6.72E-04* |
| **CADM3** | ENSG00000162706 | 1.09 | 0.220498234 | 4.961235111 | 7.00E-07 | *1.62E-05* |
| **CAMK2N2** | ENSG00000163888 | 0.77 | 0.159747591 | 4.796390995 | 1.62E-06 | *3.23E-05* |
| **CASR** | ENSG00000036828 | 1.50 | 0.264514889 | 5.652363698 | 1.58E-08 | *6.84E-07* |
| **CDH17** | ENSG00000079112 | 0.62 | 0.196940363 | 3.162717799 | 1.56E-03 | *8.58E-03* |
| **CDH6** | ENSG00000113361 | 1.04 | 0.187238278 | 5.529208272 | 3.22E-08 | *1.25E-06* |
| **CDH9** | ENSG00000113100 | 1.37 | 0.414242139 | 3.307237068 | 9.42E-04 | *5.72E-03* |
| **CHSY3** | ENSG00000198108 | 0.63 | 0.122253052 | 5.113974669 | 3.15E-07 | *8.42E-06* |
| **CNTN2** | ENSG00000184144 | 0.62 | 0.176701688 | 3.510642913 | 4.47E-04 | *3.16E-03* |
| **COLGALT2** | ENSG00000198756 | -0.96 | 0.175204379 | -5.482447754 | 4.19E-08 | *1.57E-06* |
| **DCSTAMP** | ENSG00000164935 | 0.60 | 0.160926759 | 3.73566544 | 1.87E-04 | *1.57E-03* |
| **DNM3** | ENSG00000197959 | 0.67 | 0.12489936 | 5.349279142 | 8.83E-08 | *2.94E-06* |
| **ERBB2** | ENSG00000141736 | 0.73 | 0.116719923 | 6.212983451 | 5.20E-10 | *3.79E-08* |
| **ERBB4** | ENSG00000178568 | 0.83 | 0.16828681 | 4.930819739 | 8.19E-07 | *1.84E-05* |
| **ESR1** | ENSG00000091831 | -0.84 | 0.14002632 | -6.018093887 | 1.76E-09 | *1.08E-07* |
| **FAP** | ENSG00000078098 | 0.69 | 0.136826624 | 5.025263191 | 5.03E-07 | *1.23E-05* |
| **FGFR4** | ENSG00000160867 | 0.61 | 0.157685448 | 3.896147347 | 9.77E-05 | *9.29E-04* |
| **FLRT3** | ENSG00000125848 | 0.93 | 0.18283603 | 5.096007867 | 3.47E-07 | *9.15E-06* |
| **FZD9** | ENSG00000188763 | 0.81 | 0.152030108 | 5.325712286 | 1.01E-07 | *3.24E-06* |
| **GAD1** | ENSG00000128683 | -0.89 | 0.207520047 | -4.306671603 | 1.66E-05 | *2.23E-04* |
| **GAL3ST3** | ENSG00000175229 | 1.45 | 0.303750546 | 4.784283025 | 1.72E-06 | *3.41E-05* |
| **GAP43** | ENSG00000172020 | 1.70 | 0.200737782 | 8.47299505 | 2.39E-17 | *1.28E-14* |
| **IAPP** | ENSG00000121351 | 1.49 | 0.451390003 | 3.304157789 | 9.53E-04 | *5.76E-03* |
| **ITGB3** | ENSG00000259207 | 1.02 | 0.174905213 | 5.858100713 | 4.68E-09 | *2.47E-07* |
| **ITGB6** | ENSG00000115221 | 0.92 | 0.16507593 | 5.597236919 | 2.18E-08 | *9.00E-07* |
| **JPH3** | ENSG00000154118 | 1.71 | 0.235819435 | 7.258468118 | 3.91E-13 | *6.55E-11* |
| **L1CAM** | ENSG00000198910 | 1.95 | 0.25054558 | 7.796992466 | 6.34E-15 | *1.78E-12* |
| **LIPC** | ENSG00000166035 | 0.79 | 0.168860824 | 4.684483472 | 2.81E-06 | *5.11E-05* |
| **LRRTM4** | ENSG00000176204 | 2.11 | 0.254698818 | 8.269229764 | 1.35E-16 | *5.89E-14* |
| **MAG** | ENSG00000105695 | 1.48 | 0.268201713 | 5.526164199 | 3.27E-08 | *1.26E-06* |
| **MPO** | ENSG00000005381 | 0.64 | 0.168877952 | 3.781976159 | 1.56E-04 | *1.35E-03* |
| **NCAM1** | ENSG00000149294 | 1.21 | 0.192221328 | 6.297479041 | 3.03E-10 | *2.36E-08* |
| **NDP** | ENSG00000124479 | -0.68 | 0.174410401 | -3.87906208 | 1.05E-04 | *9.86E-04* |
| **NDST3** | ENSG00000164100 | 1.93 | 0.257665898 | 7.501483801 | 6.31E-14 | *1.40E-11* |
| **PLA2G10** | ENSG00000069764 | -0.72 | 0.170262217 | -4.234360853 | 2.29E-05 | *2.88E-04* |
| **PRB4** | ENSG00000230657 | 1.73 | 0.44867274 | 3.865938108 | 1.11E-04 | *1.03E-03* |
| **SCG2** | ENSG00000171951 | 0.94 | 0.167617995 | 5.582857485 | 2.37E-08 | *9.62E-07* |
| **SST** | ENSG00000157005 | 2.93 | 0.328693247 | 8.906907104 | 5.25E-19 | *4.90E-16* |
| **SYT1** | ENSG00000067715 | 0.77 | 0.191299806 | 4.033994404 | 5.48E-05 | *5.84E-04* |
| **TMEFF2** | ENSG00000144339 | 1.55 | 0.275941458 | 5.634169574 | 1.76E-08 | *7.47E-07* |
| **TPH1** | ENSG00000129167 | -0.84 | 0.237337683 | -3.529405355 | 4.16E-04 | *2.98E-03* |

log2FoldChange, log2 fold change between the groups

lfcSE, standard error of the log2FoldChange estimate

stat, Wald statistics

p value, Wald test p-value

padj, Benjamini-Hochberg adjusted p-value
